# Palladium-catalyzed three-component radical-polar crossover carboamination of 1,3-dienes or allenes with diazo esters and amines

**DOI:** 10.3762/bjoc.20.59

**Published:** 2024-03-27

**Authors:** Geng-Xin Liu, Xiao-Ting Jie, Ge-Jun Niu, Li-Sheng Yang, Xing-Lin Li, Jian Luo, Wen-Hao Hu

**Affiliations:** 1 Guangdong Key Laboratory of Chiral Molecule and Drug Discovery, School of Pharmaceutical Sciences, Sun Yat-sen University, Guangzhou, Guangdong 510006, China

**Keywords:** carboamination, diazo chemistry, palladium catalysis, radical-polar crossover, three-component reaction

## Abstract

Herein, we report a visible-light-mediated palladium-catalyzed three-component radical-polar crossover carboamination of 1,3-dienes or allenes with diazo esters and amines, affording unsaturated γ- and ε-amino acid derivatives with diverse structures. In this methodology, the diazo compound readily transforms into a hybrid α-ester alkylpalladium radical with the release of dinitrogen. The radical intermediate selectively adds to the double bond of a 1,3-diene or allene, followed by the allylpalladium radical-polar crossover path and selective allylic substitution with the amine substrate, thereby leading to a single unsaturated γ- or ε-amino acid derivative. This approach proceeds under mild and simple reaction conditions and shows high functional group tolerance, especially in the incorporation of various bioactive molecules. The studies on scale-up reactions and diverse derivatizations highlight the practical utility of this multicomponent reaction protocol.

## Introduction

**Scheme 1:**
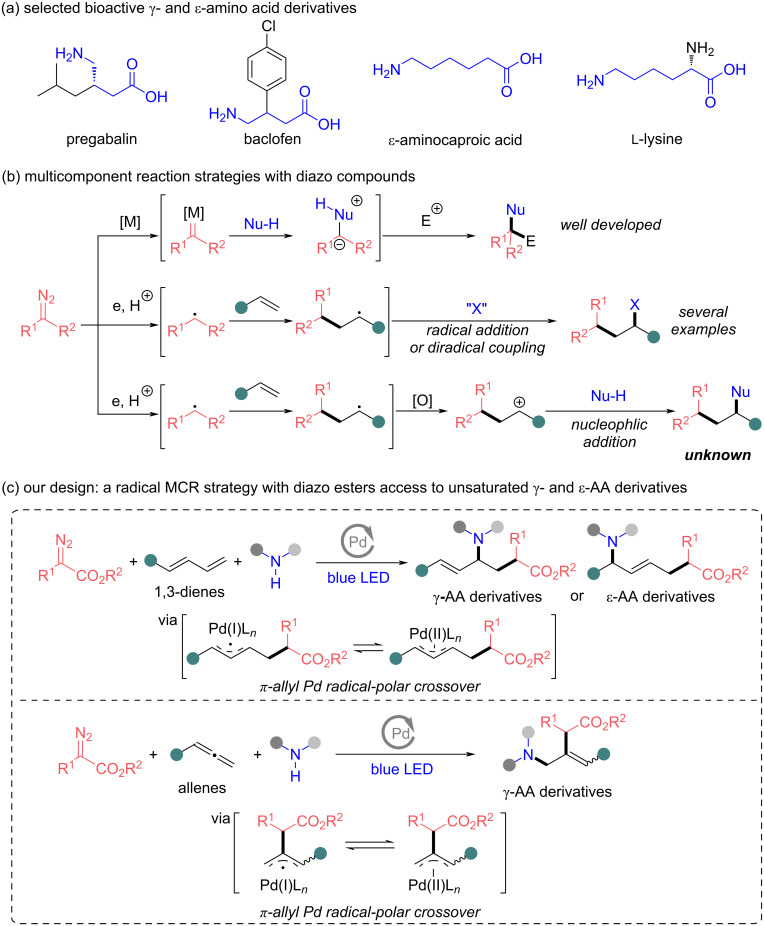
Background (a and b) and proposed carboamination MCR with diazo esters (c). a) Selected bioactive γ- and ε-amino acid derivatives. b) Multicomponent reaction strategies with diazo compounds. c) Our design: a radical MCR strategy with diazo esters to access unsaturated γ- and ε-AA derivatives.

Since the discovery of the existence of non-canonical amino acids (AAs) in organisms, such structural motifs have attracted considerable attention owing to their wide applications in medicinal chemistry [1–5]. γ- and ε-AA derivatives are widely distributed in peptide natural products, bioactive molecules, and drugs, such as pregabalin, baclofen, ε-aminocaproic acid and lysine (Scheme 1a) [6–12]. The number of reported synthetic methods for γ- and ε-AA derivatives is much lower than those of α-AA derivatives [13–14]. Although synthetic strategies of γ- and ε-AA derivatives have been developed [15–20], acquiring complex γ- and ε-AA derivatives with simple starting materials in a one-step reaction remains a challenge. In addition, many studies show that unsaturated AAs exhibit a variety of unique biological activities [21–24]. Accordingly, the development of efficient methods to synthesize unsaturated γ- and ε-AA derivatives is a highly sought-after target to enrich non-natural AA chemistry.

Multicomponent reactions (MCRs) by virtue of high efficiency for the construction of complex chemicals, have shown the superiority in high step and atom economy in organic synthesis [25–27]. Over the past two decades, our group and others have developed a transition-metal-catalyzed MCR strategy involving electrophilic trapping of onium ylides generated from metal carbenes with nucleophiles, providing an ingenious difunctionalization strategy for diazo compounds to access structurally complex and diverse molecules (Scheme 1b, top) [28–29]. In recent years, radical-mediated MCRs with diazo compounds have become a highly emerging area of research and exhibit complementary reactivity to those well-developed carbene-mediated MCRs [30–42]. In the radical-mediated difunctionalization of alkenes, the carbon-centered radical species from a diazo compound can add to diverse alkenes followed by a diradical coupling or radical addition process to achieve the difunctionalization (Scheme 1b, middle) [32–37]. However, to the best of our knowledge, the methodology involving the addition of a carbon radical from a diazo compound onto the double bond of an alkene followed by a nucleophilic addition, is unknown (Scheme 1b, bottom).

The radical-polar crossover strategy has been steadily emerging in synthetic organic chemistry during the last few years [43–46]. This strategy allows complex chemicals to be assembled with high step economy that would be difficult to achieve using either radical or polar chemistry alone. In recent years, Gevorgyan, Glorius, Huang and their co-workers reported elegant examples of the carboamination of 1,3-dienes with unactivated alkyl halides and amines under photoinduced palladium catalysis via a radical-polar crossover process [47–50]. However, activated alkyl halides are not suitable for these carboamination reactions due to the direct nucleophilic substitution of activated alkyl halides with nucleophilic reagents under the necessary alkaline conditions [51]. Recently, a Pd-catalyzed alkyl Heck reaction of diazo compounds mediated by visible light has been reported by the group of Gevorgyan, which achieves the monofunctionalization of alkenes [52]. Inspired by these collective studies, we considered diazo compounds could be a competent activated alkyl halide equivalent to overcome the synthetic limitation of the photoinduced palladium-catalyzed carboamination reactions and the radical-mediated difunctionalization of alkenes with diazo compounds. We envisioned an interesting MCR strategy with mild conditions to access unsaturated γ- and ε-AA derivatives via a π-allyl Pd radical-polar crossover process (Scheme 1c). In this process, the hybrid α-ester alkylpalladium radical species from diazo ester adds to the double bond of 1,3-dienes or allenes, followed by the allylpalladium radical-polar crossover path. As with the classical Tsuji–Trost reaction, a subsequent nucleophilic attack of an amine toward the allylpalladium species would afford the desired unsaturated γ- and ε-AA derivatives. This methodology would represent the first reaction mode for the difunctionalization of alkenes with diazo compounds via a radical-polar crossover process.

## Results and Discussion

As summarized in Table 1, we started our studies with the palladium-catalyzed MCR of ethyl diazoacetate (**1a**), 1,3-butadiene (**2a**), and 1-phenylpiperazine (**3a**) in the presence of 5 mol % Pd(OAc)_2_ and 10 mol % Xantphos as ligand. To our delight, after irradiation with blue LED light in dimethylformamide (DMF) for 12 h at room temperature (rt), the desired unsaturated ε-AA derivative **4a** was obtained in 75% isolated yield (Table 1, entry 1). Isolation and NMR analysis demonstrated that this model reaction provided amino acid **4a** with good *E*-selectivity and excellent regioselectivity (*E/Z* = 91:9, 1,4-/1,2-addition >20:1). Control experiments indicated that ligand, palladium, light and argon atmosphere were necessary for this transformation (Table 1, entries 2–5). Heating conditions could not facilitate the reaction instead of light conditions (Table 1, entry 6). The efficiency was maintained with another Pd(II) catalyst Pd(PPh_3_)_2_Cl_2_ (Table 1, entry 7), whereas only low yields of **4a** were observed with Pd(0) catalysts Pd(PPh_3_)_4_ and Pd_2_(dba)_3_ (Table 1, entries 8 and 9). Moreover, adding potassium carbonate as additive failed to furnish **4a**, demonstrating that the trace amount of acid from the Pd(II) catalyst may facilitate the formation of the hybrid α-ester alkylpalladium radical generated from the diazo ester (Table 1, entry 10) [53]. Replacing Xantphos with *rac*-BINAP or DPEphos gave very low product formation, indicating that the type of ligand was crucial for this transformation (Table 1, entries 11 and 12). Changing the reactant ratio produced the desired product **4a** in 84% yield as optimal conditions for this protocol (Table 1, entry 13).

**Table 1:** Optimization of conditions and control experiments.^a^

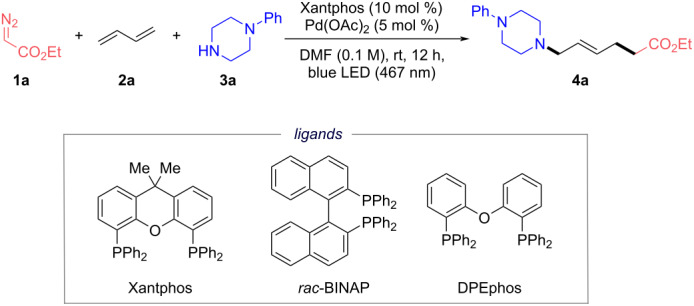		

Entry	Variations	**4a** (%)^b,c^

1	none	75^c^
2	without Xantphos	0
3	without Pd(OAc)_2_	0
4	without blue LED ligt	0
5	without argon protection	0
6	100 °C instead of blue LED light	0
7	Pd(PPh_3_)_2_Cl_2_ instead of Pd(OAc)_2_	70
8	Pd(PPh_3_)_4_ instead of Pd(OAc)_2_	20
9	Pd_2_(dba)_3_ instead of Pd(OAc)_2_	24
10	add K_2_CO_3_ (1.5 equiv)	0
11	*rac*-BINAP instead of Xantphos	<5
12	DPEphos instead of Xantphos	6
13	**1a**/**2a**/**3a** = 0.15:0.2:0.1 mmol	84

^a^Reactions (**1a**/**2a**/**3a**/Pd(OAc)_2_/Xantphos = 0.12:0.12:0.1:0.005:0.01 mmol) were irradiated with blue LED light (467 nm) in 1.0 mL DMF at rt for 12 h under argon. ^b^Yields of compound **4a** were determined by ^1^H NMR spectroscopic analyses of the reaction mixture using 1,3,5-trimethoxybenzene as the internal standard or detected by LC–MS. ^c^The crude NMR yield was consistent with the isolated yield (for more details, see the Supporting Information File 1).

**Scheme 2:**
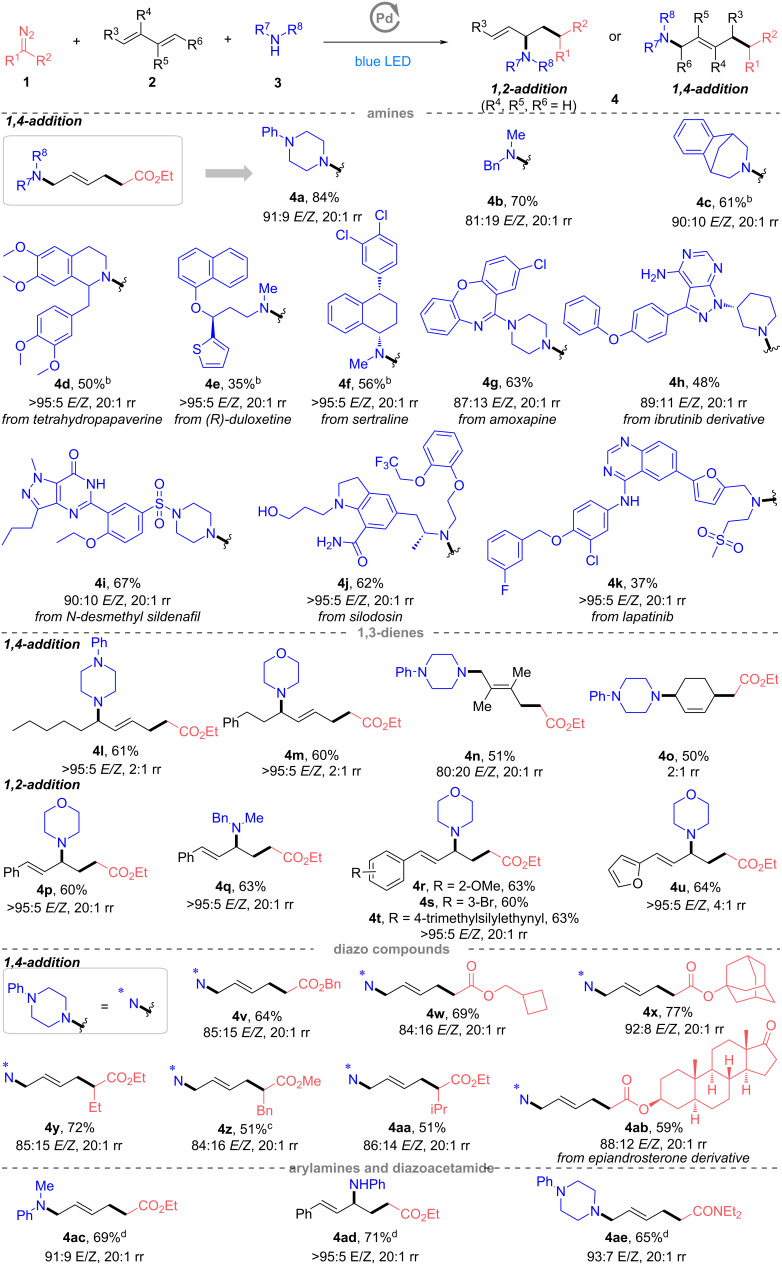
Substrate scope of diazo compounds, 1,3-dienes and amines. ^a^Reactions (**1**/**2**/**3**/Pd(OAc)_2_/Xantphos = 0.3:0.4:0.2:0.01:0.02 mmol) were irradiated with blue LED light (467 nm) in 2.0 mL DMF at rt for 12 h under argon. Isolated yields. ^b^Amine hydrochloride and Et_3_N (1.5 equiv) were used. ^c^Diazo compound (0.4 mmol) was used. ^d^Pd(Ph_3_P)_2_Cl_2_ was used. For more experimental details, see Supporting Information File 1.

With the optimized conditions obtained, we examined the generality of our palladium-catalyzed regioselective carboamination of 1,3-dienes with diazo esters and amines (Scheme 2). First, different alkylamines with various functional groups were evaluated under the optimized conditions, successfully delivering the corresponding 1,4-difunctionalized products in moderate to excellent yields (**4a**–**k**, 35–84%) with high regioselectivity. Some simple secondary amines including cyclic amines **3a**, **3c** and linear amine **3b** were found to readily participate in this protocol, furnishing the corresponding products **4a**–**c** in 61–84% yields. To our delight, this MCR strategy was compatible with a wide variety of complex bioactive molecules, including tetrahydropapaverine, (*R*)-duloxetine, sertraline, amoxapine, an ibrutinib derivative, *N*-desmethyl sildenafil, silodosin, and lapatinib (**4d**–**k**, 35–67%). The late-stage modification of these drug agents and their derivatives in this MCR underlined the synthetic value and high functional group tolerance (e.g., aromatic amine, amide, alcohol, heterocycle).

We next turned to evaluate the scope of 1,3-dienes. Although the regioselectivity control of allylic substitution can be attributed to many factors, it is agreed that steric hindrance generally is the primary factor affecting the regioselectivity of nucleophilic attack [54–57]. Monoalkyl-substituted dienes **2b** and **2c** were suitable for this MCR, affording the 1,4-addition products **4l** and **4m** albeit with moderate regioselectivity (1,4-/1,2-addition = 2:1). To our delight, the reactions with 2,3-disubstituted diene **2d** and 1,4-disubstituted diene **2e** also readily provided products **4n** and **4o**. In the case of 1,3-cyclohexadiene **2e**, the amine was expected to attack the π-allyl palladium from the exo side. Considering that substituent effects might affect the regioselectivity in this MCR, we further investigated the 1,4-/1,2-addition selectivity with 1-phenyl-substituted 1,3-dienes **2f**–**i**. Interestingly, the corresponding 1,2-addition products **4p**–**t** were formed with high selectivity (*E/Z* > 20:1, 1,2-/1,4-addition >20:1), presumably due to steric hindrance by the phenyl group. Furthermore, the 1,3-diene bearing a 1-furan group with smaller steric hindrance afforded product **4u** with moderate chemoselectivity (1,2-/1,4-addition = 4:1).

Diazo esters suitable for this transformation were examined next. The MCRs with diazo substrates equipped with different substitution patterns were accommodated under the mild photocatalytic conditions to generate the desired 1,4-addition products in moderate to good yields (**4v**–**ab**, 51–77%). α-Diazo esters with benzyl, cyclobutanemethyl, and adamantyl groups could be transformed smoothly to the products **4v**, **4w**, and **4x** in 64%, 69% and 77% yields, respectively. Gratifyingly, except for acceptor-substituted diazo esters, donor/acceptor-substituted diazo compounds were also compatible with these mild conditions (**4y**–**aa**, 51–72%). Additionally, the diazo derivative of epiandrosterone was reactive in this protocol, giving the product **4ab** in 59% yield.

Delightedly, this procedure was successfully applied to aromatic amine (*N*-methylaniline), primary amine (aniline) and diazoacetamide, affording the corresponding products **4ac**, **4ad**, and **4ae** in high yields with Pd(PPh_3_)_2_Cl_2_ (69%, 71% and 65%, respectively).

**Scheme 3:**
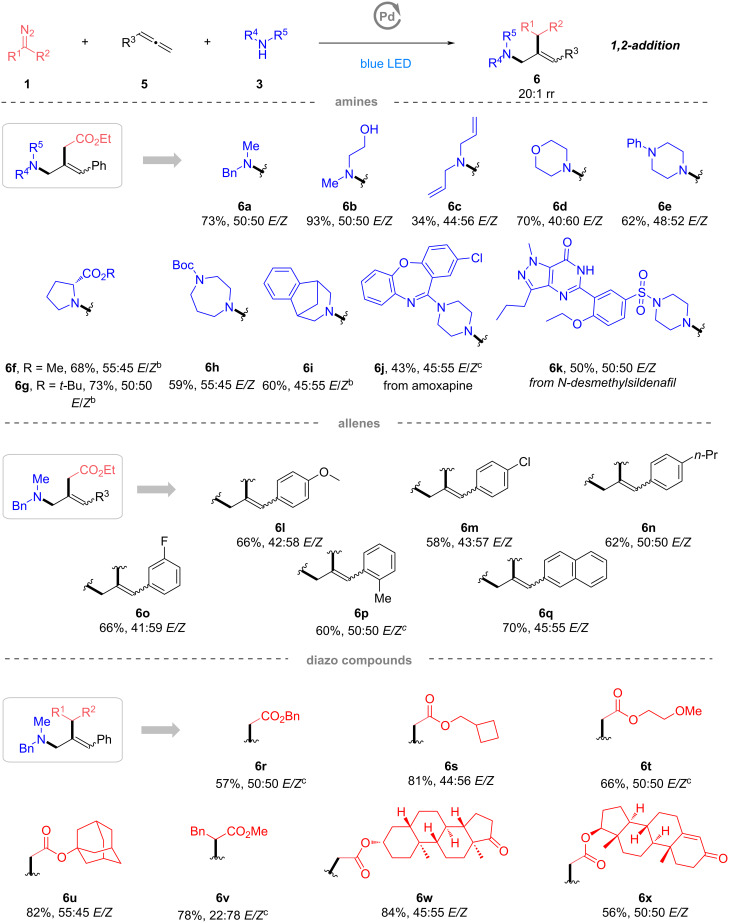
Substrate scope of diazo compounds, allenes and amines. ^a^Reactions (**1**/**5**/**3**/Pd(OAc)_2_/Xantphos = 0.3.0.4:0.2:0.01:0.02 mmol) were irradiated with blue LED light (467 nm) in 2.0 mL DMF at rt for 12 h under argon. Isolated yields. ^b^Amine hydrochloride and Et_3_N (1.5 equiv) were used. ^c^Diazo compound (0.4 mmol) was used. For more experimental details, see Supporting Information Information File 1.

With a reliable set of conditions for the carboamination of 1,3-dienes with diazo esters and amines, we wondered whether this three-component reaction could be applied to allenes that were never used as substrates in interrupted radical Heck/allylic substitution reactions. As summarized in Scheme 3, unsaturated γ-AA derivatives were observed in this reaction albeit with poor stereoselectivity. Linear amines containing alkyl, hydroxy, and terminal alkenyl groups were reactive under the photocatalytic conditions, providing the corresponding 1,2-adducts **6a**, **6b**, and **6c** smoothly in 73%, 93% and 34% yields, respectively. Commercially available amines with a broad range of heterocyclic rings (e.g., morpholine, piperazine, pyrrolidine, homopiperazine) also readily participated in this MCR, affording the products in moderate to good yields (**6d–k**, 43–73%).

Then, the investigations of the scope of allenes demonstrated that the substrates possessing substituents at *para*-, *meta*-, and *ortho*-positions of the aromatic ring were also tolerated under our catalysis conditions. *Para*-(methoxy, chloro, *n*-propyl), *meta*-fluoro, *ortho*-methyl and β-naphthyl-substituted allenes delivered the 1,2-adducts **6l**–**q** in 58–70% yields, indicating a weak influence of different electronic groups on the aromatic ring.

We further assessed the reaction applicability with respect to diazo esters. 1,2-Adducts could be produced fluently with diazo substrates containing alkyl-substituted esters. Benzyl- (**6r**, 57%), cyclobutanemethyl- (**6s**, 81%), methoxyethyl- (**6t**, 66%), and adamantyl- (**6u**, 82%) substituted diazo esters underwent this photoinitiated radical reaction well. The donor/acceptor-substituted diazo compounds with benzyl- and ester groups were also compatible with this MCR system (**6v**, 78%). Furthermore, the successful transformation of the diazo compounds derived from epiandrosterone (**6w**, 84%) and testosterone (**6x**, 56%) highlighted the general utility of this reaction in the modification of pharmaceutical scaffolds.

**Scheme 4:**
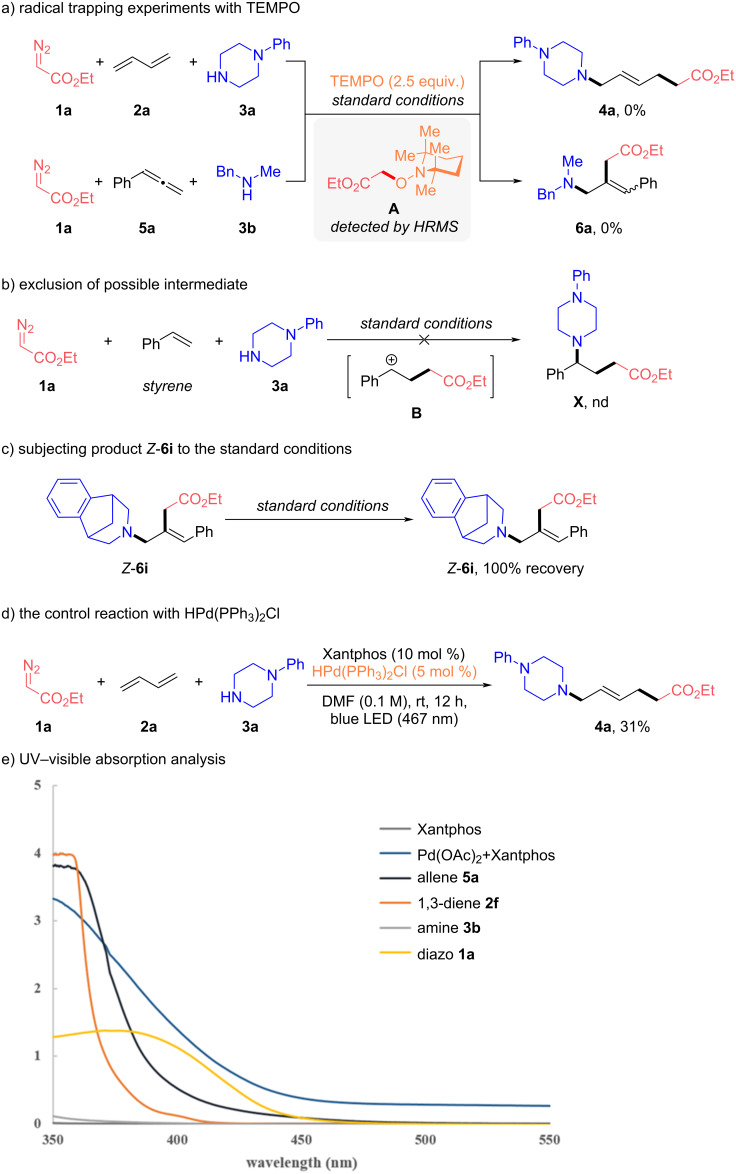
Mechanistic experiments. a) Radical trapping experiments with TEMPO. b) Exclusion of possible intermediate. c) Subjecting the product *Z*-**6i** to the standard conditions. d) The control reaction with HPd(PPh_3_)_2_Cl. e) UV–visible absorption analysis.

Naturally, we were eager to acquire detailed mechanistic insights into this protocol. To validate the radical nature of this transformation, both model reactions of 1,3-diene **2a** and allene **5a** were terminated completely with 2.5 equiv 2,2,6,6-tetramethylpiperidinyloxyl (TEMPO) and the corresponding radical-trapping product **A** could be confirmed by HRMS of both reaction mixtures, unambiguously supporting radical mechanisms (Scheme 4a). The reaction with styrene was conducted under standard conditions, but no product **X** could be detected, indicating the cationic intermediate **B** should be ruled out from this methodology (Scheme 4b). The product *Z*-**6i** was subjected to the standard conditions, but *Z*-**6i** was obtained in 100% recovery yield. Therefore, the *E/Z* selectivity of the MCRs with allenes could be determined by the allylic substitution process (Scheme 4c). Using HPd(PPh_3_)_2_Cl as catalyst, the model reaction also afforded the corresponding product **4a** in 31% yield, demonstrating the H–Pd(II)–X species could be a possible catalytic species (Scheme 4d). According to the UV–visible spectra, the only absorbing species at 467 nm consists in the pre-catalytic system Pd(OAc)_2_ and Xantphos (Scheme 4e). In addition, deuterium labeling experiments were conducted to investigate the H-source of this transformation (for more details, see Supporting Information Information File 1). The isotopic-labeling experiments suggested that both types of protons from the N–H bond of the amine and the traces amount of water in this reaction system may serve as proton sources for the formation of hybrid α-ester alkylpalladium radical.

**Scheme 5:**
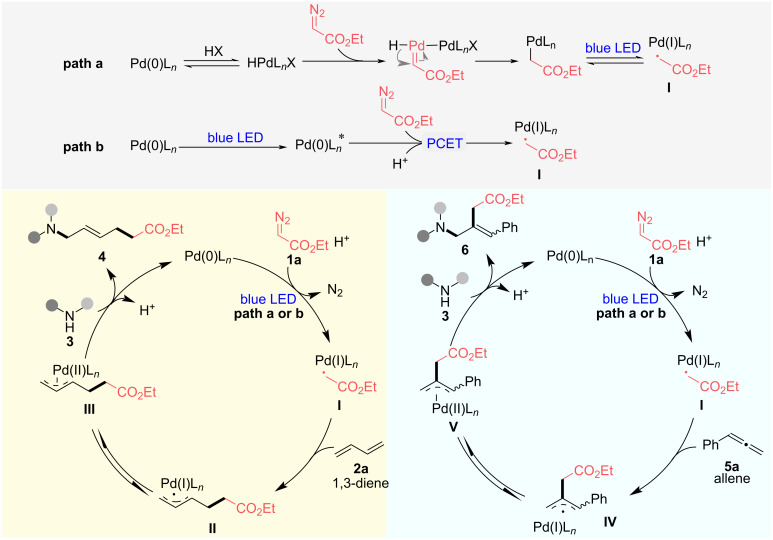
Proposed mechanisms for the carboamination of 1,3-dienes or allenes with diazo esters and amines.

On the basis of above mechanistic studies and previous reports [47–50,52,58], the following plausible mechanisms are proposed for the palladium-catalyzed carboamination of 1,3-dienes (Scheme 5, lower left) or allenes (Scheme 5, lower right) with diazo esters. There are two possible paths to generate the hybrid α-ester alkylpalladium radical **I**. Path a undergoes an oxidative addition of HX with Pd(0)L*_n_*, followed by the formation of Pd–carbene species, hydride shift process, and photoinduced homolytic cleavage of the C–Pd bond, furnishing hybrid α-ester alkylpalladium radical **I**. In path b, upon irradiation with blue light, photoexcited Pd(0)L_n_* reduces ethyl diazoacetate (**1a**) to Pd-radical species **I** by a proton-coupled electron transfer (PCET) process [32–37,59–62], upon the loss of dinitrogen. The radical **I** further adds to the terminal position of 1,3-butadiene (**2a**) to produce hybrid allylPd radical **II**, which would exist in equilibrium with π-allyl complex **III**. Following the classical Tsuji–Trost reaction mechanism, a subsequent attack of amine **3** at the latter stage would afford the unsaturated ε-AA derivative **4** and regenerates the Pd(0)L*_n_* to close the catalytic cycle. Different from the reactive site of 1,3-diene, the hybrid alkylPd radical **I** selectively adds to the central position of the allenyl group of allene **5a**, providing another type of hybrid allylPd radical **IV**. After the equilibrium shifting to the π-allyl complex **V**, the unsaturated γ-AA derivative **6** would be obtained with the nucleophilic attack of amine **3**.

**Scheme 6:**
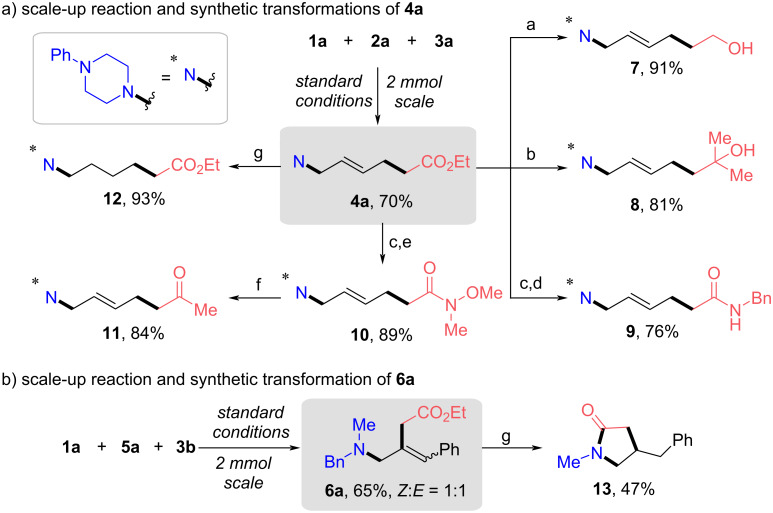
Scale-up reactions and synthetic transformations. Reaction conditions: a) LiAlH_4_, THF, 0 °C; b) MeMgBr, THF, rt to reflux; c) NaOH, MeOH, 60 °C, d) EDCI, DMAP, BnNH_2_, DCM, rt; e) EDCI, DMAP, Et_3_N, HN(OMe)(Me)·HCl, DCM, rt; f) MeMgBr, THF, 0 °C to rt; g) Pd/C, HCO_2_NH_4_, 65 °C. For more details, see Supporting Information File 1.

The utility of this protocol was further highlighted by scale-up reactions and diverse derivatizations of products **4a** and **6a** (Scheme 6). Both model reactions with diene **2a** and allene **5a** were proven to be easily scalable without further conditions optimization, delivering unsaturated γ- and ε-AA derivatives **4a** and **6a** in good yields. Starting from the unsaturated ε-AA derivative **4a**, unsaturated ζ-amino alcohols **7** and **8** were produced in high yields through LiAlH_4_ conditions or nucleophilic addition of methylmagnesium bromide. Moreover, product **4a** could be easily transformed to unsaturated ε-amino amide **9** in total 76% yield. Likewise, Weinreb amide **10** was produced and further transformed into ketone **11** in 84% yield. Compound **4a** could be hydrogenated to the corresponding reduction product **12** using Pd/C and ammonium formate conditions (Scheme 6a). Notably, as shown in Scheme 6b, treatment of the unsaturated γ-AA derivative **6a** with Pd/C and ammonium formate led to a cyclization reaction, furnishing γ-lactam **13** in a moderate yield.

## Conclusion

In summary, we have developed a visible-light-mediated palladium-catalyzed carboamination reaction of 1,3-dienes or allenes with diazo esters and amines, providing a broad array of synthetically valuable unsaturated γ- and ε-AA derivatives. This methodology represents the first reaction mode for a difunctionalization of alkenes with diazo compounds via a radical-polar crossover process. This synthetic transformation proceeds under mild reaction conditions and shows high functional group tolerance. The studies on late-stage functionalization, scale-up reactions, and diverse derivatizations further highlight the practical utility of this MCR protocol.

## Supporting Information

File 1 Full experimental details, analytical data and NMR spetra.

## Data Availability

All data that supports the findings of this study is available in the published article and/or the supporting information to this article.
