# Supplementary material for: Palladium-catalyzed three-component radical-polar crossover carboamination of 1,3-dienes or allenes with diazo esters and amines
Source: Beilstein J Org Chem. 2024 Mar 27;20:661–71. doi: 10.3762/bjoc.20.59 (PMC10999982; doi:10.3762/bjoc.20.59)

BEILSTEIN JOURNAL OF ORGANIC CHEMISTRY

Supporting Information

for

Palladium-catalyzed three-component radical-polar crossover carboamination of 1,3-dienes or allenes with diazo esters and amines

Geng-Xin Liu, Xiao-Ting Jie, Ge-Jun Niu, Li-Sheng Yang, Xing-Lin Li, Jian Luo and Wen-Hao Hu

Beilstein J. Org. Chem. **2024,** *20,* 661–671. doi:10.3762/bjoc.20.59

Full experimental details, analytical data and NMR spetra

License and Terms: This is a supporting information file under the terms of the Creative Commons Attribution License (https://creativecommons.org/licenses/by/4.0). Please note that the reuse, redistribution and reproduction in particular requires that the author(s) and source are credited and that individual graphics may be subject to special legal provisions.
The license is subject to the *Beilstein Journal of Organic Chemistry* terms and conditions: (https://www.beilstein-journals.org/bjoc/terms)

Table of contents

1. General information S2
2. Preparation of starting materials S5
3. General procedure for carboamination of 1,3-dienes or allenes with diazo compounds S9
4. Optimization of the reaction conditions S10
5. Characterization of products S14
6. Scale-up reaction and synthetic transformations S48
7. Mechanistic experiments S55
8. UV–visible absorption analysis S63
9. References S64
10. NMR Spectra of carboamination products S66

1. General information

All reactions were carried out under an inert atmosphere (Ar) unless otherwise stated, with oven-dried glassware using standard techniques. Photocatalysts were purchased from TCI. 1,3-butadiene (**2a**, 15% of 1,3-butadiene is dissolved in *n*-hexane) was purchased from MACKLIN. All commercial reagents are used without further purification. The photoreactor, equipped with a fan to keep the reactor at ambient temperature during the reaction processes, used one blue LED lamps (Kessil PR160L 467 nm, 25% intensity) approximately 1.5 cm away. Analytical thin-layer chromatography (TLC) was performed using glass plates pre-coated with 200–300 mesh silica gel. TLC visualization was performed by fluorescence quenching or iodine stain. Chromatographic purification of products was accomplished using silica gel (300–400 mesh). All NMR spectra were recorded in $CDCl_3$ on a Bruker spectrometer at 400 or 500 MHz (^{1}H NMR), 101 or 126 MHz (^{13}C NMR), and 376 or 471 MHz (^{19}F NMR). Chemical shifts were reported in ppm and calibrated using $CDCl_3$ (7.26 ppm for ^{1}H, 77.16 ppm for ^{13}C) as standards, and coupling constants (*J*) were given in hertz (Hz). The peak information was described as: br = broad, s = singlet, d = doublet, t = triplet, q = quartet, dd = doublet of doublet, m = multiplet, and comp = composite of magnetically non-equivalent protons. HRMS (ESI) and LC–MS (ESI) Mass spectra were recorded on SHIMADZU LCMS-IT-TOF mass spectrometer and Thermo TSQ QUANTUM LC-MS spectrometer, respectively. GC analyses was performed on Agilent 5977B GC-MS. UV–vis absorption spectra were recorded using SHIMADZU UV-2600 ultraviolet–visible spectrophotometer.

1,3-Dienes included in the article

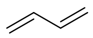

2a

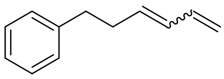

E/Z = 1:1

2b[1]

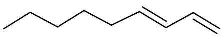

2c[7]

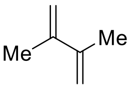

2d

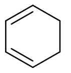

2e

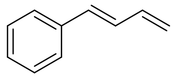

2f[6]

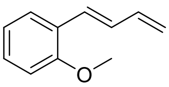

2g[4]

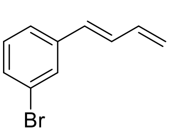

2h[2]

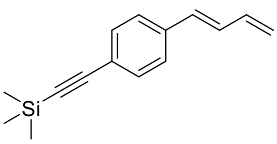

2i[3]

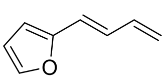

2j[5]

Allenes included in the article

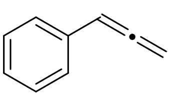

5a[9]

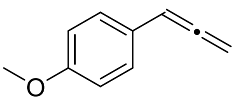

5b[8]

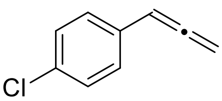

5c[9]

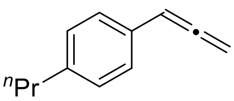

5d[9]

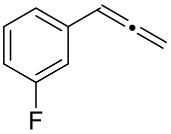

5e[9]

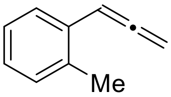

5f[9]

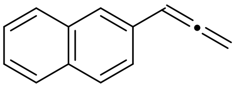

5g[9]

Diazo compounds included in the article

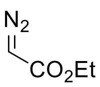

1a[10]

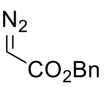

1b[10]

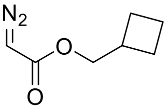

1c[10]

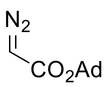

1d[10]

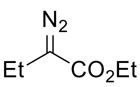

1e[12]

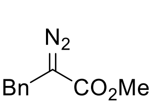

1f[13]

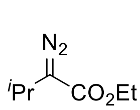

1g[12]

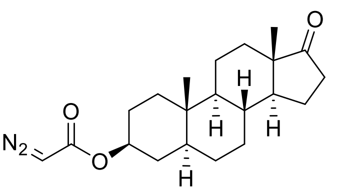

1h[10]

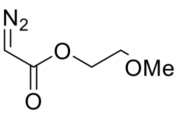

1i[10]

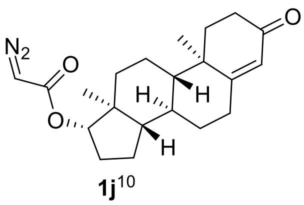

1j[10]

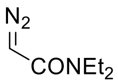

1k[10]

Amines included in the article

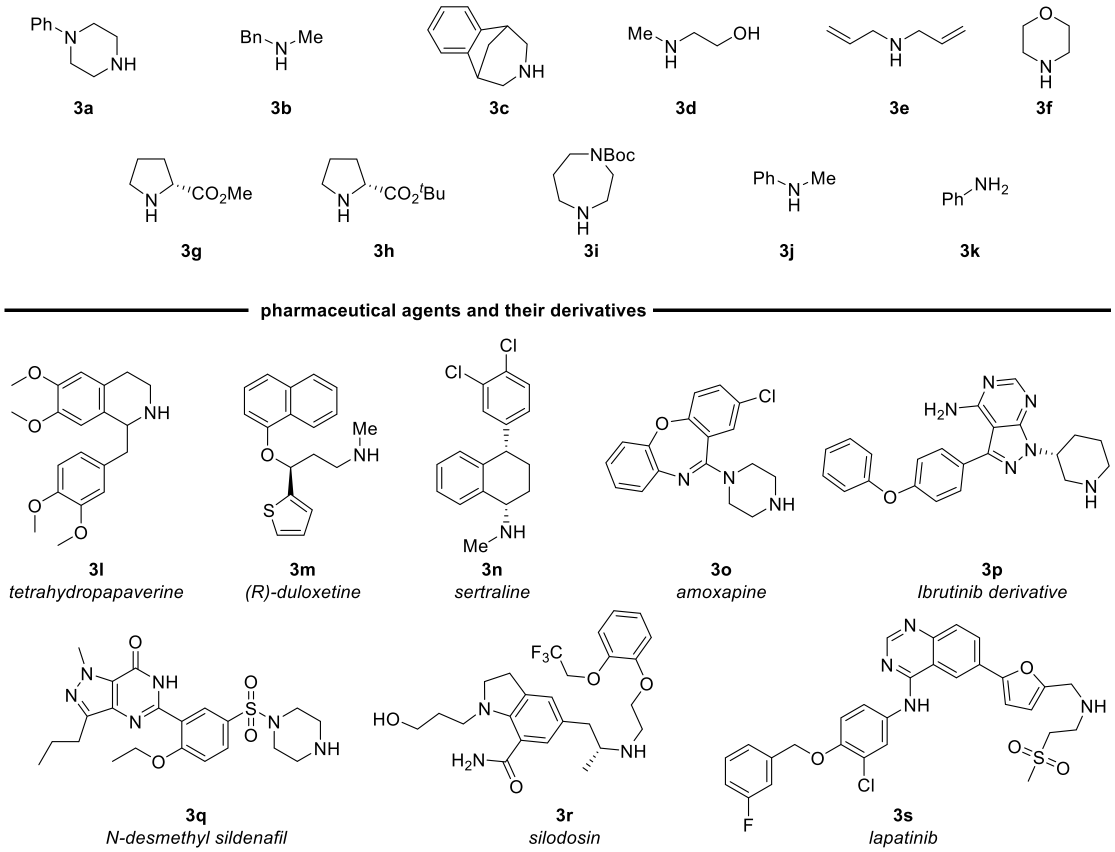

Amines **3a-3s** are commercially available.

2. Preparation of starting materials

2.1 Synthesis of 1,3-dienes

Dienes **2a**, **2d**, and **2e** are commercially available. Dienes **2b**, **2h**, and **2i** were synthesized via Witting reaction using allyltriphenylphosphonium bromide:[1-3]

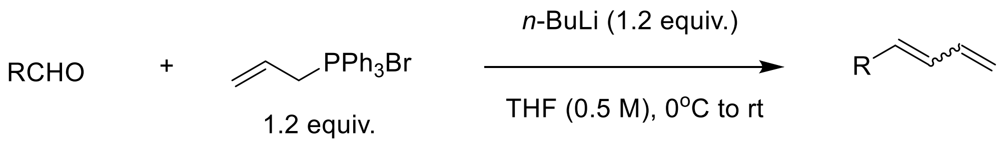

Dienes **2c**, **2f**, **2g**, and **2j** were synthesized via Witting reaction using methyltriphenylphosphonium bromide:[4-7]

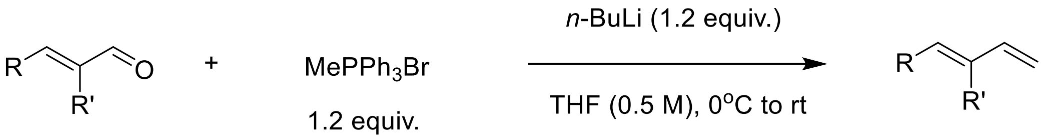

2.2 Synthesis of allenes

General procedure A:

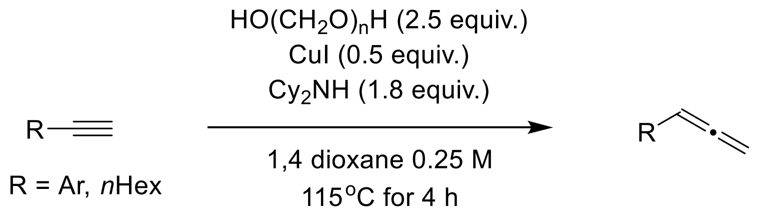

Following a modified procedure[9], the corresponding arylalkyne (1.0 equiv), paraformaldehyde (2.5 equiv), CuI (0.5 equiv) and dicyclohexylamine (1.8 equiv) were dissolved in dry 1,4-dioxane (0.25M) under nitrogen, and the reaction mixture was placed in an oil bath and heated for 4 hours at 115 °C. The reaction was quenched with H_2O (0.5 M), followed by the addition of Et_2O (0.5 M). The organic layer was separated and the aqueous layer was extracted with Et_2O (3 × 0.5 M). The combined organic layers were dried over $MgSO_4$, and the solvents were evaporated under vacuum. Flash chromatography on silica gel using *n*-pentane provided the desired allene in analytically pure form. Allenes **5a**[9], **5b**[8], and **5c**–**g**[9] were prepared using reported procedures. All spectral data were in accordance with those reported in the literature.

2.3 Synthesis of diazo compounds

General procedure B:

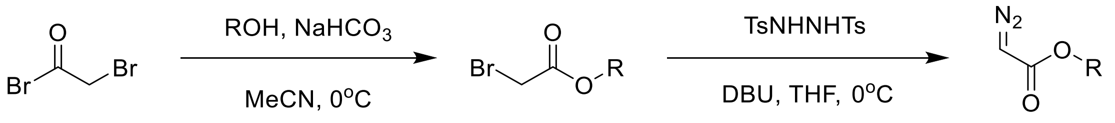

Following reported procedure[10], alcohol (1.0 equiv) and $NaHCO_3$ (5.0 equiv) were dissolved in dry CH_3CN (0.2 M) and bromoacetyl bromide (2.0 equiv) was added slowly at 0 °C and the reaction mixture was stirred for 6 h at room temperature. The reaction was then quenched with water (0.08 M) and the solution was extracted with CH_2Cl_2 (0.01 M). After washing with water (0.04 M) and drying over $MgSO_4$, the solvent was evaporated and the residue was used in the next step without further purification. The resulting crude bromoacetamide and *N,N'*-ditosylhydrazine (2.0 equiv) were dissolved in dry THF (0.2 M) and cooled to 0 °C, then DBU (5.0 equiv) was added dropwise and stirred at room temperature for 1 h. After quenching with saturated solution of $NaHCO_3$ (0.1 M) and extracting with diethyl ether (0.01 M), the organic layer was dried over $MgSO_4$, filtered, and concentrated in vacuo. The resulting crude product was purified by flash chromatography. Diazo compound **1a** is commercially available. Diazo compounds **1b**–**d** and **1h**–**j**[10] were prepared and characterized before.

2-Diazo-*N,N*-diethylacetamide (1l)

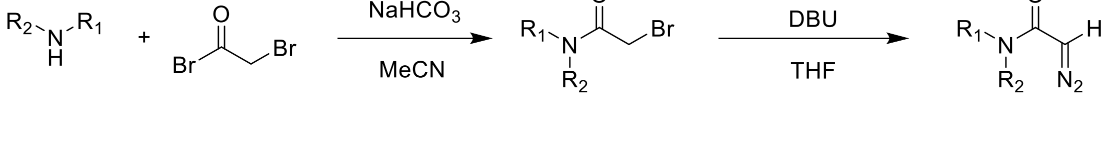

Following a reported procedure[11], amine (1.0 equiv) and $NaHCO_3$ (3.0 equiv) were dissolved in dry CH_2Cl_2 (0.5 M) and bromoacetyl bromide (2.0 equiv) was added

slowly at 0 °C and the reaction was stirred for 6 h at room temperature, quenched with 0.1 M of H_2O and the solution was extracted with CH_2Cl_2 (3 × 0.2 M). The combined organic layers were washed with water (0.1 M) and dried over $MgSO_4$, the solvent was evaporated and the residue was used in the next step without purification. The resulting 2-bromo-*N*,*N*-diethylacetamide and *N,N'*-ditosylhydrazine (0.6 equiv) were dissolved in dry THF (0.2 M) and cooled to 0 °C, then DBU (1.5 equiv) was added dropwise and stirred at room temperature for 1 h and then quenched with saturated solution of $NaHCO_3$ (0.2 M) and extracted with diethyl ether (3 × 0.2 M). The combined organic layers were dried over anhydrous $MgSO_4$. The solvent was removed under reduced pressure and purified by column chromatography. Diazo compounds **1k**[11] was prepared and characterized before.

Ethyl 2-diazobutanoate (1e) and ethyl 2-diazo-3-methylbutanoate (1g)

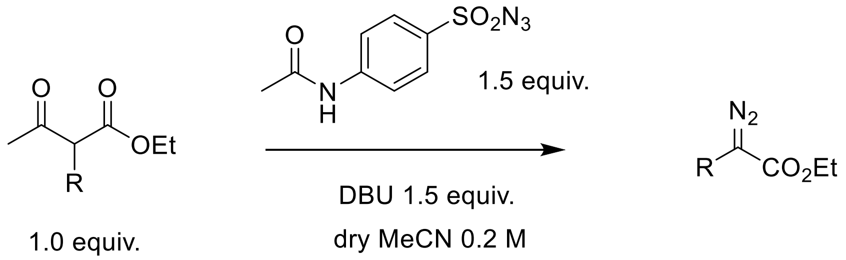

Following a reported procedure[12], to a solution of ester (1.0 equiv) and *p*-acetamidobenzenesulfonyl azide (*p*-ABSA, 1.5 equiv) in dry CH_3CN (0.2 M) was added DBU (1.5 equiv) dropwise at 0 °C. Then, the mixture was stirred at 0 °C for 1 hour and at rt for another hour. To the mixture was added water (0.4 M) and EtOAc (0.2 M). And the aqueous layer was separated and extracted with EtOAc (2 × 0.2 M). The combined organic extracts were dried (Na_2SO_4), filtered and concentrated. The yellow crude product was purified by column chromatography (silica gel, 25 × 200 mm, hexane/EtOAc 15:1) to give the products **1e** and **1g** as yellow liquids (volatile). All spectral data were in accordance with those reported in the literature.

Methyl 2-diazo-3-phenylpropanoate (1f)

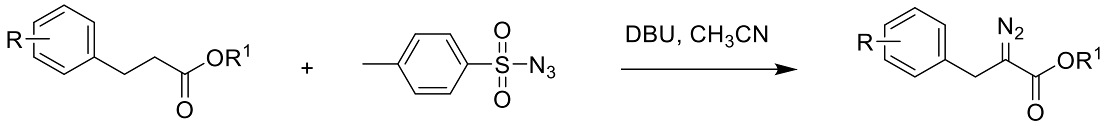

Following reported procedure[13], to a mixture of ester (1.0 equiv) and tosyl azide (1.5 equiv) in anhydrous CH_3CN (0.5 M), DBU (1.5 equiv) was added. The reaction mixture was stirred at rt for 12 h. Subsequently, the reaction mixture was quenched with saturated aqueous solution of NH_4Cl, extracted with ethyl acetate and dried over Na_2SO_4. After removal of the solvent, the crude product was purified by column chromatography (petroleum ether/ethyl acetate, 9:1). Diazo compounds **1f** was prepared and characterized before.

3. General procedure for carboamination of 1,3-dienes or allenes with diazo compounds

General procedure C for carboamination of 1,3-dienes with diazo compounds

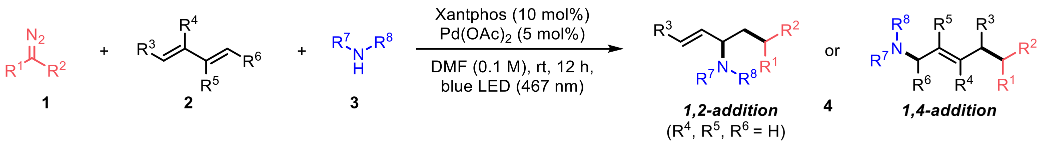

To a reaction tube equipped with a magnetic stirring bar were added $Pd(OAc)_2$ (2.2 mg, 0.01 mmol, 5.0 mol %) and Xantphos (11.6 mg, 0.02 mmol, 10.0 mol %). The tube was capped. After evacuation and backfilling with argon three times, anhydrous DMF (2.0 mL, 0.1 M) was added via a syringe, followed by stirring at room temperature for about 5 minutes. To this reaction system were added 1,3-diene **2** (0.4 mmol, 2.0 equiv), amine **3** (0.2 mmol, 1.0 equiv) and diazo compound **1** (0.3 mmol, 1.5 equiv). The reaction was irradiated by blue LED at room temperature for 12 h until the diazo compound was consumed completely, as monitored by TLC analysis. The crude mixture was purified by column chromatography on silica gel with DCM/MeOH mixtures as eluent to give the corresponding products **4**.

General procedure D for carboamination of allenes with diazo compounds

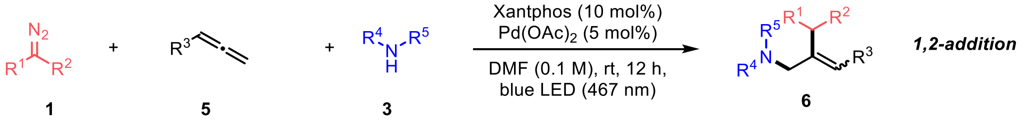

To a reaction tube equipped with a magnetic stirring bar were added $Pd(OAc)_2$ (2.2 mg, 0.01 mmol, 5.0 mol %) and Xantphos (11.6 mg, 0.02 mmol, 10.0 mol %). The tube was capped. After evacuation and backfilling with argon three times, anhydrous DMF (2.0 mL, 0.1 M) was added via a syringe, followed by stirring at room temperature for about 5 minutes. To this reaction system were added allene **5** (0.4 mmol, 2.0 equiv), amine **3** (0.2 mmol, 1.0 equiv) and diazo compound **1** (0.3 mmol, 1.5 equiv). The reaction was irradiated by blue LED at room temperature for 12 h until the diazo compound was consumed completely, as monitored by TLC analysis. The crude mixture was purified by column chromatography on silica gel with PE/EA mixtures as eluent to give the corresponding products **6**.

4. Optimization of the reaction conditions

Table S1. Ligand investigation.[a]

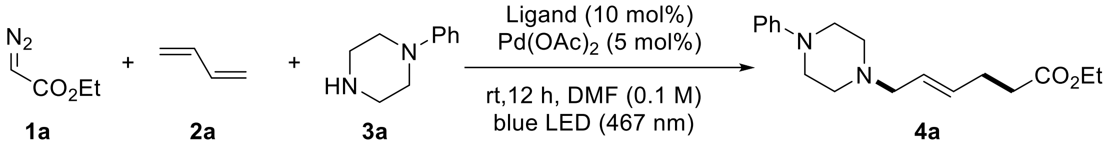

1a 2a 3a 4a

entry	Ligand	**4a** (%)[b]	entry	Ligand	**4a** (%)[b]
1	*rac*-BINAP	<5	7	dppm	0
2[c]	Xantphos	75	8	P(*p*-OMePh)$_3$	0
3	PPh$_3$	<5	9	P(2-furyl)$_3$	0
4	dppf	0	10	*t*-Bu-Xantphos	0
5	DPEphos	6	11	Xantphos, PPh$_3$	74
6	Johnphos	0	12[d]	Xantphos	<5

[a]Reactions (**1a**/**2a**/**3a** = 0.12/0.12/0.1 mmol) were irradiated by blue LED (467 nm) in 1.0 mL DMF at rt for 12 h. [b]Yields of **4a** were determined by ^{1}H NMR spectroscopic analyses of the reaction mixture using 1,3,5-trimethoxybenzene as the internal standard or detected by LC–MS. [c]*E/Z* = 91:9, 1,4-/1,2-addition selectivity >20:1. rt = room temperature. [d] The equivalent of the ligand is 5 mol %.

Table S2. Catalyst investigation[a]

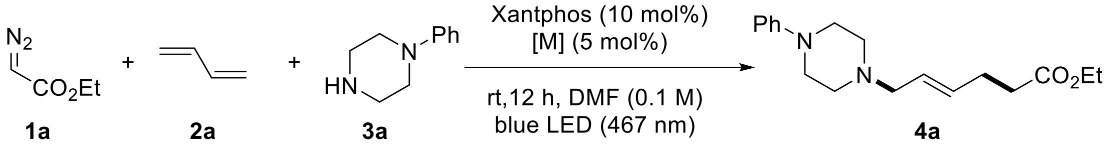

1a 2a 3a 4a

entry	[M]	**4a** (%)[b]	entry	[M]	**4a** (%)[b]
1	Pd(OAc)$_2$	75	10	HPd(PPh$_3$)$_2$Cl	31
2	PdCl$_2$	61	11[e]	Pd(Xantphos)Cl$_2$	70
3	Pd(PPh$_3$)Cl$_2$	70	12[f]	Pd(Xantphos)Cl$_2$	0
4	Pd(TFA)$_2$	35	13	Ni(OAc)$_2$·4H$_2$O	0
5	Pd(PhCN)$_2$Cl$_2$	72	14	Ni(PPh$_3$)$_2$Cl$_2$	0
6	Pd$_2$(dba)$_3$	24	15	NiCl$_2$·3H$_2$O	0
7	Pd(PPh$_3$)$_4$	20	16	NiCl$_2$·glyme	0
8[c]	Pd(OAc)$_2$	64	17	Ni(COD)$_2$	0

9[d]	$Pd(OAc)_2$	74			

[a]Reactions (**1a**/**2a**/**3a** = 0.12/0.12/0.1 mmol) were irradiated by blue LED (467 nm) in 1.0 mL DMF at rt for 12 h. [b]Yields of **4a** were determined by ^{1}H NMR spectroscopic analyses of the reaction mixture using 1,3,5-trimethoxybenzene as the internal standard or detected by LC–MS. [c]2 mol % $Pd(OAc)_2$ and 4 mol % Xantphos. [d]10 mol % $Pd(OAc)_2$, and 20 mol % Xantphos. [e]5 mol % Xantphos. [f]Without adding Xantphos.

Table S3. Solvent investigation[a]

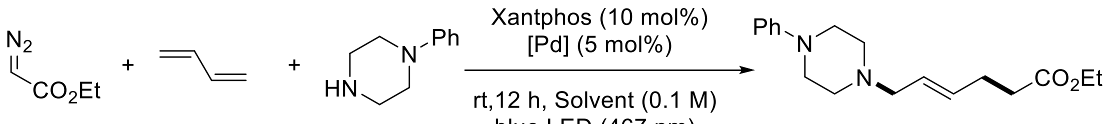

1a 2a 3a 4a

entry	Solvent	**4a** (%)[b]	entry	Solvent	**4a** (%)[b]
1	DMF	75	7	Hexane	<5
2	DMA	69	8	MeOH	<5
3	THF	0	9	Toluene	<5
4	MTBE	24	10	$PhCF_3$	<5
5	DCE	<5	11	PhCl	<5
6	MeCN	<5			

[a]Reactions (**1a**/**2a**/**3a** = 0.12/0.12/0.1 mmol) were irradiated by blue LED (467 nm) in 1.0 mL solvent at rt for 12 h. [b]Yields of **4a** were determined by ^{1}H NMR spectroscopic analyses of the reaction mixture using 1,3,5-trimethoxybenzene as the internal standard or detected by LC–MS.

Table S4. Investigation of reactant ratio, concentration and light source[a]

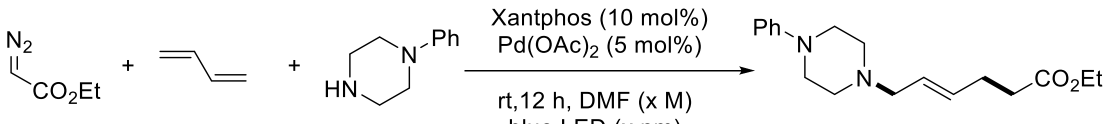

1a 2a 3a 4a

entry	**1a**/**2a**/**3a** (equiv)	conc. (M)	Light	**5a** (%)[b]
1	1.0/1.2/1.2	0.1	Blue LED (467 nm)	63
2	1.0/1.5/1.5	0.1	Blue LED (467 nm)	69
3	1.0/2.0/2.0	0.1	Blue LED (467 nm)	68

5	1.2/1.2/1.0	0.1	Blue LED (467 nm)	75
6	1.5/1.5/1.0	0.1	Blue LED (467 nm)	80
7[c]	1.5/2.0/1.0	0.1	Blue LED (467 nm)	84
8	2.0/2.0/1.0	0.1	Blue LED (467 nm)	75
9	1.5/1.5/1.0	0.05	Blue LED (467 nm)	65
10	1.5/1.5/1.0	0.2	Blue LED (467 nm)	54
11	1.5/1.5/1.0	0.1	Blue LED (427 nm)	77
12	1.5/1.5/1.0	0.1	Blue LED (390 nm)	34

[a]Reactions were irradiated by blue LED (*x* nm) in DMF at rt for 12 h. [b]Yields of **4a** were determined by ^{1}H NMR spectroscopic analyses of the reaction mixture using 1,3,5-trimethoxybenzene as the internal standard or detected by LC–MS. [c]*E*/*Z* = 91:9, 1,4-/1,2-addition selectivity >20:1.

Table S5. Control reactions for the carboamination of 1,3-dienes with diazo compounds[a]

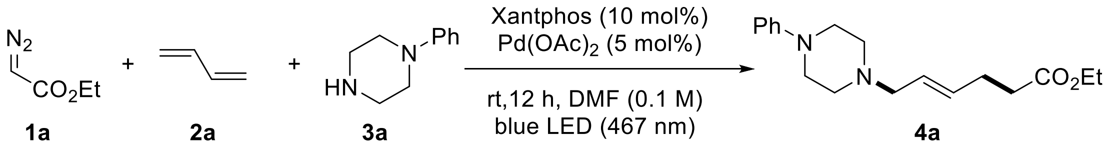

entry	Deviation from standard conditions	**4a** (%)[b]
1	None	84
2	Without Xantphos	0
3	Without $Pd(OAc)_2$	0
4	Without blue LED	0
5	Under air atmosphere	0
6	100°C instead of blue LED	0

[a]Reactions (**1a**/**2a**/**3a** = 0.12/0.12/0.1 mmol) were irradiated by blue LED (467 nm) in 1.0 mL DMF at rt for 12 h. [b]Yields of **4a** were determined by ^{1}H NMR spectroscopic analyses of the reaction mixture using 1,3,5-trimethoxybenzene as the internal standard or detected by LC–MS.

Table S6. Control reactions for the carboamination of allene with diazo compounds[a]

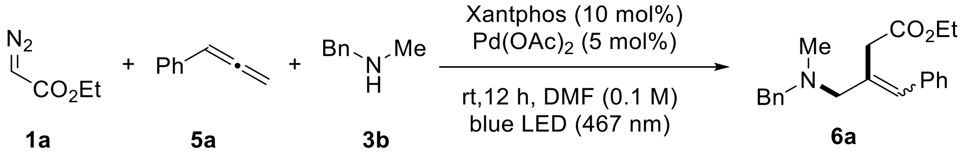

entry	Deviation from standard conditions	**6a** (%)[b]
1[c]	None	73
2	Without Xantphos	0
3	Without $Pd(OAc)_2$	0
4	Without blue LED	0
5	Under air	0
6	100°C instead of blue LED	0

[a]Reactions (**1a**/**2a**/**3a** = 0.12/0.12/0.1 mmol) were irradiated by blue LED (467 nm) in 1.0 mL DMF at rt for 12 h. [b]Yields of **6a** were determined by ^{1}H NMR spectroscopic analyses of the reaction mixture using 1,3,5-trimethoxybenzene as the internal standard or detected by LC–MS. [c]*Z/E* = 1:1, 1,2-/2,3-addition selectivity >20:1.

5. Characterization of products

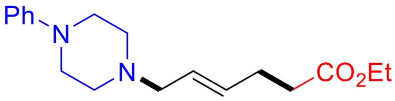

Ethyl (*E*)-6-(4-phenylpiperazin-1-yl)hex-4-enoate (4a)

Following General Procedure C on 0.2 mmol scale. *E:Z* = 91:9, the ratio was determined by GC–MS. Column chromatography on silica gel (eluent: DCM/MeOH 40:1) afforded the title product in 84% yield (50.8 mg) as a pale yellow oil. ^{1}H NMR (400 MHz, Chloroform-*d*) δ 7.27 – 7.29 (m, 2H), 7.04 – 6.71 (m, 3H), 5.83 – 5.56 (m, 2H), 4.13 (q, *J* = 7.3 Hz, 2H), 3.29 – 3.31 (m, 4H), 3.13 (d, *J* = 6.3 Hz, 2H), 2.72 – 2.74 (m, 4H), 2.40 – 2.42 (m, 4H), 1.26 (t, *J* = 7.3 Hz, 3H). ^{13}C NMR (126 MHz, Chloroform-*d*) δ 172.9, 150.9, 134.5, 129.2, 125.5, 120.2, 116.3, 60.4, 60.3, 52.5, 48.6, 33.8, 27.6, 14.3. HRMS (ESI) *m/z*: $[M+H]^+$ Calcd for $C_{18}H_{26}N_2O_2$ 303.2067; Found 303.2070.

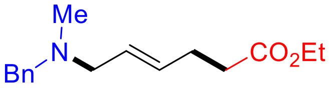

Ethyl (*E*)-6-(benzyl(methyl)amino)hex-4-enoate (4b)

Following General Procedure C on 0.2 mmol scale. *E:Z* = 81:19, the ratio was determined by GC–MS Column chromatography on silica gel (eluent: DCM/MeOH 40:1) afforded the title product in 70% yield (36.6 mg) as a yellow oil. ^{1}H NMR (400 MHz, Chloroform-*d*) δ 7.45 – 7.26 (m, 5H), 5.75 – 5.63 (m, 2H), 4.12 (q, *J* = 7.1 Hz, 2H), 3.69 (s, 2H), 3.21 (dd, *J* = 33.3, 5.9 Hz, 2H), 2.44 – 2.36 (m, 4H), 2.31 (s, 3H), 1.24 (t, *J* = 7.1 Hz, 3H). ^{13}C NMR (101 MHz, $CDCl_3$) δ ^{13}C NMR (101 MHz, CDCl3) δ 172.8, 136.4, 133.7, 130.1, 128.8, 128.5, 123.8, 60.5, 60.0, 58.1, 40.2, 33.6, 27.6, 14.2. HRMS (ESI) *m/z*: $[M+H]^+$ Calcd for $C_{16}H_{23}NO_2$ 262.1802; Found 262.1813.

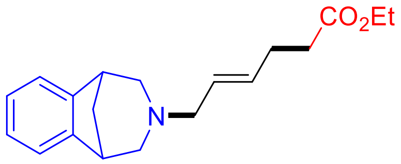

Ethyl (*E*)-6-(1,2,4,5-tetrahydro-3*H*-1,5-methanobenzo[*d*]azepin-3-yl)hex-4-enoate (4c)

Following General Procedure C on 0.2 mmol scale. *E:Z* = 90:10, the ratio was determined by GC–MS Column chromatography on silica gel (eluent: DCM/MeOH 40:1) afforded the title product in 61% yield (36.5 mg) as a yellow oil. ^{1}H NMR (400 MHz, Chloroform-*d*) δ 7.28 – 7.24 (m, 4H), 5.64 – 5.50 (m, 2H), 4.12 (q, *J* = 7.2 Hz, 2H), 3.28 – 3.04 (m, 6H), 2.96 – 2.72 (m, 2H), 2.39 – 2.27 (m, 5H), 1.87 – 1.78 (m, 1H), δ 1.25 (t, *J* = 7.1 Hz, 3H). ^{13}C NMR (101 MHz, $CDCl_3$) δ 172.8, 128.0, 127.8, 123.2, 123.0, 60.4, 60.0, 55.0, 42.7, 39.9, 33.6, 27.5, 14.3. Peaks of ^{13}C on alkenyl group were difficult to observed. HRMS (ESI) *m/z*: $[M+H]^+$ Calcd for $C_{19}H_{25}NO_2$ 300.1958; Found 300.1968.

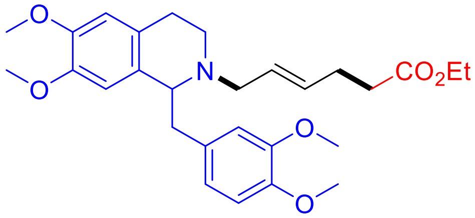

Ethyl (*E*)-6-(1-(3,4-dimethoxybenzyl)-6,7-dimethoxy-3,4-dihydroisoquinolin-2(1*H*)-yl)hex-4-enoate (4d)

Following General Procedure C on 0.2 mmol scale. *E:Z* > 95:5, the ratio was determined by crude ^{1}H NMR. Amine hydrochloride and Et_3N (1.5 equiv) was used. Column chromatography on silica gel (eluent: DCM/MeOH 40:1) afforded the title product in 50% yield (49.0 mg) as a pale yellow oil. ^{1}H NMR (400 MHz, Chloroform-*d*) δ 6.80 – 6.71 (m, 1H), 6.67 (s, 1H), 6.64 – 6.52 (m, 2H), 5.97 – 5.62 (m, 3H), 4.19 – 4.02 (m, 3H), 3.90 – 3.74 (m, 9H), 3.69 – 3.36 (m, 7H), 3.26 – 3.10 (m, 1H), 2.99 – 2.68 (m, 3H), 2.43 – 2.20 (m, 4H), δ 1.25 (t, *J* = 7.5 Hz, 3H). ^{13}C NMR (101 MHz, $CDCl_3$) δ 172.7, 148.8, 148.4, 147.9, 146.8, 122.4, 113.2, 111.3, 111.2, 111.0, 62.6,

60.5, 56.0, 56.0, 55.9, 55.6, 55.3, 42.3, 40.8, 33.6, 27.5, 22.9, 14.3. Peak overlapping was observed. Peaks of ^{13}C on alkenyl group were difficult to observed. HRMS (ESI) *m/z*: $[M+H]^+$ Calcd for $C_{28}H_{37}NO_6$ 484.2694; Found 484.2699.

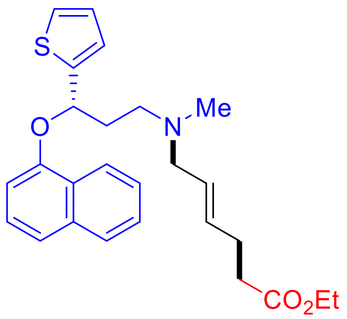

Ethyl (*S*,*E*)-6-(methyl(3-(naphthalen-1-yloxy)-3-(thiophen-2-yl)propyl)amino)hex-4-enoate (4e)

Following General Procedure C on 0.2 mmol scale. *E*:*Z* > 95:5, the ratio was determined by crude ^1H NMR. Amine hydrochloride and Et_3N (1.5 equiv) was used. Column chromatography on silica gel (eluent: DCM/MeOH 40:1) afforded the title product in 35% yield (30.6 mg) as a yellow oil. ^1H NMR (400 MHz, Chloroform-*d*) δ 8.37 – 8.16 (m, 1H), 7.85 – 7.68 (m, 1H), 7.60 – 7.44 (m, 2H), 7.44 – 7.37 (m, 1H), 7.30 – 7.26 (m, 1H), 7.25 – 7.18 (m, 1H), 7.16 – 7.10 (m, 1H), 6.94 (dd, *J* = 5.1, 3.5 Hz, 1H), 6.88 (d, *J* = 7.7 Hz, 1H), 5.96 – 5.85 (m, 1H), 5.84 – 5.58 (m, 2H), 4.10 (q, *J* = 7.1 Hz, 2H), 3.39 (d, *J* = 5.7 Hz, 2H), 3.19 – 2.90 (m, 2H), 2.78 – 2.40 (m, 5H), 2.38 – 2.24 (m, 4H), 1.24 (t, *J* = 7.2 Hz, 3H). ^{13}C NMR (126 MHz, $CDCl_3$) δ 172.6, 152.7, 143.5, 134.6, 127.6, 126.9, 126.4, 125.9, 125.7, 125.4, 125.3, 125.2, 121.8, 121.1, 107.1, 73.8, 60.5, 58.3, 51.9, 40.4, 34.0, 33.3, 27.5, 14.2. Peak overlapping was observed. Peaks of ^{13}C on alkenyl group were difficult to observed. HRMS (ESI) *m/z*: $[M+H]^+$ Calcd for $C_{26}H_{31}NO_3S$ 438.2097; Found 438.2082.

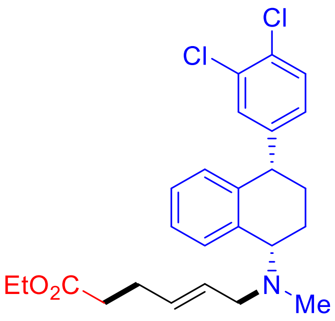

Ethyl (*E*)-6-(((1S,4S)-4-(3,4-dichlorophenyl)-1,2,3,4-tetrahydronaphthalen-1-yl)(methyl)amino)hex-4-enoate (4f)

Following General Procedure C on 0.2 mmol scale. *E:Z* > 95:5, the ratio was determined by crude ^{1}H NMR. Amine hydrochloride and Et_3N (1.5 equiv) was used. Column chromatography on silica gel (eluent: DCM/MeOH 40:1) afforded the title product in 56% yield (50.0 mg) as a pale yellow oil. ^{1}H NMR (400 MHz, Chloroform-*d*) δ 7.98 (s, 1H), 7.36 – 7.11 (m, 4H), 6.87 (dd, *J* = 38.6, 8.1 Hz, 2H), 5.85 – 5.47 (m, 2H), 4.26 – 3.93 (m, 4H), 3.21 (d, *J* = 74.4 Hz, 2H), 2.44 – 2.19 (m, 7H), 2.17 – 2.03 (m, 2H), 1.81 – 1.61 (m, 2H), 1.24 (t, *J* = 7.2 Hz, 3H). ^{13}C NMR (101 MHz, $CDCl_3$) δ 172.9, 146.8, 138.3, 132.3, 130.7, 130.5, 130.1, 128.9, 128.1, 127.4, 61.0, 60.4, 55.5, 43.4, 36.8, 33.9, 29.6, 27.6, 16.1, 14.3. Peak overlapping was observed. Peaks of ^{13}C on alkenyl group were difficult to observed. HRMS (ESI) *m/z*: $[M+H]^+$ Calcd for $C_{25}H_{29}Cl_2NO_2$ 446.1648; Found 446.1653.

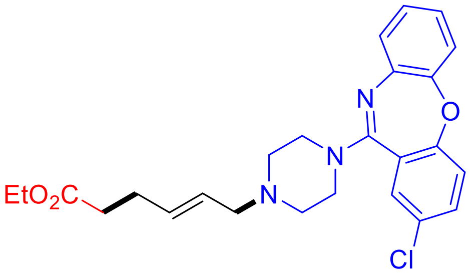

Ethyl (*E*)-6-(4-(2-chlorodibenzo[*b,f*][1,4]oxazepin-11-yl)piperazin-1-yl)hex-4-enoate (4g)

Following General Procedure C on 0.2 mmol scale. *E:Z* = 87:13, the ratio was determined by crude ^{1}H NMR. Column chromatography on silica gel (eluent: DCM/MeOH 40:1) afforded the title product in 63% yield (57.2 mg) as a pale yellow oil. ^{1}H NMR (400 MHz, Chloroform-*d*) δ 7.43 – 7.33 (m, 1H), 7.33 – 7.26 (m, 1H),

7.22 – 7.11 (m, 2H), 7.11 – 7.02 (m, 2H), 7.02 – 6.93 (m, 1H), 5.75 – 5.49 (m, 2H), 4.13 (q, J = 7.2 Hz, 2H), 3.68 – 3.43 (m, 4H), 3.10 (dd, J = 42.2, 5.5 Hz, 2H), 2.75 – 2.44 (m, 4H), 2.44 – 2.30 (m, 4H), 1.25 (t, J = 7.1 Hz, 3H). ^{13}C NMR (126 MHz, $CDCl_3$) δ 173.0, 159.3, 158.8, 151.8, 140.1, 133.3, 132.5, 130.2, 129.1, 127.1, 126.6, 125.8, 124.9, 124.5, 122.7, 120.1, 60.6, 60.4, 52.6, 47.1, 33.9, 27.7, 14.3. HRMS (ESI) *m/z*: $[M+H]^+$ Calcd for $C_{25}H_{28}ClN_3O_3$ 454.1892; Found 454.1899.

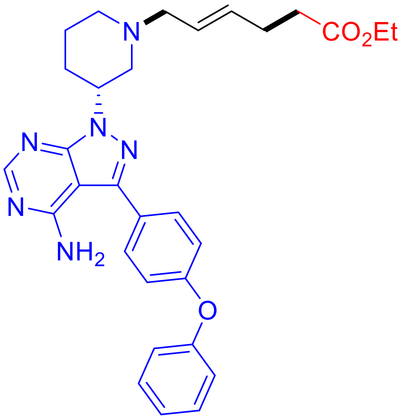

Ethyl (*R*,*E*)-6-(3-(4-amino-3-(4-phenoxyphenyl)-1*H*-pyrazolo[3,4-*d*]pyrimidin-1-yl)piperidin-1-yl)hex-4-enoate (4h)

Following General Procedure C on 0.2 mmol scale. *E:Z* = 89:11, the ratio was determined by crude ^{1}H NMR. Column chromatography on silica gel (eluent: DCM/MeOH 40:1) afforded the title product in 48% yield (50.6 mg) as a pale yellow oil. ^{1}H NMR (400 MHz, Chloroform-*d*) δ 8.33 (s, 1H), 7.74 – 7.55 (m, 2H), 7.48 – 7.31 (m, 2H), 7.26 – 7.11 (m, 3H), 7.11 – 7.00 (m, 2H), 5.92 (s, 2H), 5.76 – 5.62 (m, 2H), 5.28 – 5.12 (m, 1H), 4.09 (q, J = 7.1 Hz, 2H), 3.53 – 3.03 (m, 4H), 3.02 – 2.77 (m, 1H), 2.64 – 2.23 (m, 5H), 2.22 – 2.00 (m, 3H), 1.97 – 1.86 (m, 1H), 1.22 (t, J = 7.1 Hz, 3H). ^{13}C NMR (101 MHz, $CDCl_3$) δ 172.8, 158.5, 157.9, 156.3, 155.7, 154.1, 143.9, 135.3, 130.0, 129.9, 127.7, 124.5, 124.0, 119.5, 119.1, 98.5, 60.4, 60.2, 56.0, 52.8, 52.4, 33.6, 29.4, 27.6, 23.3, 14.2. HRMS (ESI) *m/z*: $[M+H]^+$ Calcd for $C_{30}H_{34}N_6O_3$ 527.2765; Found 527.2766.

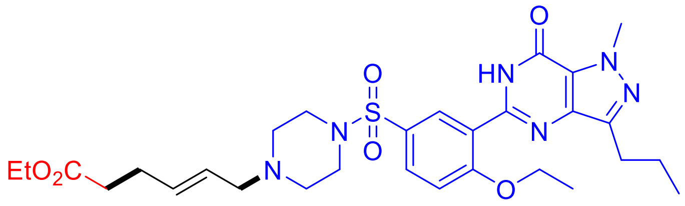

Ethyl (*E*)-6-(4-((4-ethoxy-3-(1-methyl-7-oxo-3-propyl-6,7-dihydro-1*H*-pyrazolo[4,3-*d*]pyrimidin-5-yl)phenyl)sulfonyl)piperazin-1-yl)hex-4-enoate (4i)

Following General Procedure C on 0.2 mmol scale. *E:Z* = 90:10, the ratio was determined by crude ^{1}H NMR. Column chromatography on silica gel (eluent: DCM/MeOH 40:1) afforded the title product in 67% yield (80.5 mg) as a pale yellow oil. ^{1}H NMR (400 MHz, Chloroform-*d*) δ 10.92 (s, 1H), 8.75 (s, 1H), 7.80 (d, *J* = 8.6 Hz, 1H), 7.15 (d, *J* = 8.7 Hz, 1H), 5.75 – 5.33 (m, 2H), 4.37 (q, *J* = 7.0 Hz, 2H), 4.26 (s, 3H), 4.10 (q, *J* = 7.0 Hz, 2H), 3.21 – 3.03(m, 4H), 2.98 – 2.89 (m, 4H), 2.70 – 2.52 (m, 4H), 2.44 – 2.25 (m, 4H), 1.91 – 1.81 (m, 2H), 1.63 (t, *J* = 6.9 Hz, 3H), 1.23 (t, *J* = 7.0 Hz, 3H), 1.02 (t, *J* = 7.4 Hz, 3H). ^{13}C NMR (126 MHz, $CDCl_3$) δ 172.8, 159.3, 153.7, 146.9, 146.5, 138.3, 133.3, 131.6, 131.1, 128.5, 126.5, 124.4, 121.1, 113.0, 66.0, 60.3, 60.0, 51.8, 45.9, 38.2, 33.7, 27.7, 27.5, 22.2, 14.5, 14.2, 14.0. HRMS (ESI) *m/z*: $[M+H]^+$ Calcd for $C_{29}H_{40}N_6O_6S$ 601.2803; Found 601.2810.

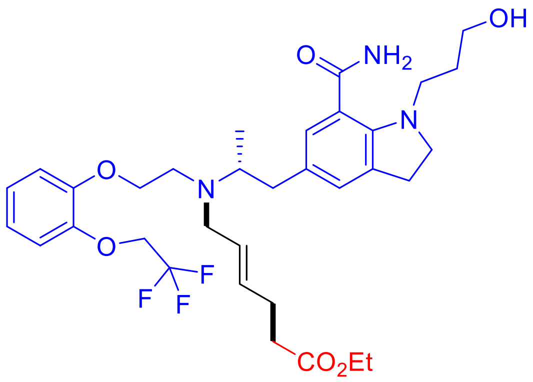

Ethyl (*R*,*E*)-6-((1-(7-carbamoyl-1-(3-hydroxypropyl)indolin-5-yl)propan-2-yl)(2-(2-(2,2,2-trifluoroethoxy)phenoxy)ethyl)amino)hex-4-enoate (4j)

Following General Procedure C on 0.2 mmol scale. *E:Z* > 95:5, the ratio was determined by crude ^{1}H NMR. Column chromatography on silica gel (eluent: DCM/MeOH 40:1) afforded the title product in 62% yield (78.8 mg) as a grown oil. ^{1}H NMR (500 MHz, Chloroform-*d*) δ 7.16 (s, 1H), 7.10 – 6.86 (m, 6H), 6.55 (s, 1H), 5.86

– 5.45 (m, 2H), 4.36 (q, J = 8.3 Hz, 2H), 4.28 – 4.00 (m, 4H), 3.79 – 3.69 (m, 2H), 3.63 – 2.82 (m, 13H), 2.48 – 2.27 (m, 5H), 1.88 – 1.66 (m, 2H), 1.22 (t, J = 7.2 Hz, 3H), 1.12 – 0.93 (m, 3H). ^{13}C NMR (101 MHz, $CDCl_3$) δ 173.0, 171.5, 149.9, 149.2, 147.1, 133.7, 128.1, 128.0, 124.9, 123.6 (q, J = 278.6 Hz), 121.4, 118.1, 117.3, 114.2, 67.8 (q, J = 35.9 Hz), 60.4, 59.5, 58.7, 53.7, 53.5, 50.7, 48.6, 38.2, 33.7, 31.0, 28.2, 27.5, 14.2, 14.1. Peak overlapping was observed. ^{19}F NMR (376 MHz, $CDCl_3$) δ -74.05. HRMS (ESI) *m/z*: $[M+H]^+$ Calcd for $C_{33}H_{44}F_3N_3O_6$ 636.3255; Found 636.3264.

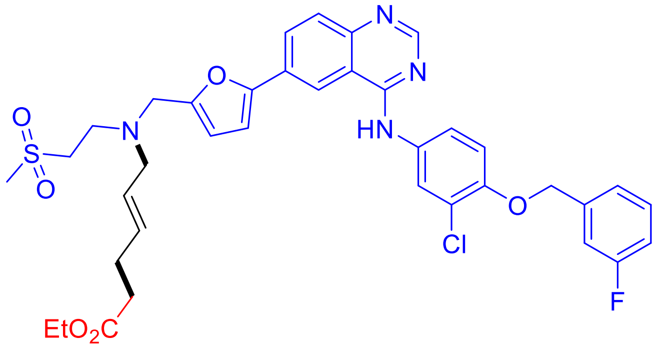

Ethyl (*E*)-6-(((5-(4-((3-chloro-4-((3-fluorobenzyl)oxy)phenyl)amino)quinazolin-6-yl)furan-2-yl)methyl)(2-(methylsulfonyl)ethyl)amino)hex-4-enoate (4k)

Following General Procedure C on 0.2 mmol scale. *E:Z* > 95:5, the ratio was determined by crude ^{1}H NMR. Column chromatography on silica gel (eluent: DCM/MeOH 40:1) afforded the title product in 37% yield (53.4 mg) as a yellow oil. ^{1}H NMR (400 MHz, Chloroform-*d*) δ 8.67 (s, 2H), 8.42 (s, 1H), 7.97 – 7.80 (m, 3H), 7.68 (d, J = 8.8 Hz, 1H), 7.40 – 7.31 (m, 1H), 7.27 – 7.20 (m, 2H), 7.05 – 6.92 (m, 2H), 6.78 – 6.62 (m, 1H), 6.41 – 6.23 (m, 1H), 5.77 – 5.44 (m, 2H), 5.14 (s, 2H), 4.12 (q, J = 7.1 Hz, 2H), 3.76 (s, 2H), 3.38 (t, J = 7.3 Hz, 2H), 3.24 (t, J = 7.2 Hz, 2H), 3.11 (d, J = 6.4 Hz, 2H), 2.97 (s, 3H), 2.50 – 2.32 (m, 4H), 1.25 – 1.21 (m, 3H). ^{13}C NMR (101 MHz, $CDCl_3$) δ 173.0, 163.0 (d, J = 246.1 Hz), 158.0, 154.7, 153.0, 151.6, 150.9, 149.0, 139.2 (d, J = 7.3 Hz), 133.3, 132.6, 130.2 (d, J = 8.1 Hz), 129.0, 128.7, 128.6, 127.4, 125.1, 123.1, 122.5 (d, J = 2.9 Hz), 122.4, 115.6, 115.5, 114.9 (d, J = 21.1 Hz), 114.2, 114.0 (d, J = 22.6 Hz), 111.8, 107.2, 70.4, 60.5, 56.6, 52.3, 48.0, 46.2, 41.9, 33.9, 27.6, 14.2. ^{19}F NMR (376 MHz, $CDCl_3$) δ -112.66. HRMS (ESI) *m/z*: $[M+H]^+$ Calcd for $C_{37}H_{38}ClFN_4O_6S$ 721.2257; Found 721.2250.

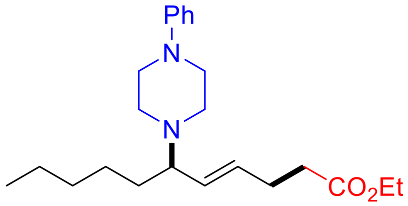

Ethyl (*E*)-6-(4-phenylpiperazin-1-yl)undec-4-enoate (4l)

Following General Procedure C on 0.2 mmol scale. *E:Z* > 95:5, the ratio was determined by crude ^{1}H NMR. 1,4-:1,2-addition = 2:1, the ratio was determined by GC–MS Column chromatography on silica gel (eluent: DCM/MeOH 40:1) afforded the title product in 61% yield (45.5 mg) as a pale yellow oil. ^{1}H NMR (contain 1,4- and 1, 2-adducts, 400 MHz, Chloroform-*d*) δ 7.34 – 7.17 (comp, 2H, major; comp, 2H, minor; overlap), 7.10 – 6.62 (comp, 3H, major; comp, 3H, minor; overlap), 5.69 – 5.23 (comp, 2H, major; comp, 2H, minor; overlap), 4.12 (comp, 2H, major; comp, 2H, minor; overlap), 3.45 – 3.13 (comp, 4H, major; comp, 4H, minor; overlap), 3.04 – 2.50 (comp, 5H, major; comp, 5H, minor; overlap), 2.44 – 2.32 (comp, 3H, major; comp, 3H, minor; overlap), 2.16 – 1.99 (comp, 1H, major; comp, 1H, minor; overlap), 1.91 – 1.68 (comp, 1H, major; comp, 1H, minor; overlap), 1.60 – 1.41 (comp, 1H, major; comp, 1H, minor; overlap), 1.42 – 1.33 (comp, 1H, major; comp, 1H, minor; overlap), 1.31 – 1.23 (comp, 8H, major; comp, 8H, minor; overlap), 0.91 – 0.84 (comp, 3H, major; comp, 3H, minor; overlap). ^{13}C NMR (contain 1,4- and 1, 2-adducts, 101 MHz, $CDCl_3$) δ 173.6, 172.9, 151.2, 151.0, 129.1, 129.0, 120.1, 119.8, 116.4, 116.1, 68.1, 67.2, 60.5, 60.3, 49.5, 49.4, 49.2, 48.8, 34.0, 32.4, 31.7, 31.4, 31.3, 29.7, 29.0, 27.7, 26.9, 26.1, 22.6, 22.5, 14.3, 14.1. Peak overlapping was observed. Peaks of ^{13}C on alkenyl group were difficult to observed. HRMS (ESI) *m/z*: $[M+H]^+$ Calcd for $C_{23}H_{36}N_2O_2$ 373.2850; Found 373.2857.

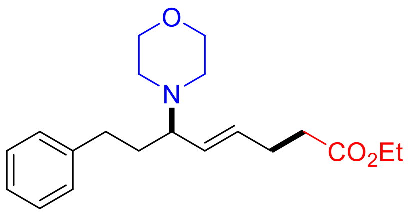

Ethyl (*E*)-6-morpholino-8-phenyloct-4-enoate (4m)

Following General Procedure C on 0.2 mmol scale. *E:Z* > 95:5, the ratio was determined by crude ^{1}H NMR. 1,4-:1,2-addition = 2:1, the ratio was determined by GC–MS Column chromatography on silica gel (eluent: DCM/MeOH 40:1) afforded the title product in 60% yield (39.7 mg) as a colorless oil. ^{1}H NMR (400 MHz, Chloroform-*d*) δ 7.41 – 7.25 (comp, 2H, major; comp, 2H, minor; overlap), 7.25 – 6.97 (comp, 3H, major; comp, 3H, minor; overlap), 5.69 – 5.17 (comp, 2H, major; comp, 2H, minor; overlap), 4.13 (comp, 2H, major; comp, 2H, minor; overlap), 3.93 – 3.47 (comp, 4H, major; comp, 4H, minor; overlap), 2.82 – 2.37 (comp, 10H, major; comp, 10H, minor; overlap), 2.34 – 2.14 (comp, 1H, major; comp, 1H, minor; overlap), 2.07 – 1.95 (comp, 1H, major; comp, 1H, minor; overlap), 1.78 – 1.62 (comp, 1H, major; comp, 1H, minor; overlap), 1.25 (comp, 3H, major; comp, 3H, minor; overlap). ^{13}C NMR (126 MHz, $CDCl_3$) δ 173.6, 172.9, 141.9, 141.4, 128.5, 128.4, 128.3, 125.9, 125.8, 67.5, 67.3, 66.9, 60.5, 60.3, 50.1, 49.9, 35.6, 34.1, 33.1, 32.4, 31.1, 27.8, 26.4, 14.3. Peak overlapping was observed. Peaks of ^{13}C on alkenyl group were difficult to observed. HRMS (ESI) *m/z*: $[M+H]^+$ Calcd for $C_{20}H_{29}NO_3$ 332.2220; Found 332.2229.

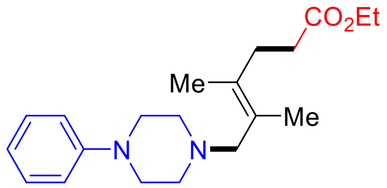

Ethyl (*E*)-4,5-dimethyl-6-(4-phenylpiperazin-1-yl)hex-4-enoate (4n)

Following General Procedure C on 0.2 mmol scale. *E:Z* = 80:20, the ratio was determined by crude ^{1}H NMR. Column chromatography on silica gel (eluent: DCM/MeOH 40:1) afforded the title product in 51% yield (33.7 mg) as a colorless oil. ^{1}H NMR of *E*-product (400 MHz, Chloroform-*d*) δ 7.29 – 7.23 (m, 2H), 6.97 – 6.81 (m,

3H), 4.13 (q, J = 7.1 Hz, 2H), 3.31 – 3.17 (m, 4H), 3.11 – 2.98 (m, 2H), 2.73 – 2.52 (m, 4H), 2.47 – 2.34 (m, 4H), 1.82 – 1.68 (m, 6H), 1.26 (t, J = 7.1 Hz, 3H). ^{13}C NMR of *E*-product (126 MHz, $CDCl_3$) δ 173.4, 151.2, 132.1, 129.1, 126.1, 119.7, 116.2, 60.8, 60.4, 52.8, 48.9, 32.8, 30.4, 18.1, 17.5, 14.3. HRMS (ESI) *m/z*: $[M+H]^+$ Calcd for $C_{20}H_{30}N_2O_2$ 331.2380; Found 331.2389.

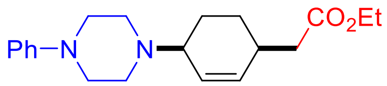

Ethyl 2-(4-(4-phenylpiperazin-1-yl)cyclohex-2-en-1-yl)acetate (4o)

Following General Procedure C on 0.2 mmol scale. 1,4-:1,2-addition = 2:1, the ratio was determined by GC–MS Column chromatography on silica gel (eluent: DCM/MeOH 40:1) afforded the title product in 50% yield (32.8 mg) as a colorless oil. ^{1}H NMR (500 MHz, Chloroform-*d*) δ 7.34 – 7.22 (m, 2H), 7.10 – 6.72 (m, 3H), 6.03 – 5.75 (m, 2H), 4.15 (q, J = 7.2 Hz, 2H), 3.62 – 3.18 (m, 5H), 3.11 – 2.74 (m, 4H), 2.67 – 2.57 (m, 1H), 2.41 – 2.26 (m, 2H), 1.97 – 1.67 (m, 3H), 1.64 – 1.55 (m, 1H), δ 1.30 – 1.23 (m, 3H). ^{13}C NMR (126 MHz, $CDCl_3$) δ 172.3, 150.8, 135.2, 135.1, 129.2, 120.3, 116.5, 60.5, 59.8, 48.7, 48.6, 39.5, 31.2, 25.6, 19.6, 14.3. HRMS (ESI) *m/z*: $[M+H]^+$ Calcd for $C_{20}H_{28}N_2O_2$ 329.2224; Found 329.2232.

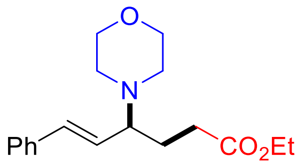

Ethyl (*E*)-4-morpholino-6-phenylhex-5-enoate (4p)

Following General Procedure C on 0.2 mmol scale. *E*:*Z* > 95:5, the ratio was determined by crude ^{1}H NMR. Column chromatography on silica gel (eluent: DCM/MeOH 40:1) afforded the title product in 60% yield (36.3 mg) as a colorless oil. ^{1}H NMR (400 MHz, Chloroform-*d*) δ 7.39 – 7.23 (m, 5H), 6.47 (d, J = 15.9 Hz, 1H), 6.10 (dd, J = 16.0, 9.0 Hz, 1H), 4.09 (q, J = 7.2 Hz, 2H), 3.80 – 3.67 (m, 4H), 3.06 – 2.89 (m, 1H), 2.78 – 2.62 (m, 2H), 2.61 – 2.52 (m, 2H), 2.42 – 2.32 (m, 2H), 2.17 – 2.07 (m, 1H), 1.90 – 1.79 (m, 1H), 1.22 (t, J = 7.1 Hz, 3H). ^{13}C NMR (101 MHz,

$CDCl_3$) δ 173.5, 136.5, 133.8, 128.6, 128.0, 127.8, 126.4, 67.7, 67.0, 60.4, 50.4, 31.1, 26.6, 14.2. HRMS (ESI) *m/z*: $[M+H]^+$ Calcd for $C_{18}H_{25}NO_3$ 304.1907; Found 304.1911.

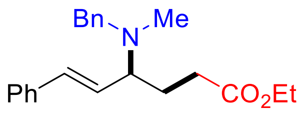

Ethyl (*E*)-4-(benzyl(methyl)amino)-6-phenylhex-5-enoate (4q)

Following General Procedure C on 0.2 mmol scale. *E:Z* > 95:5, the ratio was determined by crude ^1H NMR. Column chromatography on silica gel (eluent: DCM/MeOH 40:1) afforded the title product in 63% yield (42.5 mg) as a colorless oil. ^1H NMR (400 MHz, Chloroform-*d*) δ 7.43 – 7.23 (m, 10H), 6.47 (d, *J* = 15.9 Hz, 1H), 6.22 (dd, *J* = 15.9, 8.9 Hz, 1H), 4.15 – 4.04 (m, 2H), 3.75 (d, *J* = 13.4 Hz, 1H), 3.55 (d, *J* = 13.3 Hz, 1H), 3.26 – 3.14 (m, 1H), 2.50 – 2.37 (m, 2H), 2.26 (s, 3H), 2.19 – 2.09 (m, 1H), 2.00 – 1.88 (m, 1H), 1.22 (t, *J* = 7.1 Hz, 3H). ^{13}C NMR (101 MHz, $CDCl_3$) δ 173.7, 136.7, 133.8, 129.0, 128.6, 128.3, 127.7, 127.1, 126.4, 64.9, 60.3, 58.5, 37.1, 31.4, 27.6, 14.2. Peak overlapping was observed. HRMS (ESI) *m/z*: $[M+H]^+$ Calcd for $C_{22}H_{27}NO_2$ 338.2115; Found 338.2115.

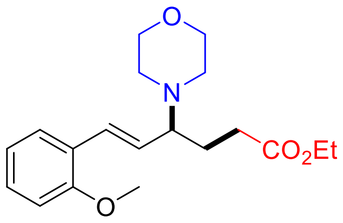

Ethyl (*E*)-6-(2-methoxyphenyl)-4-morpholinohex-5-enoate (4r)

Following General Procedure C on 0.2 mmol scale. *E:Z* > 95:5, the ratio was determined by crude ^1H NMR. Column chromatography on silica gel (eluent: DCM/MeOH 40:1) afforded the title product in 63% yield (42.0 mg) as a colorless oil. ^1H NMR (400 MHz, Chloroform-*d*) ^1H NMR (400 MHz, Chloroform-*d*) δ 7.43 (d, *J* = 7.7 Hz, 1H), 7.26 – 7.18 (m, 1H), 6.96 – 6.84 (m, 2H), 6.78 (d, *J* = 16.0 Hz, 1H), 6.09 (dd, *J* = 16.0, 9.1 Hz, 1H), 4.10 (q, *J* = 7.1 Hz, 2H), 3.84 (s, 3H), 3.77 – 3.69 (m, 4H), 3.03 – 2.92 (m, 1H), 2.73 – 2.63 (m, 2H), 2.61 – 2.55 (m, 2H), 2.42 – 2.34 (m, 2H), 2.15 – 2.08 (m, 1H), 1.90 – 1.81 (m, 1H), 1.23 (t, *J* = 7.1 Hz, 3H). ^{13}C NMR (101 MHz,

$CDCl_3$) δ 173.6, 156.7, 128.8, 128.6, 126.8, 125.5, 120.6, 110.9, 68.2, 67.1, 60.3, 55.5, 50.4, 31.2, 26.8, 14.2. Peak overlapping was observed. HRMS (ESI) *m/z*: $[M+H]^+$ Calcd for $C_{19}H_{27}NO_4$ 334.2013; Found 334.2017.

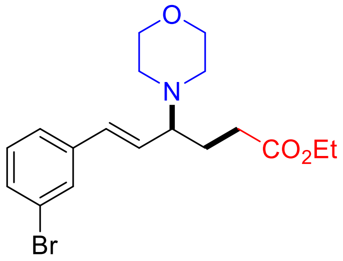

Ethyl (*E*)-6-(3-bromophenyl)-4-morpholinohex-5-enoate (4s)

Following General Procedure C on 0.2 mmol scale. *E:Z* > 95:5, the ratio was determined by crude ^1H NMR. Column chromatography on silica gel (eluent: DCM/MeOH 40:1) afforded the title product in 60% yield (45.9 mg) as a pale yellow oil. ^1H NMR (400 MHz, Chloroform-*d*) δ 7.55 – 7.48 (m, 1H), 7.39 – 7.35 (m, 1H), 7.29 – 7.25 (m, 1H), 7.21 – 7.15 (m, 1H), 6.40 (d, *J* = 15.9 Hz, 1H), 6.11 (dd, *J* = 15.9, 9.0 Hz, 1H), 4.10 (q, *J* = 7.1 Hz, 2H), 3.76 – 3.66 (m, 4H), 2.99 – 2.89 (m, 1H), 2.66 – 2.60 (m, 2H), 2.57 – 2.49 (m, 2H), 2.40 – 2.33 (m, 2H), 2.15 – 2.04 (m, 1H), 1.89 – 1.77 (m, 1H), 1.23 (t, *J* = 7.2 Hz, 3H). ^{13}C NMR (101 MHz, $CDCl_3$) δ 173.4, 138.7, 132.1, 130.5, 130.1, 130.0, 129.2, 125.0, 122.8, 67.4, 67.1, 60.4, 50.3, 31.1, 26.6, 14.2. HRMS (ESI) *m/z*: $[M+H]^+$ Calcd for $C_{18}H_{24}BrNO_3$ 382.1012; Found 382.1021.

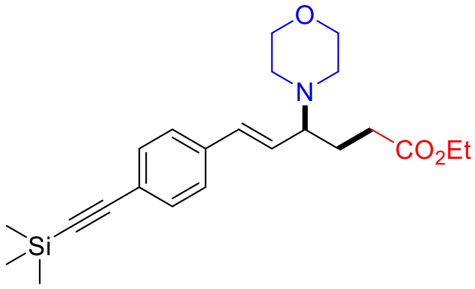

Ethyl (*E*)-4-morpholino-6-(4-((trimethylsilyl)ethynyl)phenyl)hex-5-enoate (4t)

Following General Procedure C on 0.2 mmol scale. *E:Z* > 95:5, the ratio was determined by crude ^1H NMR. Column chromatography on silica gel (eluent: DCM/MeOH 40:1) afforded the title product in 63% yield (50.4 mg) as a pale yellow

oil. ^{1}H NMR (400 MHz, Chloroform-*d*) δ 7.41 (d, *J* = 8.3 Hz, 2H), 7.28 (d, *J* = 8.3 Hz, 2H), 6.43 (d, *J* = 15.9 Hz, 1H), 6.11 (dd, *J* = 15.9, 9.0 Hz, 1H), 4.09 (qd, *J* = 7.1, 1.9 Hz, 2H), 3.78 – 3.61 (m, 4H), 2.98 – 2.86 (m, 1H), 2.69 – 2.59 (m, 2H), 2.59 – 2.48 (m, 2H), 2.41 – 2.33 (m, 2H), 2.14 – 2.05 (m, 1H), 1.91 – 1.76 (m, 1H), 1.22 (t, *J* = 7.2 Hz, 3H), 0.25 (s, 9H). ^{13}C NMR (101 MHz, $CDCl_3$) δ 173.5, 136.6, 132.9, 132.2, 129.5, 126.1, 122.3, 105.0, 95.0, 67.6, 67.2, 60.4, 50.4, 31.1, 26.7, 14.2, 0.0. HRMS (ESI) *m/z*: $[M+H]^+$ Calcd for $C_{23}H_{33}NO_3Si$ 400.2302; Found 400.2307.

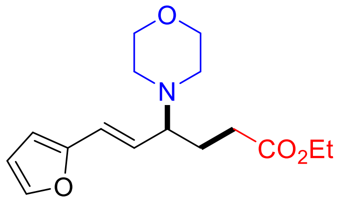

Ethyl (*E*)-6-(furan-2-yl)-4-morpholinohex-5-enoate (4u)

Following General Procedure C on 0.2 mmol scale. *E:Z* > 95:5, the ratio was determined by crude ^{1}H NMR. 1,4-:1,2-addition = 4:1, the ratio was determined by GC–MS Column chromatography on silica gel (eluent: DCM/MeOH 40:1) afforded the title product in 64% yield (37.6 mg) as a yellow oil. ^{1}H NMR (400 MHz, Chloroform-*d*) δ 7.34 (d, *J* = 1.7 Hz, 1H), 6.44 – 6.17 (m, 3H), 6.02 (dd, *J* = 15.9, 9.1 Hz, 1H), 4.10 (q, *J* = 7.1 Hz, 2H), 3.75 – 3.63 (m, 4H), 2.99 – 2.81 (m, 1H), 2.70 – 2.58 (m, 2H), 2.57 – 2.46 (m, 2H), 2.41 – 2.33 (m, 2H), 2.12 – 2.03 (m, 1H), 1.88 – 1.75 (m, 1H), 1.24 (t, *J* = 7.2 Hz, 3H). ^{13}C NMR (126 MHz, $CDCl_3$) δ 173.5, 152.1, 141.9, 126.9, 121.8, 111.3, 107.8, 67.3, 67.2, 60.4, 50.2, 31.1, 26.7, 14.2. HRMS (ESI) *m/z*: $[M+H]^+$ Calcd for $C_{16}H_{23}NO_4$ 294.1700; Found 294.1704.

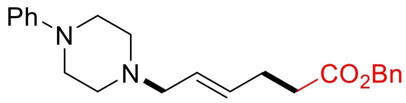

Benzyl (*E*)-6-(4-phenylpiperazin-1-yl)hex-4-enoate (4v)

Following General Procedure C on 0.2 mmol scale. *E:Z* = 85:15, the ratio was determined by crude ^{1}H NMR. Column chromatography on silica gel (eluent: DCM/MeOH 40:1) afforded the title product in 64% yield (46.7 mg) as a pale yellow oil. ^{1}H NMR (400 MHz, Chloroform-*d*) δ 7.46 – 7.05 (m, 7H), 7.00 – 6.67 (m, 3H),

5.81 – 5.40 (m, 2H), 5.12 (s, 2H), 3.44 – 3.12 (m, 4H), δ 3.12 (dd, J = 39.4, 5.3 Hz, 2H), 2.84 – 2.49 (m, 4H), 2.49 – 2.27 (m, 4H). ^{13}C NMR (101 MHz, $CDCl_3$) δ 172.7, 151.0, 135.9, 133.8, 129.2, 128.6, 128.3, 128.2, 126.2, 120.0, 116.3, 66.3, 60.4, 52.7, 48.7, 33.8, 27.6. HRMS (ESI) *m/z*: $[M+H]^+$ Calcd for $C_{23}H_{28}N_2O_2$ 365.2224; Found 365.2216.

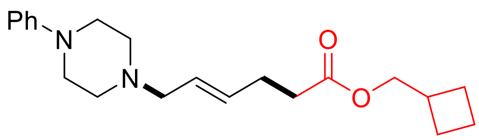

Cyclobutylmethyl (*E*)-6-(4-phenylpiperazin-1-yl)hex-4-enoate (4w)

Following General Procedure C on 0.2 mmol scale. *E:Z* = 84:16, the ratio was determined by crude ^{1}H NMR. Column chromatography on silica gel (eluent: DCM/MeOH 40:1) afforded the title product in 69% yield (47.3 mg) as a yellow oil. ^{1}H NMR (400 MHz, Chloroform-*d*) δ 7.36 – 7.14 (m, 2H), 7.02 – 6.51 (m, 3H), 5.82 – 5.44 (m, 2H), 4.05 (d, J = 6.8 Hz, 2H), 3.35 – 3.13 (m, 4H), 3.12 (dd, J = 42.2, 5.5 Hz, 1H), 2.88 – 2.46 (m, 5H), 2.46 – 2.26 (m, 4H), 2.16 – 1.98 (m, 2H), 1.96 – 1.83 (m, 2H), 1.81 – 1.70 (m, 2H). ^{13}C NMR (101 MHz, $CDCl_3$) δ 173.1, 151.1, 133.7, 129.1, 126.3, 119.9, 116.2, 68.3, 60.5, 52.8, 48.8, 34.1, 33.9, 27.7, 24.8, 18.4. HRMS (ESI) *m/z*: $[M+H]^+$ Calcd for $C_{21}H_{30}N_2O_2$ 343.2380; Found 343.2389.

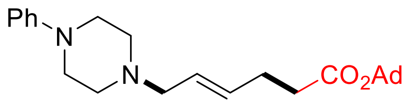

(3*s*,5*s*,7*s*)-Adamantan-1-yl (*E*)-6-(4-phenylpiperazin-1-yl)hex-4-enoate (4x)

Following General Procedure C on 0.2 mmol scale. *E:Z* = 92:8, the ratio was determined by crude ^{1}H NMR. Column chromatography on silica gel (eluent: DCM/MeOH 40:1) afforded the title product in 77% yield (62.9 mg) as a pale yellow oil. ^{1}H NMR (400 MHz, Chloroform-*d*) δ 7.31 – 7.23 (m, 2H), 7.00 – 6.79 (m, 3H), 5.79 – 5.55 (m, 2H), 3.40 – 3.22 (m, 4H), 3.16 (d, J = 6.4 Hz, 2H), 2.92 – 2.63 (m, 4H), 2.46 – 2.25 (m, 4H), 2.21 – 2.12 (m, 3H), 2.12 – 2.03 (m, 6H), 1.69 – 1.58 (m, 6H). ^{13}C NMR (126 MHz, $CDCl_3$) δ 172.0, 150.9, 135.0, 129.2, 125.0, 120.2, 116.3, 80.5, 60.3,

52.5, 48.5, 41.4, 36.2, 35.1, 30.8, 27.8. HRMS (ESI) *m/z*: $[M+H]^+$ Calcd for $C_{26}H_{36}N_2O_2$ 409.2850; Found 409.2851.

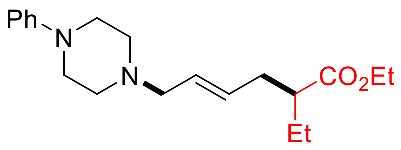

Ethyl (*E*)-2-ethyl-6-(4-phenylpiperazin-1-yl)hex-4-enoate (4y)

Following General Procedure C on 0.2 mmol scale. *E:Z* = 85:15, the ratio was determined by crude ^1H NMR. Column chromatography on silica gel (eluent: DCM/MeOH 40:1) afforded the title product in 72% yield (47.6 mg) as a yellow oil. ^1H NMR (400 MHz, Chloroform-*d*) δ 7.31 – 7.22 (m, 2H), 6.95 – 6.89 (m, 2H), 6.89 – 6.82 (m, 1H), 5.66 – 5.54 (m, 2H), 4.13 (qd, *J* = 7.1, 1.7 Hz, 2H), 3.28 – 3.20 (m, 4H), 3.09 (dd, *J* = 36.3, 4.6 Hz, 2H), 2.64 (t, *J* = 5.1 Hz, 4H), 2.42 – 2.31 (m, 2H), 2.31 – 2.20 (m, 1H), 1.69 – 1.50 (m, 2H), 1.25 (t, *J* = 7.1 Hz, 3H), 0.91 (t, *J* = 7.4 Hz, 3H). ^{13}C NMR (101 MHz, $CDCl_3$) δ 175.5, 151.1, 132.2, 129.1, 127.6, 119.8, 116.2, 60.5, 60.2, 52.8, 48.9, 47.1, 34.7, 25.0, 14.4, 11.7. HRMS (ESI) *m/z*: $[M+H]^+$ Calcd for $C_{20}H_{30}N_2O_2$ 331.2380; Found 331.2384.

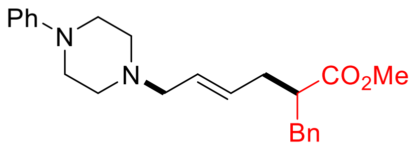

Methyl (*E*)-2-benzyl-6-(4-phenylpiperazin-1-yl)hex-4-enoate (4z)

Following General Procedure C on 0.2 mmol scale. *E:Z* = 84:16, the ratio was determined by crude ^1H NMR. Diazo compounds (0.4 mmol) was used. Column chromatography on silica gel (eluent: DCM/MeOH 40:1) afforded the title product in 51% yield (38.6 mg) as a pale yellow oil. ^1H NMR (400 MHz, Chloroform-*d*) δ 7.30 – 7.14 (m, 7H), 6.96 – 6.89 (m, 2H), 6.88 – 6.79 (m, 1H), 5.69 – 5.50 (m, 2H), 3.60 (s, 3H), 3.22 (t, *J* = 4.7 Hz, 4H), 3.08 – 2.92 (m, 3H), 2.81 – 2.71 (m, 2H), 2.61 (t, *J* = 4.7 Hz, 4H), 2.42 – 2.26 (m, 2H). ^{13}C NMR (101 MHz, $CDCl_3$) δ 175.2, 151.2, 139.1, 131.3, 129.1, 128.9, 128.6, 128.4, 126.4, 119.8, 116.1, 60.6, 52.9, 51.5, 49.0, 47.5, 37.9, 34.6. HRMS (ESI) *m/z*: $[M+H]^+$ Calcd for $C_{24}H_{30}N_2O_2$ 379.2380; Found 379.2385.

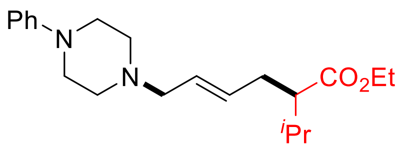

Ethyl (*E*)-2-isopropyl-6-(4-phenylpiperazin-1-yl)hex-4-enoate (4aa)

Following General Procedure C on 0.2 mmol scale. *E:Z* = 86:14, the ratio was determined by crude ^{1}H NMR. Column chromatography on silica gel (eluent: DCM/MeOH 40:1) afforded the title product in 51% yield (35.1 mg) as a yellow oil. ^{1}H NMR (400 MHz, Chloroform-*d*) δ 7.29 – 7.23 (m, 2H), 6.96 – 6.89 (m, 2H), 6.89 – 6.81 (m, 1H), 5.63 – 5.52 (m, 2H), 4.17 – 4.05 (m, 2H), 3.30 – 3.19 (m, 4H), 3.16 – 3.00 (m, 2H), 2.63 (t, *J* = 5.0 Hz, 4H), 2.39 – 2.15 (m, 3H), 1.95 – 1.81 (m, 1H), 1.25 (t, *J* = 7.1 Hz, 3H), 0.94 (dd, *J* = 11.6, 6.8 Hz, 6H). ^{13}C NMR (101 MHz, $CDCl_3$) δ 174.9, 151.2, 132.6, 129.1, 127.4, 119.8, 116.1, 60.6, 60.0, 52.8, 52.7, 48.9, 32.6, 30.3, 20.3, 20.2, 14.4. HRMS (ESI) *m/z*: $[M+H]^+$ Calcd for $C_{21}H_{32}N_2O_2$ 345.2537; Found 345.2545.

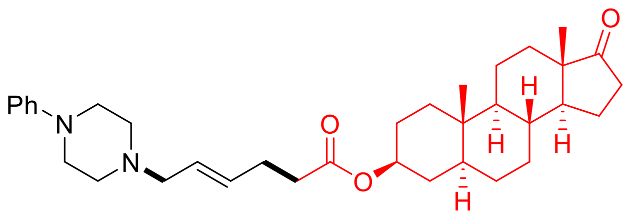

(3*S*,5*S*,8*R*,9*S*,10*S*,13*S*,14*S*)-10,13-Dimethyl-17-oxohexadecahydro-1*H*-cyclopenta[*a*]phenanthren-3-yl (*E*)-6-(4-phenylpiperazin-1-yl)hex-4-enoate (4ab)

Following General Procedure C on 0.2 mmol scale. *E:Z* = 88:12, the ratio was determined by crude ^{1}H NMR. Column chromatography on silica gel (eluent: DCM/MeOH 40:1) afforded the title product in 59% yield (64.5 mg) as a pale yellow oil. ^{1}H NMR (500 MHz, Chloroform-*d*) δ 7.34 – 7.17 (m, 2H), 6.99 – 6.81 (m, 3H), 5.85 – 5.47 (m, 2H), 4.70 (tt, *J* = 10.9, 4.9 Hz, 1H), 3.28 (t, *J* = 5.0 Hz, 4H), 3.18 (dd, *J* = 55.8, 5.7 Hz, 2H), 2.72 (t, *J* = 5.0 Hz, 4H), 2.47 – 2.35 (m, 5H), 2.11 – 2.03 (m, 1H), 1.96 – 1.90 (m, 1H), 1.83 – 1.72 (m, 4H), 1.66 – 1.59 (m, 2H), 1.56 – 1.46 (m, 3H), 1.33 – 1.22 (m, 7H), 1.06 – 0.96 (m, 2H), 0.87 – 0.82 (m, 6H), 0.73 – 0.68 (m, 1H). ^{13}C NMR (126 MHz, $CDCl_3$) δ 221.3, 172.4, 150.9, 134.4, 129.2, 125.5, 120.1,

116.3, 73.6, 60.3, 54.3, 52.5, 51.3, 48.6, 47.8, 44.6, 36.7, 35.8, 35.6, 35.0, 34.1, 34.0, 31.5, 30.8, 28.3, 27.7, 27.5, 21.8, 20.5, 13.8, 12.2. HRMS (ESI) *m/z*: $[M+H]^+$ Calcd for $C_{35}H_{50}N_2O_3$ 547.3894; Found 547.3901.

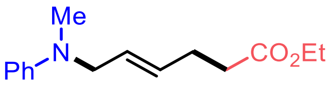

Ethyl (*E*)-6-(methyl(phenyl)amino)hex-4-enoate (4ac)

Following General Procedure C on 0.2 mmol scale replacing $Pd(OAc)_2$ with $Pd(PPh_3)_2Cl_2$. *E*:*Z* = 91:9, the ratio was determined by crude ^1H NMR. Column chromatography on silica gel (eluent: DCM/MeOH = 200:1) afforded the title product in 69% yield (36.0 mg) as a pale yellow oil. ^1H NMR (400 MHz, Chloroform-*d*) δ 7.24 – 7.17 (m, 2H), 6.76 – 6.67 (m, 3H), 5.63 – 5.47 (m, 2H), 4.10 (q, *J* = 7.1 Hz, 2H), 3.85 (d, *J* = 5.1 Hz, 2H), 2.88 (s, 3H), 2.37 – 2.30 (m, 4H), 1.23 (t, *J* = 7.1 Hz, 3H). ^{13}C NMR (101 MHz, $CDCl_3$) δ 173.0, 149.6, 130.6, 129.1, 126.6, 116.4, 112.6, 60.3, 54.4, 37.7, 34.1, 27.6, 14.2. HRMS (ESI) *m/z*: $[M+H]^+$ Calcd for $C_{16}H_{23}NO_2$ 262.1802; Found 262.1805.

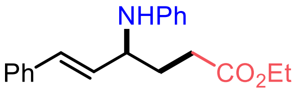

Ethyl (*E*)-6-phenyl-4-(phenylamino)hex-5-enoate (4ad)

Following General Procedure C on 0.2 mmol scale replacing $Pd(OAc)_2$ with $Pd(PPh_3)_2Cl_2$. *E*:*Z* > 95:5, the ratio was determined by crude ^1H NMR. Column chromatography on silica gel (eluent: DCM/MeOH = 200:1) afforded the title product in 71% yield (41.8 mg) as a pale yellow oil. ^1H NMR (400 MHz, Chloroform-*d*) δ 7.34 – 7.12 (m, 7H), 6.72 – 6.51 (m, 4H), 6.10 (dd, *J* = 16.0, 6.4 Hz, 1H), 4.14 – 4.00 (m, 3H), 3.86 (s, 1H), 2.52 – 2.41 (m, 2H), 2.02 (qd, *J* = 7.1, 1.9 Hz, 2H), 1.22 (t, *J* = 7.1 Hz, 3H). ^{13}C NMR (101 MHz, $CDCl_3$) δ 173.6, 147.4, 136.8, 131.1, 130.9, 129.2, 128.5, 127.5, 126.4, 117.5, 113.5, 60.6, 55.4, 31.0, 30.8, 14.3. HRMS (ESI) *m/z*: $[M+H]^+$ Calcd for $C_{20}H_{23}NO_2$ 310.1802; Found 310.1807.

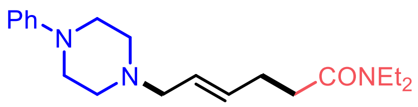

(*E*)-*N*,*N*-Diethyl-6-(4-phenylpiperazin-1-yl)hex-4-enamide (4ae)

Following General Procedure C on 0.2 mmol scale replacing $Pd(OAc)_2$ with $Pd(PPh_3)_2Cl_2$. *E*:*Z* = 93:7, the ratio was determined by crude ^{1}H NMR. Column chromatography on silica gel (eluent: DCM/MeOH = 30:1) afforded the title product in 65% yield (48.4 mg) as a pale yellow oil. ^{1}H NMR (400 MHz, Chloroform-*d*) δ 7.31 – 7.23 (m, 2H), 6.98 – 6.81 (m, 3H), 5.81 (dt, *J* = 15.7, 5.8 Hz, 1H), 5.65 (dt, *J* = 14.8, 6.9 Hz, 1H), 3.42 – 3.28 (m, 8H), 3.17 (d, *J* = 6.8 Hz, 2H), 2.86 – 2.70 (m, 4H), 2.49 – 2.37 (m, 4H), 1.17 (t, *J* = 7.1 Hz, 3H), 1.11 (t, *J* = 7.1 Hz, 3H). ^{13}C NMR (101 MHz, $CDCl_3$) δ 171.1, 150.8, 136.1, 129.2, 124.3, 120.2, 116.4, 60.3, 52.4, 48.5, 42.0, 40.2, 32.4, 28.0, 14.4, 13.2. HRMS (ESI) *m/z*: $[M+H]^+$ Calcd for $C_{20}H_{31}N_3O$ 330.2540; Found 330.2556.

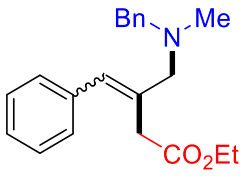

Ethyl 3-((benzyl(methyl)amino)methyl)-4-phenylbut-3-enoate (6a)

Following General Procedure D on 0.2 mmol scale. Column chromatography on silica gel (eluent: PE/EA = 30:1) afforded the title product in 73% yield (47.2 mg) as a colorless oil. *E*:*Z* = 50:50, the ratio was determined by ^{1}H NMR. ^{1}H NMR (500 MHz, Chloroform-*d*) δ 7.35 – 7.20 (comp, 10H, major; comp, 10H, minor; overlap), 6.72 – 6.57 (comp, 1H, major; comp, 1H, minor; overlap), 4.14 (q, *J* = 7.0 Hz, 2H, major; q, *J* = 7.0 Hz, 2H, minor; overlap), 3.57 – 3.12 (comp, 6H, major; comp, 6H, minor; overlap), 2.22 – 2.02 (comp, 3H, major; comp, 3H, minor; overlap), 1.27 (t, *J* = 7.1 Hz, 3H, major; t, *J* = 7.1 Hz, 3H, minor; overlap). ^{13}C NMR (126 MHz, $CDCl_3$) δ 172.0, 171.7, 139.4, 137.0, 134.7, 133.7, 131.6, 130.8, 129.1, 128.9, 128.8, 128.6, 128.3, 128.2, 128.1, 128.0, 126.9, 126.8, 126.7, 64.8, 62.1, 62.0, 60.6, 60.5, 56.5, 42.0, 41.0, 35.4, 14.3. Peak overlapping was observed. HRMS (ESI) *m/z*: $[M+H]^+$ Calcd for $C_{21}H_{25}NO_2$ 324.1958; Found 324.1968. The hydrogen on the alkenyl group provided the

characteristic peak of ^{1}H NMR spectra. Based on the reported ^{1}H NMR spectra of product **3b** in reference 14[14], we determined the corresponding *E/Z* ratios for each product generated from allenes.

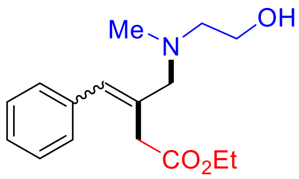

Ethyl 3-(((2-hydroxyethyl)(methyl)amino)methyl)-4-phenylbut-3-enoate (6b)

Following General Procedure D on 0.2 mmol scale. Column chromatography on silica gel (eluent: PE/EA = 30:1) afforded the title product in 93% yield (51.6 mg) as a colorless oil. *E:Z* = 50:50, the ratio was determined by ^{1}H NMR. ^{1}H NMR (400 MHz, Chloroform-*d*) δ 7.36 – 7.30 (comp, 3H, major; comp, 3H, minor; overlap), 7.26 – 7.17 (comp, 2H, major; comp, 2H, minor; overlap), 6.71 – 6.60 (comp, 1H, major; comp, 1H, minor; overlap), 4.21 – 4.15 (comp, 2H, major; comp, 2H, minor; overlap), 3.67 – 3.56 (comp, 2H, major; comp, 2H, minor; overlap), 3.35 – 3.19 (comp, 4H, major; comp, 4H, minor; overlap), 2.66 – 2.46 (comp, 3H, major; comp, 3H, minor; overlap), 2.26 – 2.10 (comp, 3H, major; comp, 3H, minor; overlap), 1.32 – 1.27 (comp, 3H, major; comp, 3H, minor; overlap). ^{13}C NMR (101 MHz, $CDCl_3$) δ 172.2, 172.0, 136.8, 136.6, 132.6, 131.7, 129.0, 128.6, 128.3, 128.1, 127.2, 126.9, 65.2, 61.0, 60.9, 59.5, 59.4, 58.7, 58.6, 56.6, 41.8, 41.5, 36.0, 14.2, 14.1. Peak overlapping was observed. HRMS (ESI) *m/z*: $[M+H]^+$ Calcd for $C_{16}H_{23}NO_3$ 278.1751; Found 278.1760.

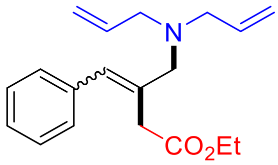

Ethyl 3-((diallylamino)methyl)-4-phenylbut-3-enoate (6c)

Following General Procedure D on 0.2 mmol scale. Column chromatography on silica gel (eluent: PE/EA = 30:1) afforded the title product in 34% yield (20.4 mg) as a colorless oil. *E:Z* = 44:56, the ratio was determined by ^{1}H NMR. ^{1}H NMR (400 MHz, Chloroform-*d*) δ 7.34 – 7.17 (comp, 5H, major; comp, 5H, minor; overlap), 6.69 – 6.54

(comp, 1H, major; comp, 1H, minor; overlap), 5.95 – 5.63 (comp, 2H, major; comp, 2H, minor; overlap), 5.25 – 5.01 (comp, 4H, major; comp, 4H, minor; overlap), 4.25 – 4.12 (comp, 2H, major; comp, 2H, minor; overlap), 3.36 – 2.82 (comp, 8H, major; comp, 8H, minor; overlap), 1.34 – 1.26 (comp, 3H, major; comp, 3H, minor; overlap). ^{13}C NMR (126 MHz, $CDCl_3$) δ 172.0, 171.7, 137.1, 137.0, 136.0, 135.0, 133.8, 131.7, 130.7, 129.1, 128.6, 128.2, 128.0, 126.9, 126.6, 117.2, 117.1, 60.6, 60.4, 56.5, 56.4, 52.3, 41.1, 35.5, 14.3, 14.2. Peak overlapping was observed. HRMS (ESI) *m/z*: $[M+H]^+$ Calcd for $C_{19}H_{25}NO_2$ 300.1958; Found 300.1966.

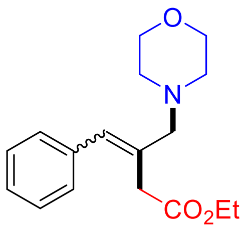

Ethyl 3-(morpholinomethyl)-4-phenylbut-3-enoate (6d)

Following General Procedure D on 0.2 mmol scale. Column chromatography on silica gel (eluent: PE/EA = 30:1) afforded the title product in 70% yield (40.5 mg) as a colorless oil. *E:Z* = 40:60, the ratio was determined by ^{1}H NMR. ^{1}H NMR (500 MHz, Methanol-d_4) δ 7.34 – 7.19 (comp, 5H, major; comp, 5H, minor; overlap), 6.71 – 6.62 (comp, 1H, major; comp, 1H, minor; overlap), 4.15 (q, *J* = 7.2 Hz, 2H, major; q, *J* = 7.2 Hz, 2H, minor; overlap), 3.68 – 3.57 (comp, 4H, major; comp, 4H, minor; overlap), 3.30 – 3.27 (comp, 2H, major; comp, 2H, minor; overlap), 3.22 – 3.11 (comp, 2H, major; comp, 2H, minor; overlap), 2.50 – 2.26 (comp, 4H, major; comp, 4H, minor; overlap), 1.30 – 1.25 (comp, 3H, major; comp, 3H, minor; overlap). ^{13}C NMR (126 MHz, MeOD) δ 172.5, 172.0, 136.8, 136.7, 133.0, 132.4, 131.8, 131.6, 128.6, 128.2, 127.9, 127.7, 126.8, 126.5, 66.7, 66.6, 65.9, 60.4, 60.3, 57.4, 53.3, 53.2, 40.8, 35.2, 13.2, 13.1. Peak overlapping was observed. HRMS (ESI) *m/z*: $[M+H]^+$ Calcd for $C_{17}H_{23}NO_3$ 290.1751; Found 290.1760.

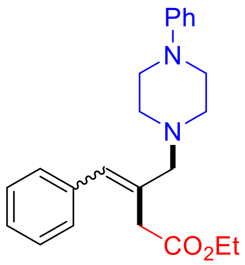

Ethyl 4-phenyl-3-((4-phenylpiperazin-1-yl)methyl)but-3-enoate (6e)

Following General Procedure D on 0.2 mmol scale. Column chromatography on silica gel (eluent: PE/EA = 30:1) afforded the title product in 62% yield (45.2 mg) as a colorless oil. *E*:*Z* = 48:52, the ratio was determined by ^{1}H NMR. ^{1}H NMR (500 MHz, Chloroform-*d*) δ 7.38 – 7.26 (comp, 7H, major; comp, 7H, minor; overlap), 6.99 – 6.84 (comp, 3H, major; comp, 3H, minor; overlap), 6.73 – 6.62 (comp, 1H, major; comp, 1H, minor; overlap), 4.19 (q, *J* = 7.1 Hz, 2H, major; q, *J* = 7.1 Hz, 2H, minor; overlap), 3.39 – 3.11 (comp, 8H, major; comp, 8H, minor; overlap), 2.69 – 2.49 (comp, 4H, major; comp, 4H, minor; overlap), 1.30 (t, *J* = 7.1 Hz, 3H, major; t, *J* = 7.1 Hz, 3H, minor; overlap). ^{13}C NMR (101 MHz, $CDCl_3$) δ 172.0, 171.7, 151.4, 151.4, 136.9, 136.8, 133.7, 132.5, 132.1, 131.2, 129.1, 129.1, 129.0, 128.6, 128.3, 128.1, 127.0, 126.8, 119.6, 119.5, 116.0, 115.9, 65.8, 60.6, 60.5, 57.4, 53.1, 53.0, 49.3, 49.2, 41.4, 35.8, 14.3, 14.2. Peak overlapping was observed. HRMS (ESI) *m/z*: $[M+H]^+$ Calcd for $C_{23}H_{28}N_2O_2$ 365.2224; Found 365.2218.

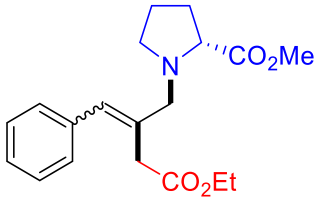

Methyl (2-benzylidene-4-ethoxy-4-oxobutyl)-D-prolinate (6f)

Following General Procedure D on 0.2 mmol scale. Column chromatography on silica gel (eluent: PE/EA = 30:1) afforded the title product in 68% yield (45.1 mg) as a colorless oil. *E*:*Z* = 55:45, the ratio was determined by ^{1}H NMR. ^{1}H NMR (400 MHz, Chloroform-*d*) δ 7.36 – 7.18 (comp, 5H, major; comp, 5H, minor; overlap), 6.67 – 6.45 (comp, 1H, major; comp, 1H, minor; overlap), 4.23 – 4.12 (comp, 2H, major; comp, 2H, minor; overlap), 3.71 – 3.55 (comp, 3H, major; comp, 3H, minor; overlap), 3.51 –

3.25 (comp, 4H, major; comp, 4H, minor; overlap), 3.14 – 2.97 (comp, 1H, major; comp, 1H, minor; overlap), 2.47 – 2.19 (comp, 1H, major; comp, 1H, minor; overlap), 2.12 – 1.66 (comp, 5H, major; comp, 5H, minor; overlap), 1.29 (t, *J* = 6.6 Hz, 3H, major; t, *J* = 6.6 Hz, 3H, minor; overlap). ^{13}C NMR (126 MHz, $CDCl_3$) δ 174.8, 174.6, 172.1, 171.8, 137.0, 136.9, 134.6, 133.8, 131.0, 130.5, 129.1, 128.6, 128.2, 128.0, 126.9, 126.7, 65.5, 65.3, 61.8, 60.6, 60.4, 53.5, 53.2, 53.1, 51.6, 51.5, 40.7, 35.2, 29.6, 29.3, 23.4, 23.3, 14.3, 14.2. HRMS (ESI) *m/z*: $[M+H]^+$ Calcd for $C_{19}H_{25}NO_4$ 332.1856; Found 332.1858.

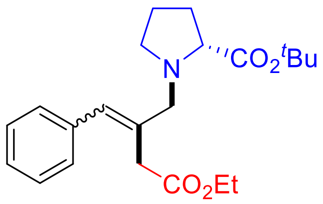

***tert*-Butyl (2-benzylidene-4-ethoxy-4-oxobutyl)-D-prolinate (6g)**

Following General Procedure D on 0.2 mmol scale. Column chromatography on silica gel (eluent: PE/EA = 30:1) afforded the title product in 73% yield (54.5 mg) as a colorless oil. *E:Z* = 50:50, the ratio was determined by ^{1}H NMR. ^{1}H NMR (400 MHz, Chloroform-*d*) δ 7.34 – 7.19 (comp, 5H, major; comp, 5H, minor; overlap), 6.68 – 6.49 (comp, 1H, major; comp, 1H, minor; overlap), 4.20 – 4.10 (comp, 2H, major; comp, 2H, minor; overlap), 3.55 – 3.22 (comp, 4H, major; comp, 4H, minor; overlap), 3.21 – 2.89 (comp, 2H, major; comp, 2H, minor; overlap), 2.52 – 2.21 (comp, 1H, major; comp, 1H, minor; overlap), 2.14 – 1.70 (comp, 4H, major; comp, 4H, minor; overlap), 1.47 – 1.37 (comp, 9H, major; comp, 9H, minor; overlap), 1.33 – 1.25 (comp, 3H, major; comp, 3H, minor; overlap). ^{13}C NMR (101 MHz, $CDCl_3$) δ 173.6, 173.5, 172.2, 171.9, 137.2, 137.1, 134.9, 134.0, 130.8, 130.2, 129.2, 128.6, 128.2, 128.0, 126.8, 126.6, 80.3, 66.3, 66.0, 61.4, 60.5, 60.4, 53.2, 53.0, 40.7, 35.3, 29.5, 29.4, 28.1, 28.0, 23.4, 23.3, 14.3, 14.2. Peak overlapping was observed. HRMS (ESI) *m/z*: $[M+H]^+$ Calcd for $C_{22}H_{31}NO_4$ 374.2326; Found 374.2319.

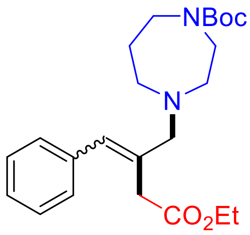

***tert*-Butyl 4-(2-benzylidene-4-ethoxy-4-oxobutyl)-1,4-diazepane-1-carboxylate (6h)**

Following General Procedure D on 0.2 mmol scale. Column chromatography on silica gel (eluent: PE/EA = 30:1) afforded the title product in 59% yield (47.5 mg) as a colorless oil. *E:Z* = 55:45, the ratio was determined by ^{1}H NMR. ^{1}H NMR (400 MHz, Chloroform-*d*) δ 7.37 – 7.27 (comp, 3H, major; comp, 3H, minor; overlap), 7.25 – 7.16 (comp, 2H, major; comp, 2H, minor; overlap), 6.69 – 6.52 (comp, 1H, major; comp, 1H, minor; overlap), 4.24 – 4.11 (comp, 2H, major; comp, 2H, minor; overlap), 3.50 – 3.22 (comp, 8H, major; comp, 8H, minor; overlap), 2.69 – 2.43 (comp, 4H, major; comp, 4H, minor; overlap), 1.84 – 1.72 (comp, 2H, major; comp, 2H, minor; overlap), 1.48 – 1.41 (comp, 9H, major; comp, 9H, minor; overlap), 1.31 – 1.25 (comp, 3H, major; comp, 3H, minor; overlap). ^{13}C NMR (101 MHz, $CDCl_3$) δ 172.0, 171.7, 155.6, 155.4, 136.9, 134.7, 133.6, 131.4, 130.6, 129.0, 128.6, 128.3, 128.0, 127.0, 126.7, 79.2, 64.8, 60.6, 60.4, 56.8, 56.4, 56.1, 56.0, 55.7, 54.7, 54.5, 46.5, 46.1, 45.1, 41.0, 35.4, 28.5, 28.4, 28.0, 14.3, 14.2. Peak overlapping was observed. HRMS (ESI) *m/z*: $[M+H]^+$ Calcd for $C_{23}H_{34}N_2O_4$ 403.2591; Found 403.2581.

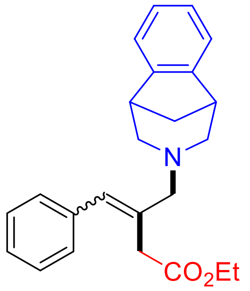

Ethyl 4-phenyl-3-((1,2,4,5-tetrahydro-3*H*-1,5-methanobenzo[*d*]azepin-3-yl)methyl)but-3-enoate (6i)

Following General Procedure D on 0.2 mmol scale. Column chromatography on silica gel (eluent: PE/EA = 30:1) afforded the title product in 60% yield (43.4 mg) as a

colorless oil. *E:Z* = 45:55, the ratio was determined by ^{1}H NMR. **6i**: ^{1}H NMR (500 MHz, Chloroform-*d*) δ 7.31 – 7.08 (comp, 9H, major; comp, 9H, minor; overlap), 6.37 (comp, 1H, major; comp, 1H, minor; overlap), 4.02 – 3.95 (comp, 2H, major; comp, 2H, minor; overlap), 3.24 – 2.99 (comp, 4H, major; comp, 4H, minor; overlap), 2.84 – 2.64 (comp, 4H, major; comp, 4H, minor; overlap), 2.51 – 2.41 (comp, 1H, major; comp, 1H, minor; overlap), 2.36 – 2.18 (comp, 2H, major; comp, 2H, minor; overlap), 1.72 – 1.58 (comp, 1H, major; comp, 1H, minor; overlap), 1.22 – 1.15 (comp, 3H, major; comp, 3H, minor; overlap). ^{13}C NMR (126 MHz, $CDCl_3$) δ 172.0, 171.7, 146.2, 137.1, 129.1, 128.6, 128.2, 127.9, 126.7, 126.5, 126.3, 121.4, 121.3, 63.5, 60.3, 60.1, 56.4, 56.3, 55.6, 43.7, 41.2, 40.1, 14.2, 14.2. Peak overlapping was observed. HRMS (ESI) *m/z*: $[M+H]^+$ Calcd for $C_{24}H_{27}NO_2$ 362.2115; Found 362.2120.

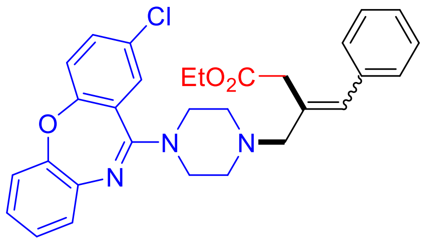

Ethyl 3-((4-(2-chlorodibenzo[*b,f*][1,4]oxazepin-11-yl)piperazin-1-yl)methyl)-4-phenylbut-3-enoate (6j)

Following General Procedure D on 0.2 mmol scale. Diazo compound (0.4 mmol) was used. Column chromatography on silica gel (eluent: PE/EA = 30:1) afforded the title product in 43% yield (44.1 mg) as a colorless oil. *E:Z* = 45:55, the ratio was determined by ^{1}H NMR. ^{1}H NMR (500 MHz, Chloroform-*d*) δ 7.38 – 7.05 (comp, 11H, major; comp, 11H, minor; overlap), 7.04 – 6.89 (comp, 1H, major; comp, 1H, minor; overlap), 6.75 – 6.57 (comp, 1H, major; comp, 1H, minor; overlap), 4.18 (q, *J* = 7.2 Hz, 2H, major; q, *J* = 7.2 Hz, 2H, minor; overlap), 3.68 – 3.39 (comp, 4H, major; comp, 4H, minor; overlap), 3.38 – 3.26 (comp, 3H, major; comp, 3H, minor; overlap), 3.19 (s, 1H, major; s, 1H, minor; overlap), 2.72 – 2.37 (comp, 4H, major; comp, 4H, minor; overlap), 1.31 – 1.26 (comp, 3H, major; comp, 3H, minor; overlap). ^{13}C NMR (126 MHz, $CDCl_3$) δ 172.0, 171.7, 159.3, 159.2, 158.9, 158.8, 151.9, 151.8, 140.3, 140.2, 136.8, , 133.4, 132.5, 132.4, 132.3, 131.4, 130.3, 130.2, 129.2, 129.1, 129.0, 128.6, 128.3, 128.1, 127.1,

127.0, 126.8, 125.8, 125.7, 125.1, 125.0, 124.4, 124.3, 122.7, 122.6, 120.1, 120.0, 65.8, 60.7, 60.5, 57.3, 52.9, 52.8, 47.5, 41.2, 35.8, 14.4, 14.3. Peak overlapping was observed. HRMS (ESI) *m/z*: $[M+H]^+$ Calcd for $C_{30}H_{30}ClN_3O_3$ 516.2048; Found 516.2047.

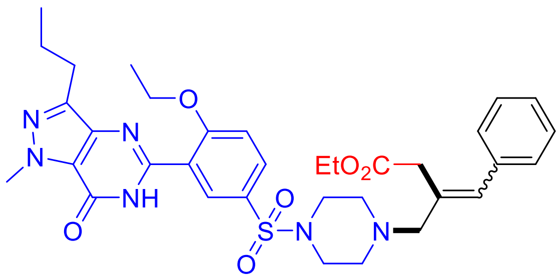

Ethyl 3-((4-((4-ethoxy-3-(1-methyl-7-oxo-3-propyl-6,7-dihydro-1*H*-pyrazolo[4,3-*d*]pyrimidin-5-yl)phenyl)sulfonyl)piperazin-1-yl)methyl)-4-phenylbut-3-enoate (6k)

Following General Procedure D on 0.2 mmol scale. Column chromatography on silica gel (eluent: PE/EA = 30:1) afforded the title product in 50% yield (66.3 mg) as a pale yellow oil. *E:Z* = 50:50, the ratio was determined by ^{1}H NMR. ^{1}H NMR (400 MHz, Chloroform-*d*) δ 10.88 (s, 1H, major; s, 1H, minor, overlap), 8.94 – 8.65 (comp, 1H, major; comp, 1H, minor; overlap), 7.93 – 7.70 (comp, 1H, major; comp, 1H, minor; overlap), 7.32 – 7.21 (comp, 4H, major; comp, 4H, minor; overlap), 7.19 – 7.11 (comp, 2H, major; comp, 2H, minor; overlap), 6.71 – 6.47 (comp, 1H, major; comp, 1H, minor; overlap), 4.38 (q, *J* = 7.0 Hz, 2H, major; q, *J* = 7.0 Hz, 2H, minor; overlap), 4.26 (s, 3H, major; s, 3H, minor; overlap), 4.08 – 3.91 (comp, 2H, major; comp, 2H, minor; overlap), 3.25 – 2.89 (comp, 10H, major; comp, 10H, minor; overlap), 2.61 – 2.39 (comp, 4H, major; comp, 4H, minor; overlap), 1.92 – 1.80 (comp, 1H, major; comp, 1H, minor; overlap), 1.64 (t, *J* = 6.9 Hz, 3H, major; t, *J* = 6.9 Hz, 3H, minor; overlap), 1.18 – 1.09 (comp, 3H, major; comp, 3H, minor; overlap), 1.06 – 0.98 (comp, 3H, major; comp, 3H, minor; overlap). ^{13}C NMR (126 MHz, $CDCl_3$) δ 171.6, 171.4, 159.3, 153.7, 153.6, 146.9, 146.5, 138.3, 136.5, 132.7, 132.6, 131.7, 131.6, 131.5, 131.0, 129.1, 128.8, 128.4, 128.3, 128.1, 127.1, 126.9, 124.5, 124.4, 121.1, 121.0, 113.0, 66.0, 65.2, 60.5, 60.4, 56.8, 51.9, 46.2, 46.1, 41.0, 38.2, 35.5, 27.7, 22.3, 22.2, 14.6, 14.5, 14.1, 14.0, 14.0. Peak overlapping was observed. HRMS (ESI) *m/z*: $[M+H]^+$ Calcd for $C_{34}H_{42}N_6O_6S$ 663.2959; Found 663.2963.

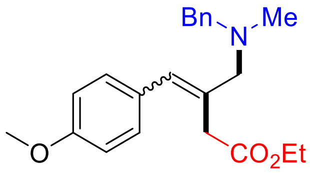

Ethyl 3-((benzyl(methyl)amino)methyl)-4-(4-methoxyphenyl)but-3-enoate (6l)

Following General Procedure D on 0.2 mmol scale. Column chromatography on silica gel (eluent: PE/EA = 30:1) afforded the title product in 66% yield (46.7 mg) as a colorless oil. *E:Z* = 42:58, the ratio was determined by ^{1}H NMR. ^{1}H NMR (400 MHz, Chloroform-*d*) δ 7.34 – 7.14 (comp, 7H, major; comp, 7H, minor; overlap), 6.86 (d, *J* = 8.3 Hz, 2H, major; d, *J* = 8.3 Hz, 2H, minor; overlap), 6.64 – 6.49 (comp, 1H, major; comp, 1H, minor; overlap), 4.12 (q, *J* = 7.0 Hz, 2H, major; q, *J* = 7.0 Hz, 2H, minor; overlap), 3.83 – 3.78 (comp, 3H, major; comp, 3H, minor; overlap), 3.53 – 3.13 (comp, 6H, major; comp, 6H, minor; overlap), 2.19 – 2.03 (comp, 3H, major; comp, 3H, minor; overlap), 1.25 (t, *J* = 7.8 Hz, 3H, major; t, *J* = 7.8 Hz, 3H, minor; overlap). ^{13}C NMR (101 MHz, $CDCl_3$) δ 172.1, 171.8, 158.6, 158.4, 139.3, 133.4, 132.1, 131.3, 130.5, 130.3, 129.8, 129.6, 129.2, 128.9, 128.8, 128.3, 128.2, 128.1, 126.9, 126.8, 113.7, 113.4, 64.9, 62.1, 62.0, 60.6, 60.4, 56.5, 55.3, 42.0, 41.9, 41.1, 35.4, 14.3, 14.2. Peak overlapping was observed. HRMS (ESI) *m/z*: $[M+H]^+$ Calcd for $C_{22}H_{27}NO_3$ 354.2064; Found 354.2072.

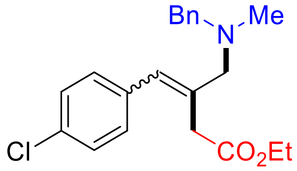

Ethyl 3-((benzyl(methyl)amino)methyl)-4-(4-chlorophenyl)but-3-enoate (6m)

Following General Procedure D on 0.2 mmol scale. Column chromatography on silica gel (eluent: PE/EA = 30:1) afforded the title product in 58% yield (41.2 mg) as a colorless oil. *E:Z* = 43:57, the ratio was determined by ^{1}H NMR. ^{1}H NMR (500 MHz, Chloroform-*d*) δ 7.37 – 7.10 (comp, 9H, major; comp, 9H, minor; overlap), 6.75 – 6.44 (comp, 1H, major; comp, 1H, minor; overlap), 4.27 – 4.01 (comp, 2H, major; comp, 2H, minor; overlap), 3.52 (s, 1H, major; s, 1H, minor; overlap), 3.47 – 3.18 (comp, 4H,

major; comp, 4H, minor; overlap), 3.17 – 3.11 (comp, 1H, major; comp, 1H, minor; overlap), 2.18 – 2.03 (comp, 3H, major; comp, 3H, minor; overlap), 1.28 – 1.22 (comp, 3H, major; comp, 3H, minor; overlap). ^{13}C NMR (126 MHz, $CDCl_3$) δ 171.8, 171.5, 139.2, 135.5, 135.4, 134.5, 132.7, 132.5, 130.4, 129.9, 129.6, 128.9, 128.8, 128.4, 128.3, 128.2, 128.1, 127.0, 64.7, 62.2, 62.0, 60.7, 60.5, 56.4, 42.1, 42.0, 41.0, 35.4, 14.3, 14.2. Peak overlapping was observed. HRMS (ESI) *m/z*: $[M+H]^+$ Calcd for $C_{21}H_{24}ClNO_2$ 358.1568; Found 358.1560.

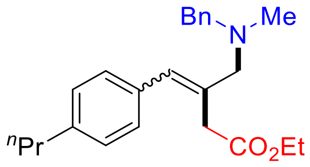

Ethyl 3-((benzyl(methyl)amino)methyl)-4-(4-propylphenyl)but-3-enoate (6n)

Following General Procedure D on 0.2 mmol scale. Column chromatography on silica gel (eluent: PE/EA = 30:1) afforded the title product in 62% yield (45.3 mg) as a colorless oil. *E*:*Z* = 50:50, the ratio was determined by ^{1}H NMR. ^{1}H NMR (500 MHz, Chloroform-*d*) δ 7.37 – 7.12 (comp, 9H, major; comp, 9H, minor; overlap), 6.71 – 6.49 (comp, 1H, major; comp, 1H, minor; overlap), 4.27 – 4.05 (comp, 2H, major; comp, 2H, minor; overlap), 3.53 (s, 1H, major; s, 1H, minor; overlap), 3.47 – 3.26 (comp, 4H, major; comp, 4H, minor; overlap), 3.16 (s, 1H, major; s, 1H, minor; overlap), 2.63 – 2.50 (comp, 2H, major; comp, 2H, minor; overlap), 2.19 – 2.02 (comp, 3H, major; comp, 3H, minor; overlap), 1.70 – 1.61 (comp, 2H, major; comp, 2H, minor; overlap), 1.27 – 1.22 (comp, 3H, major; comp, 3H, minor; overlap), 1.02 – 0.91 (comp, 3H, major; comp, 3H, minor; overlap). ^{13}C NMR (126 MHz, $CDCl_3$) δ 172.1, 171.8, 141.5, 141.2, 134.3, 134.3, 129.0, 128.9, 128.8, 128.5, 128.4, 128.2, 128.1, 128.1, 126.9, 64.9, 62.1, 61.9, 60.6, 60.4, 56.5, 42.0, 41.9, 41.1, 37.8, 37.7, 35.4, 24.5, 14.3, 14.2, 13.9, 13.8. Peak overlapping was observed. HRMS (ESI) *m/z*: $[M+H]^+$ Calcd for $C_{24}H_{31}NO_2$ 366.2428; Found 366.2421.

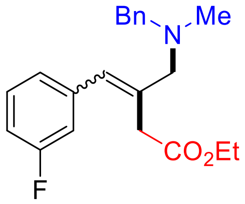

Ethyl 3-((benzyl(methyl)amino)methyl)-4-(3-fluorophenyl)but-3-enoate (6o)

Following General Procedure D on 0.2 mmol scale. Column chromatography on silica gel (eluent: PE/EA = 30:1) afforded the title product in 66% yield (45.1 mg) as a colorless oil. *E:Z* = 41:59, the ratio was determined by ^{1}H NMR. ^{1}H NMR (400 MHz, Chloroform-*d*) δ 7.36 – 7.21 (comp, 6H, major; comp, 6H, minor; overlap), 7.09 – 6.88 (comp, 3H, major; comp, 3H, minor; overlap), 6.68 – 6.52 (comp, 1H, major; comp, 1H, minor; overlap), 4.19 – 4.08 (comp, 2H, major; comp, 2H, minor; overlap), 3.53 – 3.11 (comp, 6H, major; comp, 6H, minor; overlap), 2.19 – 2.01 (comp, 3H, major; comp, 3H, minor; overlap), 1.27 – 1.22 (comp, 3H, major; comp, 3H, minor; overlap). ^{13}C NMR (101 MHz, $CDCl_3$) δ 171.7, 171.4, 163.9, 163.8, 161.5, 161.3, 139.3, 139.2, 139.1, 135.9, 134.9, 130.5, 130.4, 129.7, 129.6, 129.6, 129.5, 129.4, 128.9, 128.8, 128.2, 128.1, 127.0, 126.9, 124.9, 124.8, 124.4, 124.3, 116.0, 115.8, 115.5, 115.3, 113.9, 113.7, 113.5, 64.6, 62.2, 62.1, 60.7, 60.5, 56.5, 42.1, 42.0, 41.0, 35.4, 14.2, 14.1. ^{19}F NMR (376 MHz, $CDCl_3$) δ -113.40, -113.65. Peak overlapping was observed. HRMS (ESI) *m/z*: $[M+H]^+$ Calcd for $C_{21}H_{24}FNO_2$ 342.1864; Found 342.1863.

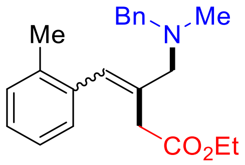

Ethyl 3-((benzyl(methyl)amino)methyl)-4-(*o*-tolyl)but-3-enoate (6p)

Following General Procedure D on 0.2 mmol scale. Diazo compound (0.4 mmol) was used. Column chromatography on silica gel (eluent: PE/EA = 30:1) afforded the title product in 60% yield (40.5 mg) as a colorless oil. *E:Z* = 50:50, the ratio was determined by ^{1}H NMR. ^{1}H NMR (500 MHz, Chloroform-*d*) δ 7.35 – 7.06 (comp, 9H, major; comp, 9H, minor; overlap), 6.71 – 6.49 (comp, 1H, major; comp, 1H, minor; overlap), 4.21 – 4.03 (comp, 2H, major; comp, 2H, minor; overlap), 3.55 (s, 1H, major; s, 1H, minor;

overlap), 3.42 – 3.32 (comp, 2H, major; comp, 2H, minor; overlap), 3.25 – 3.14 (comp, 2H, major; comp, 2H, minor; overlap), 3.09 (s, 1H, major; s,1H, minor; overlap), 2.23 (s, 1H, major; s, 1H, minor; overlap), 2.21 – 2.00 (comp, 3H, major; comp, 3H, minor; overlap), 1.27 – 1.19 (comp, 3H, major; comp, 3H, minor; overlap). ^{13}C NMR (126 MHz, $CDCl_3$) δ 172.0, 171.7, 139.4, 136.4, 136.4, 136.3, 136.3, 134.4, 133.7, 131.0, 130.2, 129.7, 129.6, 129.5, 128.9, 128.8, 128.7, 128.2, 128.1, 127.2, 127.1, 126.9, 126.8, 125.6, 125.3, 64.1, 62.1, 61.9, 60.5, 56.6, 42.0, 41.9, 40.5, 35.2, 20.0, 19.9, 14.3, 14.2. Peak overlapping was observed. HRMS (ESI) *m/z*: $[M+H]^+$ Calcd for $C_{22}H_{27}NO_2$ 338.2115; Found 338.2122.

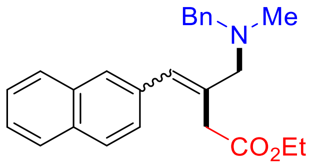

Ethyl 3-((benzyl(methyl)amino)methyl)-4-(naphthalen-2-yl)but-3-enoate (6q)

Following General Procedure D on 0.2 mmol scale. Column chromatography on silica gel (eluent: PE/EA = 30:1) afforded the title product in 70% yield (52.3 mg) as a colorless oil. *E:Z* = 45:55, the ratio was determined by ^{1}H NMR. ^{1}H NMR (500 MHz, Chloroform-*d*) δ 7.86 – 7.64 (comp, 4H, major; comp, 4H, minor; overlap), 7.51 – 7.21 (comp, 8H, major; comp, 8H, minor; overlap), 6.91 – 6.68 (comp, 1H, major; comp, 1H, minor; overlap), 4.28 – 4.09 (comp, 2H, major; comp, 2H, minor; overlap), 3.57 (s, 1H, major; s, 1H, minor; overlap), 3.53 – 3.28 (comp, 4H, major; comp, 4H, minor; overlap), 3.22 (s, 1H, major; s, 1H, minor; overlap), 2.26 – 2.03 (comp, 3H, major; comp, 3H, minor; overlap), 1.30 – 1.24 (comp, 3H, major; comp, 3H, minor; overlap). ^{13}C NMR (126 MHz, $CDCl_3$) δ 172.0, 171.7, 139.4, 134.6, 134.5, 133.3, 133.2, 132.4, 132.2, 131.7, 130.9, 128.9, 128.8, 128.2, 128.2, 128.0, 127.9, 127.9, 127.8, 127.6, 127.6, 127.5, 127.5, 127.3, 127.0, 127.0, 126.9, 126.1, 125.8, 64.8, 62.2, 62.0, 60.7, 60.5, 56.6, 42.1, 42.0, 41.1, 35.5, 14.3, 14.2. Peak overlapping was observed. HRMS (ESI) *m/z*: $[M+H]^+$ Calcd for $C_{25}H_{27}NO_2$ 374.2115; Found 374.2110.

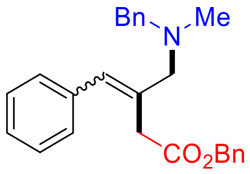

Benzyl 3-((benzyl(methyl)amino)methyl)-4-phenylbut-3-enoate (6r)

Following General Procedure D on 0.2 mmol scale. Diazo compound (0.4 mmol) was used. Column chromatography on silica gel (eluent: PE/EA = 30:1) afforded the title product in 57% yield (43.9 mg) as a colorless oil. *E*:*Z* = 50:50, the ratio was determined by ^{1}H NMR. ^{1}H NMR (400 MHz, Chloroform-*d*) δ 7.36 – 7.17 (comp, 15H, major; comp, 15H, minor; overlap), 6.74 – 6.54 (comp, 1H, major; comp, 1H, minor; overlap), 5.16 – 5.09 (comp, 2H, major; comp, 2H, minor; overlap), 3.52 (s, 1H, major; s, 1H, minor; overlap), 3.46 – 3.40 (comp, 2H, major; comp, 2H, minor; overlap), 3.37 (s, 1H, major; s, 1H, minor; overlap), 3.32 – 3.25 (comp, 1H, major; comp, 1H, minor; overlap), 3.17 (s, 1H, major; s, 1H, minor; overlap), 2.16 – 2.01 (comp, 3H, major; comp, 3H, minor; overlap). ^{13}C NMR (101 MHz, $CDCl_3$) δ 171.8, 171.5, 136.9, 136.8, 136.0, 129.1, 129.0, 128.9, 128.6, 128.5, 128.3, 128.2, 128.1, 128.0, 127.0, 126.9, 126.8, 66.5, 66.3, 64.7, 62.0, 61.8, 41.9, 41.8, 40.9, 35.4. Peak overlapping was observed. HRMS (ESI) *m/z*: $[M+H]^+$ Calcd for $C_{26}H_{27}NO_2$ 386.2115; Found 386.2106.

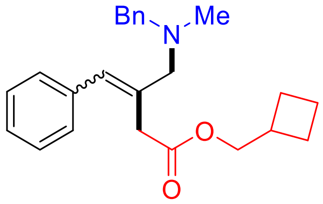

Cyclobutylmethyl 3-((benzyl(methyl)amino)methyl)-4-phenylbut-3-enoate (6s)

Following General Procedure D on 0.2 mmol scale. Column chromatography on silica gel (eluent: PE/EA = 30:1) afforded the title product in 81% yield (58.9 mg) as a colorless oil. *E*:*Z* = 44:56, the ratio was determined by ^{1}H NMR. ^{1}H NMR (500 MHz, Chloroform-*d*) δ 7.36 – 7.19 (comp, 10H, major; comp, 10H, minor; overlap), 6.73 – 6.56 (comp, 1H, major; comp, 1H, minor; overlap), 4.11 – 4.02 (comp, 2H, major; comp, 2H, minor; overlap), 3.55 (s, 1H, major; s, 1H, minor; overlap), 3.47 – 3.33 (comp, 3H, major; comp, 3H, minor; overlap), 3.29 (s, 1H, major; s, 1H, minor; overlap), 3.18 (s,

1H, major; s,1H, minor; overlap), 2.66 – 2.56 (comp, 1H, major; comp, 1H, minor; overlap), 2.24 – 2.13 (comp, 2H, major; comp, 2H, minor; overlap), 2.05 (s, 3H, major; s, 3H, minor; overlap), 1.94 – 1.84 (comp, 2H, major; comp, 2H, minor; overlap), 1.81 – 1.72 (comp, 2H, major; comp, 2H, minor; overlap). ^{13}C NMR (126 MHz, $CDCl_3$) δ 172.2, 171.9, 137.0, 136.9, 129.1, 128.9, 128.8, 128.6, 128.3, 128.2, 128.1, 128.0, 127.0, 126.7, 68.5, 68.4, 64.7, 62.0, 61.9, 56.4, 42.0, 41.9, 40.9, 35.4, 34.1, 34.1, 24.8, 18.5, 18.4. Peak overlapping was observed. HRMS (ESI) *m/z*: $[M+H]^+$ Calcd for $C_{24}H_{29}NO_2$ 364.2271; Found 364.2267.

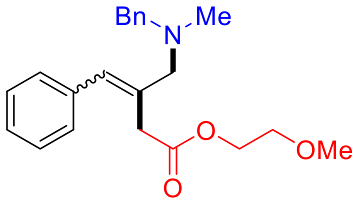

2-Methoxyethyl 3-((benzyl(methyl)amino)methyl)-4-phenylbut-3-enoate (6t)

Following General Procedure D on 0.2 mmol scale. Diazo compound (0.4 mmol) was used. Column chromatography on silica gel (eluent: PE/EA = 30:1) afforded the title product in 66% yield (46.6 mg) as a colorless oil. *E:Z* = 50:50, the ratio was determined by ^{1}H NMR. ^{1}H NMR (400 MHz, Chloroform-*d*) δ 7.40 – 7.20 (comp, 10H, major; comp, 10H, minor; overlap), 6.76 – 6.53 (comp, 1H, major; comp, 1H, minor; overlap), 4.32 – 4.13 (comp, 2H, major; comp, 2H, minor; overlap), 3.63 – 3.51 (comp, 3H, major; comp, 3H, minor; overlap), 3.48 – 3.33 (comp, 6H, major; comp, 6H, minor; overlap), 3.29 (s, 1H, major; s, 1H, minor; overlap), 3.18 (s, 1H, major; s, 1H, minor; overlap), 2.19 – 2.03 (comp, 3H, major; comp, 3H, minor; overlap). ^{13}C NMR (101 MHz, $CDCl_3$) δ 172.0, 171.7, 137.0, 136.9, 129.1, 129.0, 128.9, 128.6, 128.3, 128.2, 128.1, 128.0, 127.0, 126.9, 126.7, 70.5, 70.4, 64.7, 63.6, 63.5, 62.0, 61.9, 59.1, 59.0, 56.4, 42.0, 41.9, 40.8, 35.2. Peak overlapping was observed. HRMS (ESI) *m/z*: $[M+H]^+$ Calcd for $C_{22}H_{27}NO_3$ 354.2064; Found 354.2054.

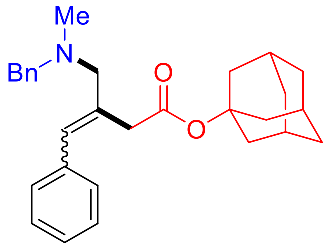

(3*s*,5*s*,7*s*)-Adamantan-1-yl 3-((benzyl(methyl)amino)methyl)-4-phenylbut-3-enoate (6u)

Following General Procedure D on 0.2 mmol scale. Column chromatography on silica gel (eluent: PE/EA 30:1) afforded the title product in 82% yield (70.5 mg) as a colorless oil. *E:Z* = 55:45, the ratio was determined by ^{1}H NMR. ^{1}H NMR (500 MHz, Chloroform-*d*) δ 7.39 – 7.19 (comp, 10H, major; comp, 10H, minor; overlap), 6.76 – 6.52 (comp, 1H, major; comp, 1H, minor; overlap), 3.57 (s, 1H, major; s, 1H, minor; overlap), 3.41 (s, 1H, major; s, 1H, minor; overlap), 3.28 (s, 3H, major; s, 3H, minor; overlap), 3.19 (s, 1H, major; s, 1H, minor; overlap), 2.23 – 2.05 (comp, 12H, major; comp, 12H, minor; overlap), 1.69 – 1.62 (comp, 6H, major; comp, 6H, minor; overlap). ^{13}C NMR (126 MHz, $CDCl_3$) δ 171.1, 170.7, 137.2, 137.0, 129.1, 128.9, 128.6, 128.3, 128.2, 128.1, 128.0, 126.9, 126.6, 80.8, 80.5, 64.6, 62.0, 61.8, 56.5, 42.3, 42.0, 41.4, 41.3, 36.8, 36.3, 36.2, 30.9, 30.8. Peak overlapping was observed. HRMS (ESI) *m/z*: $[M+H]^+$ Calcd for $C_{29}H_{35}NO_2$ 430.2741; Found 430.2733.

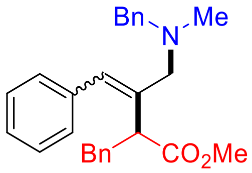

Methyl 2-benzyl-3-((benzyl(methyl)amino)methyl)-4-phenylbut-3-enoate (6v)

Following General Procedure D on 0.2 mmol scale. Column chromatography on silica gel (eluent: PE/EA = 30:1) afforded the title product in 78% yield (44.6 mg) as a colorless oil. *E:Z* = 22:78, the ratio was determined by ^{1}H NMR. ^{1}H NMR (500 MHz, Chloroform-*d*) δ 7.39 – 7.10 (comp, 15H, major; comp, 15H, minor; overlap), 6.83 – 6.72 (comp, 1H, major; comp, 1H, minor; overlap), 4.08 – 3.91 (comp, 1H, major; comp, 1H, minor; overlap), 3.61 (s, 3H, major; s, 3H, minor; overlap), 3.54 – 3.44 (comp, 1H,

major; comp, 1H, minor; overlap), 3.36 – 3.06 (comp, 3H, major; comp, 3H, minor; overlap), 3.02 – 2.77 (comp, 2H, major; comp, 2H, minor; overlap), 2.15 – 1.99 (comp, 3H, major; comp, 3H, minor; overlap). ^{13}C NMR (101 MHz, $CDCl_3$) δ 174.3, 139.7, 139.2, 138.5, 137.0, 130.4, 129.3, 129.1, 129.0, 129.0, 128.8, 128.6, 128.3, 128.1, 128.0, 126.9, 126.7, 126.2, 62.1, 56.5, 51.7, 50.1, 41.9, 38.3. Peak overlapping was observed. HRMS (ESI) *m/z*: $[M+H]^+$ Calcd for $C_{27}H_{29}NO_2$ 400.2271; Found 400.2262.

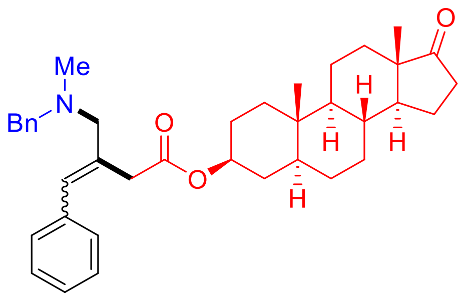

(3*S*,5*S*,8*R*,9*S*,10*S*,13*S*,14*S*)-10,13-Dimethyl-17-oxohexadecahydro-1*H*-cyclopenta[*a*]phenanthren-3-yl 3-((benzyl(methyl)amino)methyl)-4-phenylbut-3-enoate (6w)

Following General Procedure D on 0.2 mmol scale. Column chromatography on silica gel (eluent: PE/EA = 30:1) afforded the title product in 84% yield (95.4 mg) as a colorless oil. *E:Z* = 45:55, the ratio was determined by ^{1}H NMR. ^{1}H NMR (400 MHz, Chloroform-*d*) δ 7.36 – 7.20 (comp, 10H, major; comp, 10H, minor; overlap), 6.74 – 6.53 (comp, 1H, major; comp, 1H, minor; overlap), 4.82 – 4.52 (comp, 1H, major; comp, 1H, minor; overlap), 3.54 (s, 1H, major; s, 1H, minor; overlap), 3.39 (s, 1H, major; s, 1H, minor; overlap), 3.36 – 3.30 (comp, 2H, major; comp, 2H, minor; overlap), 3.28 (s, 1H, major; s, 1H, minor; overlap), 3.17 (s, 1H, major; s, 1H, minor; overlap), 2.47 – 2.39 (comp, 1H, major; comp, 1H, minor; overlap), 2.22 – 2.01 (comp, 4H, major; comp, 4H, minor; overlap), 1.96 – 1.89 (comp, 1H, major; comp, 1H, minor; overlap), 1.82 – 1.70 (comp, 4H, major; comp, 4H, minor; overlap), 1.66 – 1.45 (comp, 5H, major; comp, 5H, minor; overlap), 1.35 – 1.23 (comp, 7H, major; comp, 7H, minor; overlap), 1.07 – 0.94 (comp, 2H, major; comp, 2H, minor; overlap), 0.86 – 0.83 (comp, 6H, major; comp, 6H, minor; overlap), 0.75 – 0.68 (m, 1H). ^{13}C NMR (126 MHz, $CDCl_3$) δ 221.3, 171.5, 171.2, 137.0, 129.1, 129.0, 128.9, 128.6, 128.3, 128.2, 128.1, 128.0, 126.9, 126.7, 73.8,

73.6, 64.7, 62.1, 61.9, 56.5, 54.3, 51.4, 47.8, 44.7, 44.6, 42.0, 41.2, 36.8, 36.7, 35.9, 35.7, 35.6, 35.0, 33.9, 33.8, 31.5, 30.8, 28.3, 27.4, 27.3, 21.8, 20.5, 13.8, 12.3. Peak overlapping was observed. HRMS (ESI) *m/z*: $[M+H]^+$ Calcd for $C_{38}H_{49}NO_3$ 568.3785; Found 568.3786.

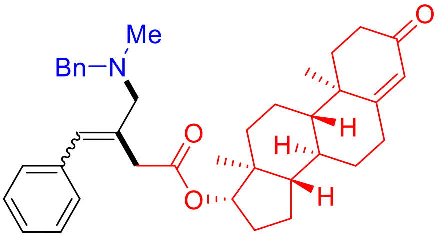

(8*R*,9*S*,10*R*,13*S*,14*S*,17*S*)-10,13-Dimethyl-3-oxo-2,3,6,7,8,9,10,11,12,13,14,15,16,17-tetradecahydro-1*H*-cyclopenta[*a*]phenanthren-17-yl 3-((benzyl(methyl)amino)methyl)-4-phenylbut-3-enoate (6x)

Following General Procedure D on 0.2 mmol scale. Column chromatography on silica gel (eluent: PE/EA = 30:1) afforded the title product in 56% yield (63.4 mg) as a colorless oil. *E:Z* = 50:50, the ratio was determined by ^{1}H NMR. ^{1}H NMR (500 MHz, Chloroform-*d*) δ 7.36 – 7.19 (comp, 10H, major; comp, 10H, minor; overlap), 6.74 – 6.51 (comp, 1H, major; comp, 1H, minor; overlap), 5.73 (s, 1H, major; s, 1H, minor; overlap), 4.62 (q, *J* = 8.5 Hz, 1H, major; q, *J* = 8.5 Hz,1H, minor; overlap), 3.54 (s, 1H, major; s, 1H, minor; overlap), 3.38 (s, 3H, major; s, 3H, minor; overlap), 3.28 (s, 1H, major; s, 1H, minor; overlap), 3.18 (s, 1H, major; s, 1H, minor; overlap), 2.45 – 2.16 (comp, 7H, major; comp, 7H, minor; overlap), 2.05 – 2.00 (comp, 2H, major; comp, 2H, minor; overlap), 1.87 – 1.64 (comp, 6H, major; comp, 6H, minor; overlap), 1.60 – 1.54 (comp, 2H, major; comp, 2H, minor; overlap), 1.36 – 1.31 (comp, 2H, major; comp, 2H, minor; overlap), 1.19 (s, 3H, major; s, 3H, minor; overlap), 1.10 – 1.02 (comp, 2H, major; comp, 2H, minor; overlap), 0.98 – 0.93 (comp, 1H, major; comp, 1H, minor; overlap), 0.87 – 0.81 (comp, 3H, major; comp, 3H, minor; overlap). ^{13}C NMR (126 MHz, $CDCl_3$) δ 199.5, 172.0, 171.6, 171.2, 171.0, 137.0, 136.9, 129.1, 128.9, 128.8, 128.6, 128.3, 128.2, 128.2, 128.1, 127.0, 126.7, 123.9, 82.8, 82.5, 64.7, 62.0, 61.8, 56.5, 53.7, 53.6, 50.3, 50.20, 42.5, 42.4, 42.0, 41.0, 38.6, 36.7, 36.6, 36.6, 35.7, 35.4, 33.9, 32.7, 31.5, 27.6, 27.5, 27.4, 23.5, 23.4, 21.2, 20.6, 20.5, 17.4, 12.2, 12.1, 12.0. Peak overlapping was observed. HRMS (ESI) *m/z*: $[M+H]^+$ Calcd for $C_{38}H_{47}NO_3$ 566.3629; Found 566.3628.

6. Scale-up reaction and synthetic transformations

(1) Scale-up reaction

Scale-up synthesis of ethyl (*E*)-6-(4-phenylpiperazin-1-yl)hex-4-enoate (4a)

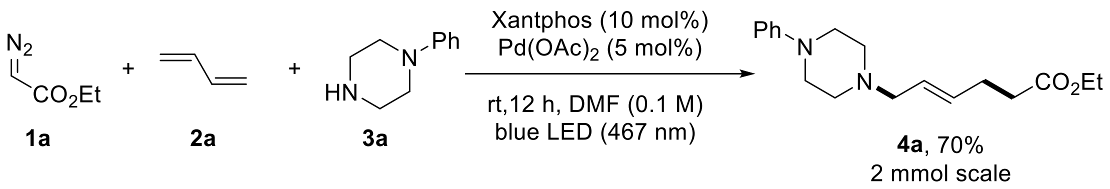

To a reaction tube equipped with a magnetic stirring bar were added $Pd(OAc)_2$ (22.0 mg, 0.1 mmol, 5.0 mol %) and Xantphos (116.0 mg, 0.2 mmol, 10.0 mol %). The tube was capped. After evacuation and backfilling with argon three times, anhydrous DMF (20.0 mL, 0.1 M) was added via a syringe, followed by stirring at room temperature for about 5 minutes. To this reaction system were added 1,3-diene **2a** (4.0 mmol, 2.0 equiv), amine **3a** (324.0 mg, 2.0 mmol, 1.0 equiv), and diazo compound **1a** (342.0 mg, 3.0 mmol, 1.5 equiv). The reaction mixture was irradiated by blue LED at room temperature for 12 h until the diazo compound was consumed completely, as monitored by TLC analysis. The crude mixture was purified by column chromatography on silica gel with DCM/MeOH mixtures as eluent to give the corresponding product **4a** (422.8 mg, 70% yield, *E:Z* = 90:10).

Scale-up synthesis of ethyl 3-((benzyl(methyl)amino)methyl)-4-phenylbut-3-enoate (6a)

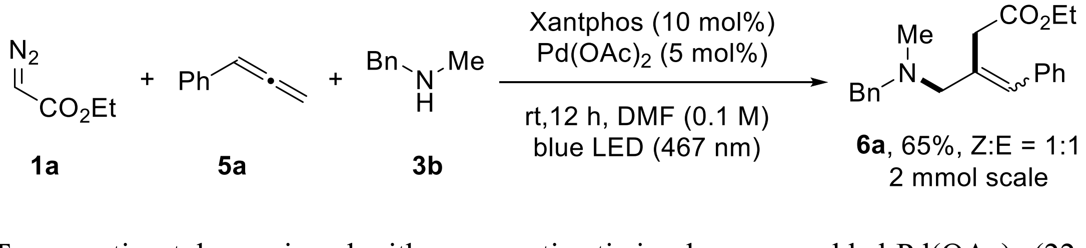

To a reaction tube equipped with a magnetic stirring bar were added $Pd(OAc)_2$ (22.0 mg, 0.1 mmol, 5.0 mol %) and Xantphos (116.0 mg, 0.2 mmol, 10.0 mol %). The tube was capped. After evacuation and backfilling with argon three times, anhydrous DMF (20.0 mL, 0.1 M) was added via a syringe, followed by stirring at room temperature for

about 5 minutes. To this reaction system were added allene **5a** (464.0 mg, 4.0 mmol, 2.0 equiv), amine **3b** (242.0 mg, 2.0 mmol, 1.0 equiv), and diazo compound **1a** (342.0 mg, 3.0 mmol, 1.5 equiv). The reaction mixture was irradiated by blue LED at room temperature for 12 h until the diazo compound was consumed completely, as monitored by TLC analysis. The crude mixture was purified by column chromatography on silica gel with DCM/MeOH mixtures as eluent to give the corresponding product **6a** (420.4 mg, 65% yield, *E:Z* = 1:1).

(2) Synthetic transformations

Synthesis of (*E*)-6-(4-phenylpiperazin-1-yl)hex-4-en-1-ol (7)

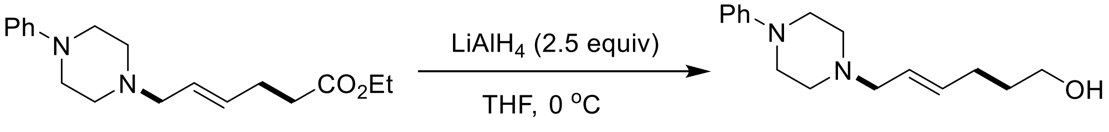

4a **7**, 91%

To a solution of $LiAlH_4$ (2.5 equiv) in anhydrous THF (2.0 mL) was slowly added the solution of **4a** (30.2 mg, 0.1 mmol) in THF (1.0 mL) below 0 °C. After stirring at 0 °C for 1 h, the reaction mixture was quenched by H_2O (10 mL), then extracted with EtOAc (15 mL × 3). The combined organic layers were washed with brine, dried over anhydrous $NaSO_4$ and concentrated in vacuo. The crude mixture was purified by column chromatography on silica gel (eluent: DCM/MeOH 50:1) to give product **7** (pale yellow oil, 91% yield, 23.7 mg). ^{1}H NMR (400 MHz, Chloroform-*d*) δ 7.34 – 7.22 (m, 2H), 7.05 – 6.79 (m, 3H), 5.85 – 5.73 (m, 1H), 5.73 – 5.57 (m, 1H), 3.66 (t, *J* = 6.4 Hz, 2H), 3.55 – 3.05 (m, 7H), 2.84 (t, *J* = 5.0 Hz, 4H), 2.19 (q, *J* = 7.1 Hz, 2H), 1.74 – 1.63 (m, 2H). ^{13}C NMR (101 MHz, $CDCl_3$) δ 150.6, 137.5, 129.2, 123.3, 120.4, 116.5, 62.1, 60.2, 52.3, 48.3, 31.8, 28.8. HRMS (ESI) *m/z*: $[M+H]^+$ Calcd for $C_{16}H_{24}N_2O$ 261.1961; Found 261.1964.

Synthesis of (*E*)-2-methyl-7-(4-phenylpiperazin-1-yl)hept-5-en-2-ol (8)

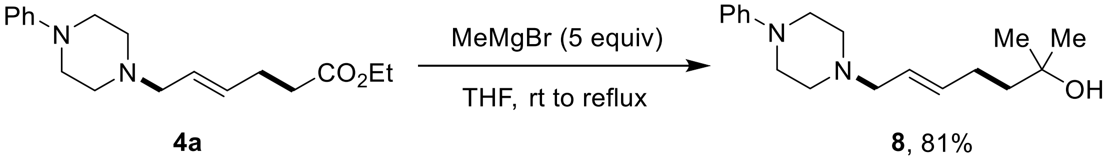

To a solution of methylmagnesium bromide (5.0 equiv) in THF (2.0 mL) was slowly added the solution of **4a** (30.2 mg, 0.1 mmol) in THF (1.0 mL) under rt. The resulting heterogeneous mixture was heated to reflux. After 3 h, a saturated aqueous solution of NH_4Cl was added dropwise at 0 °C with vigorous stirring, and then extracted with EtOAc (15.0 Ml × 3). The combined organic layers were washed with brine, dried over anhydrous $NaSO_4$ and concentrated in vacuo. The crude mixture was purified by column chromatography on silica gel (eluent: DCM/MeOH 40:1) to give product **8** (yellow oil, 81% yield, 23.3 mg). ^{1}H NMR (400 MHz, Chloroform-*d*) δ 7.31 – 7.21 (m, 2H), 6.99 – 6.89 (m, 2H), 6.86 (t, *J* = 7.3 Hz, 1H), 5.77 – 5.65 (m, 1H), 5.65 – 5.49 (m, 1H), 3.35 – 3.20 (m, 4H), 3.08 (d, *J* = 6.6 Hz, 2H), 2.80 – 2.53 (m, 5H), 2.22 – 2.11 (m, 2H), 1.63 – 1.53 (m, 2H), 1.23 (s, 6H). ^{13}C NMR (126 MHz, $CDCl_3$) δ 151.1, 136.1, 129.1, 124.9, 119.9, 116.2, 70.9, 60.6, 52.7, 48.8, 43.1, 29.3, 27.4. HRMS (ESI) *m/z*: $[M+H]^+$ Calcd for $C_{18}H_{28}N_2O$ 289.2274; Found 289.2283.

Synthesis of (*E*)-*N*-benzyl-6-(4-phenylpiperazin-1-yl)hex-4-enamide (9)

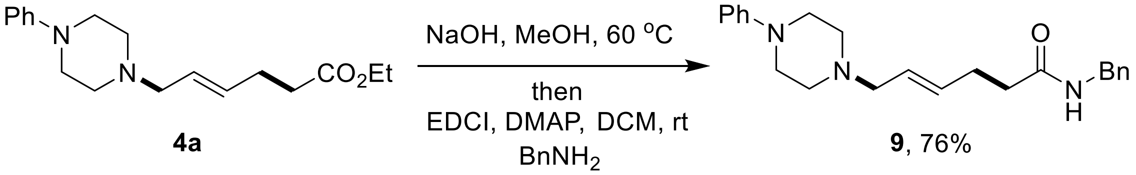

To a solution of **4a** (30.2 mg, 0.1 mmol) in MeOH (2.0 mL) was added NaOH (5.0 equiv). After stirring at 60 °C for 2 h, to the reaction mixture was added H_2O (5.0 mL), followed by the dropwise addition of concentrated hydrochloric acid until pH 7, then extracted with EtOAc (15.0 mL × 3). The combined organic layers were concentrated in vacuo. The resulting crude mixture can be used in the next step.

To a solution of the above-mentioned crude mixture in DCM (2.0 mL) was added 1-(3-dimethylaminopropyl)-3-ethylcarbodiimide hydrochloride (EDCI, 1.5 equiv) and DMAP (0.1 equiv). After stirring for 15 min, benzylamine (1.5 equiv) was added and the reaction was stirred at room temperature for 12 h. After completion of the reaction, the mixture was added by H_2O (10.0 mL), then extracted with EtOAc (15.0 mL × 3). The combined organic layers were washed with brine, dried over anhydrous $NaSO_4$ and concentrated in vacuo. The crude mixture was purified by column chromatography on silica gel (eluent: DCM/MeOH 40:1) to give product **9** (yellow oil, 76% yield, 27.6 mg). ^{1}H NMR (500 MHz, Chloroform-*d*) δ 7.37 – 7.31 (m, 2H), 7.29 – 7.23 (m, 5H), 6.91 (d, *J* = 8.0 Hz, 2H), 6.87 (t, *J* = 7.3 Hz, 1H), 6.06 – 5.90 (m, 1H), 5.73 (dt, *J* = 13.6, 6.4 Hz, 1H), 5.62 (dt, *J* = 15.3, 6.8 Hz, 1H), 4.44 (d, *J* = 5.7 Hz, 2H), 3.24 (t, *J* = 4.9 Hz, 4H), 3.07 (d, *J* = 6.6 Hz, 2H), 2.73 – 2.65 (m, 4H), 2.48 – 2.41 (m, 2H), 2.36 – 2.30 (m, 2H). ^{13}C NMR (126 MHz, $CDCl_3$) δ 172.0, 151.0, 138.4, 134.4, 129.2, 128.7, 127.8, 127.5, 125.9, 120.0, 116.3, 60.4, 52.7, 48.7, 43.6, 36.0, 28.3. HRMS (ESI) *m/z*: $[M+H]^+$ Calcd for $C_{23}H_{29}N_3O$ 364.2383; Found 364.2390.

Synthesis of (*E*)-*N*-methoxy-*N*-methyl-6-(4-phenylpiperazin-1-yl)hex-4-enamide (10)

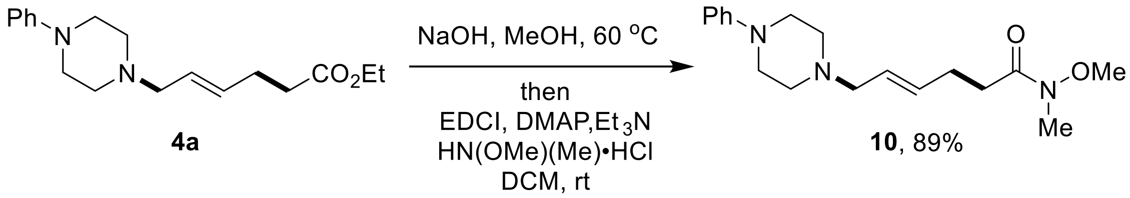

To a solution of **4a** (60.4 mg, 0.2 mmol) in MeOH (2.0 mL) was added NaOH (5.0 equiv). After stirring at 60 °C for 2 h, to the reaction mixture was added H_2O (5.0 mL), followed by the dropwise addition of concentrated hydrochloric acid until pH 7, then extracted with EtOAc (15.0 mL × 3). The combined organic layers were concentrated in vacuo. The resulting crude mixture can be used in the next step.

To a solution of above-mentioned crude mixture in DCM (2.0 mL) was added 1-(3-dimethylaminopropyl)-3-ethylcarbodiimide hydrochloride (EDCI, 1.5 equiv) and DMAP (0.1 equiv). After stirring for 15 min, a solution of *N,O*-dimethylhydroxylamine

hydrochloride (HN(OMe)(Me)Cl, 1.5 equiv) and Et_3N (1.5 equiv) in DCM (2.0 mL) were added, and the reaction mixture was stirred at room temperature for 12 h. After completion of the reaction, to the mixture was added H_2O (10.0 mL), then extracted with EtOAc (15.0 mL × 3). The combined organic layers were washed with brine, dried over anhydrous $NaSO_4$ and concentrated in vacuo. The crude mixture was purified by column chromatography on silica gel (eluent: DCM/MeOH 40:1) to give product **10** (yellow oil, 89% yield, 56.4 mg). ^{1}H NMR (400 MHz, Chloroform-*d*) δ 7.28 – 7.26 (m, 2H), 6.95 – 6.89 (m, 2H), 6.86 (t, *J* = 7.3 Hz, 1H), 5.82 – 5.67 (m, 1H), 5.61 (dt, *J* = 15.4, 6.6 Hz, 1H), 3.69 (s, 3H), 3.24 (t, *J* = 5.0 Hz, 4H), 3.18 (s, 3H), 3.05 (d, *J* = 6.6 Hz, 2H), 2.72 – 2.60 (m, 4H), 2.58 – 2.49 (m, 2H), 2.44 – 2.37 (m, 2H). ^{13}C NMR (126 MHz, $CDCl_3$) δ 173.8, 151.3, 133.6, 129.1, 126.7, 119.7, 116.1, 61.3, 60.8, 53.0, 49.0, 32.2, 31.4, 27.3. HRMS (ESI) *m/z*: $[M+H]^+$ Calcd for $C_{18}H_{27}N_3O_2$ 318.2176; Found 318.2180.

Synthesis of (*E*)-7-(4-phenylpiperazin-1-yl)hept-5-en-2-one (11)

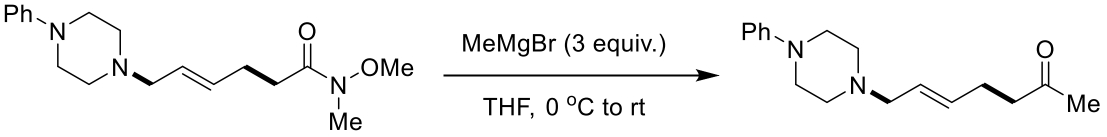

10 **11**, 84%

To a solution of **10** (31.7 mg, 0.1 mmol) in THF (1.0 mL) was added MeMgBr (3.0 equiv) at 0 °C. The mixture was allowed to warm to room temperature. After stirring for 6 h, saturated aqueous solution of NH_4Cl was added dropwise at 0 °C with vigorous stirring, and then extracted with EtOAc (15.0 mL × 3). The combined organic layers were washed with brine, dried over anhydrous $NaSO_4$ and concentrated in vacuo. The crude mixture was purified by column chromatography on silica gel (eluent: DCM/MeOH 30:1) to give product **11** (colorless oil, 84% yield, 22.8 mg). ^{1}H NMR (500 MHz, Chloroform-*d*) δ 7.29 – 7.25 (m, 2H), 6.98 – 6.91 (m, 2H), 6.88 (t, *J* = 7.4 Hz, 1H), 5.71 (dt, *J* = 13.5, 6.4 Hz, 1H), 5.61 (dt, *J* = 14.6, 6.9 Hz, 1H), 3.35 – 3.26 (m, 4H), 3.11 (d, *J* = 6.6 Hz, 2H), 2.78 – 2.67 (m, 4H), 2.56 (t, *J* = 7.3 Hz, 2H), 2.39 – 2.32 (m, 2H), 2.16 (s, 3H). ^{13}C NMR (126 MHz, $CDCl_3$) δ 208.0, 150.9, 134.6, 129.2, 125.3,

120.1, 116.3, 60.4, 52.6, 48.7, 42.8, 30.0, 26.4. HRMS (ESI) *m/z*: $[M+H]^+$ Calcd for $C_{17}H_{24}N_2O$ 273.1961; Found 273.1962.

Synthesis of ethyl 6-(4-phenylpiperazin-1-yl)hexanoate (12)

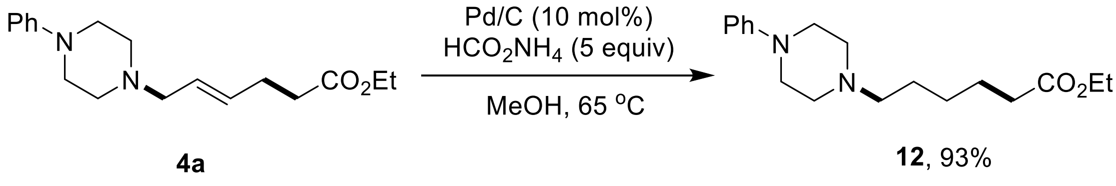

To a solution of **4a** (30.2 mg, 0.1 mmol) in MeOH (2.0 mL) was added HCO_2NH_4 (5.0 equiv). After 5 min, 10% Pd/C was added and the reaction mixture was heated at 65 °C for 2 h. The reaction mixture was filtered through a plug of Celite and washed with MeOH. The resulting filtrate was concentrated under reduced pressure. The crude mixture was purified by column chromatography on silica gel (eluent: DCM/MeOH 50:1) to give product **12** (yellow oil, 93% yield, 28.3 mg). ^1H NMR (400 MHz, Chloroform-*d*) δ 7.30 – 7.21 (m, 2H), 6.98 – 6.89 (m, 2H), 6.89 – 6.80 (m, 1H), 4.13 (q, *J* = 7.1 Hz, 2H), 3.37 – 3.20 (m, 4H), 2.68 (t, *J* = 5.0 Hz, 4H), 2.52 – 2.41 (m, 2H), 2.31 (t, *J* = 7.5 Hz, 2H), 1.72 – 1.55 (m, 4H), 1.45 – 1.33 (m, 2H), 1.26 (t, *J* = 7.1 Hz, 3H). ^{13}C NMR (101 MHz, $CDCl_3$) δ 173.7, 151.1, 129.1, 119.9, 116.2, 60.3, 58.3, 53.1, 48.8, 34.2, 27.0, 26.1, 24.8, 14.3. HRMS (ESI) *m/z*: $[M+H]^+$ Calcd for $C_{18}H_{28}N_2O_2$ 305.2224; Found 305.2229.

Synthesis of 4-benzyl-1-methylpyrrolidin-2-one (13)

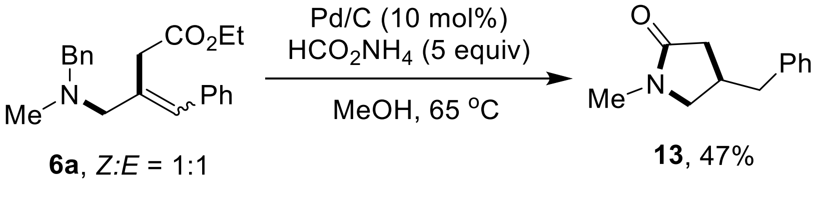

To a solution of **6a** (64.6 mg, 0.2 mmol) in MeOH (2.0 mL) was added HCO_2NH_4 (5.0 equiv). After 5 min, 10% Pd/C was added and the reaction mixture was heated at 65 °C for 2 h. The reaction mixture was filtered through a plug of Celite and washed with MeOH. The resulting filtrate was concentrated under reduced pressure. The crude mixture was purified by column chromatography on silica gel (eluent: DCM/MeOH

40:1) to give product **13** (colorless oil, 47% yield, 17.8 mg). ^{1}H NMR (400 MHz, Chloroform-*d*) δ 7.34 – 7.19 (m, 3H), 7.18 – 7.12 (m, 2H), 3.37 (dd, *J* = 9.8, 7.4 Hz, 1H), 3.09 (dd, *J* = 9.8, 5.6 Hz, 1H), 2.86 – 2.75 (m, 4H), 2.73 – 2.59 (m, 2H), 2.50 (dd, *J* = 16.7, 8.1 Hz, 1H), 2.17 (dd, *J* = 16.7, 6.3 Hz, 1H). ^{13}C NMR (101 MHz, $CDCl_3$) δ 174.2, 139.2, 128.7, 128.6, 126.5, 54.7, 40.7, 37.2, 33.1, 29.6. HRMS (ESI) *m/z*: $[M+Na]^+$ Calcd for $C_{12}H_{15}NO$ 212.1046; Found 212.1061.

7. Mechanistic experiments

(1) Radical trapping experiment with TEMPO

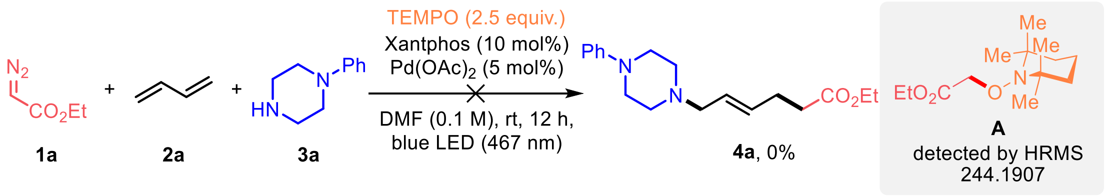

To a reaction tube equipped with a magnetic stirring bar were added $Pd(OAc)_2$ (2.2 mg, 0.01 mmol, 5.0 mol %), Xantphos (11.6 mg, 0.02 mmol, 10.0 mol %), and TEMPO (78 mg, 0.5 mmol, 2.5 equiv). The tube was capped. After evacuation and backfilling with argon three times, anhydrous DMF (2.0 mL, 0.1 M) was added via a syringe, followed by stirring at room temperature for about 5 minutes. To this reaction system were added 1,3-diene **2a** (0.4 mmol, 2.0 equiv), amine **3a** (0.2 mmol, 1.0 equiv), and diazo compound **1a** (0.3 mmol, 1.5 equiv). The reaction mixture was irradiated by blue LED at room temperature for 12 h. Product **4a** was not detected by LC–MS or ^{1}H NMR. Notably, radical trapping product **A** could be observed by HRMS.

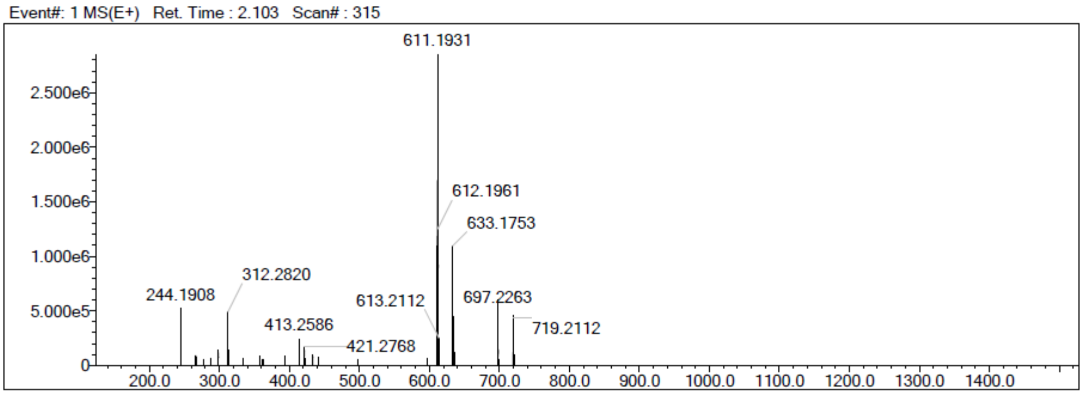

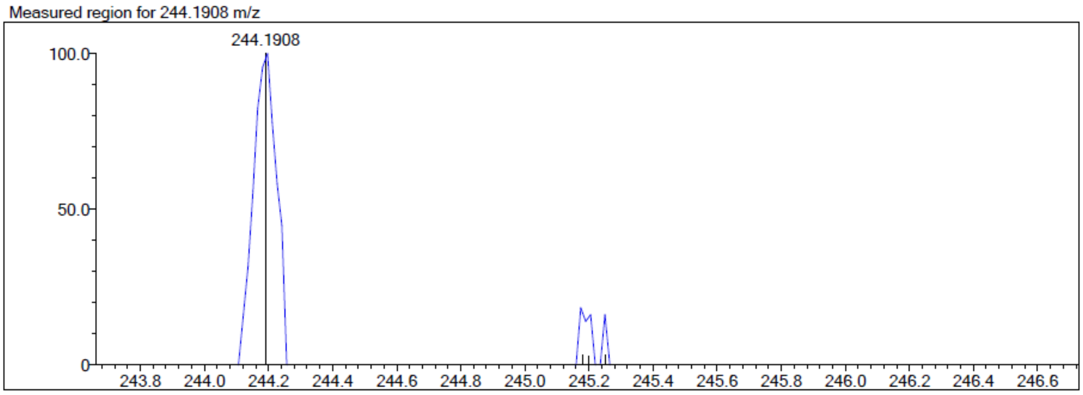

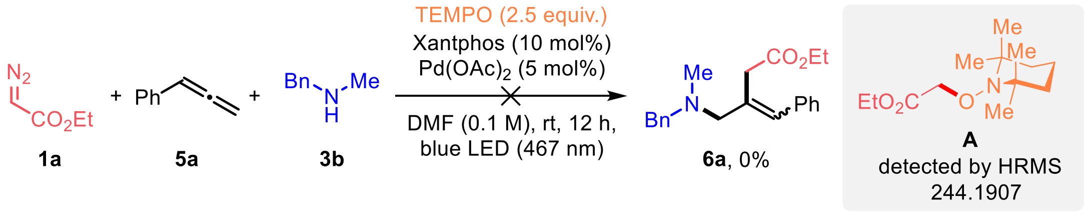

To a reaction tube equipped with a magnetic stirring bar were added $Pd(OAc)_2$ (2.2 mg, 0.01 mmol, 5.0 mol %) and Xantphos (11.6 mg, 0.02 mmol, 10.0 mol %). The tube was capped. After evacuation and backfilling with argon three times, anhydrous DMF (2.0 mL, 0.1 M) was added via a syringe, followed by stirring at room temperature for about 5 minutes. To this reaction system were added allene **5a** (0.4 mmol, 2.0 equiv), amine **3b** (0.2 mmol, 1.0 equiv), and diazo compound **1a** (0.3 mmol, 1.5 equiv). The reaction mixture was irradiated by blue LED at room temperature for 12 h. Product **6a** was not detected by LC–MS or ^{1}H NMR. Notably, radical trapping product **A** could be observed by HRMS.

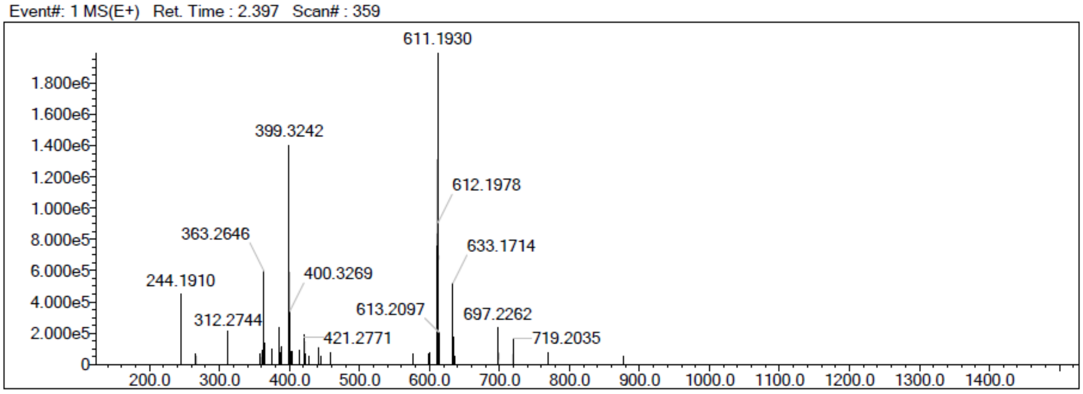

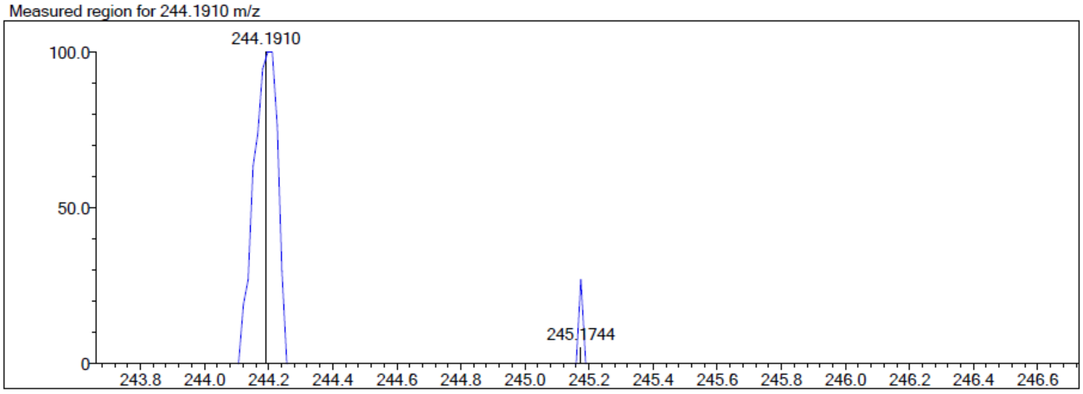

Conclusion: Using TEMPO as the radical trapping reagent, the model reaction was completely inhibited and radical trapping product **A** could be observed by HRMS, indicating the radical nature of the transformation.

(2) Isotope-labeling studies with deuterated amines

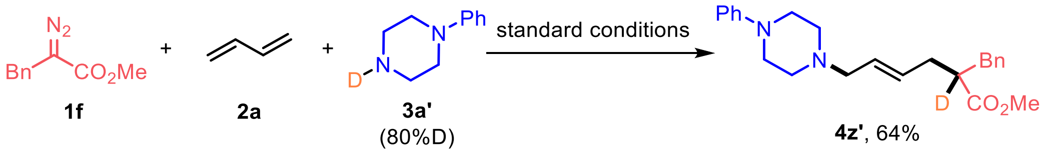

To a reaction tube equipped with a magnetic stirring bar were added $Pd(OAc)_2$ (2.2 mg, 0.01 mmol, 5.0 mol %) and Xantphos (11.6 mg, 0.02 mmol, 10.0 mol %). The tube was capped. After evacuation and backfilling with argon three times, anhydrous DMF (2.0 mL, 0.1 M) was added via a syringe, followed by stirring at room temperature for about 5 minutes. To this reaction system were added 1,3-diene **2a** (0.4 mmol, 2.0 equiv), deuterated amine **3a′** (0.2 mmol, 1 equiv, 80% deuterated ratio), and diazo compound **1f** (0.3 mmol, 1.5 equiv). The reaction mixture was irradiated by blue LED at room temperature for 12 h until the diazo compound was consumed completely, as monitored by TLC analysis. The crude mixture was purified by column chromatography on silica gel with DCM/MeOH mixtures as eluent to give the deuterated products **4z′** in 64% yield (36% deuterated ratio).

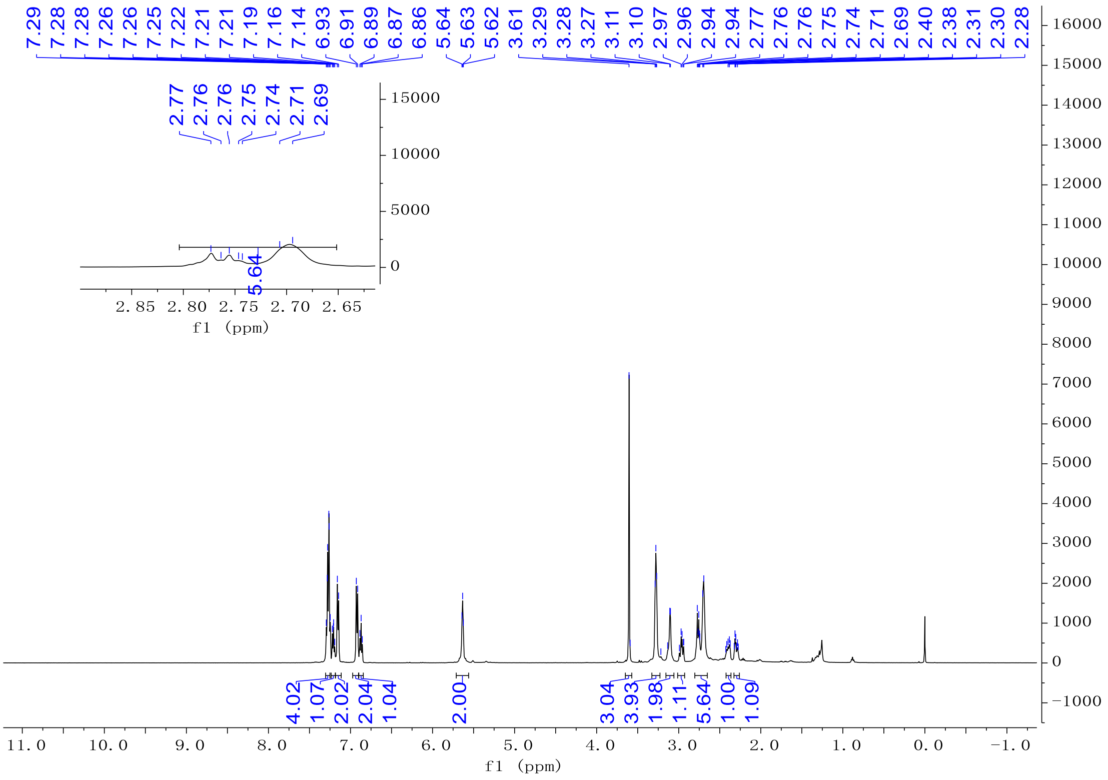

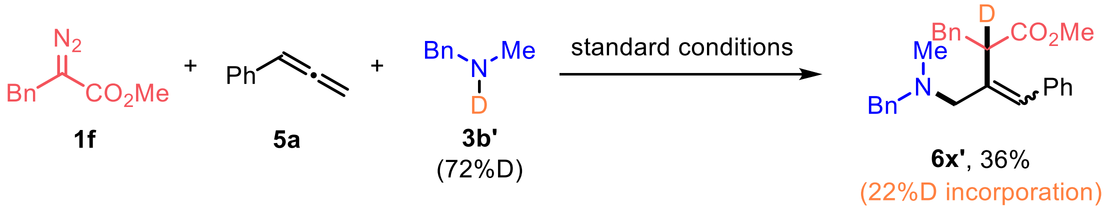

To a reaction tube equipped with a magnetic stirring bar were added $Pd(OAc)_2$ (2.2 mg, 0.01 mmol, 5.0 mol %) and Xantphos (11.6 mg, 0.02 mmol, 10.0 mol %). The tube was capped. After evacuation and backfilling with argon three times, anhydrous DMF (2.0 mL, 0.1 M) was added via a syringe, followed by stirring at room temperature for about 5 minutes. To this reaction system were added allene **5a** (0.4 mmol, 2.0 equiv), deuterated amine **3b′** (0.2 mmol, 1.0 equiv, 72% deuterated ratio), and diazo compound **1f** (0.3 mmol, 1.5 equiv). The reaction mixture was irradiated by blue LED at room temperature for 12 h until the diazo compound was consumed completely, as monitored by TLC analysis. The crude mixture was purified by column chromatography on silica gel with DCM/MeOH mixtures as eluent to give the corresponding product **6x′** in 36% yield (22% deuterated ratio).

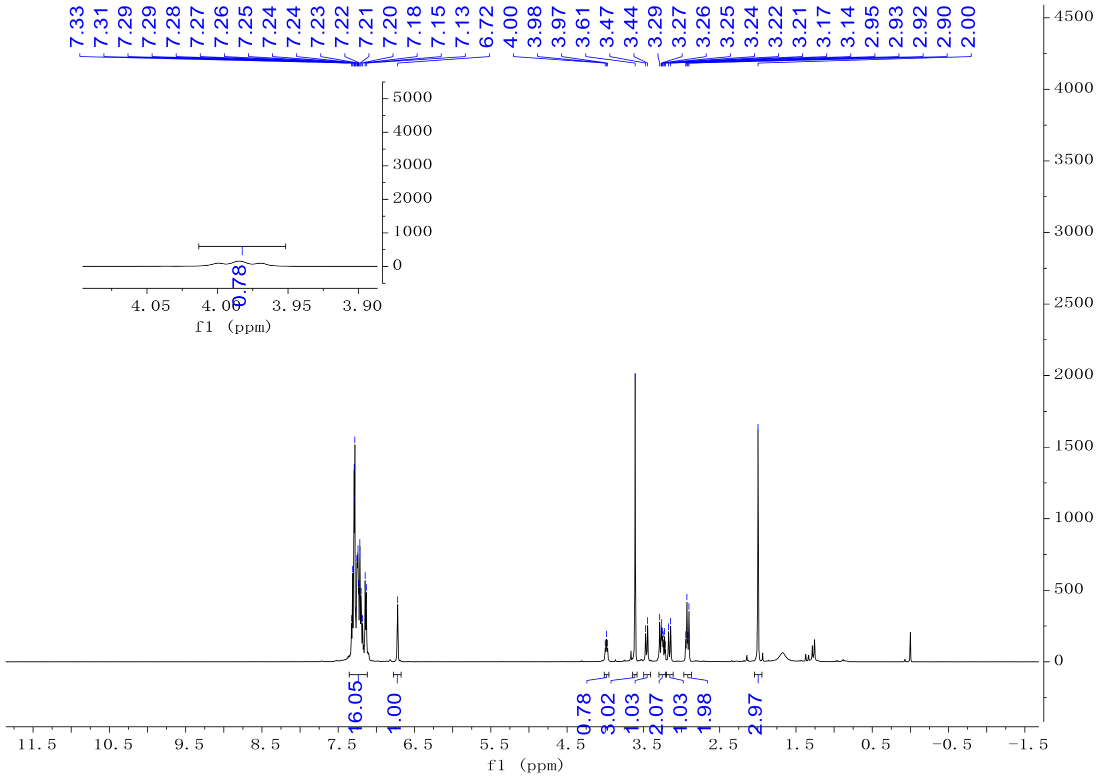

(3) Isotope-labeling studies with deuterium oxide

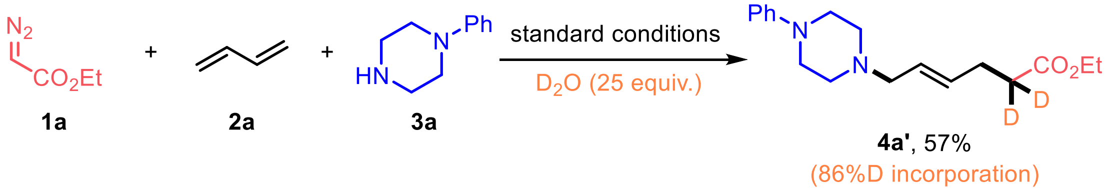

To a reaction tube equipped with a magnetic stirring bar were added $Pd(OAc)_2$ (2.2 mg, 0.01 mmol, 5.0 mol %) and Xantphos (11.6 mg, 0.02 mmol, 10.0 mol %). The tube was capped. After evacuation and backfilling with argon three times, anhydrous DMF (2.0 mL, 0.1 M) was added via a syringe, followed by stirring at room temperature for about 5 minutes. To this reaction system were added D_2O (5.0 mmol, 25.0 equiv), 1,3-diene **2a** (0.4 mmol, 2.0 equiv), deuterated amine **3a** (0.2 mmol, 1.0 equiv, 80% deuterated ratio), and diazo compound **1a** (0.3 mmol, 1.5 equiv). The reaction mixture was irradiated by blue LED at room temperature for 12 h until the diazo compound was consumed completely, as monitored by TLC analysis. The crude mixture was purified by column chromatography on silica gel with DCM/MeOH mixtures as eluent to give the deuterated products **4a′** in 57 % yield (86% deuterated ratio).

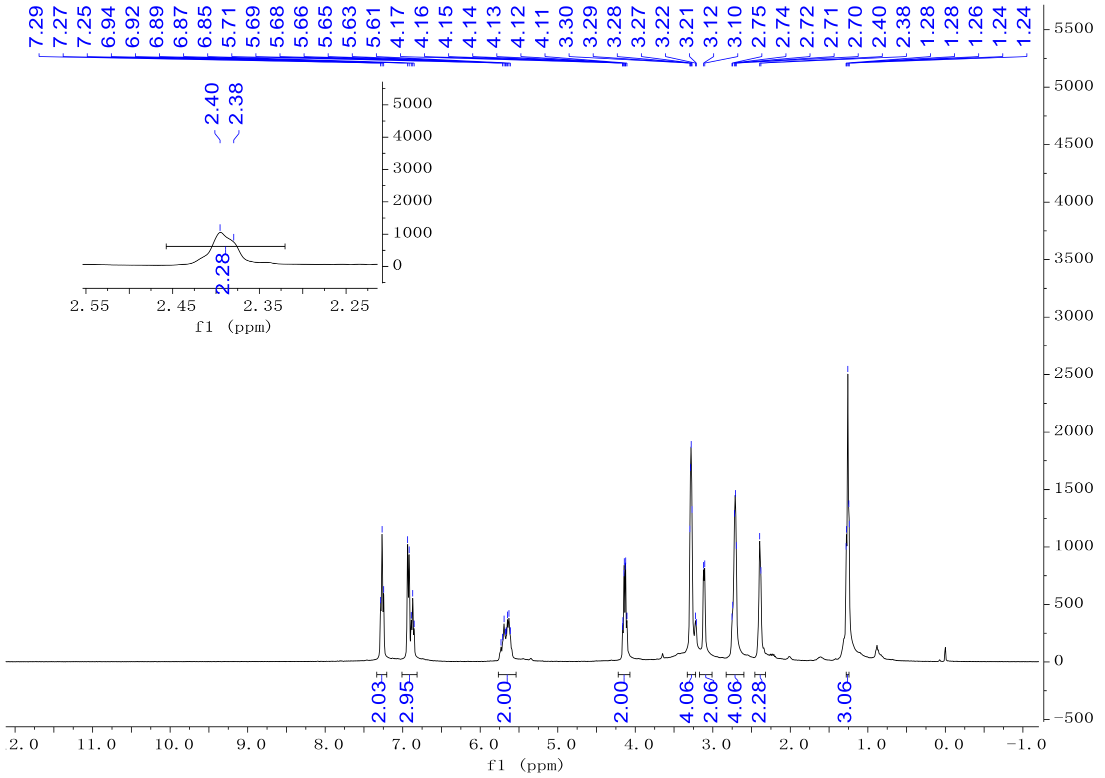

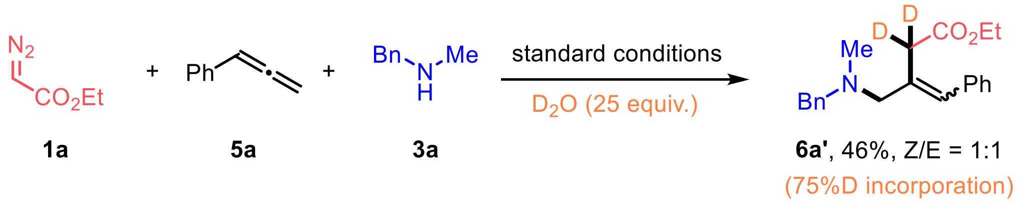

To a reaction tube equipped with a magnetic stirring bar were added $Pd(OAc)_2$ (2.2 mg, 0.01 mmol, 5.0 mol %) and Xantphos (11.6 mg, 0.02 mmol, 10.0 mol %). The tube was capped. After evacuation and backfilling with argon three times, anhydrous DMF (2.0 mL, 0.1 M) was added via a syringe, followed by stirring at room temperature for about 5 minutes. To this reaction system were added D_2O (5.0 mmol, 25.0 equiv), allene **5a** (0.4 mmol, 2.0 equiv), amine **3a** (0.2 mmol, 1.0 equiv), and diazo compound **1a** (0.3 mmol, 1.5 equiv). The reaction mixture was irradiated by blue LED at room temperature for 12 h until the diazo compound was consumed completely, as monitored by TLC analysis. The crude mixture was purified by column chromatography on silica gel with DCM/MeOH mixtures as eluent to give the corresponding product **6a′** in 46% yield (75% deuterated ratio).

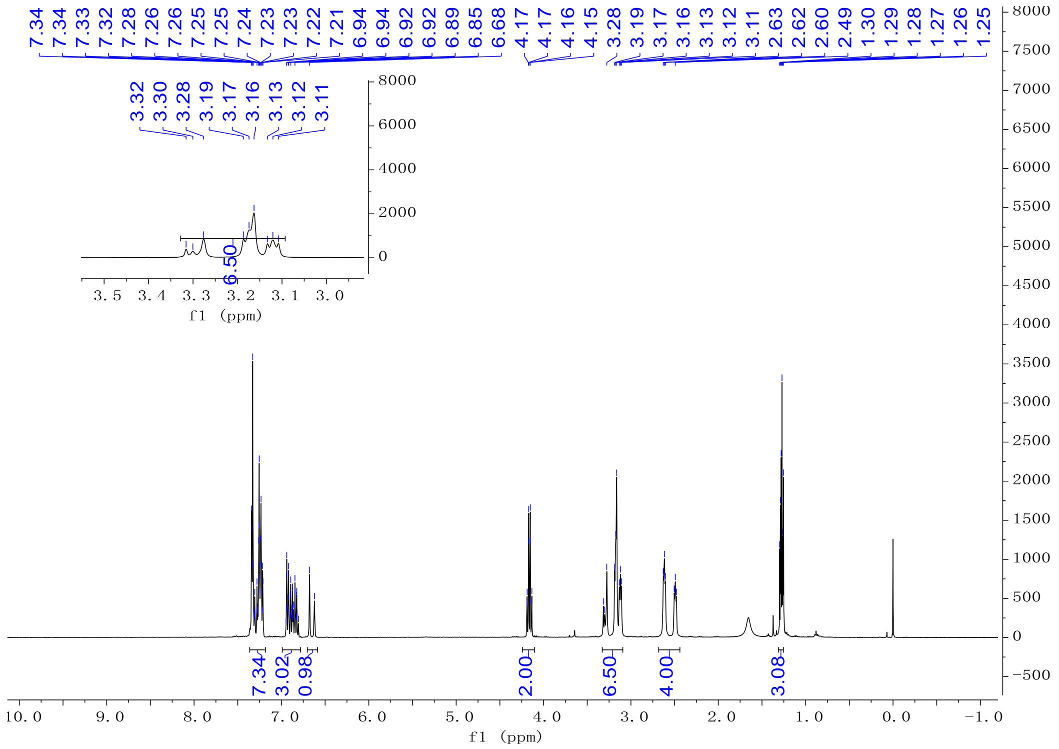

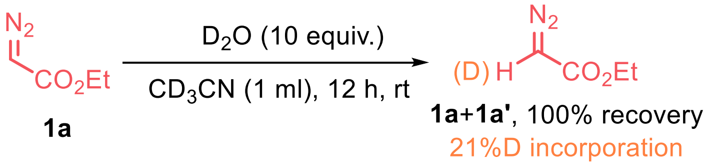

To a reaction tube equipped with a magnetic stirring bar were added EDA **1a** (0.2 mmol) and D_2O (10 equiv). The tube was capped. After evacuation and backfilling with argon three times, CD_3CN (1 mL) was added via a syringe, followed by stirring at room temperature for 12 h. The crude mixture was detected by ^1H NMR (**1a** + **1a′**, 100% recovery, 21% deuterated ratio).

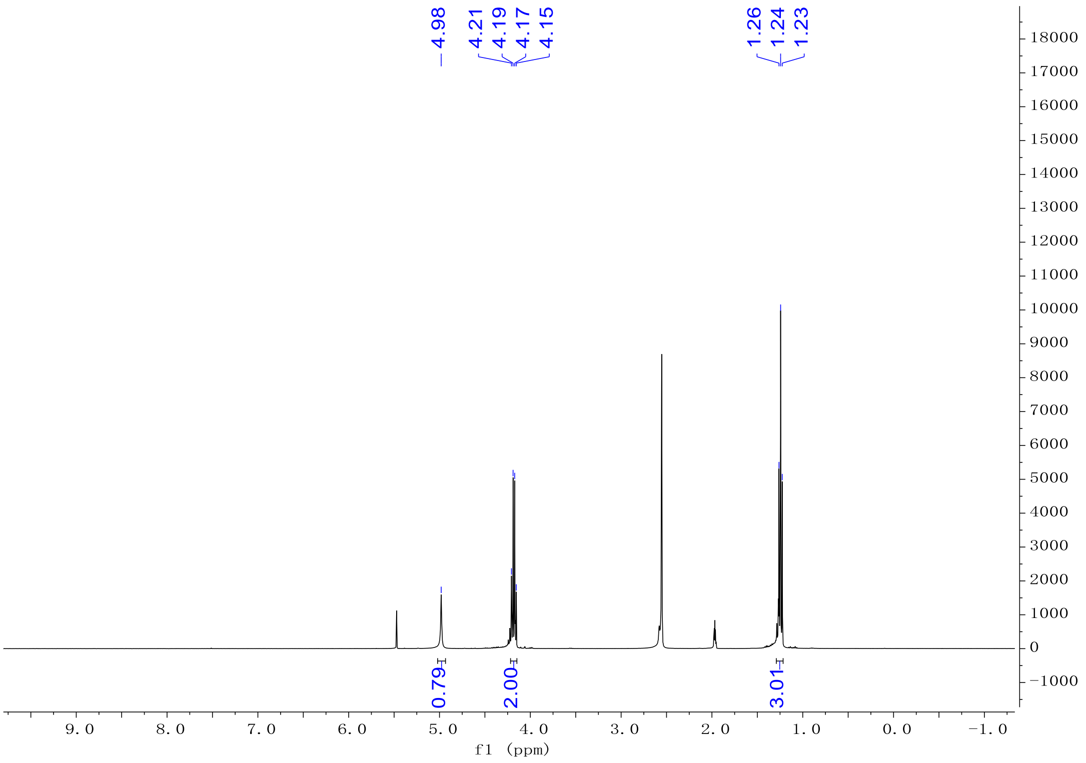

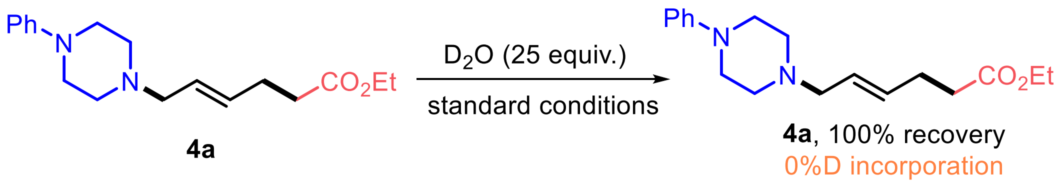

To a reaction tube equipped with a magnetic stirring bar were added $Pd(OAc)_2$ (2.2 mg, 0.01 mmol, 5.0 mol %) and Xantphos (11.6 mg, 0.02 mmol, 10.0 mol %). The tube was capped. After evacuation and backfilling with argon three times, anhydrous DMF (2.0 mL, 0.1 M) was added via a syringe, followed by stirring at room temperature for about 5 minutes. To this reaction system were added D_2O (5.0 mmol, 25.0 equiv), product **4a**

(0.2 mmol, 1.0 equiv). The reaction mixture was irradiated by blue LED at room temperature for 12 h. The crude mixture was purified by column chromatography on silica gel with DCM/MeOH mixtures as eluent to give the corresponding product **4a** in 100% recovery yield (0% deuterated ratio).

(4) Subjecting product *Z*-6i to the standard conditions

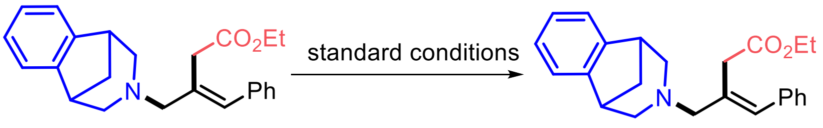

To a reaction tube equipped with a magnetic stirring bar were added $Pd(OAc)_2$ (2.2 mg, 0.01 mmol, 5.0 mol %) and Xantphos (11.6 mg, 0.02 mmol, 10.0 mol %). The tube was capped. After evacuation and backfilling with argon three times, anhydrous DMF (2.0 mL, 0.1 M) was added via a syringe, followed by stirring at room temperature for about 5 minutes. To this reaction system were added product *Z*-**6a** (0.2 mmol, 1.0 equiv). The reaction mixture was irradiated by blue LED at room temperature for 12 h. The crude mixture was purified by column chromatography on silica gel with DCM/MeOH mixtures as eluent to give the corresponding product *Z*-**6a** in 100% recovery yield. *Z*-**6i**: ^{1}H NMR of (400 MHz, Chloroform-*d*) δ 7.29 – 7.26 (m, 1H), 7.25 – 7.16 (m, 2H), 7.15 – 7.08 (m, 6H), 6.39 (s, 1H), 3.98 (q, *J* = 7.1 Hz, 2H), 3.17 (s, 2H), 3.05 (t, *J* = 4.2 Hz, 2H), 2.75 – 2.56 (m, 4H), 2.31 (d, *J* = 10.0 Hz, 2H), 2.26 – 2.20 (m, 1H), 1.62 (s, 1H), 1.20 (t, *J* = 7.1 Hz, 3H). ^{13}C NMR (101 MHz, $CDCl_3$) δ 172.0, 146.2, 137.1, 134.9, 130.8, 129.1, 127.9, 126.5, 126.2, 121.3, 60.1, 56.3, 55.6, 43.6, 41.2, 40.1, 14.2.

Conclusion: The products are not selectively formed and then isomerized.

(5) Interrupted radical Heck/Tsuji–Trost studies with activated halides using Gevorgyan's[15] and Glorius'[16] conditions

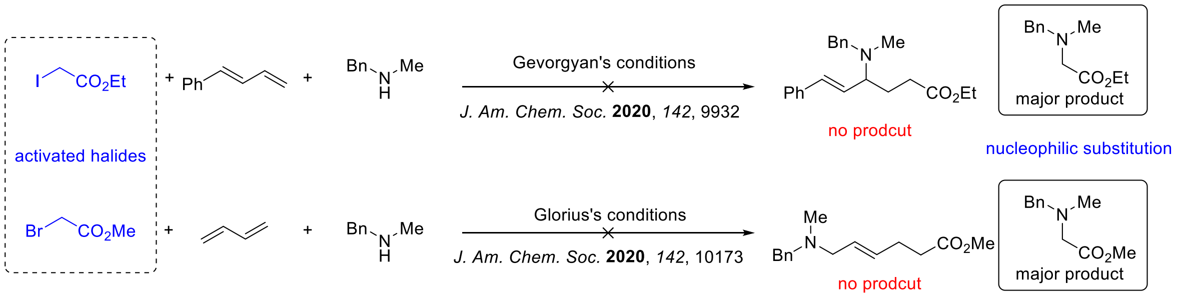

To determine whether activated olefin could be involved in the interrupted radical Heck/Tsuji-Trost reactions reported by Gevorgyan and Glorius, we conducted the above two experiments. The results showed that the target products could not be obtained at all, and the main by-products were the nucleophilic substitution products of activated halides and amines.

Conclusion: Palladium-catalyzed interrupted radical Heck/Tsuji-Trost reactions with activated alkyl halides remains a challenge owing to the direct nucleophilic substitution of activated alkyl halides with nucleophilic reagents under alkaline conditions.

8. UV–visible absorption analysis

UV–vis absorption spectra were recorded using a SHIMADZU UV-2600 ultraviolet-visible spectrophotometer. The UV–vis absorption of Xantphos, $Pd(OAc)_2$ + Xantphos, allene **5a**, diene **2f**, amine **3b**, and diazo **1a** were measured in DMF as solvent.

Conclusion: According to the UV–vis spectra, the only absorbing species at 467 nm consists in the pre-catalytic system $Pd(OAc)_2$ and Xantphos.

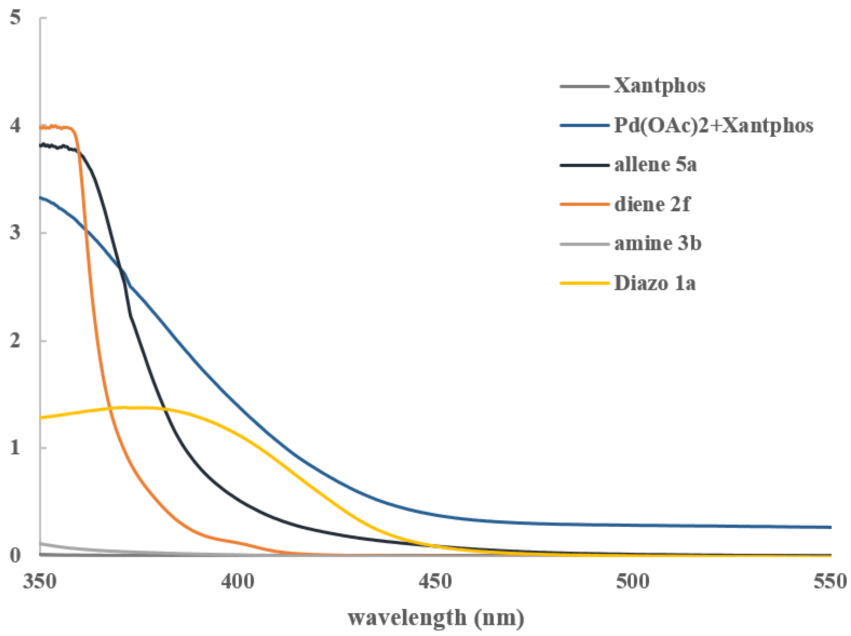

9. References

1. Sun, Y. & Zhang, G. Palladium-Catalyzed Formal [4+2] Cycloaddition of Benzoic and Acrylic Acids with 1,3-Dienes via C-H Bond Activation: Efficient Access to 3,4-Dihydroisocoumarin and 5,6-Dihydrocoumalins. *Chin. J. Chem*. **36,** 708–711 (2018).
2. Bhowmik, A. & Fernandes, R. A. Iron(III)/O2-Mediated Regioselective Oxidative Cleavage of 1-Arylbutadienes to Cinnamaldehydes. *Org. Lett.* **21***,* 9203−9207 (2019).
3. Gao, W. et al. One-Pot and Unsymmetrical Bis-Allylation of Malononitrile with Conjugated Dienes and Allylic Alcohols. *Org. Lett.* **24***,* 9355–9360 (2022).
4. Xu, L. et al. Iridium-catalyzed alkenyl C–H allylation using conjugated dienes. *Chem. Commun.,* **55***,* 9757 (2019).
5. Yu, S., Noble, A., Bedford, R. B. & Aggarwal, V. K. Methylenespiro[2.3]hexanes via Nickel-Catalyzed Cyclopropanations with [1.1.1]Propellane. *J. Am. Chem. Soc.* **141***,* 20325–20334 (2019).
6. Zhu, S., Guo, Z., Huang, Z. & Jiang, H. Bioinspired Intramolecular Diels–Alder Reaction: A Rapid Access to the Highly-Strained Cyclopropane-Fused Polycyclic Skeleton. *Chem. Eur. J*. **20**, 2425–2430 (2014).
7. Kliman, L. T., Mlynarsk, S. N. & Ferris, G. E. Catalytic Enantioselective 1,2-Diboration of 1,3-Dienes: Versatile Reagents for Stereoselective Allylation. *Angew. Chem. Int. Ed*. **51**, 521–524 (2011).
8. Zhao, Z., Racicot, L. & Murphy, G. K. Fluorinative Rearrangements of Substituted Phenylallenes Mediated by (Difluoroiodo)toluene: Synthesis of α-(Difluoromethyl)styrenes. *Angew. Chem. Int. Ed.* **56**, 11620 –11623 (2017).
9. Kippo, T., Fukuyama, T. & Ryu, I. Regioselective Radical Bromoallylation of Allenes Leading to 2-Bromo-Substituted 1,5-Dienes. *Org. Lett.* **15**, 3864-3867 (2011).
10. Hari, D. P. & Waser, J. Enantioselective Copper-Catalyzed Oxy-Alkynylation of Diazo Compounds. *J. Am. Chem. Soc.* **139**, 8420 – 8423 (2017).
11. Pisella, G., Gagnebin, A. & Waser, J. Copper-Catalyzed Oxyvinylation of Diazo Compounds. *Org. Lett*. **22**, 3884 – 3889 (2020).

12. Huang, L. & Wulff, W. D. Catalytic Asymmetric Synthesis of Trisubstituted Aziridines. *J. Am. Chem. Soc.* **133***,* 8892–8895 (2011).

13. Kumar, N., Venkatesh, R. & Kandasamy, J. Synthesis of functionalized S-benzyl dithiocarbamates from diazo-compounds via multi-component reactions with carbon disulfide and secondary amines. *Org. Biomol. Chem.,* **20***,* 6766-6770 (2022).

14. Dai, X., Mao, R., Guan, B., Xu, X. & Li, X. Visible light photoredox catalysis: regioselective radical addition of aminoalkyl radicals to 2,3-allenoates. *RSC Adv.*, **5**, 55290-55294 (2015).

15. Cheung, K. P. S., Kurandina, D., Yata, T. & Gevorgyan, V. Photoinduced Palladium-Catalyzed Carbofunctionalization of Conjugated Dienes Proceeding via Radical-Polar Crossover Scenario: 1,2-Aminoalkylation and Beyond. *J. Am. Chem. Soc.* **142**, 9932-9937 (2020).

16. Huang, H.-M., Bellotti, P., Pflüger, P. M., Schwarz, J. L., Heidrich, B. & Glorius, F. Three-Component, Interrupted Radical Heck/Allylic Substitution Cascade Involving Unactivated Alkyl Bromides. *J. Am. Chem. Soc.* **142**, 10173-10183 (2020).

10. NMR Spectra of carboamination products

^{1}H NMR (400 MHz, $CDCl_3$) and ^{13}C NMR (126 MHz, $CDCl_3$) spectra for product 4a

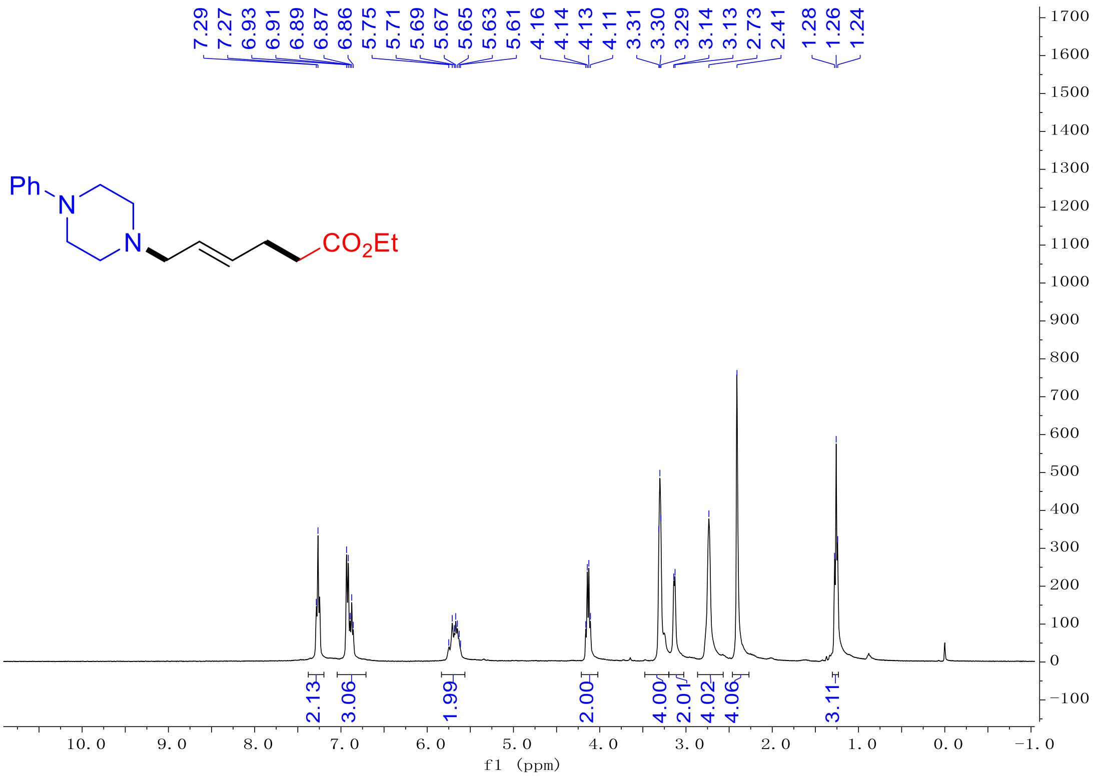

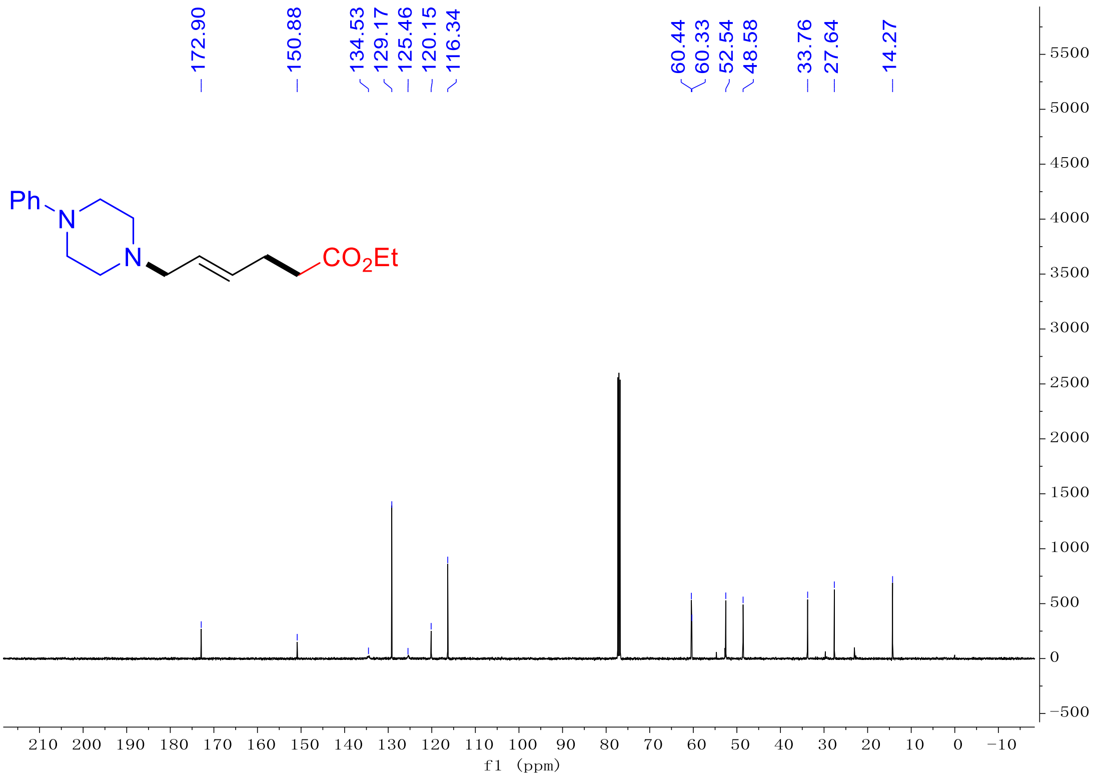

^{1}H NMR (400 MHz, $CDCl_3$) and ^{13}C NMR (101 MHz, $CDCl_3$) spectra for product 4b

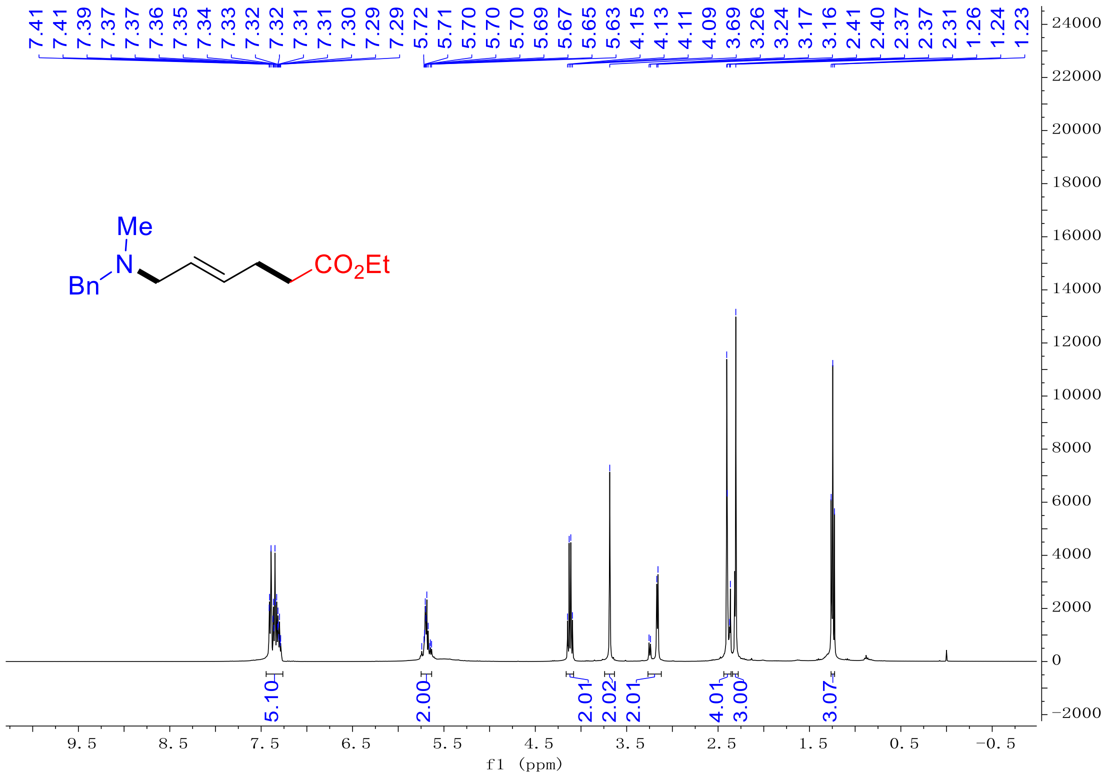

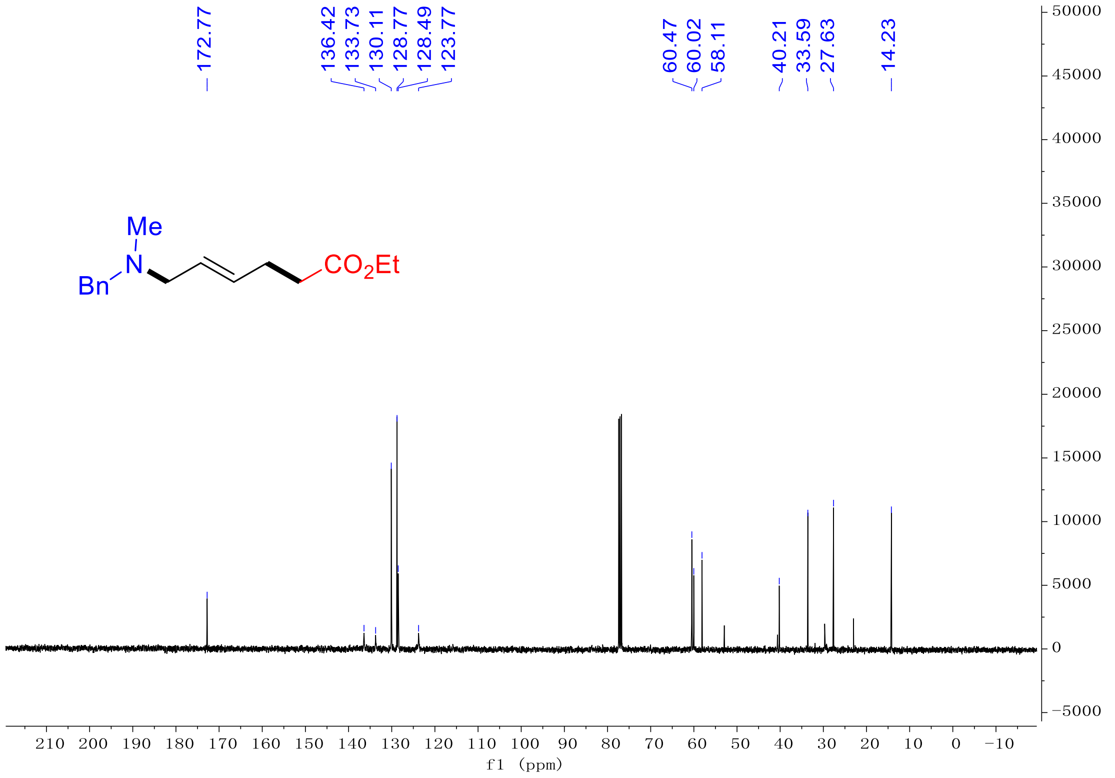

^{1}H NMR (400 MHz, CDCl3) and ^{13}C NMR (101 MHz, CDCl3) spectra for product 4c

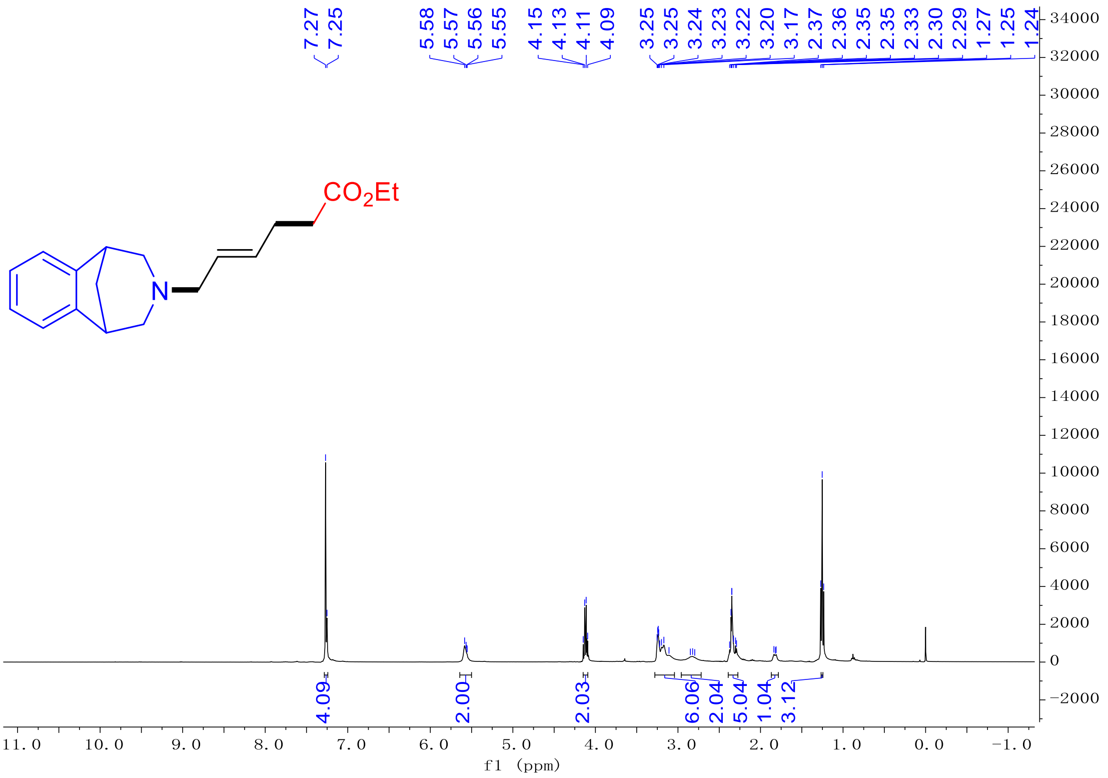

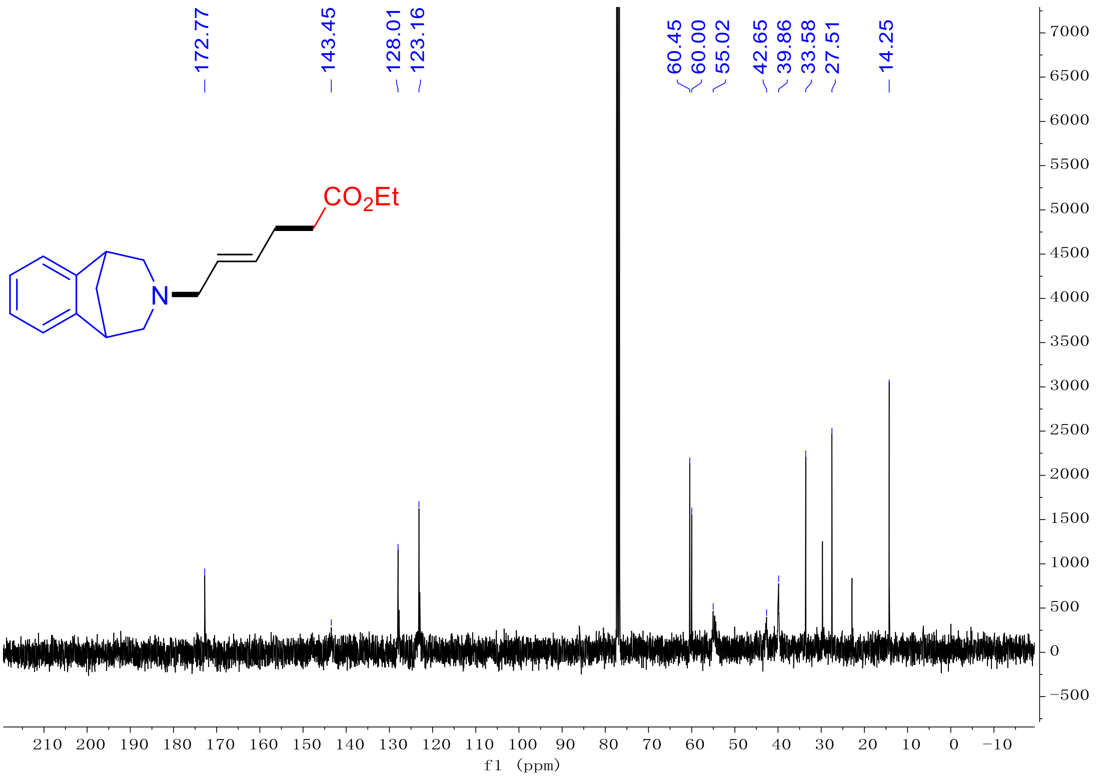

^{1}H NMR (400 MHz, $CDCl_3$) and ^{13}C NMR (101 MHz, $CDCl_3$) spectra for product 4d

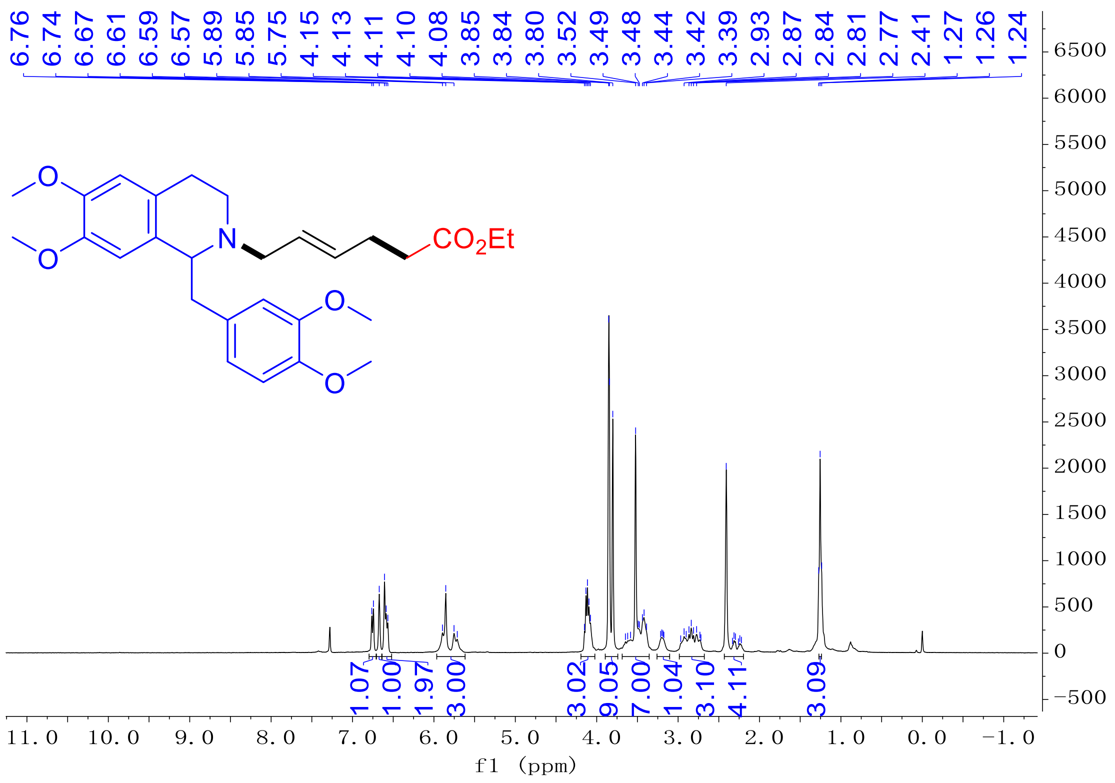

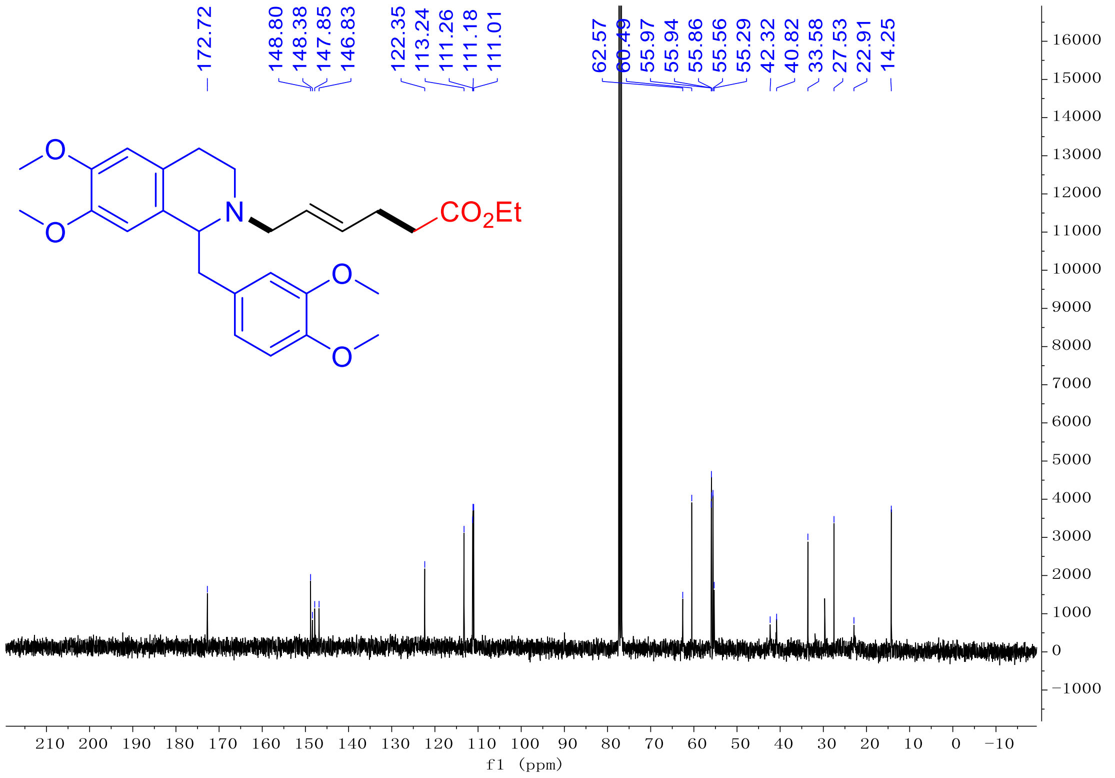

^{1}H NMR (400 MHz, $CDCl_3$) and ^{13}C NMR (126 MHz, $CDCl_3$) spectra for product 4e

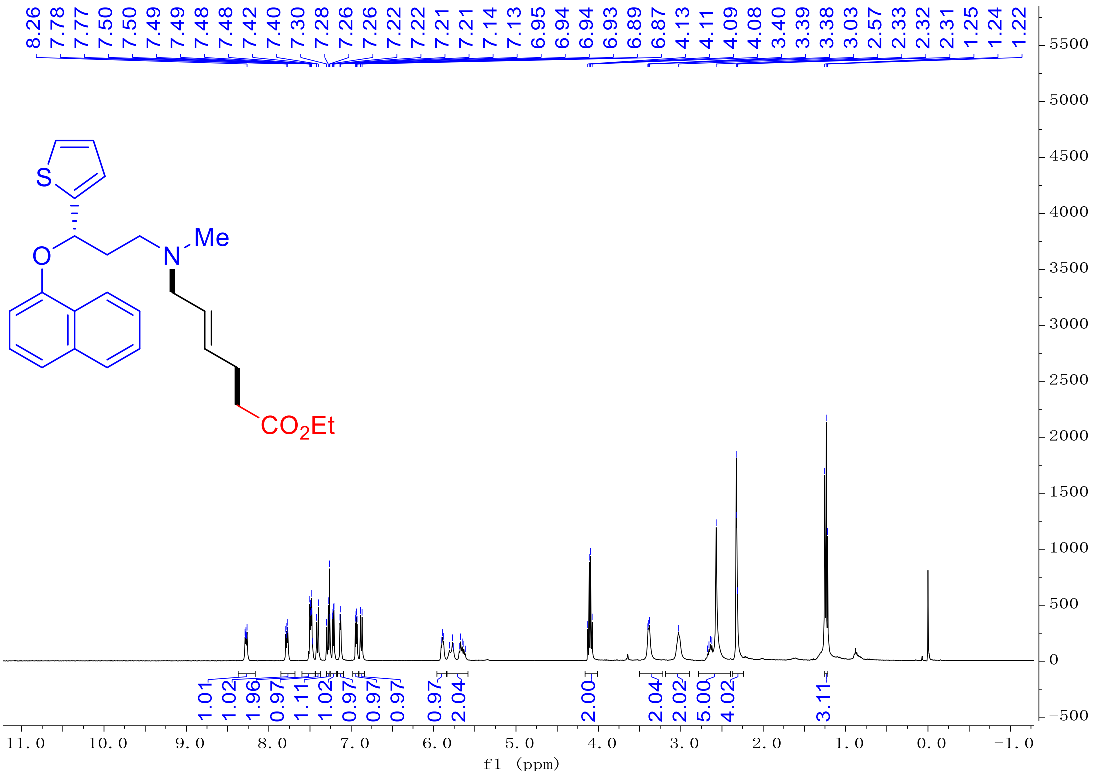

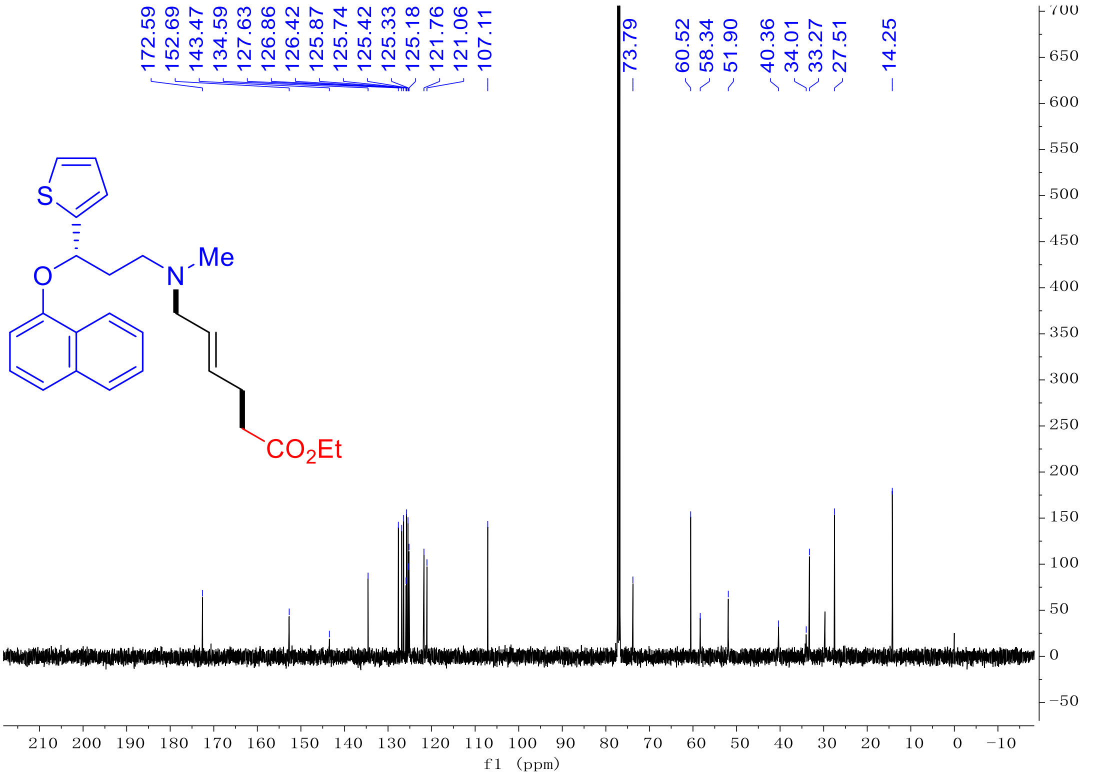

^{1}H NMR (400 MHz, CDCl3) and ^{13}C NMR (101 MHz, CDCl3) spectra for product 4f

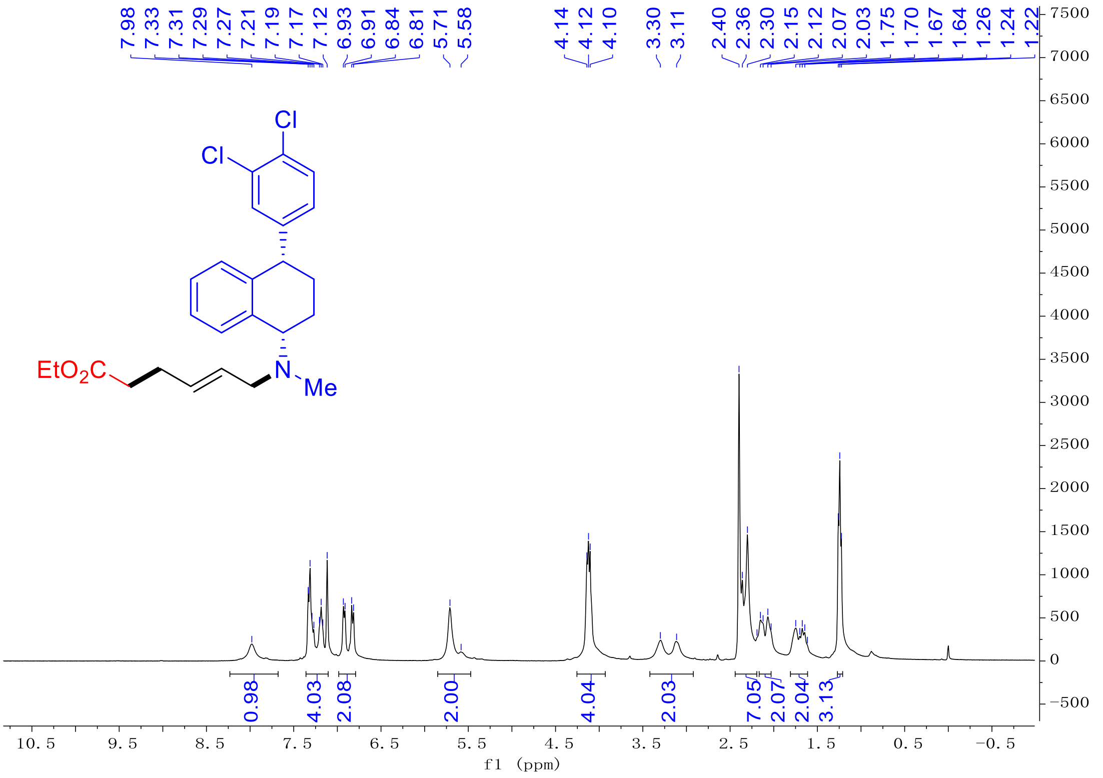

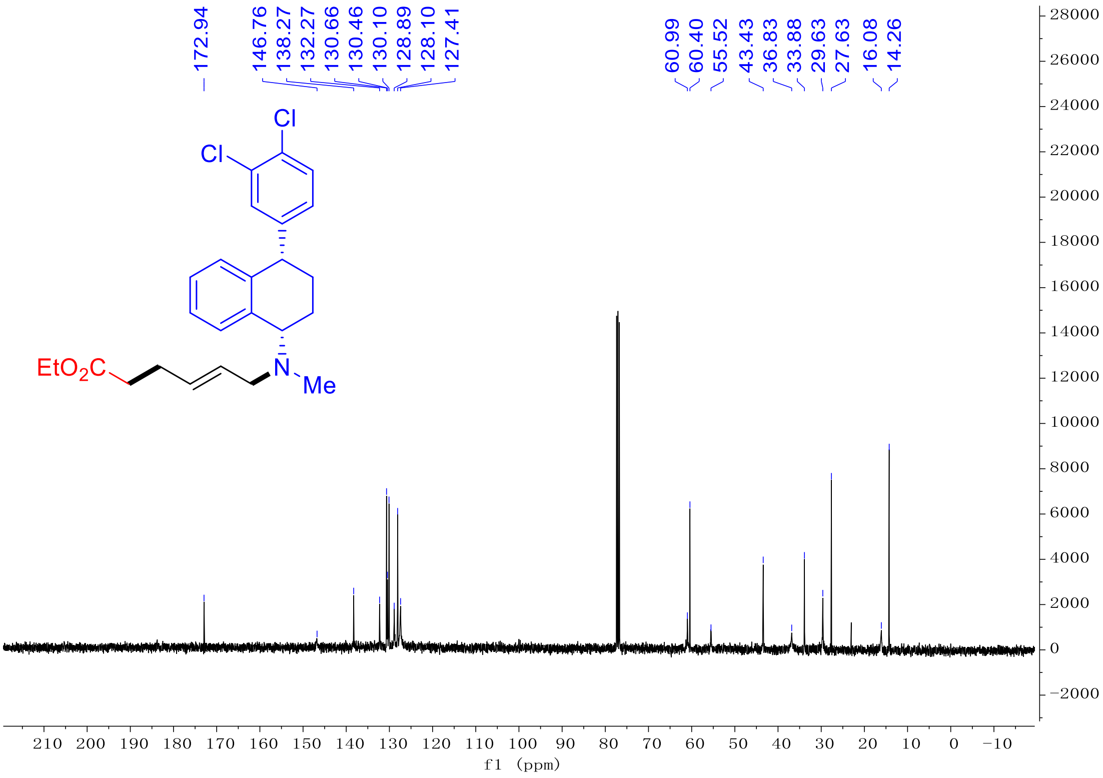

^{1}H NMR (400 MHz, $CDCl_3$) and ^{13}C NMR (126 MHz, $CDCl_3$) spectra for product 4g

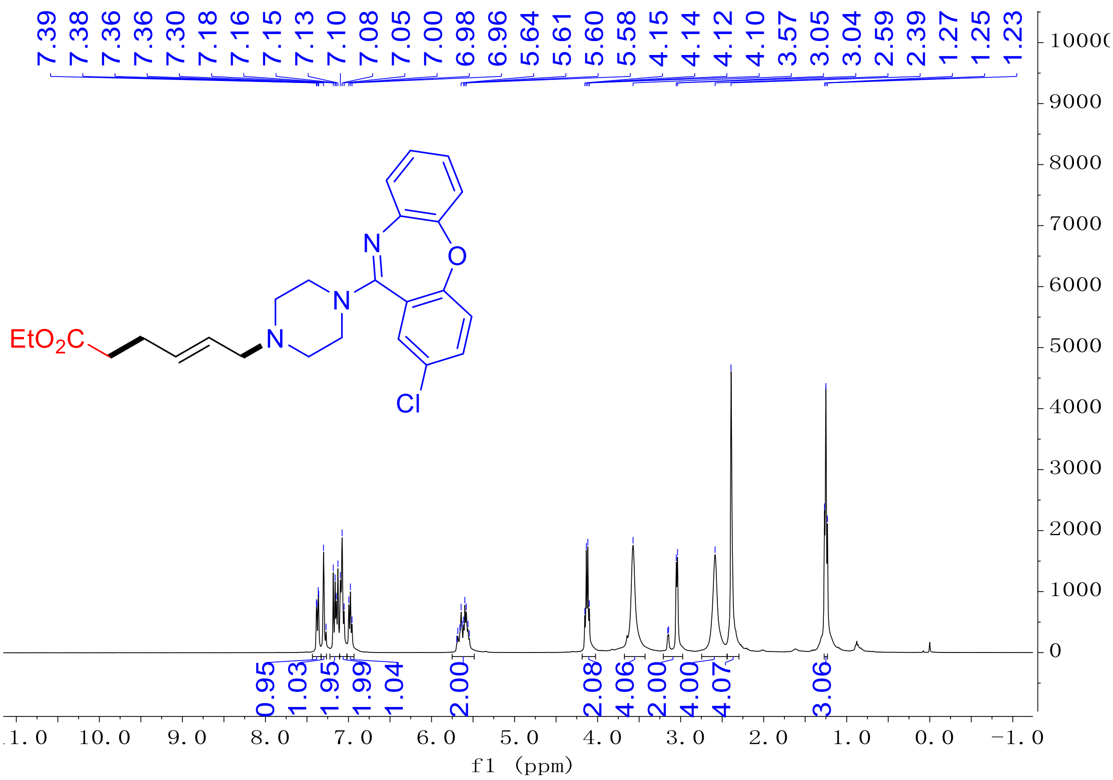

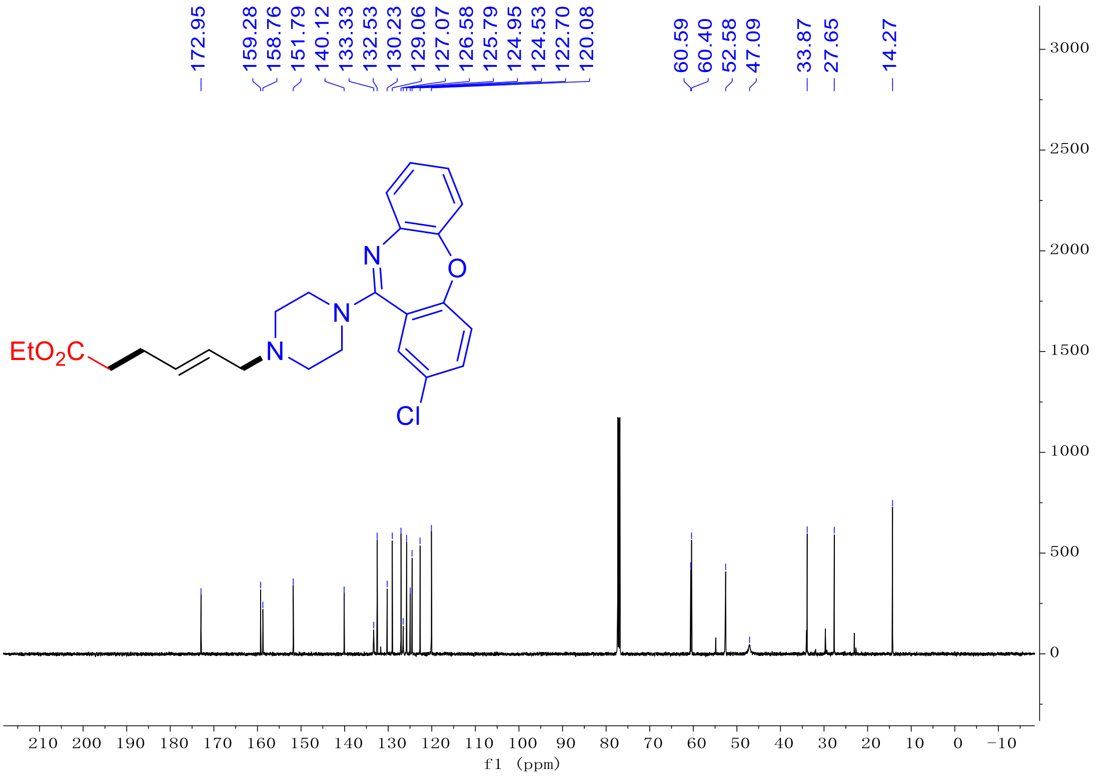

^{1}H NMR (400 MHz, CDCl3) and ^{13}C NMR (101 MHz, CDCl3) spectra for product 4h

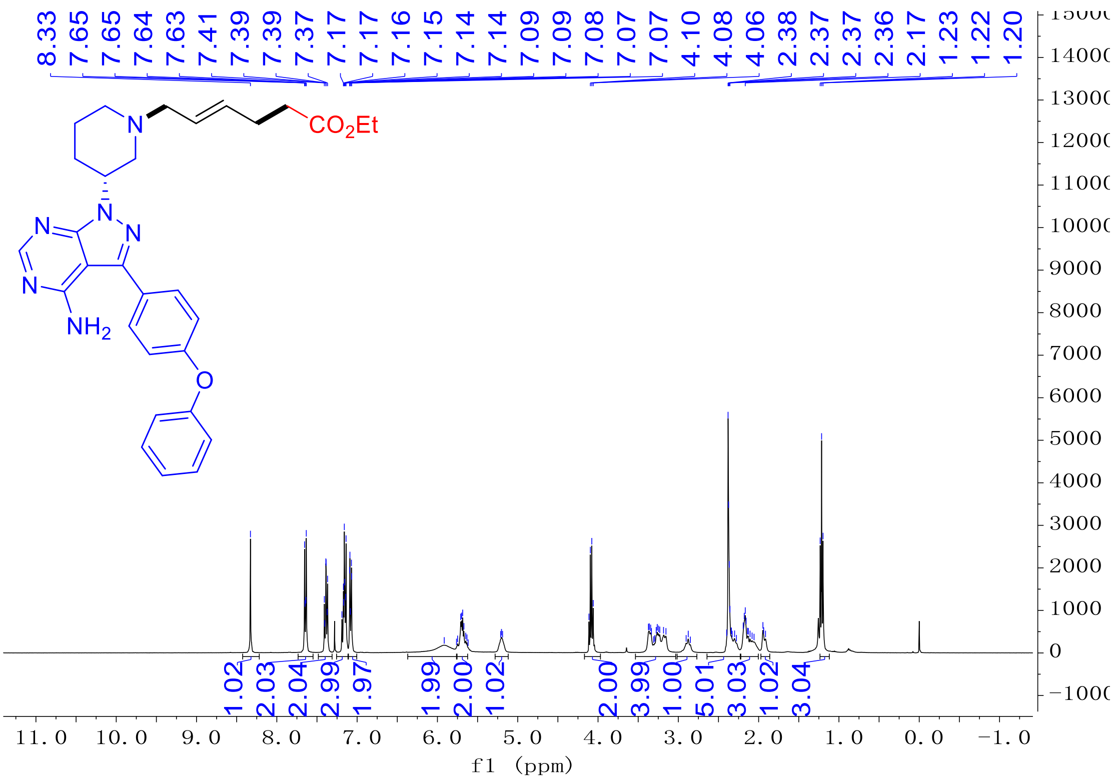

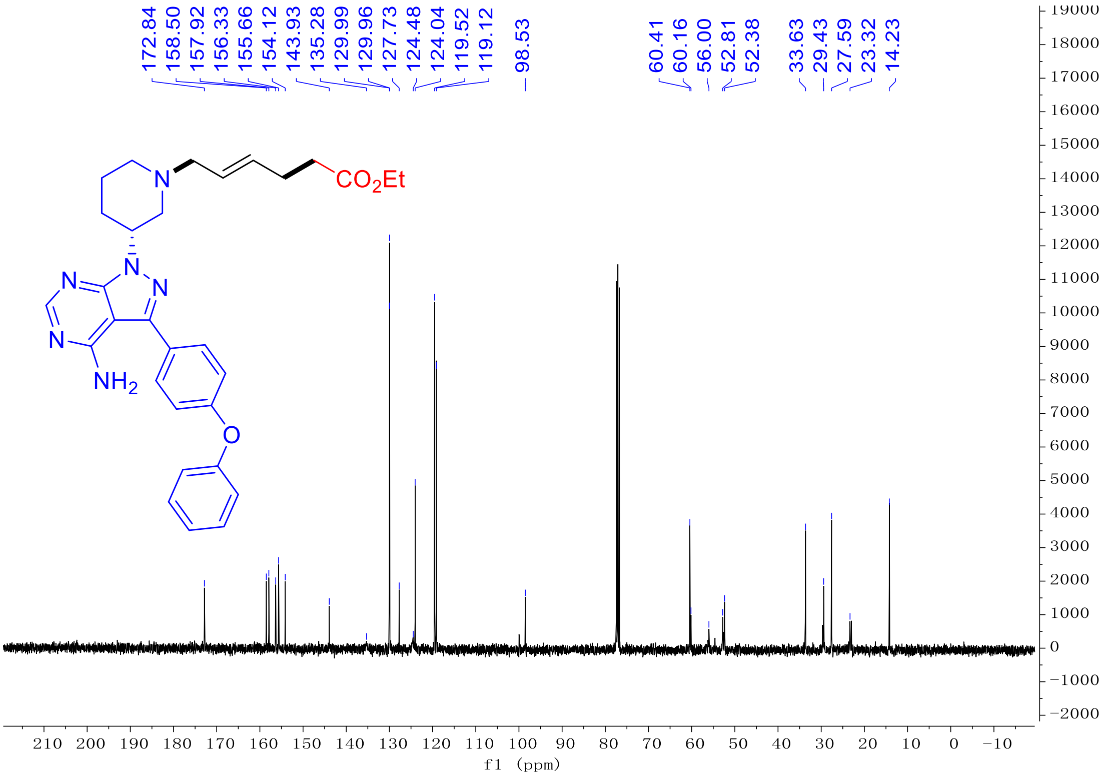

^{1}H NMR (400 MHz, $CDCl_3$) and ^{13}C NMR (126 MHz, $CDCl_3$) spectra for product 4i

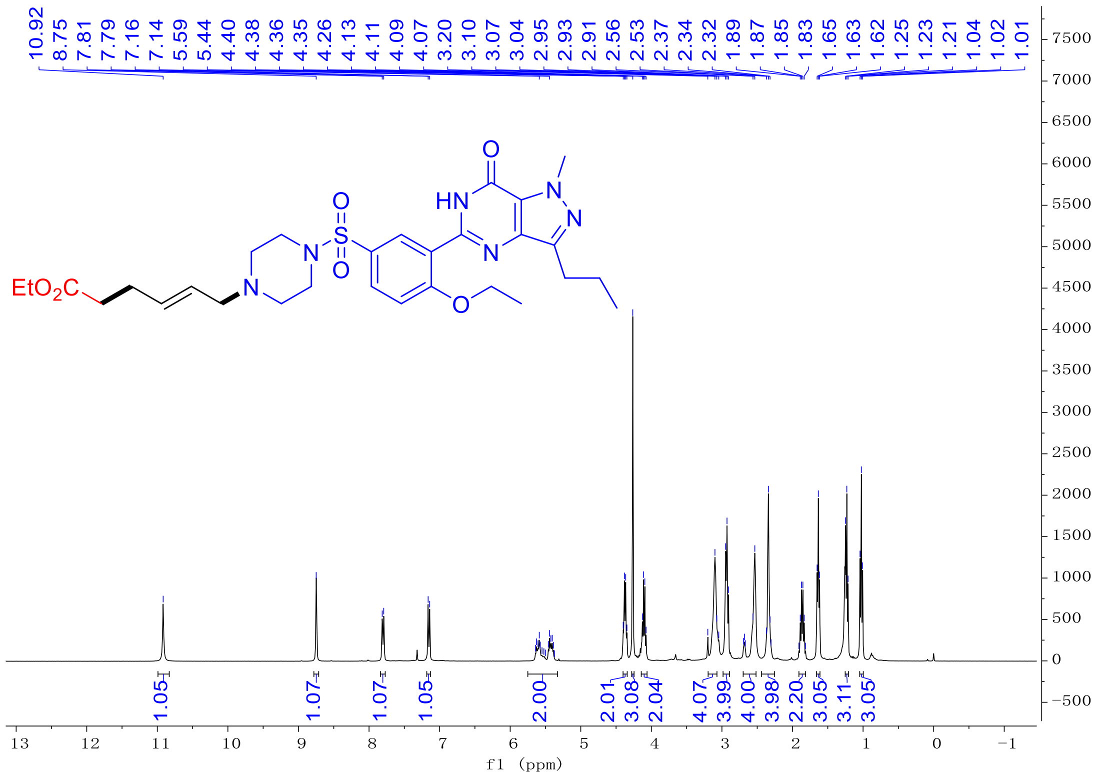

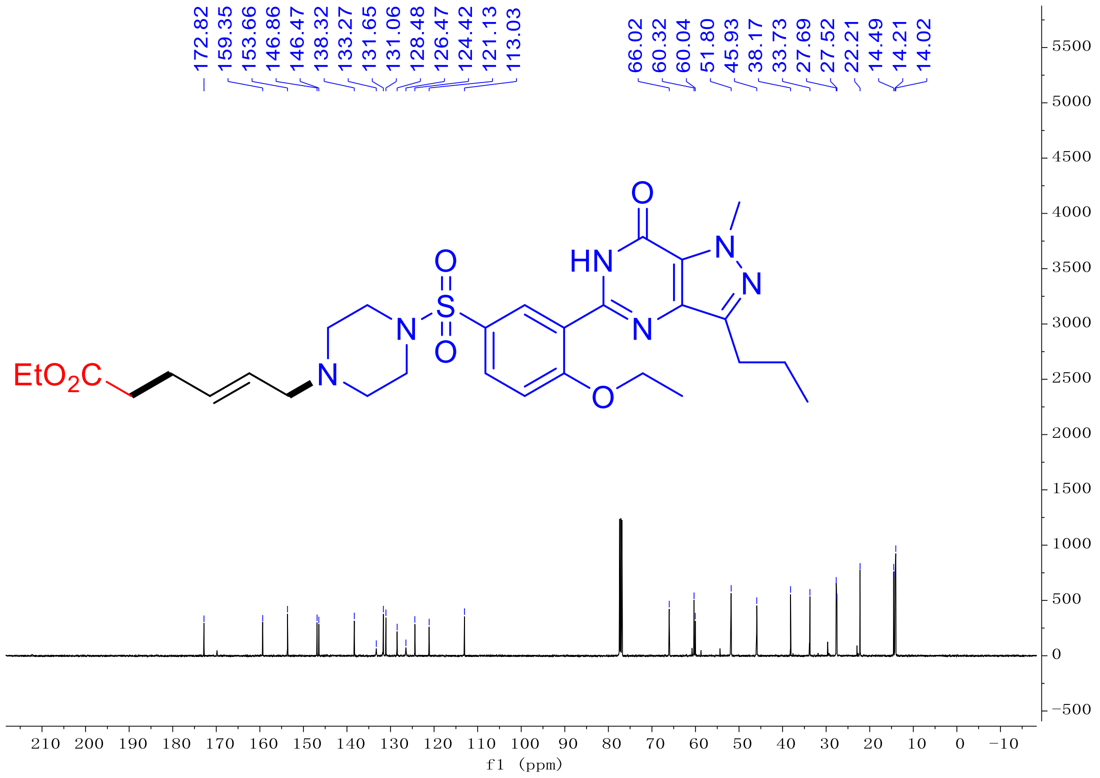

^{1}H NMR (400 MHz, CDCl$_3$) , ^{13}C NMR (101 MHz, CDCl$_3$) and ^{19}F NMR (376 MHz, CDCl$_3$) spectra for product 4j

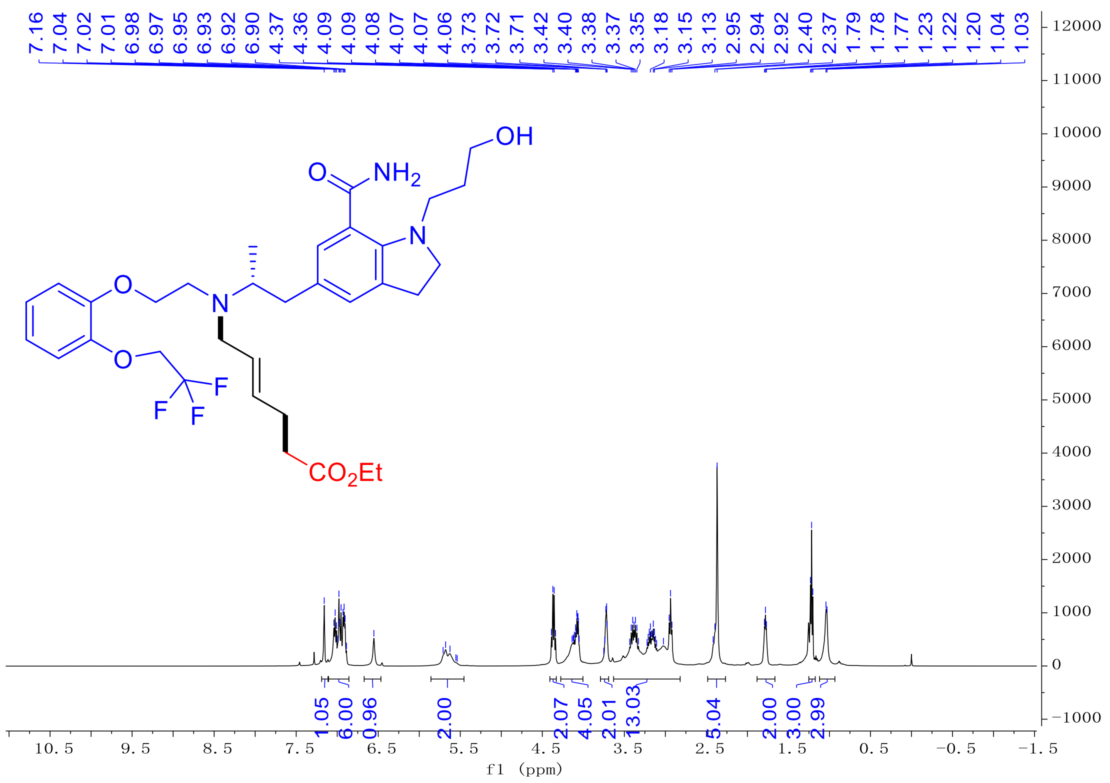

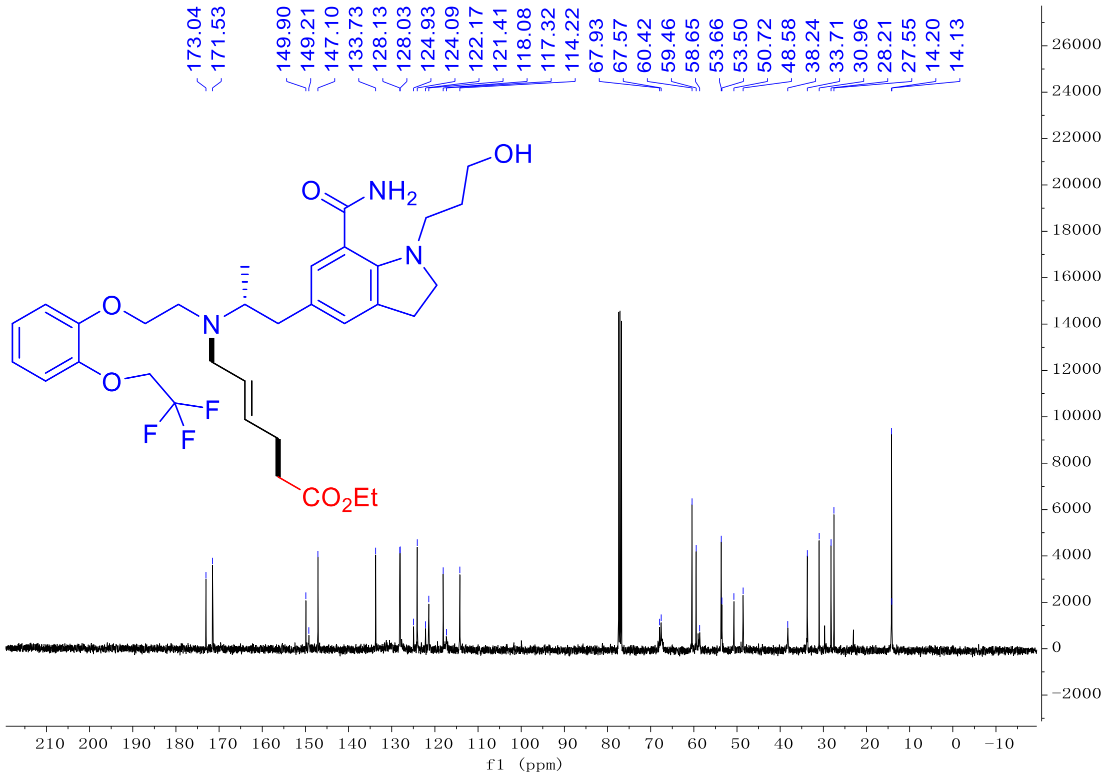

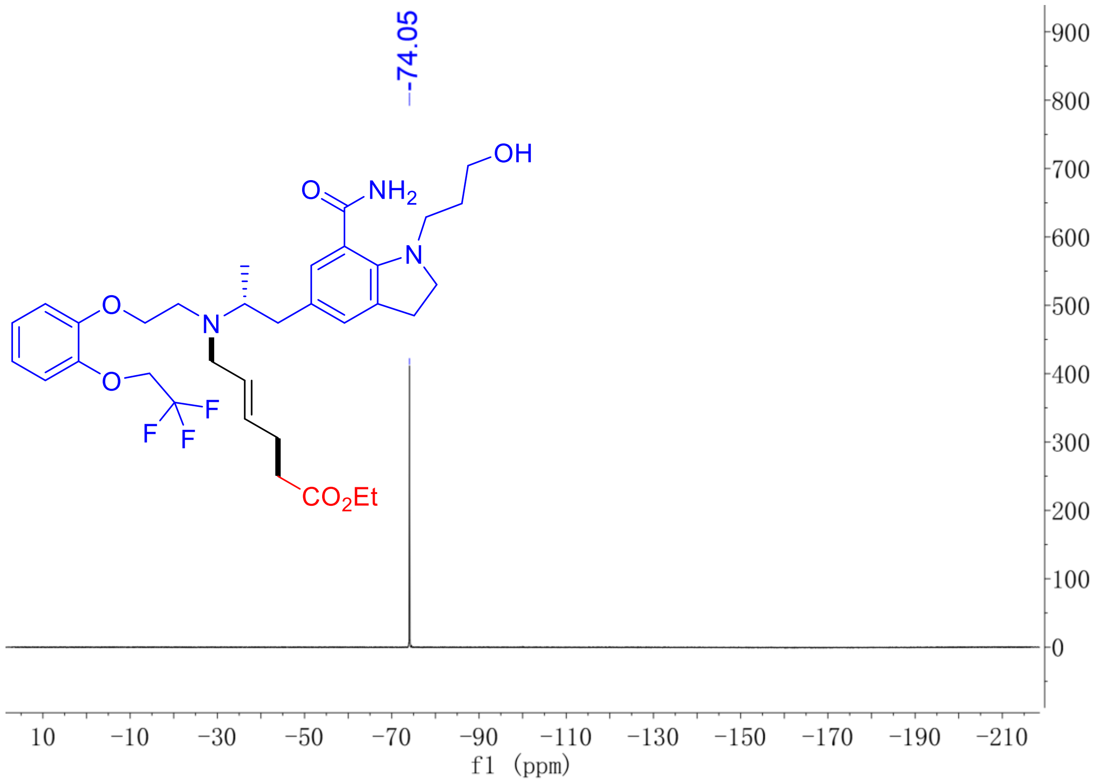

-74.05
OH
O
NH2
N
N
O
O
F
F
F
CO2Et
900
800
700
600
500
400
300
200
100
0
10
−10
−30
−50
−70
−90
−110
−130
−150
−170
−190
−210
f1 (ppm)

^{1}H NMR (400 MHz, CDCl$_3$), ^{13}C NMR (101 MHz, CDCl$_3$) and ^{19}F NMR (376 MHz, CDCl$_3$) spectra for product 4k

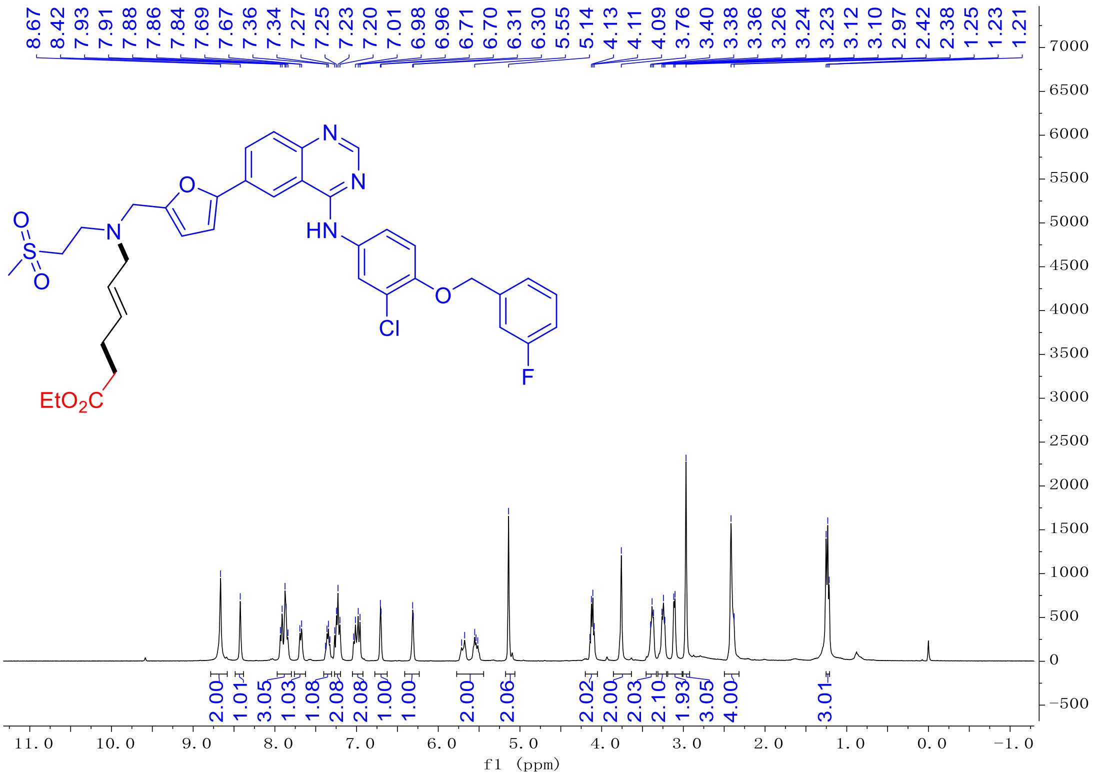

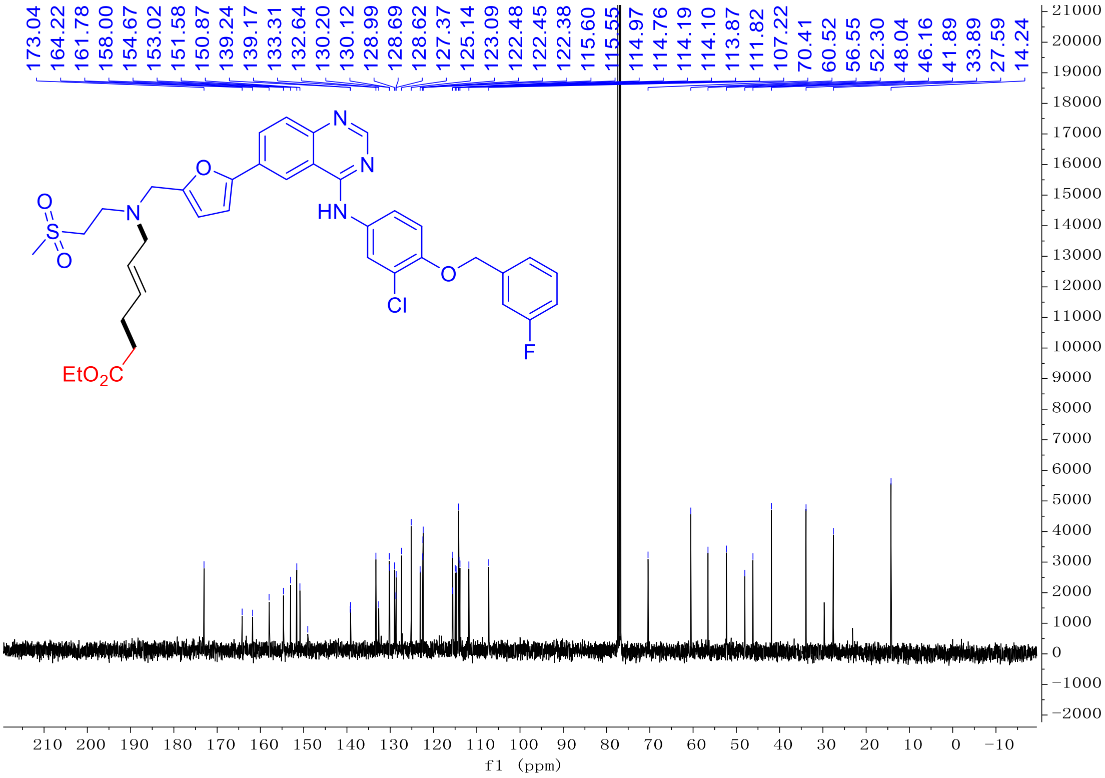

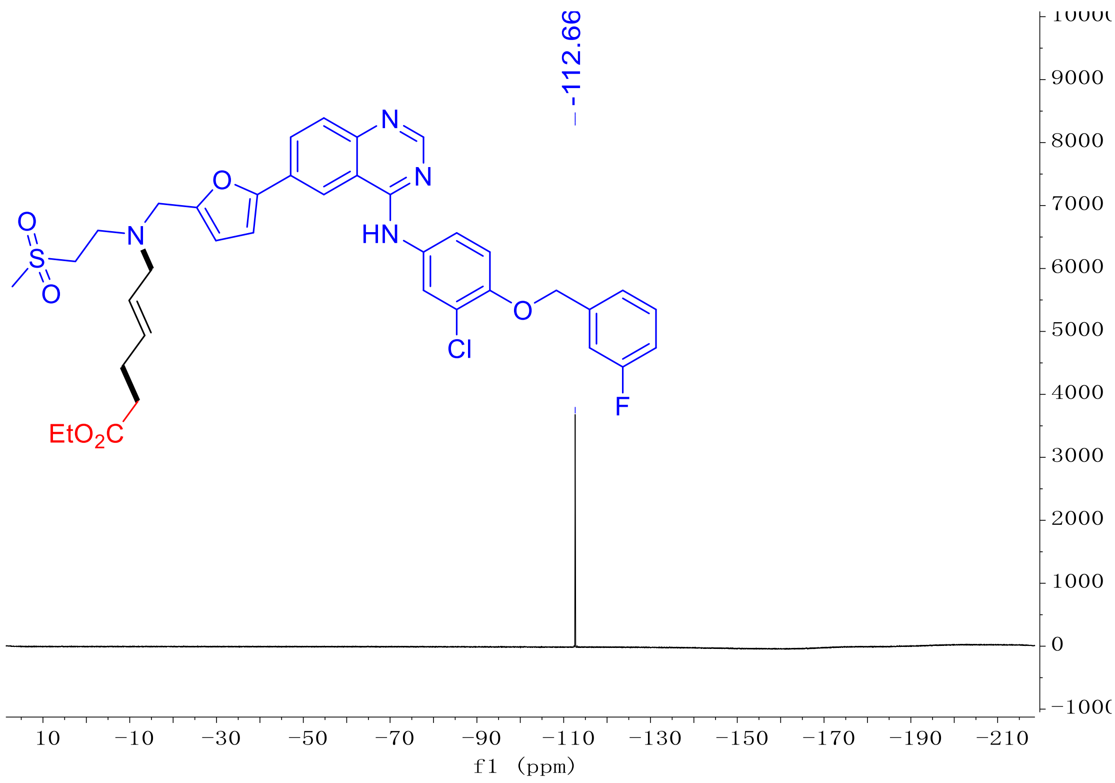

-112.66
10000
9000
8000
7000
6000
5000
4000
3000
2000
1000
0
-1000
10 -10 -30 -50 -70 -90 -110 -130 -150 -170 -190 -210
f1 (ppm)

^1H NMR (400 MHz, $CDCl_3$) and ^{13}C NMR (101 MHz, $CDCl_3$) spectra for product 4l

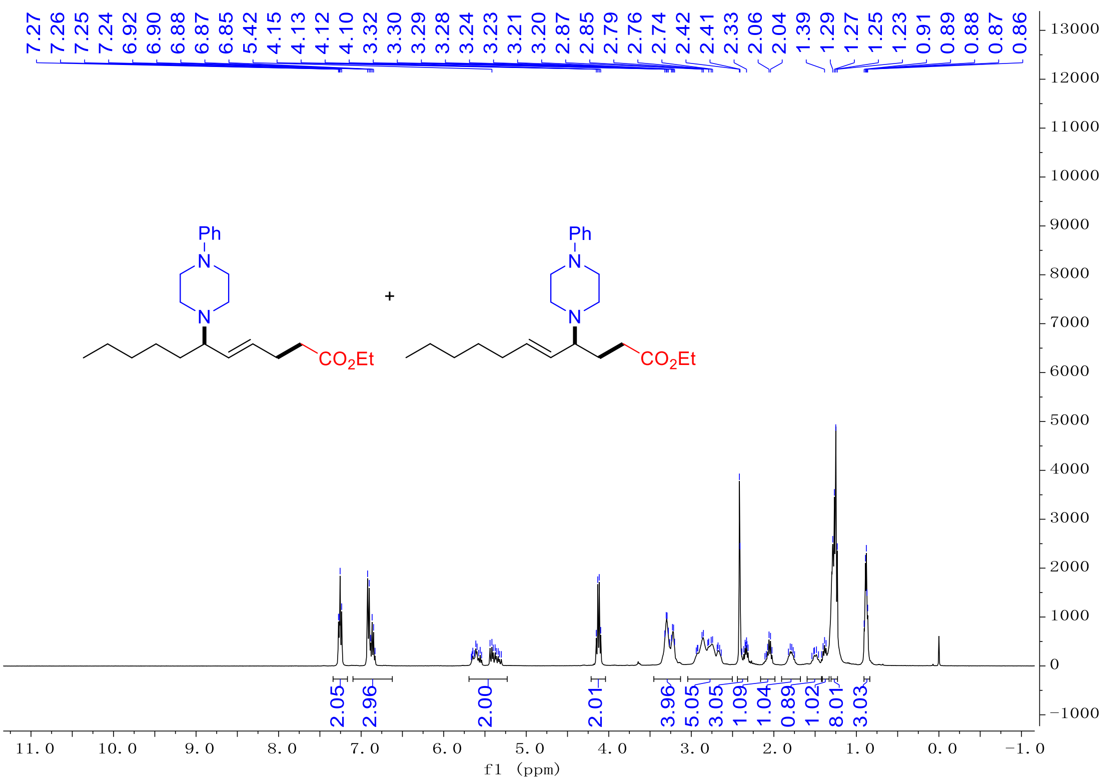

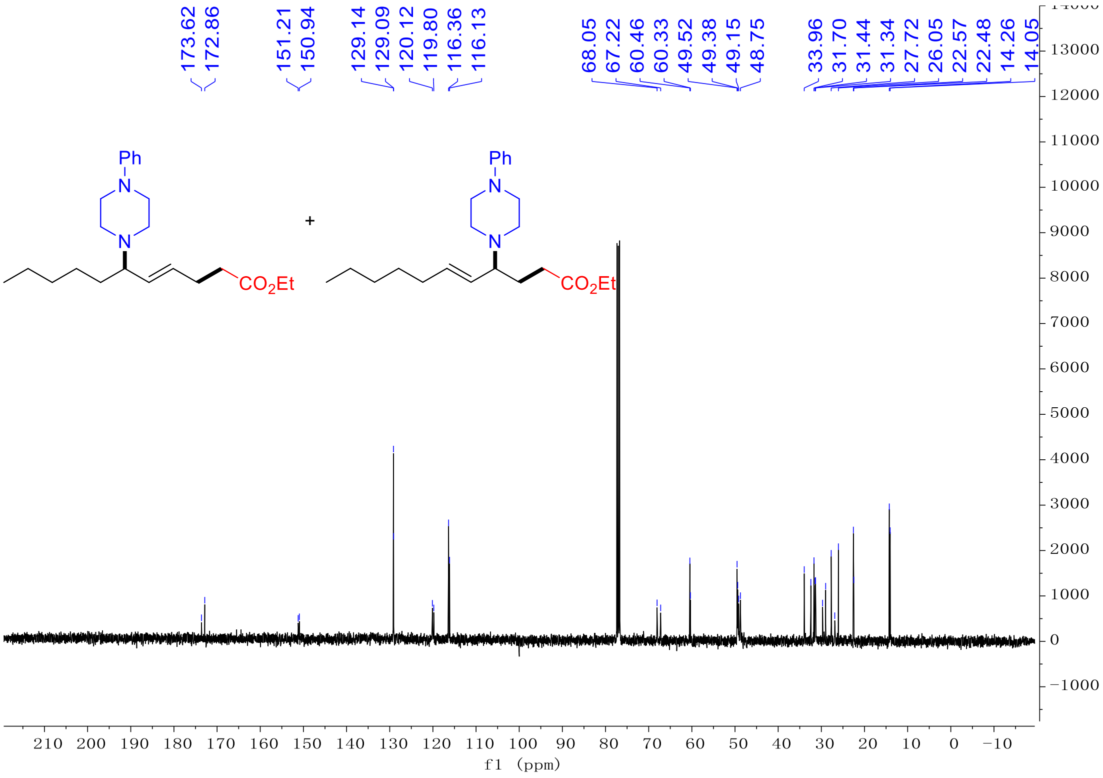

^{1}H NMR (400 MHz, CDCl$_3$) and ^{13}C NMR (126 MHz, CDCl$_3$) spectra for product 4m

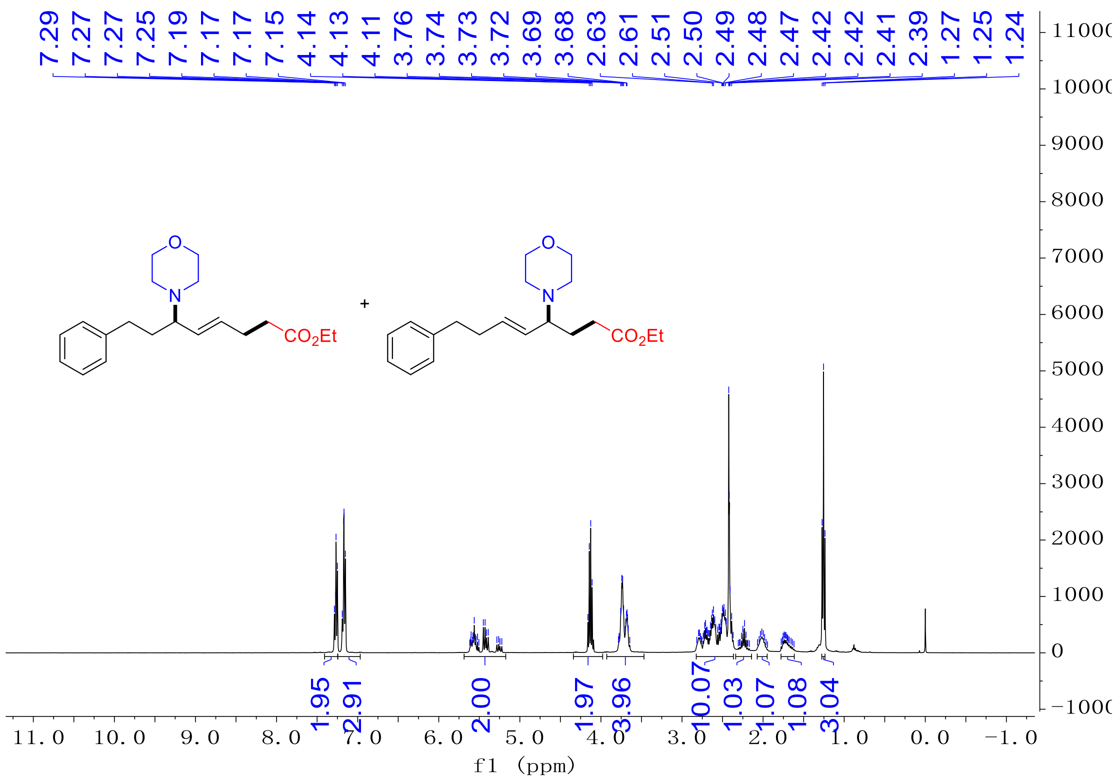

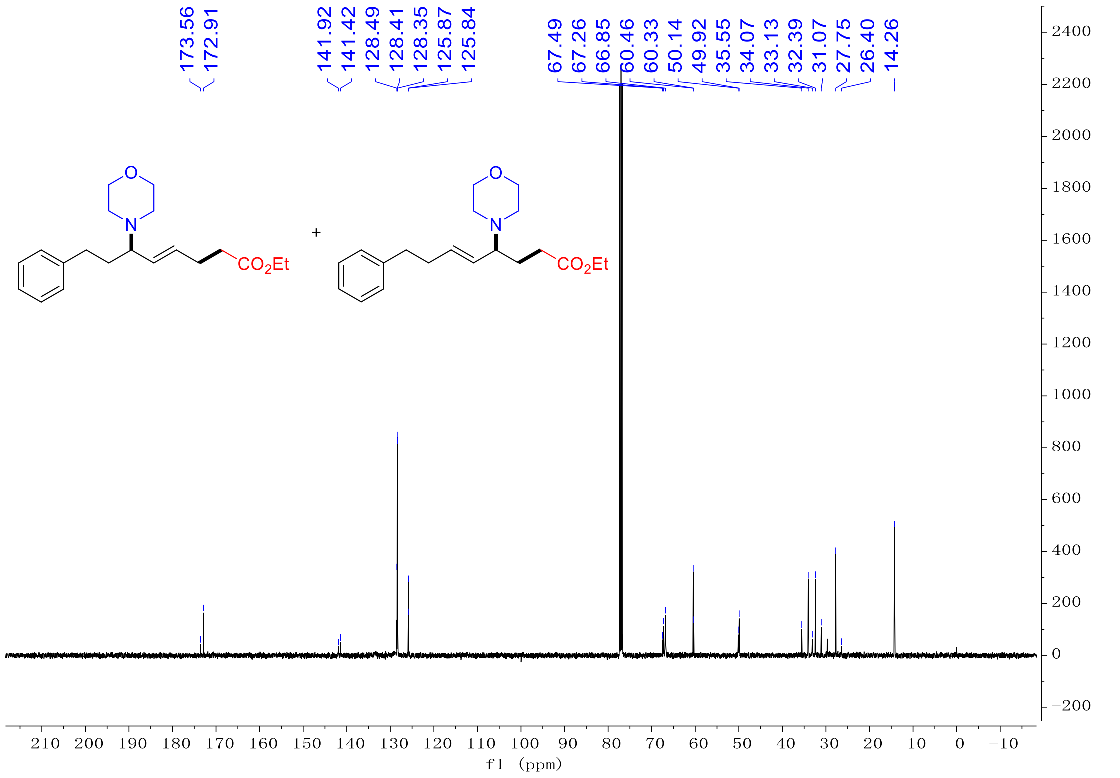

^{1}H NMR (400 MHz, CDCl3) and ^{13}C NMR (126 MHz, CDCl3) spectra for product 4n

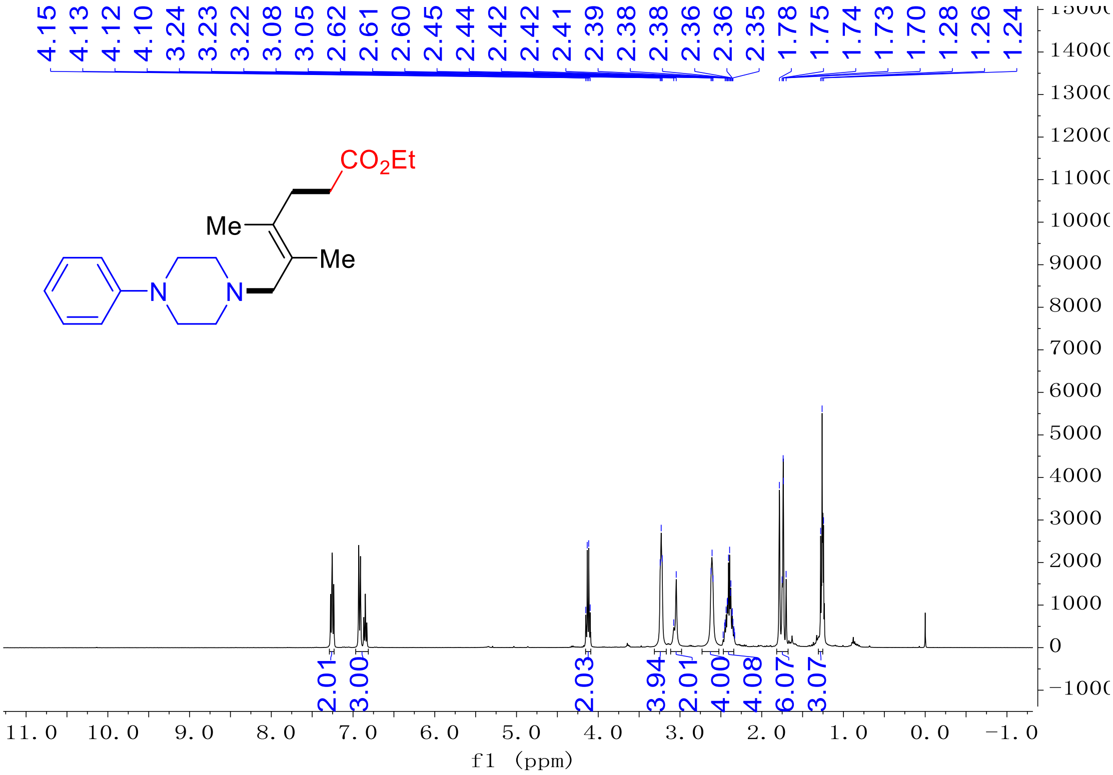

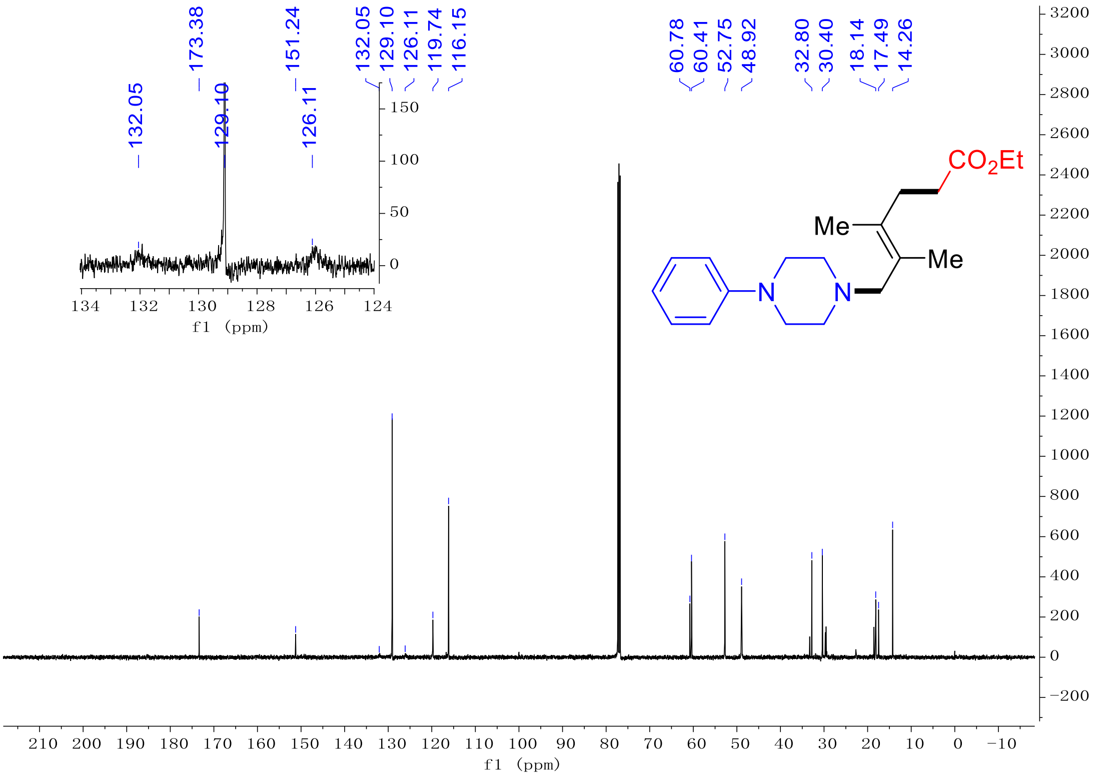

^{1}H NMR (500 MHz, CDCl₃) and ^{13}C NMR (126 MHz, CDCl₃) spectra for product 4o

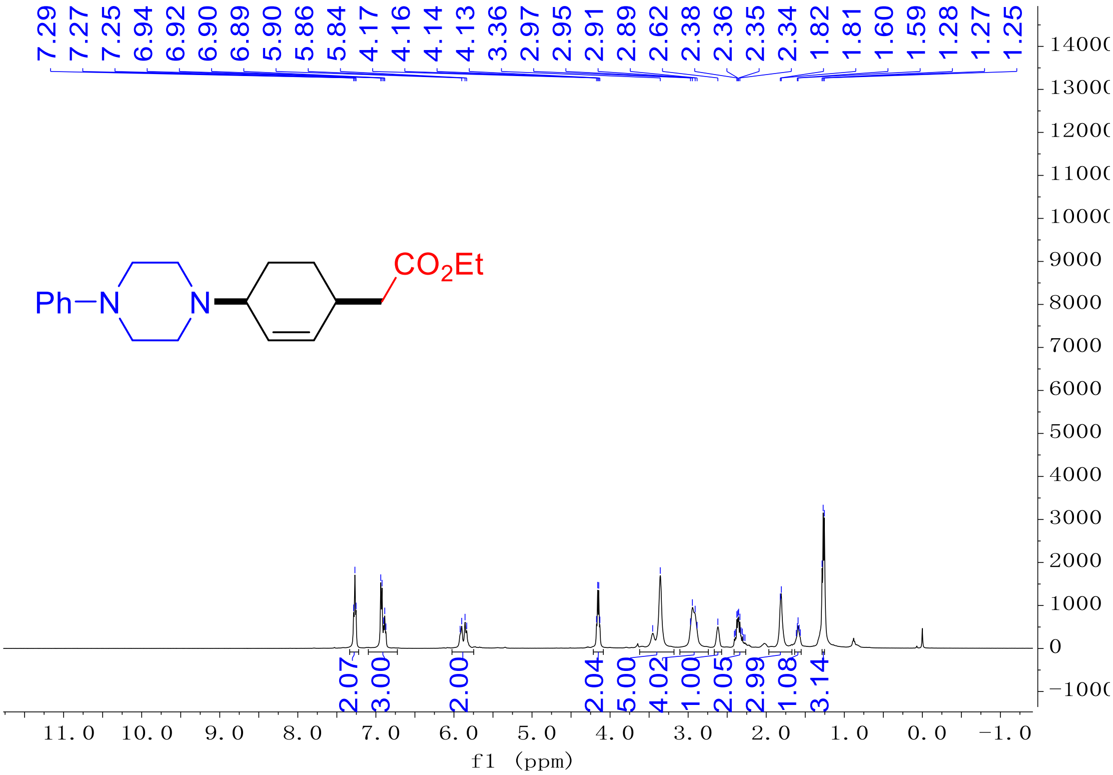

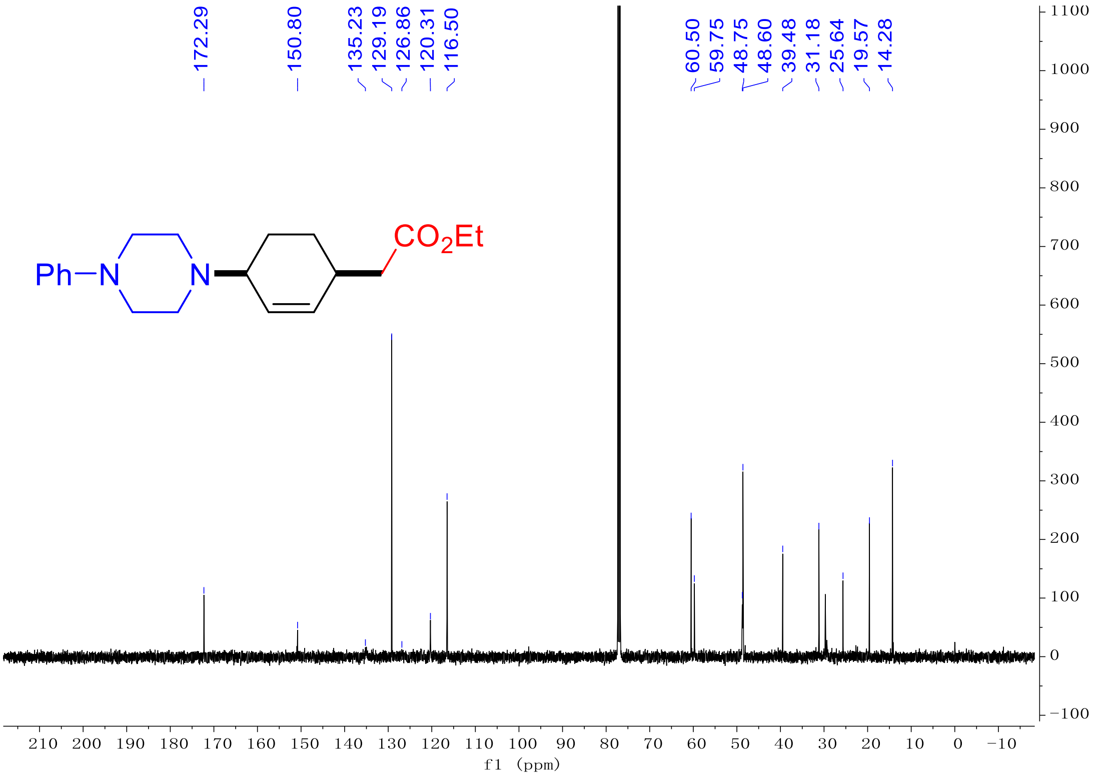

^{1}H NMR (400 MHz, CDCl3) and ^{13}C NMR (101 MHz, CDCl3) spectra for product 4p

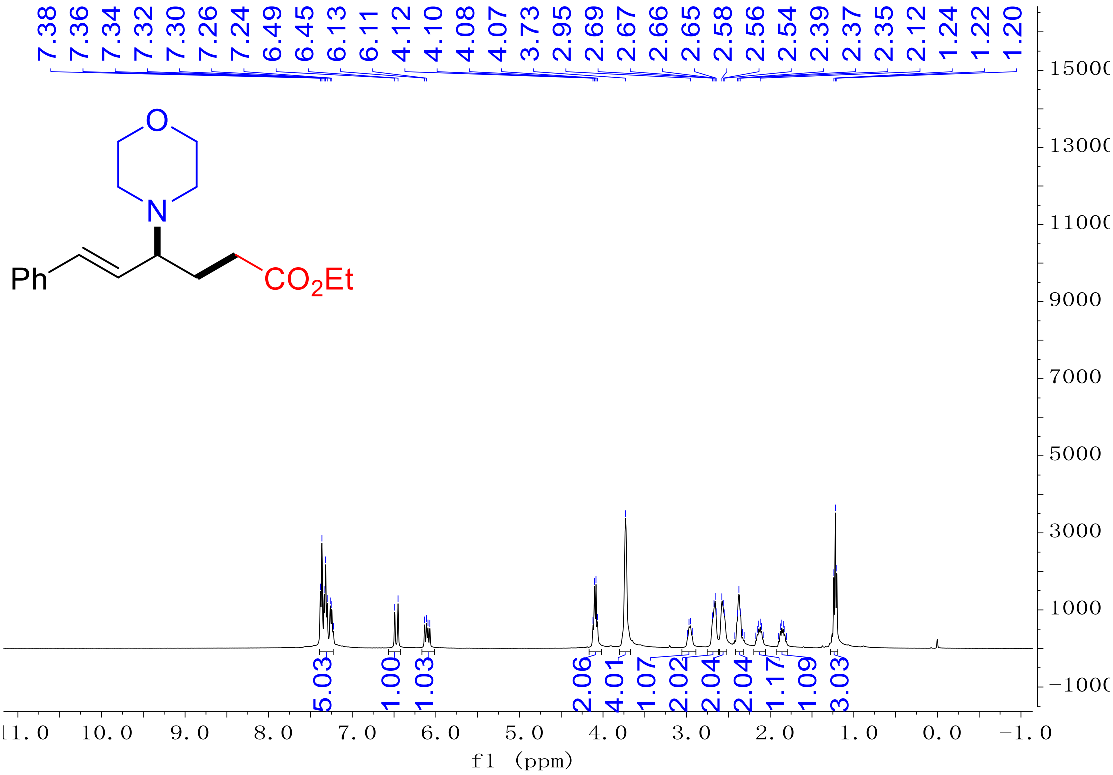

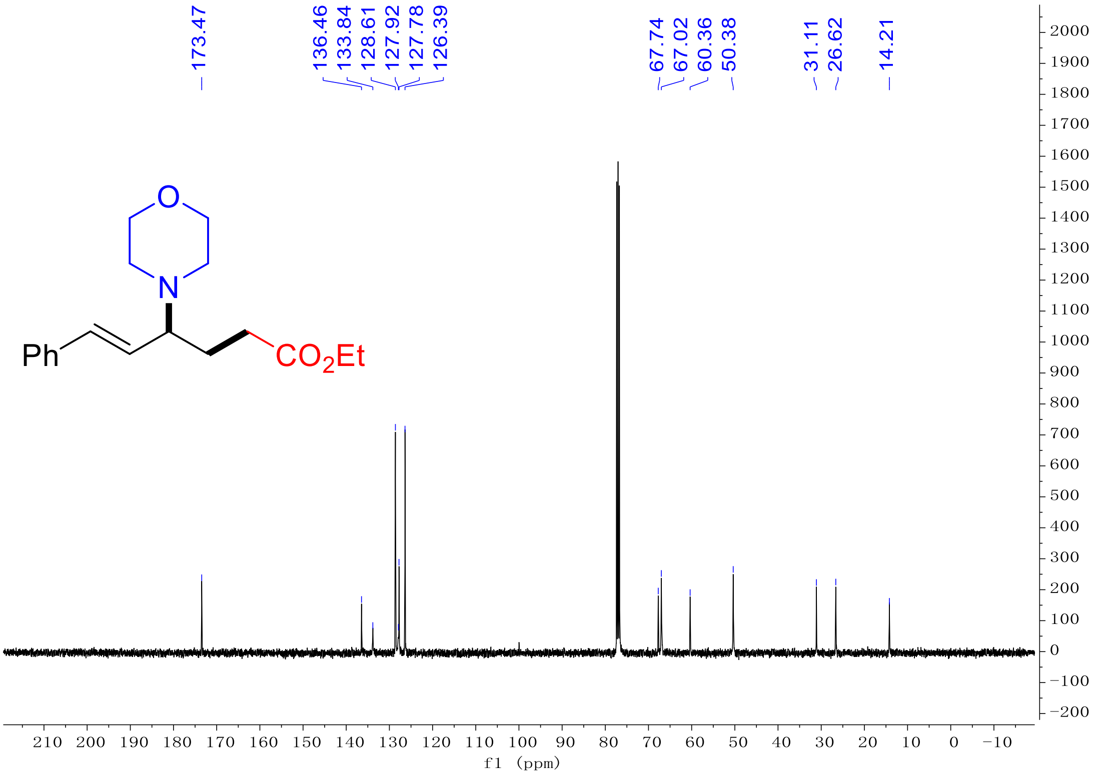

^{1}H NMR (400 MHz, $CDCl_3$) and ^{13}C NMR (101 MHz, $CDCl_3$) spectra for product 4q

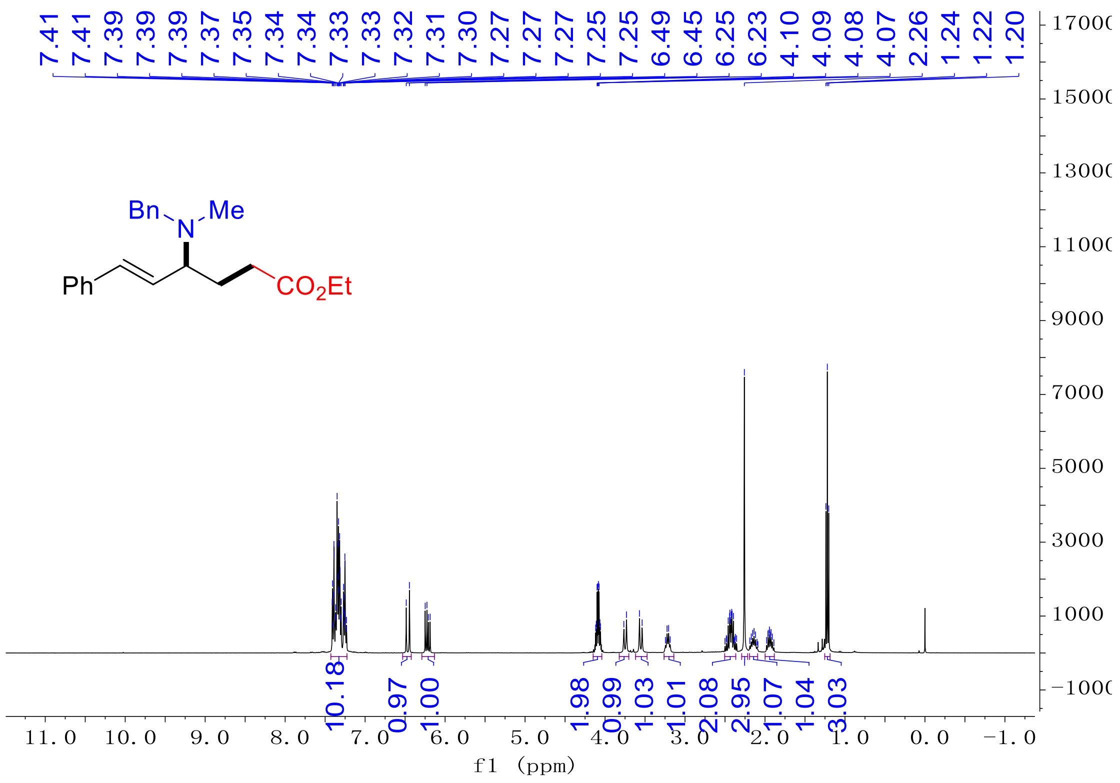

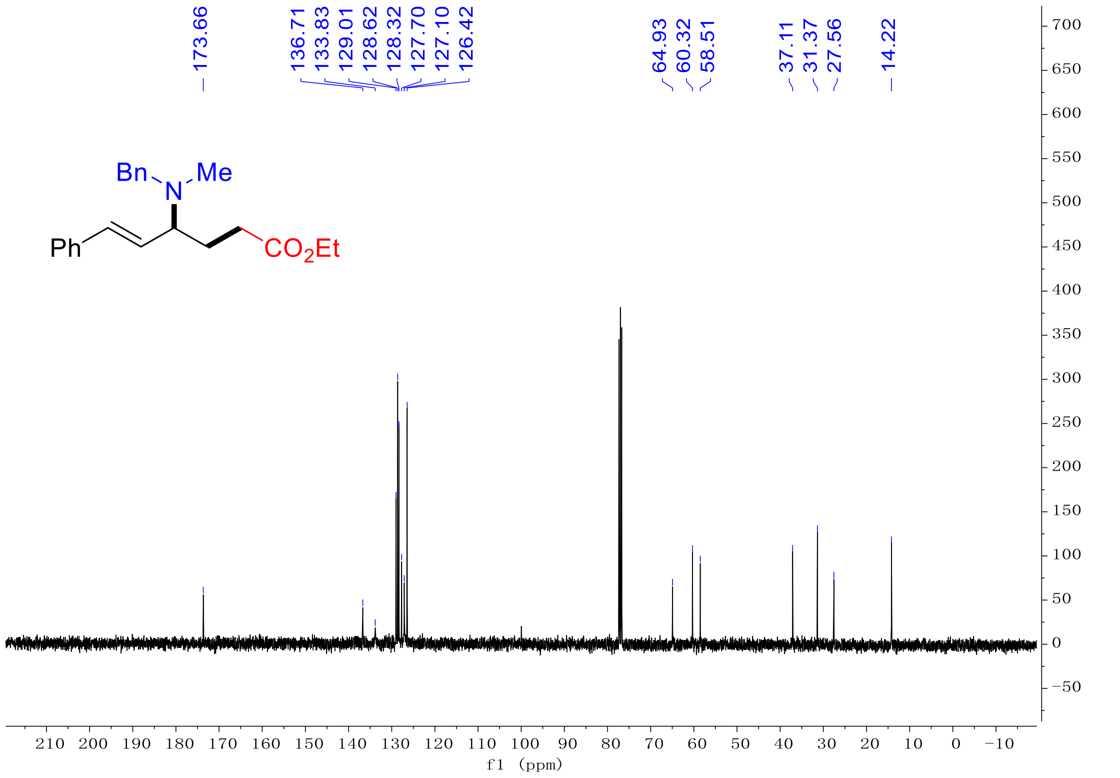

^{1}H NMR (400 MHz, $CDCl_3$) and ^{13}C NMR (101 MHz, $CDCl_3$) spectra for product 4r

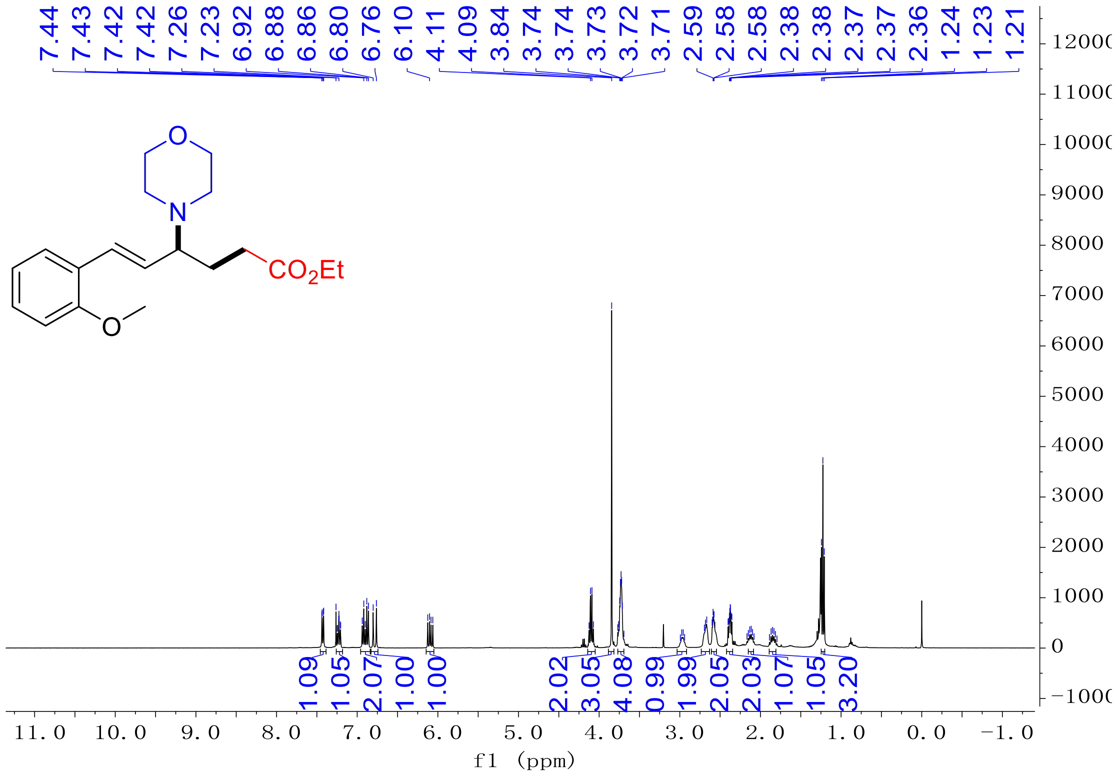

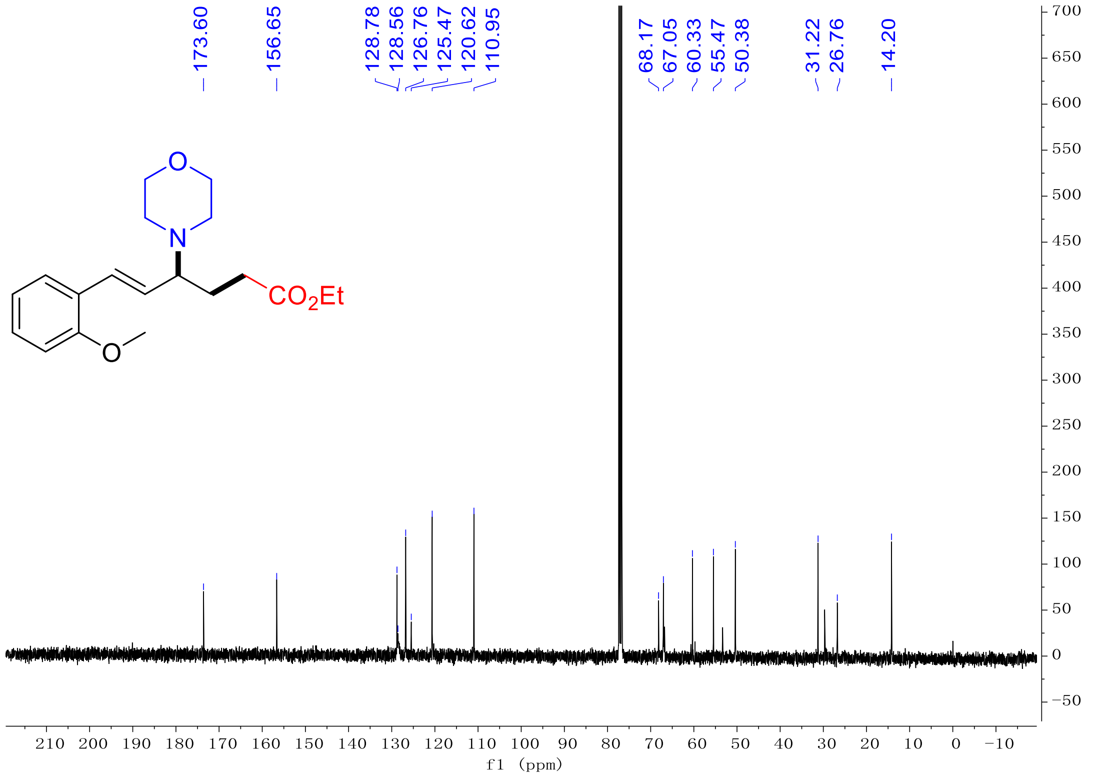

^{1}H NMR (400 MHz, $CDCl_3$) and ^{13}C NMR (101 MHz, $CDCl_3$) spectra for product 4s

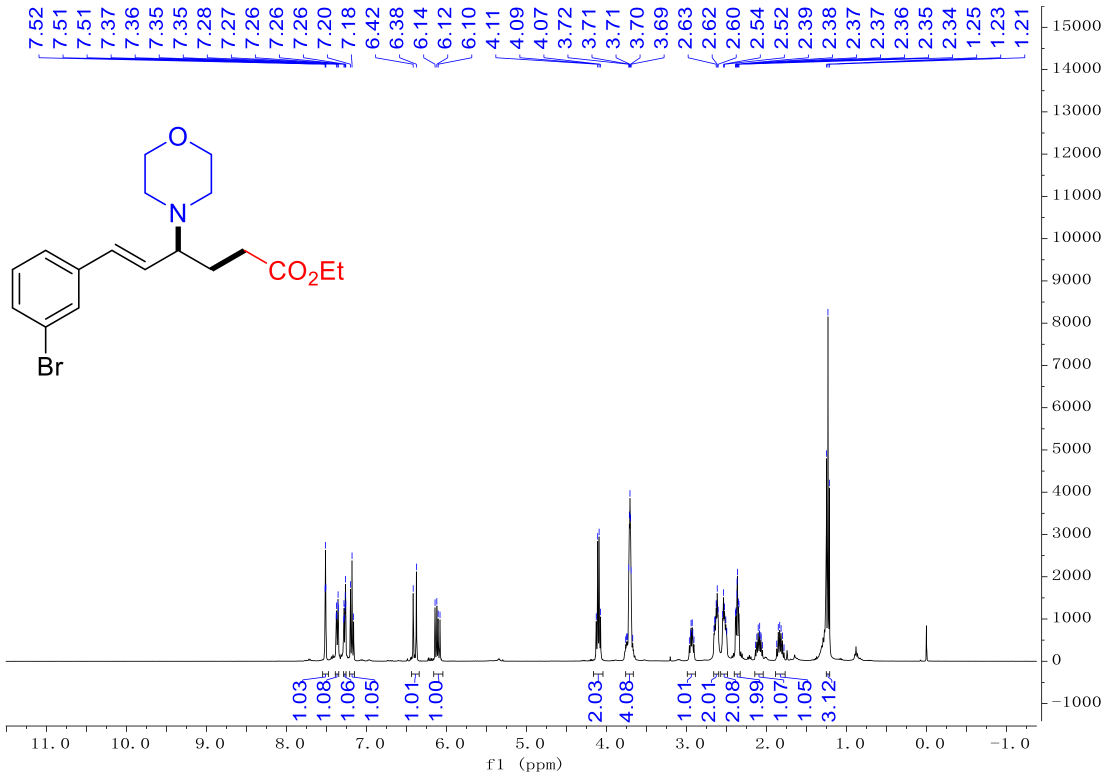

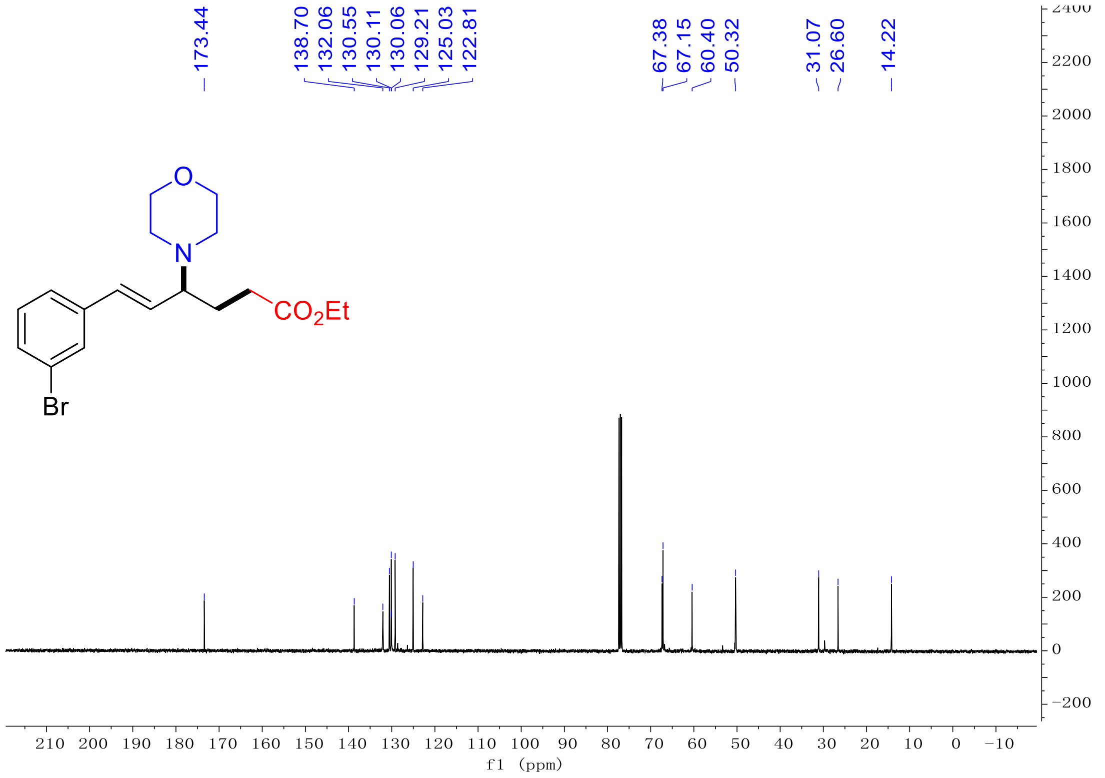

^{1}H NMR (400 MHz, $CDCl_3$) and ^{13}C NMR (101 MHz, $CDCl_3$) spectra for product 4t

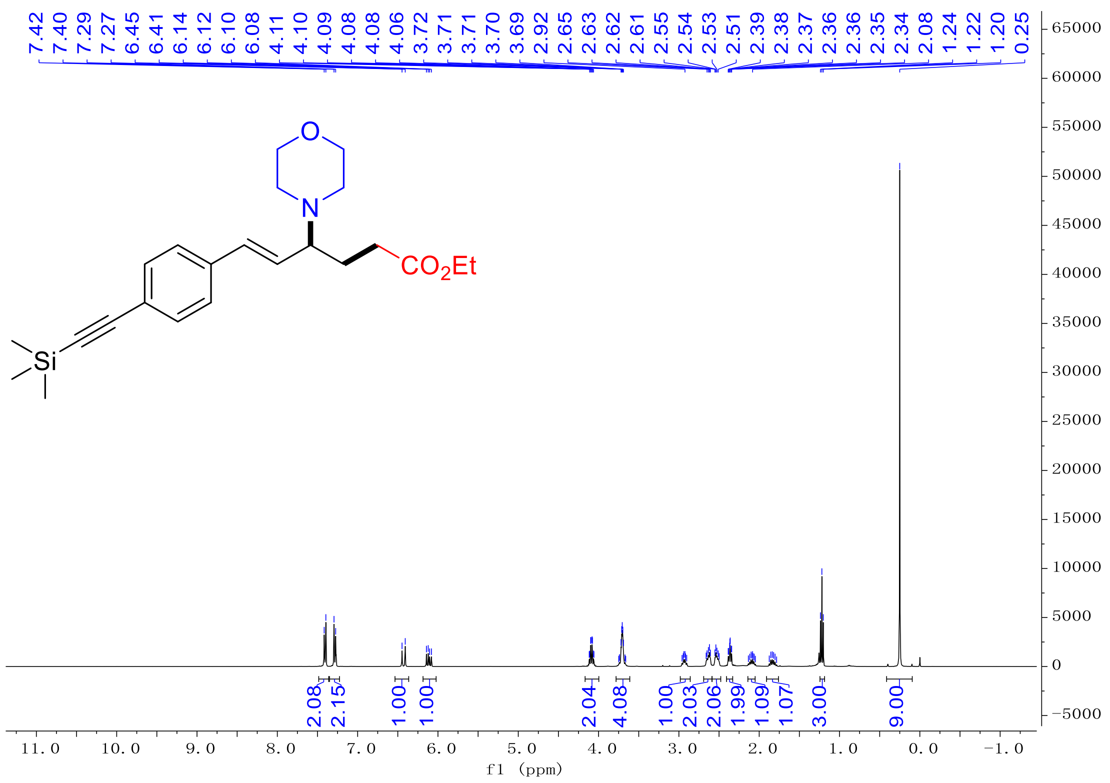

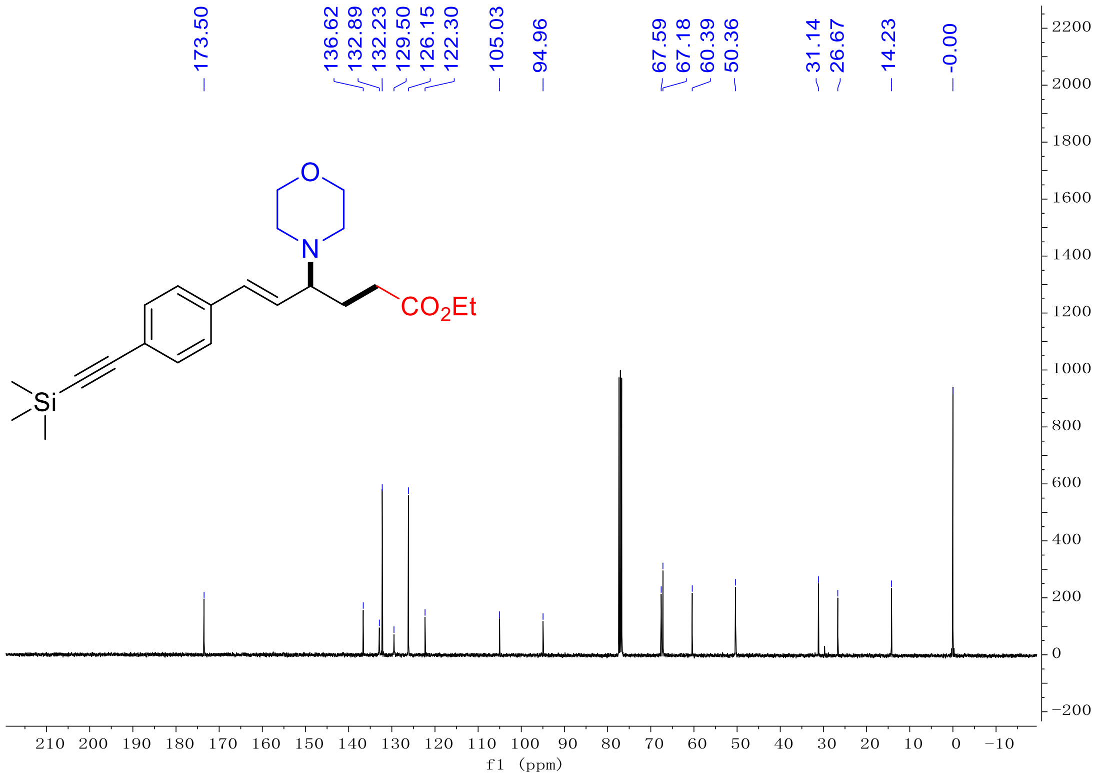

^{1}H NMR (400 MHz, CDCl$_3$) and ^{13}C NMR (126 MHz, CDCl$_3$) spectra for product 4u

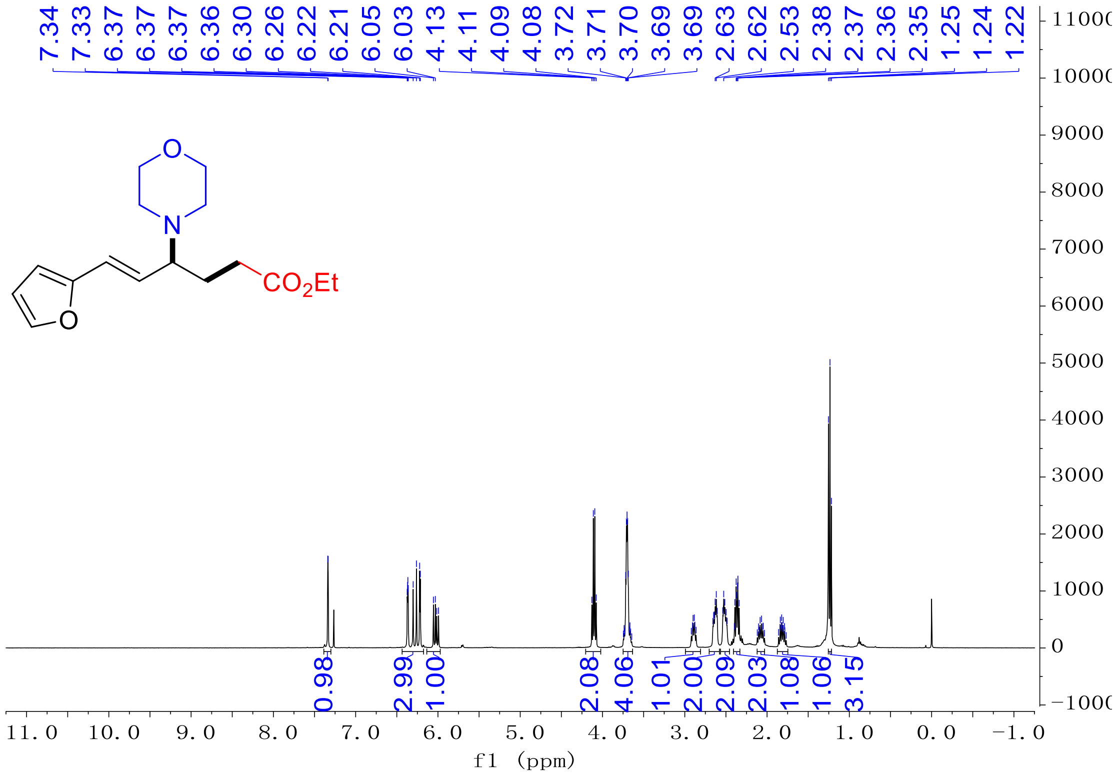

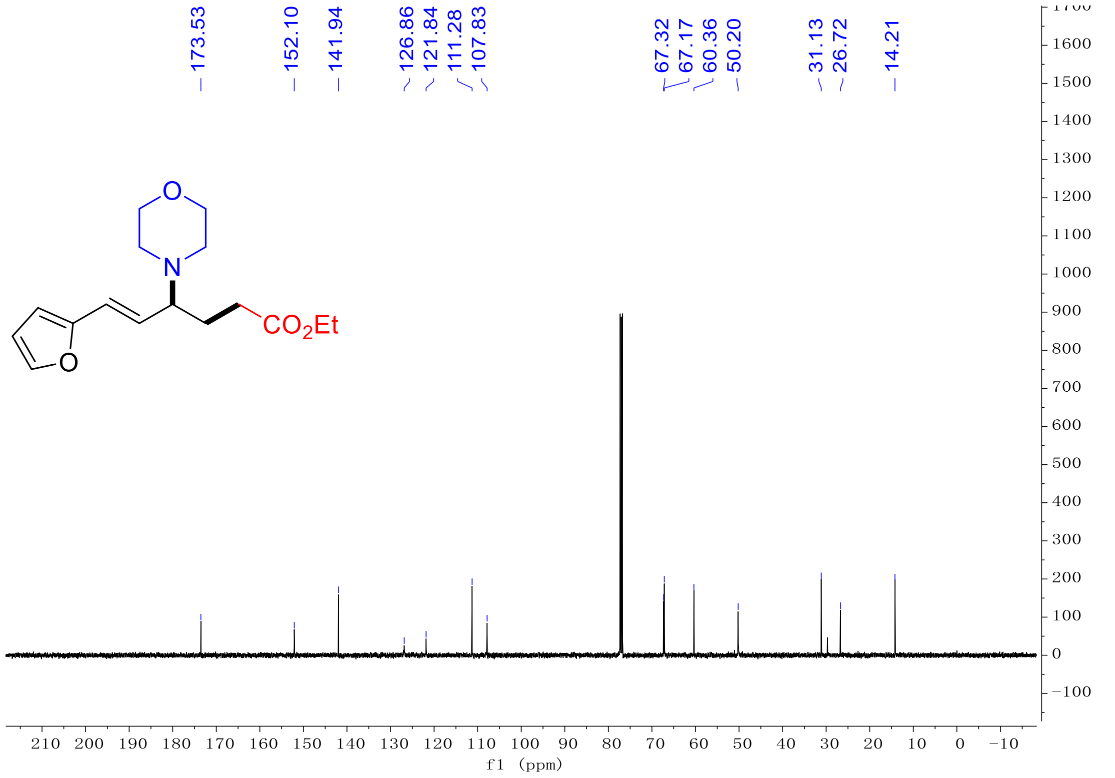

^{1}H NMR (400 MHz, CDCl3) and ^{13}C NMR (101 MHz, CDCl3) spectra for product 4v

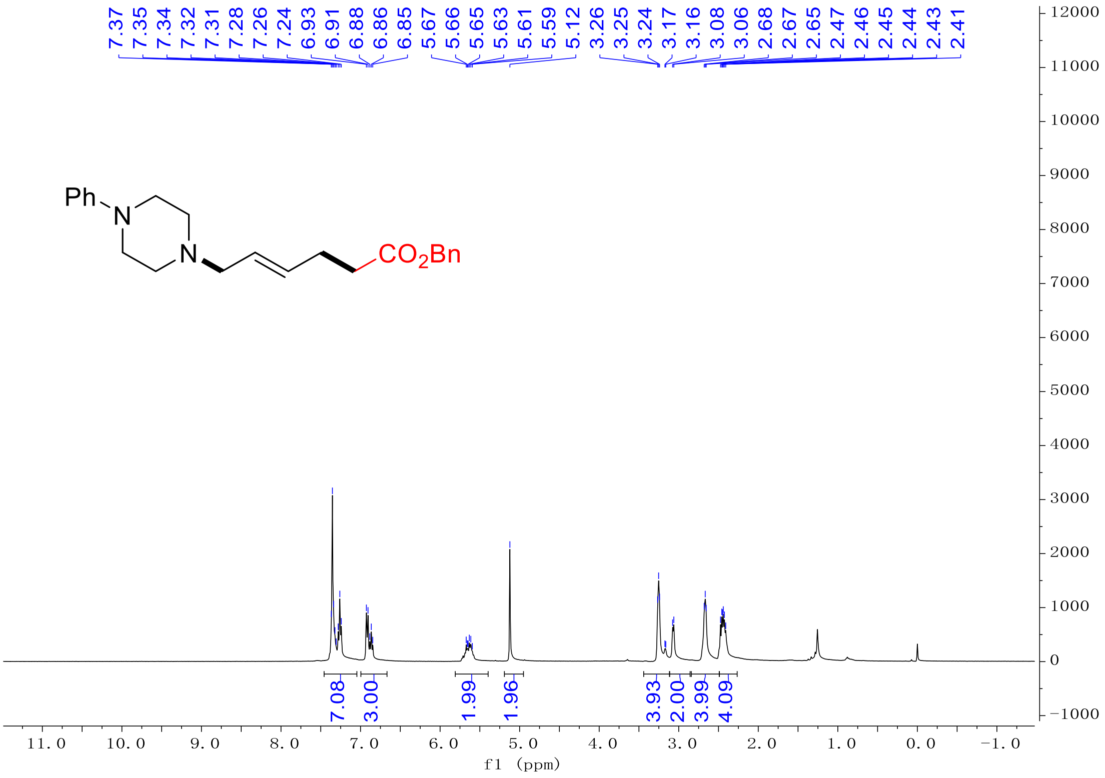

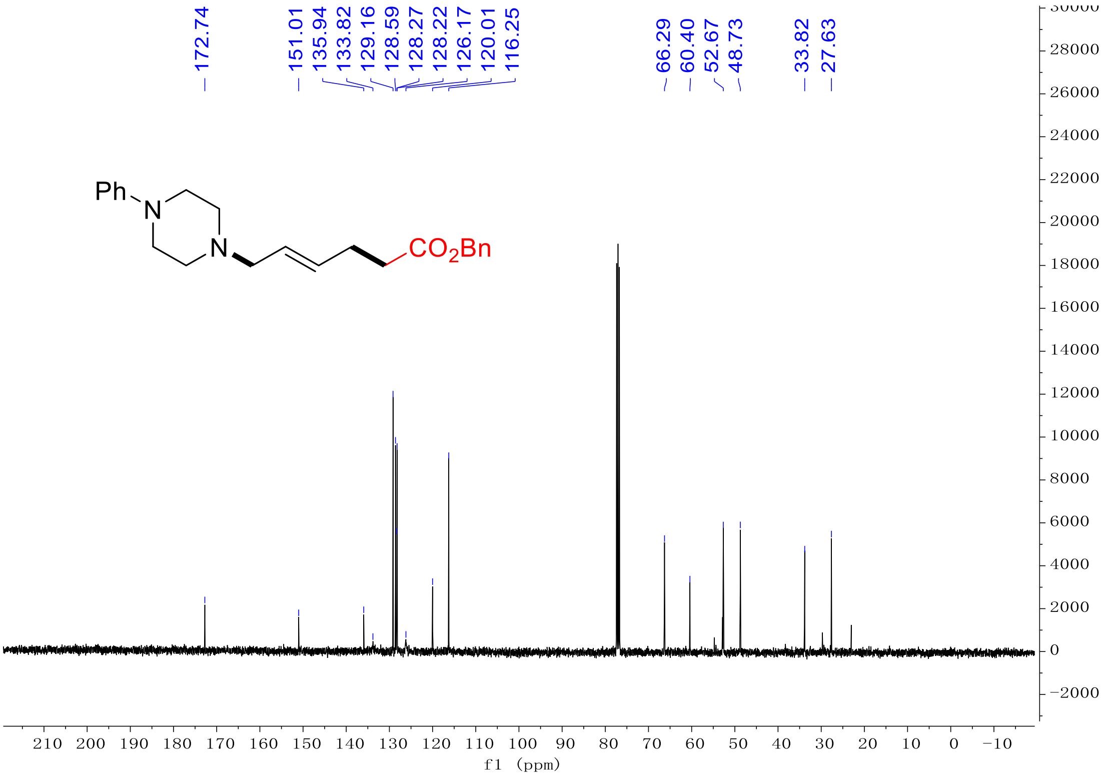

^{1}H NMR (400 MHz, $CDCl_3$) and ^{13}C NMR (101 MHz, $CDCl_3$) spectra for product 4w

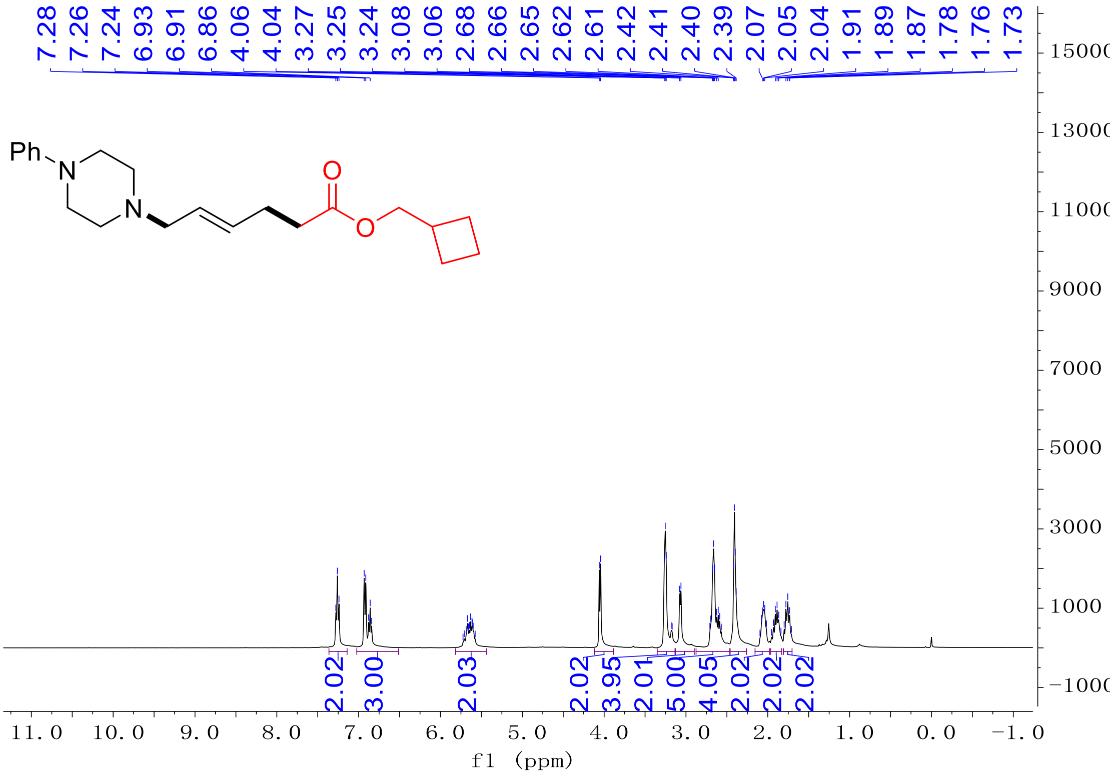

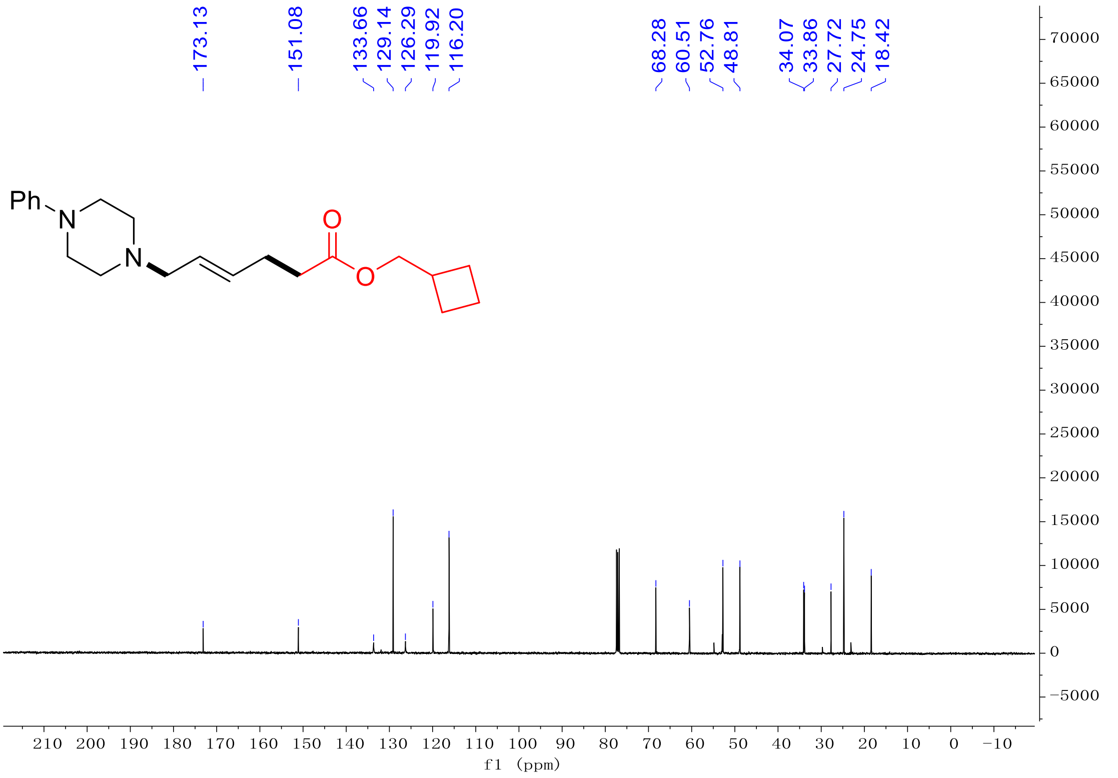

^{1}H NMR (400 MHz, $CDCl_3$) and ^{13}C NMR (126 MHz, $CDCl_3$) spectra for product 4x

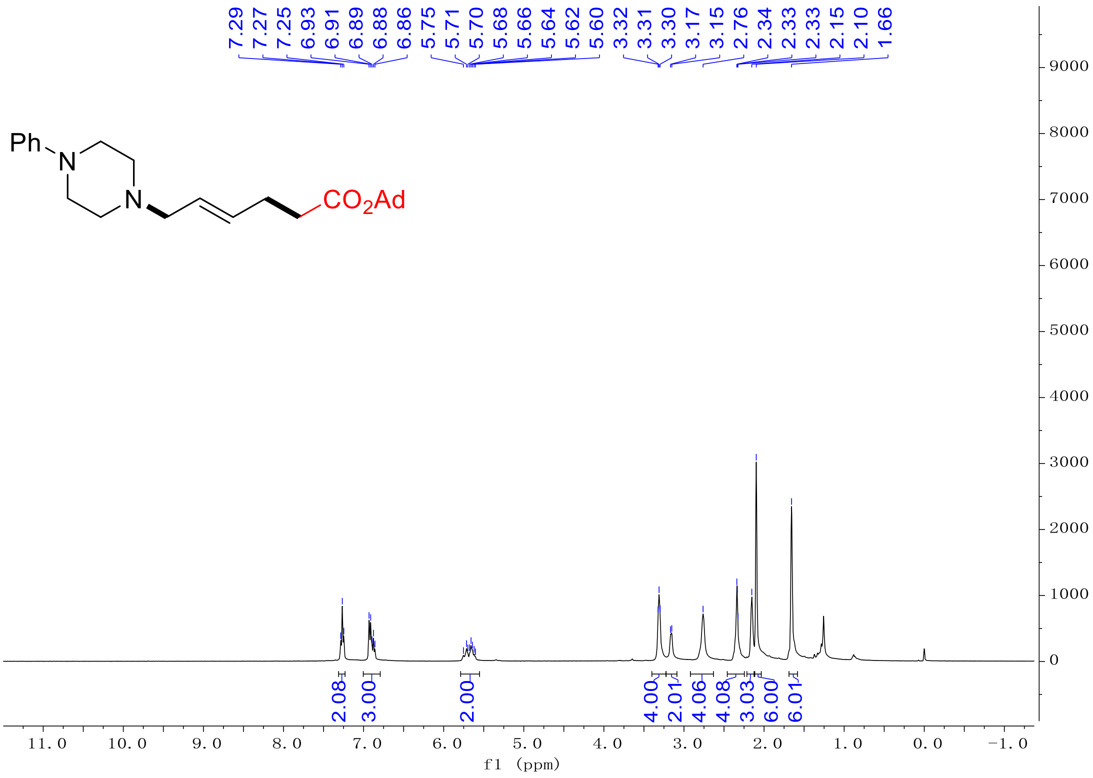

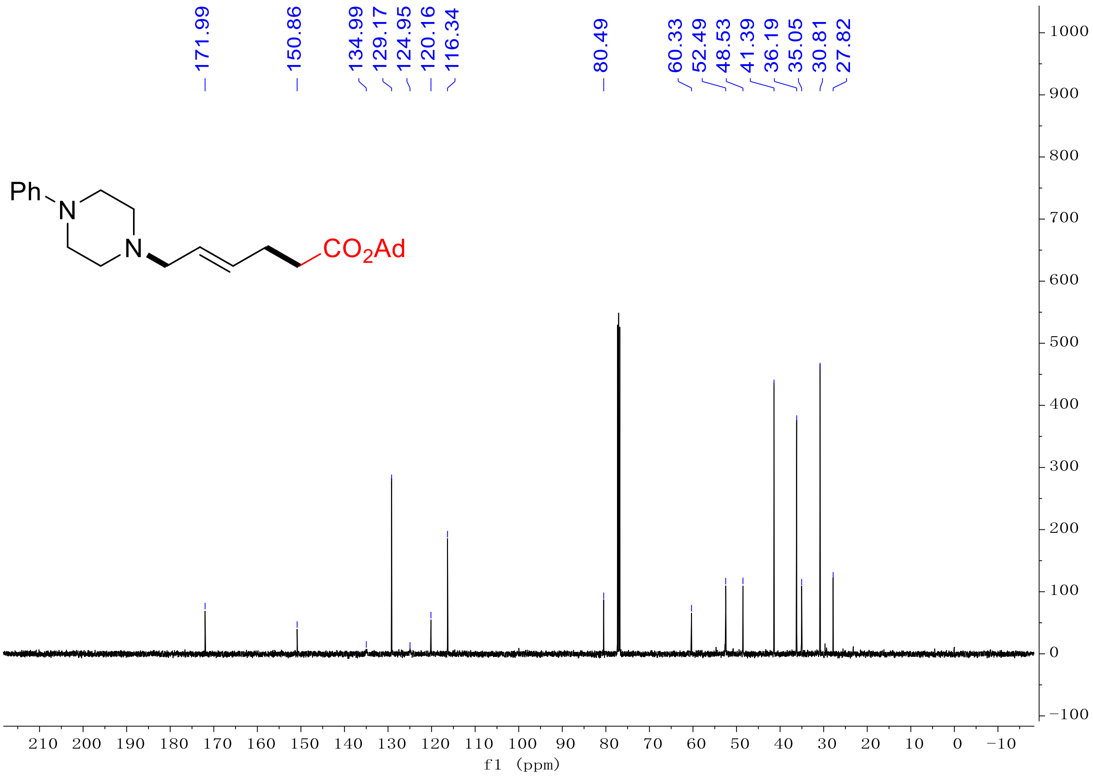

^{1}H NMR (400 MHz, $CDCl_3$) and ^{13}C NMR (101 MHz, $CDCl_3$) spectra for product 4y

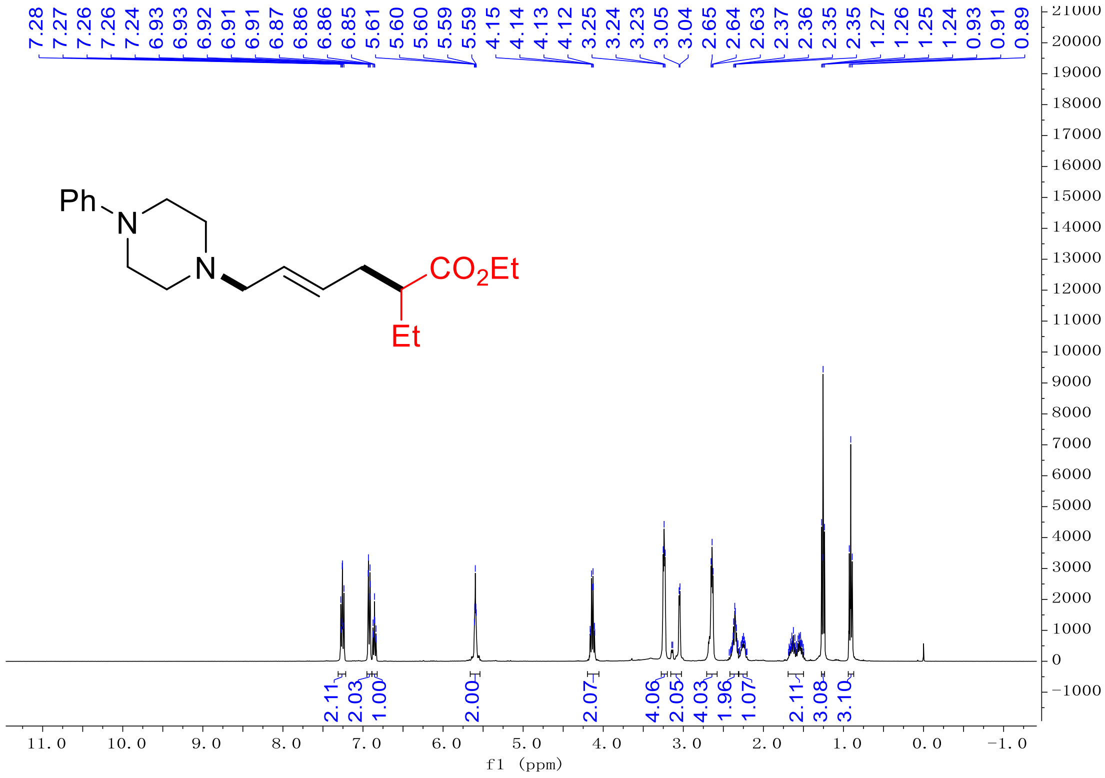

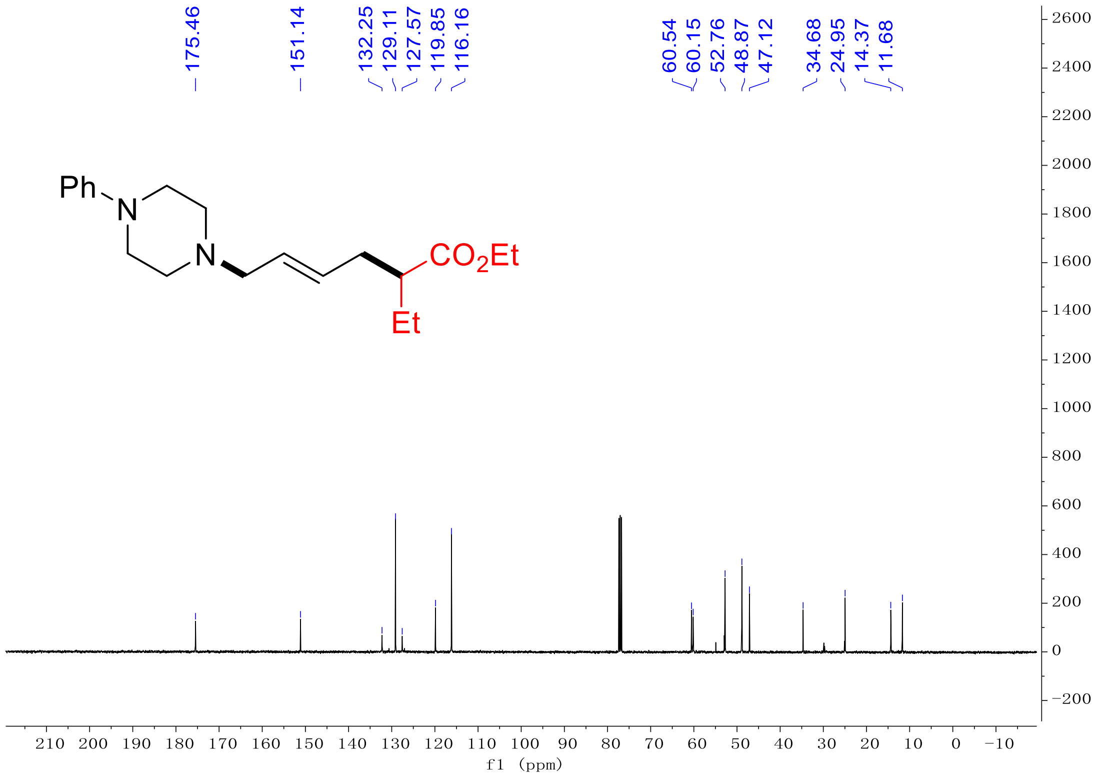

^{1}H NMR (400 MHz, CDCl3) and ^{13}C NMR (101 MHz, CDCl3) spectra for product 4z

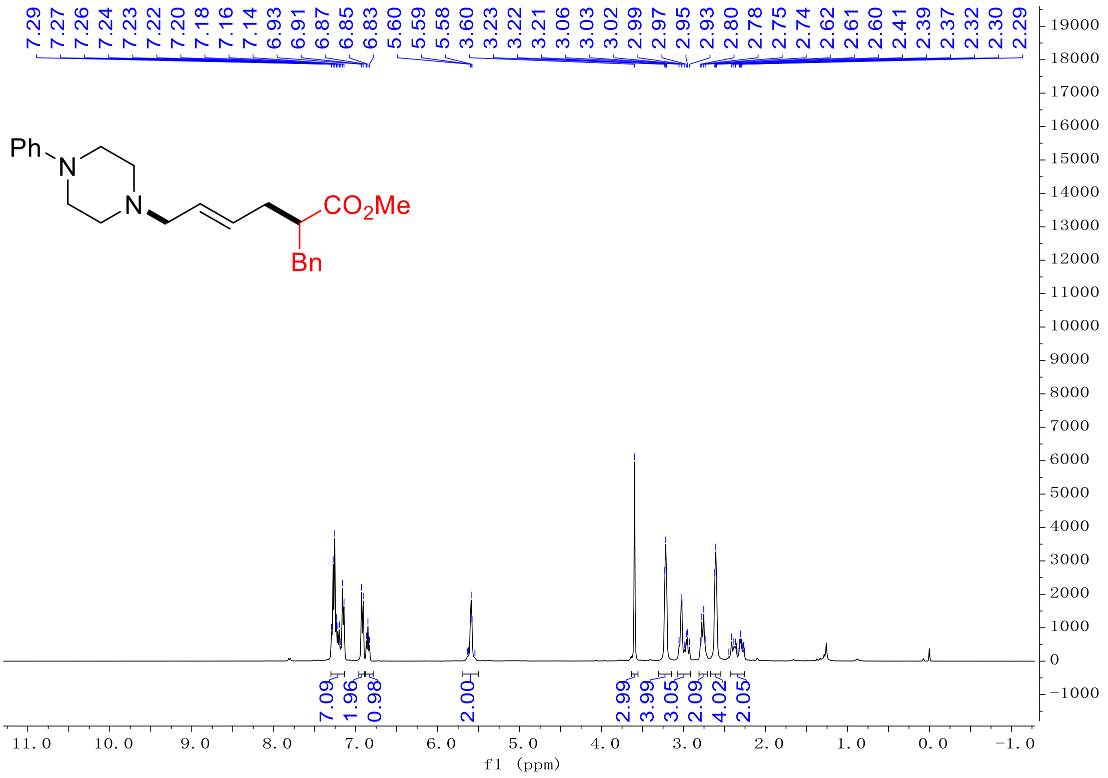

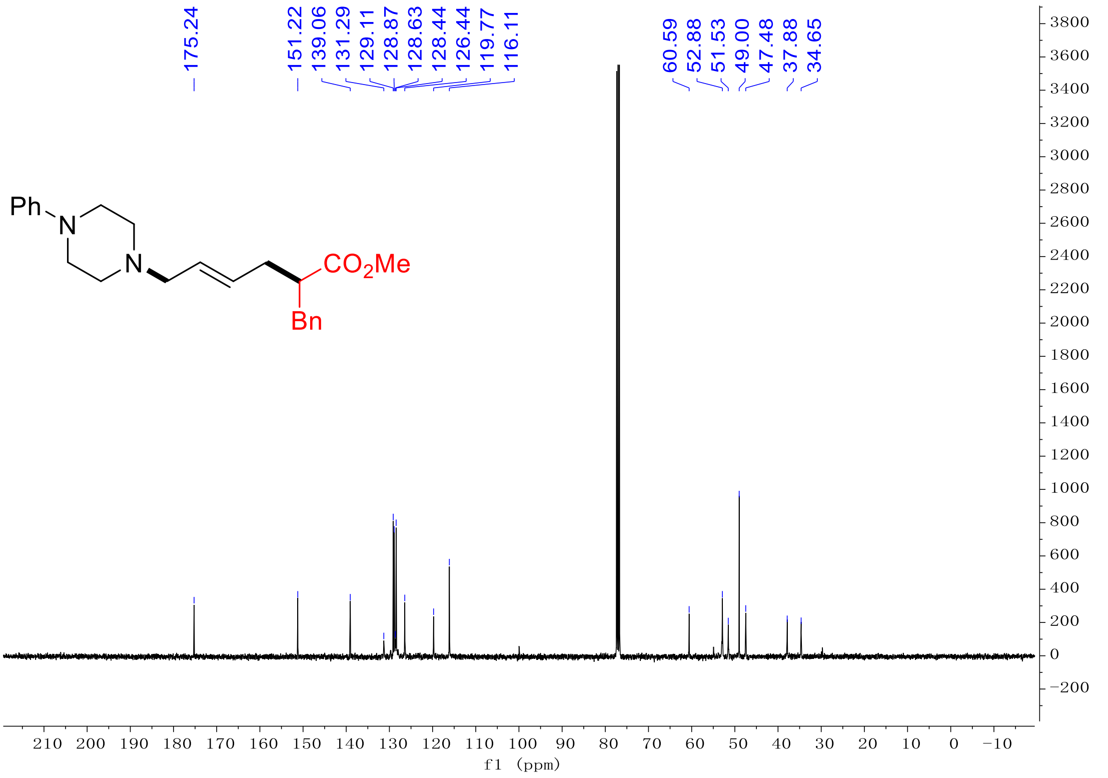

^1H NMR (400 MHz, $CDCl_3$) and ^{13}C NMR (101 MHz, $CDCl_3$) spectra for product 4aa

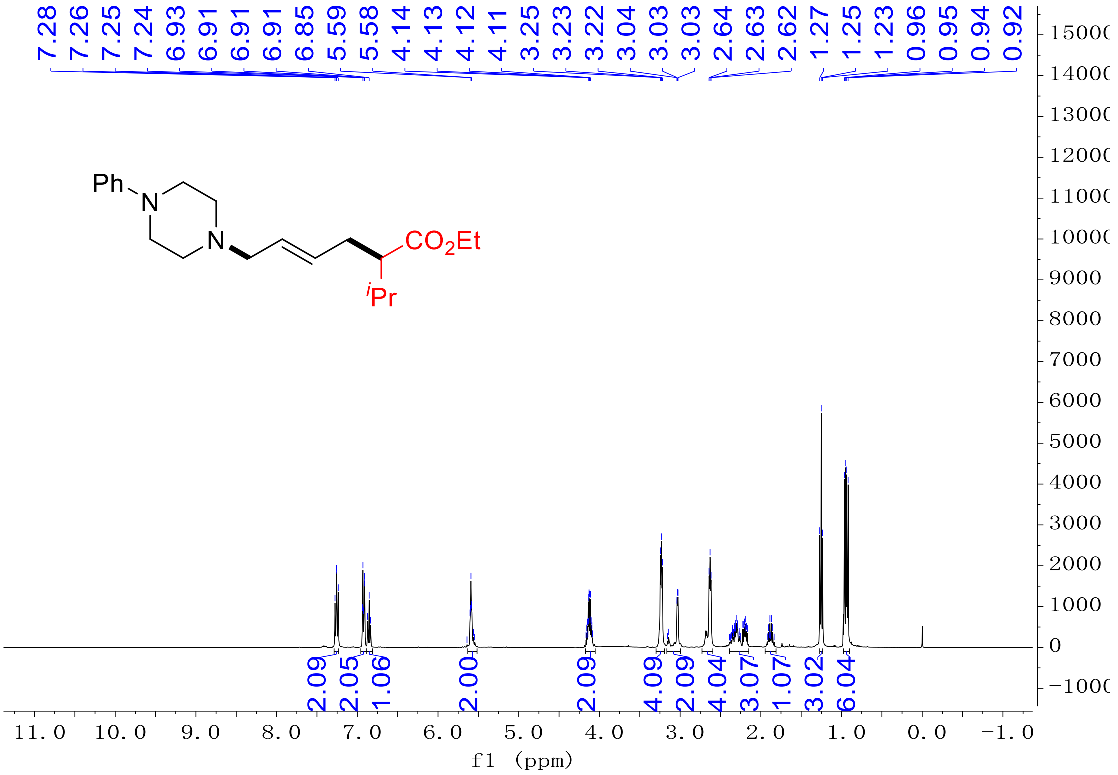

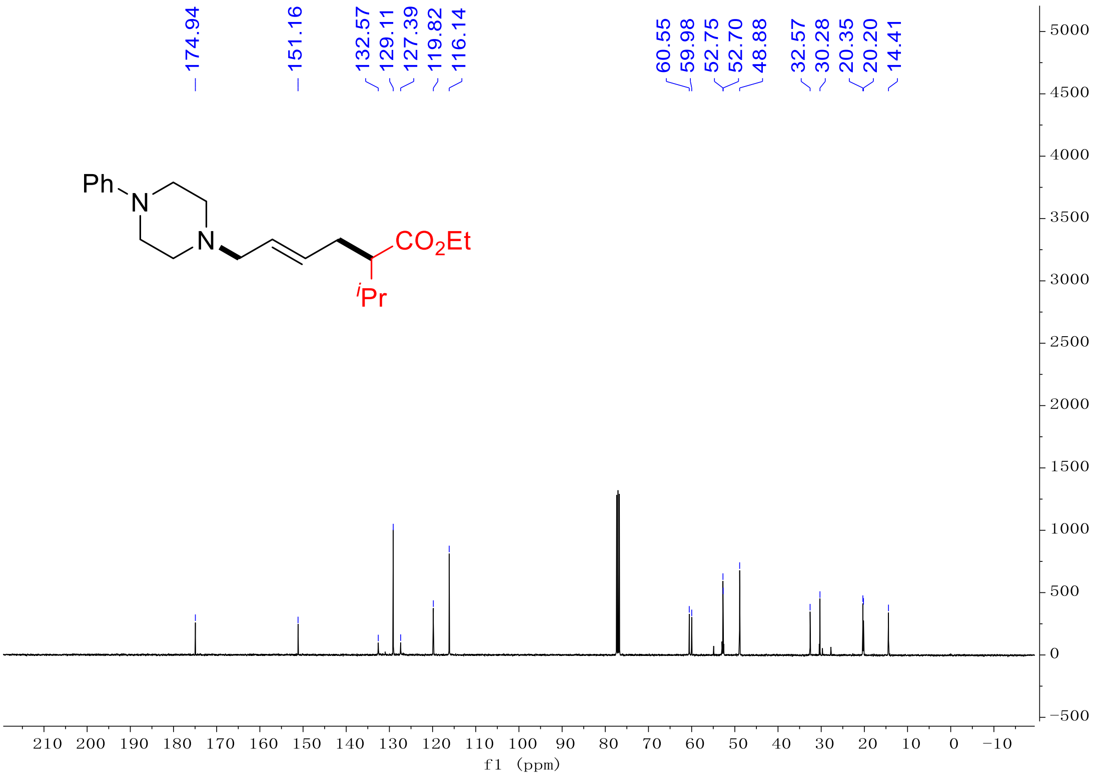

^{1}H NMR (500 MHz, $CDCl_3$) and ^{13}C NMR (126 MHz, $CDCl_3$) spectra for product 4ab

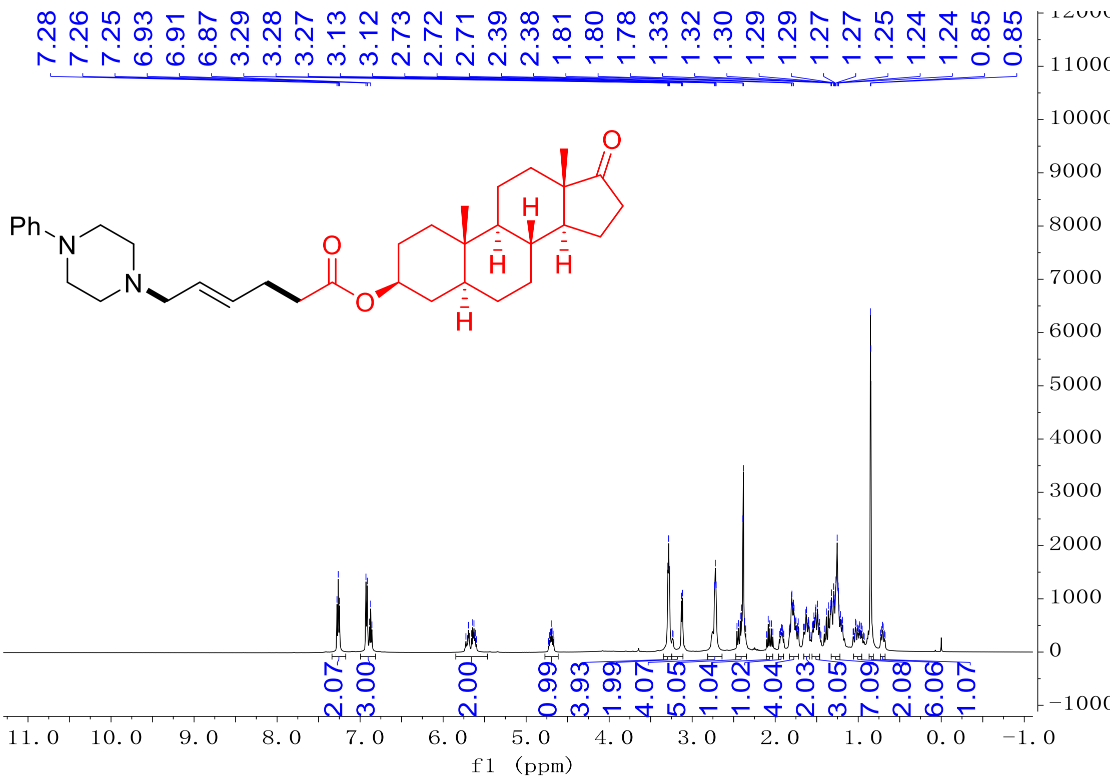

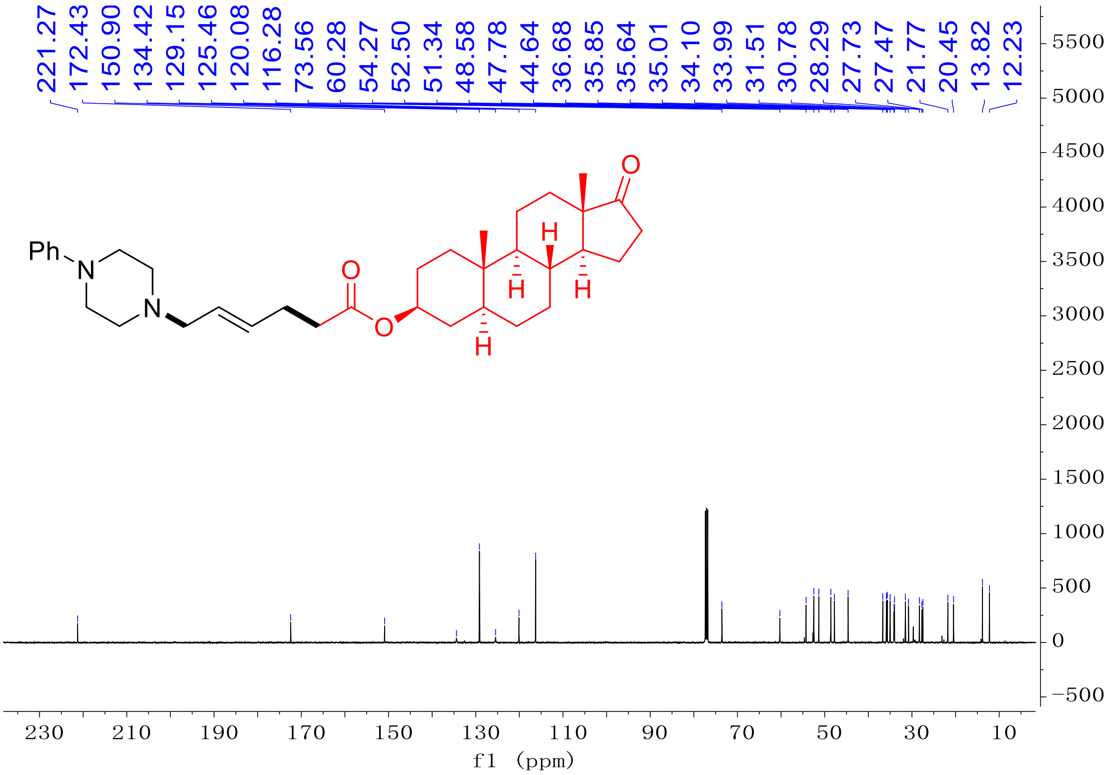

^{1}H NMR (400 MHz, $CDCl_3$) and ^{13}C NMR (101 MHz, $CDCl_3$) spectra for product 4ac

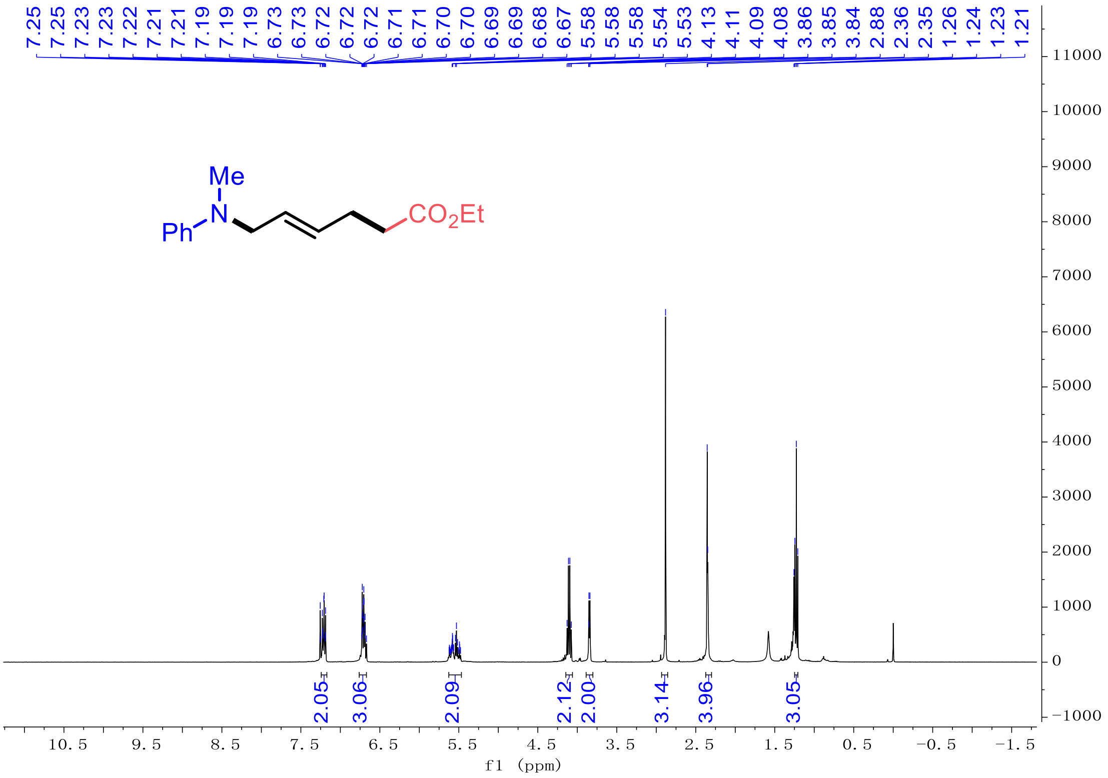

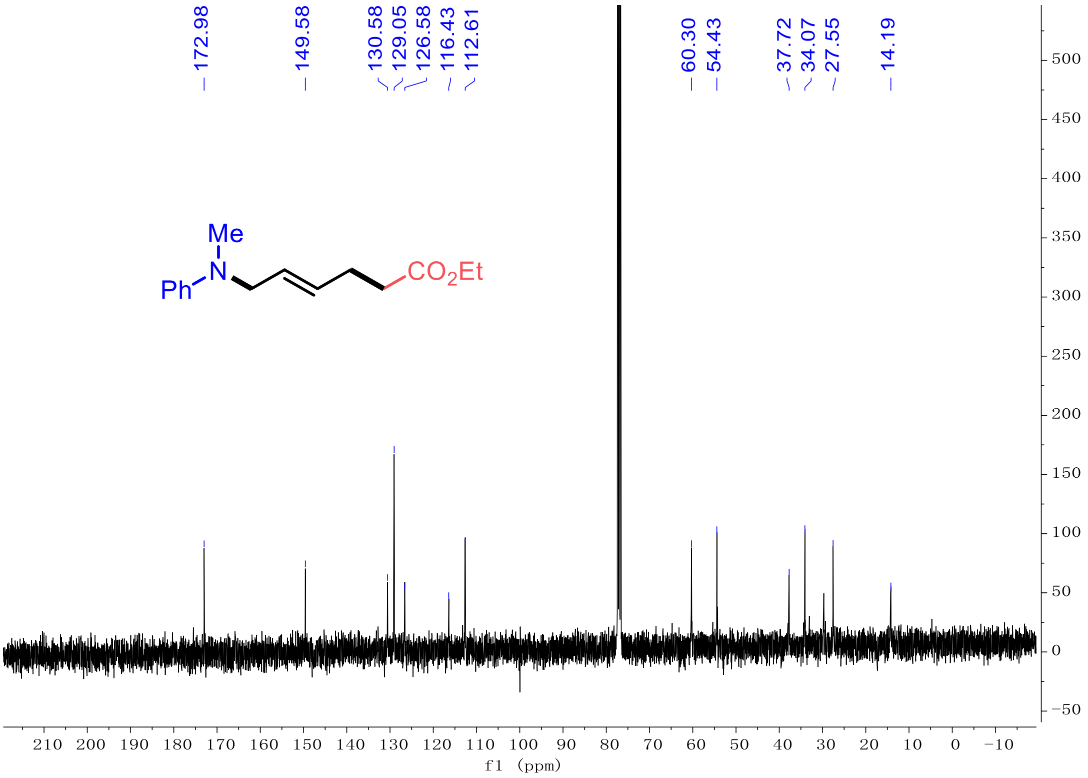

^{1}H NMR (400 MHz, CDCl3) and ^{13}C NMR (101 MHz, CDCl3) spectra for product 4ad

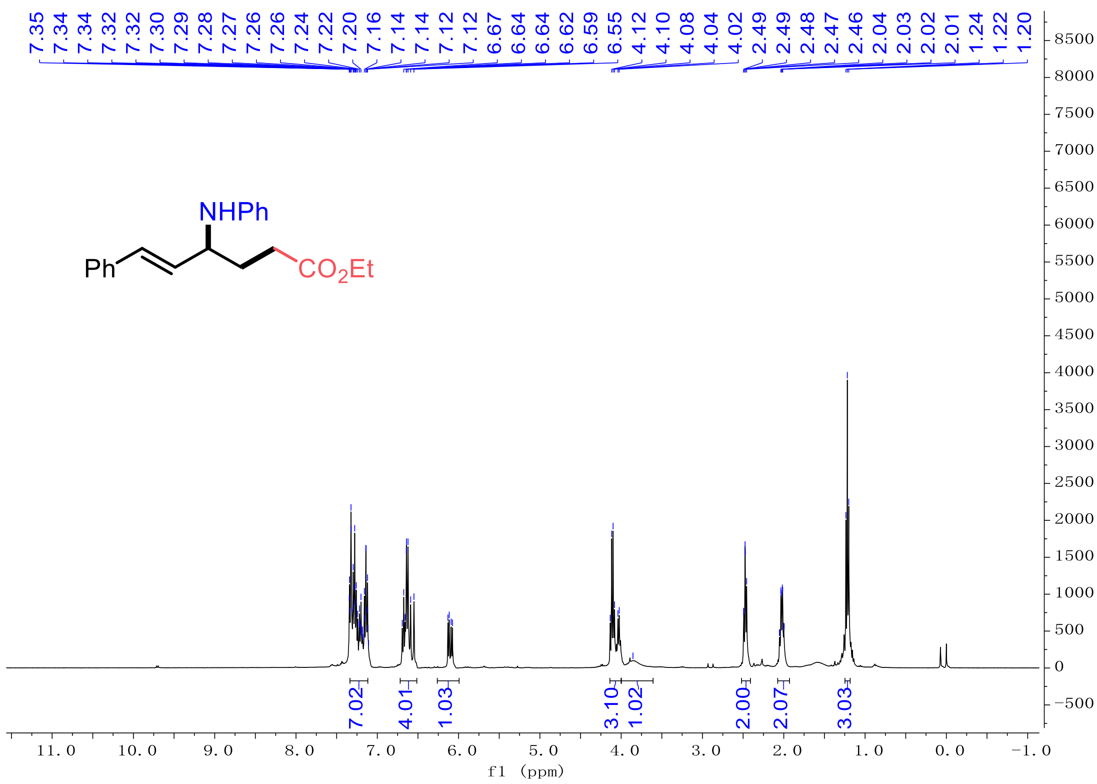

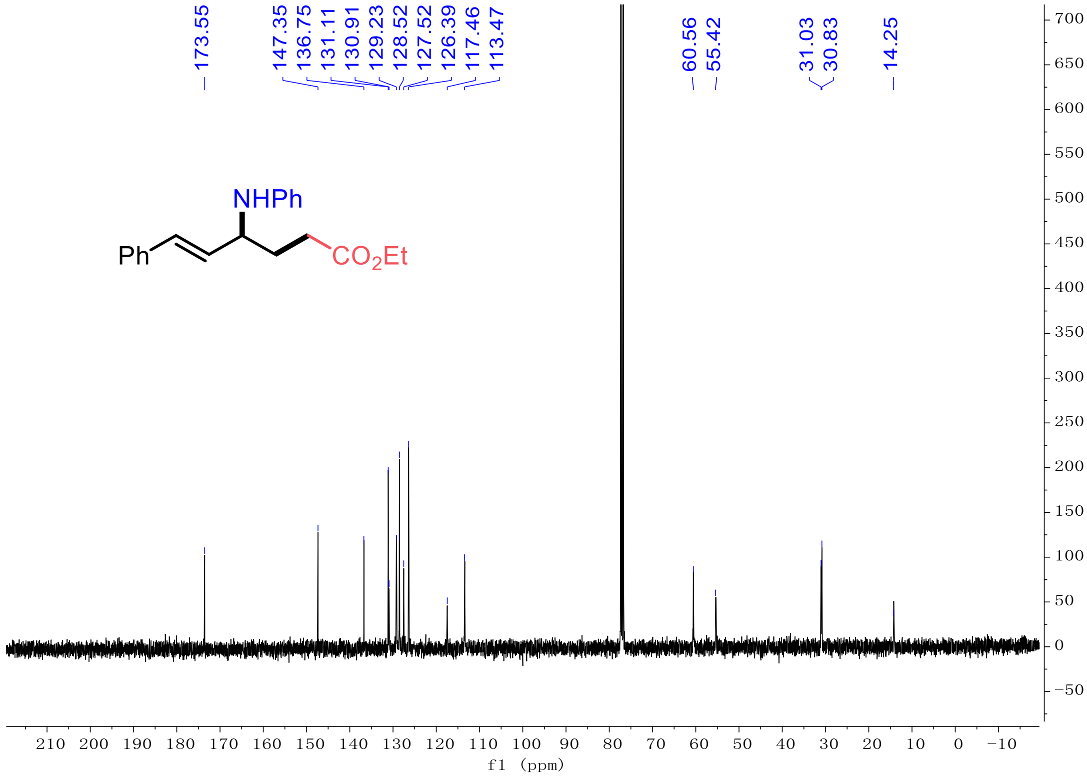

^{1}H NMR (400 MHz, $CDCl_3$) and ^{13}C NMR (101 MHz, $CDCl_3$) spectra for product 4ae

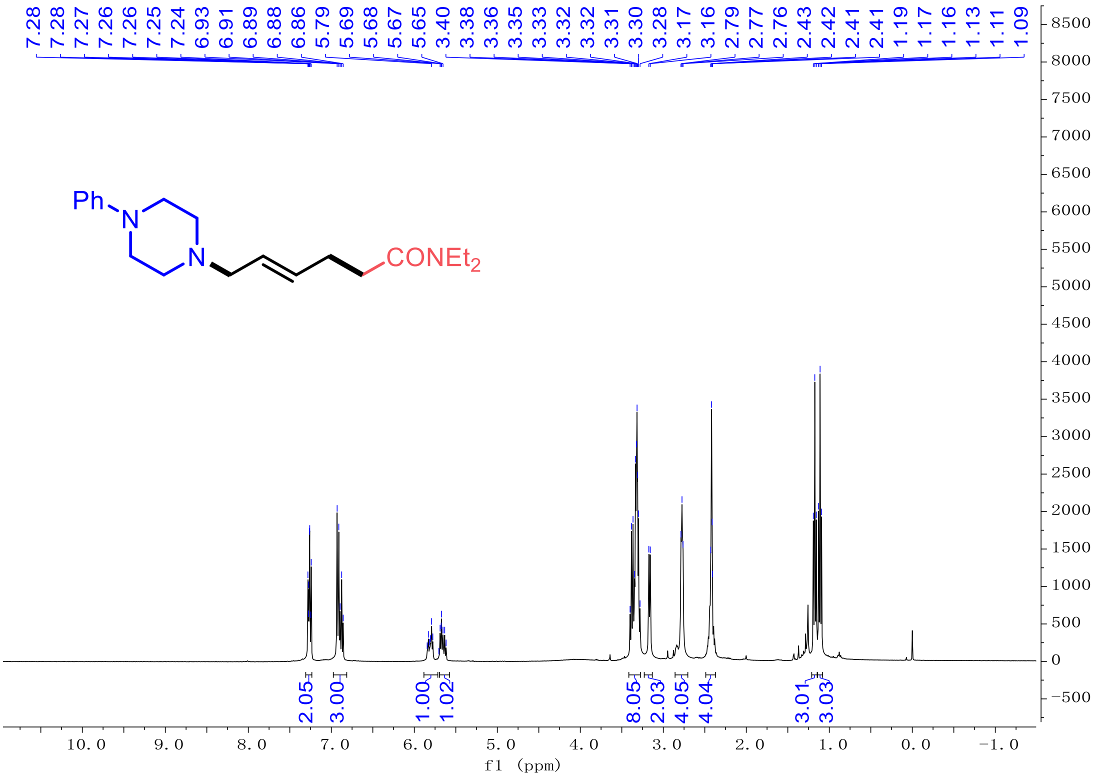

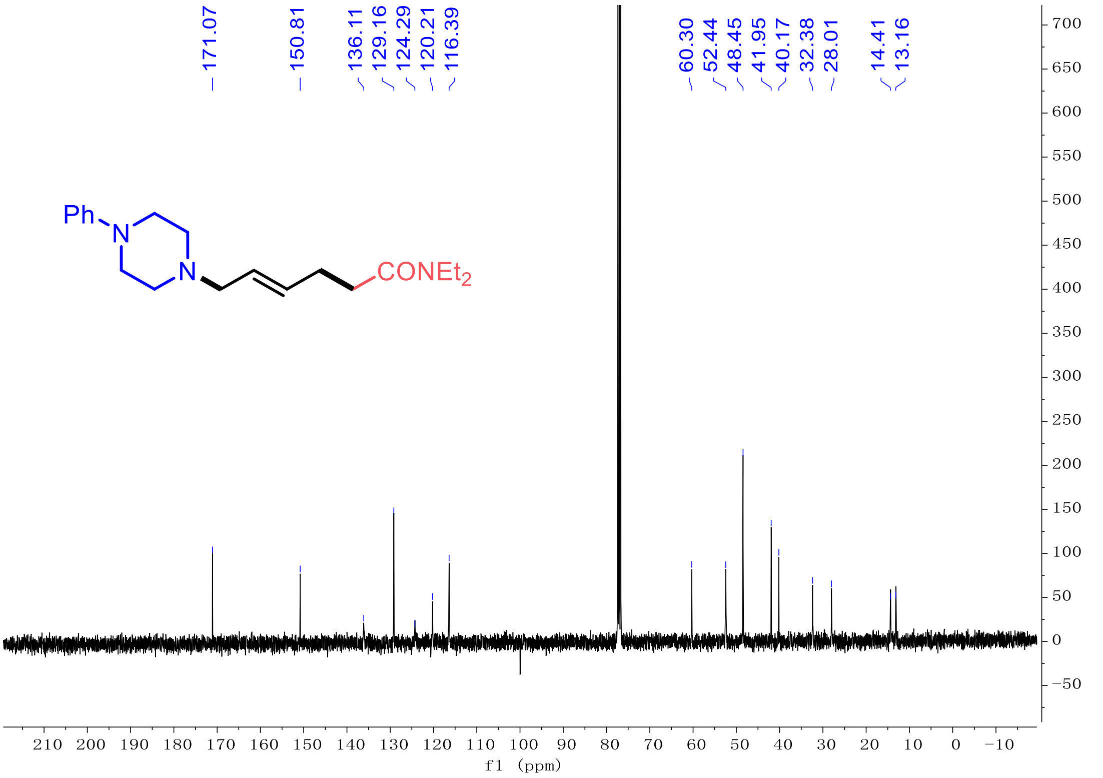

^{1}H NMR (500 MHz, CDCl3) and ^{13}C NMR (126 MHz, CDCl3) spectra for product 6a

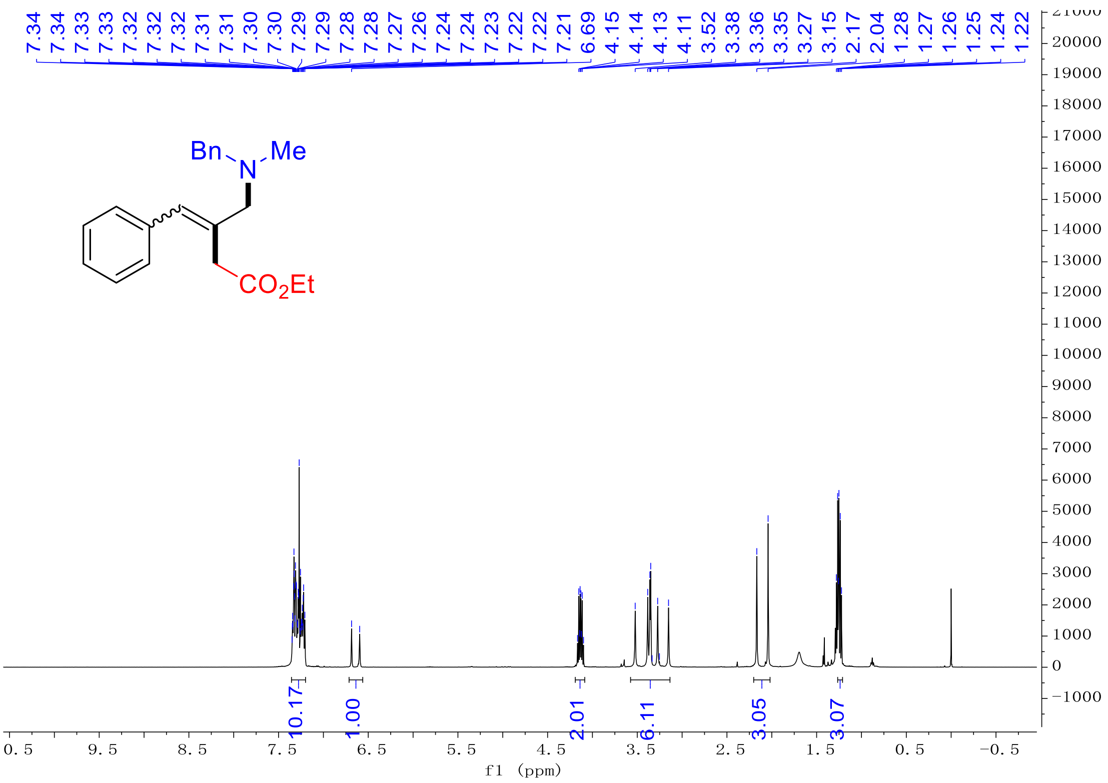

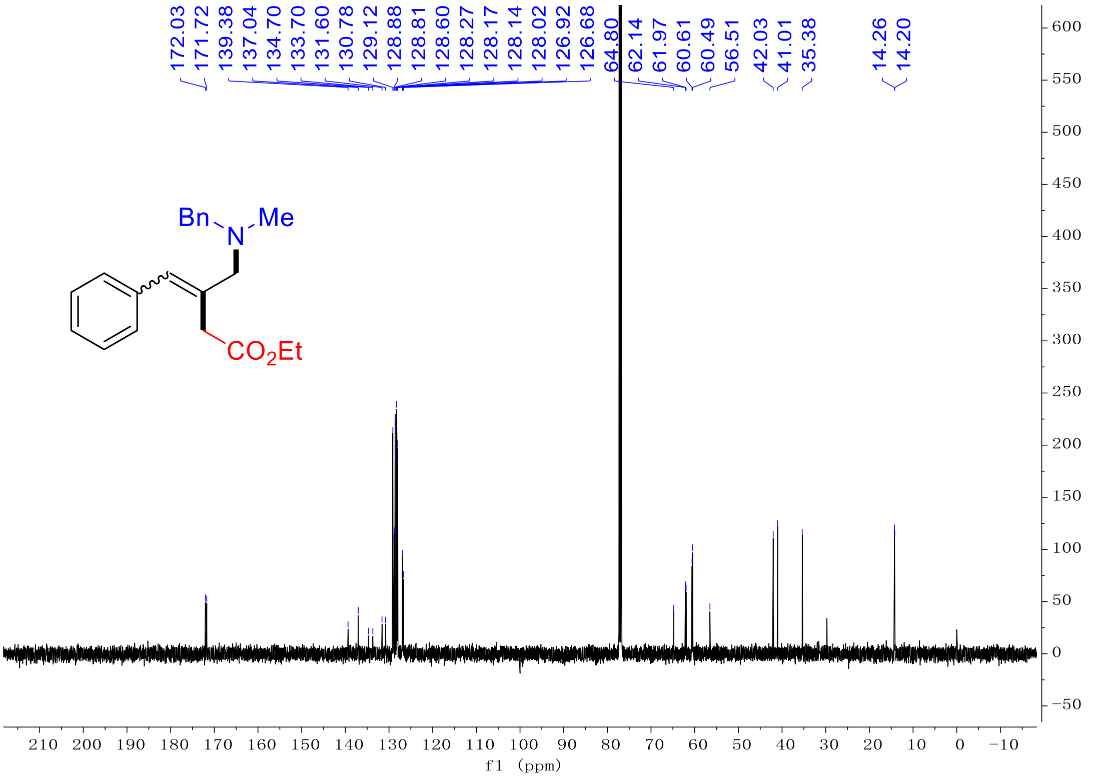

^{1}H NMR (400 MHz, CDCl$_3$) and ^{13}C NMR (101 MHz, CDCl$_3$) spectra for product 6b

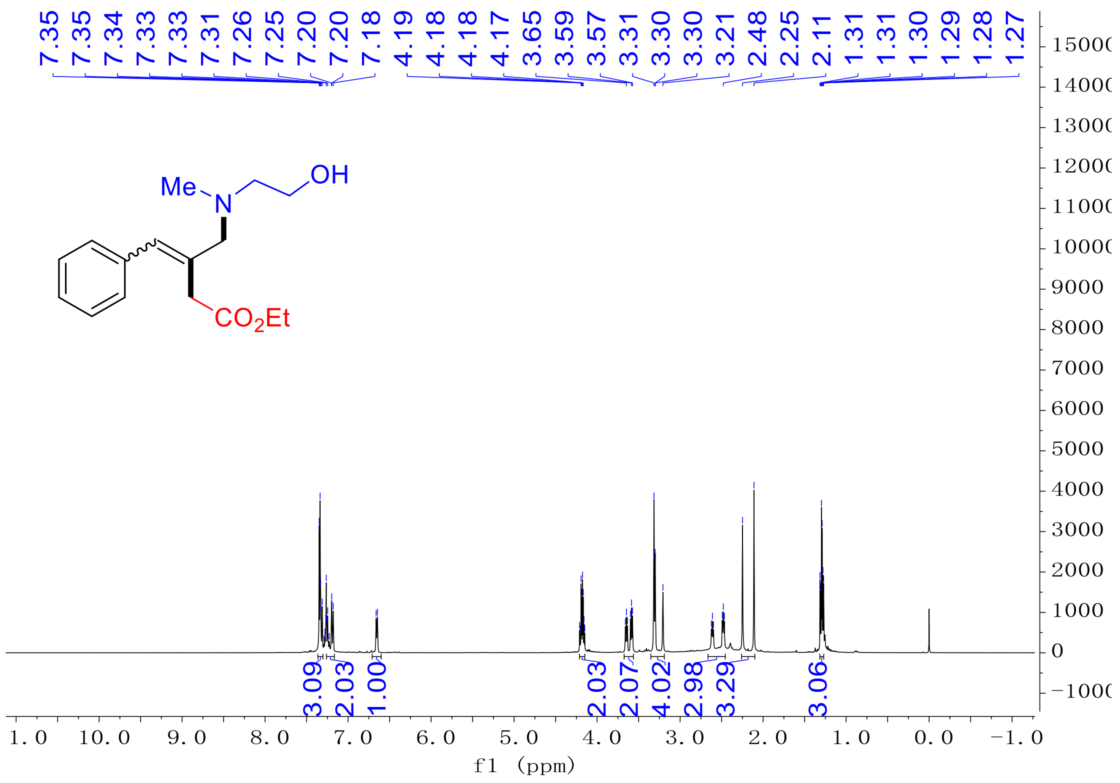

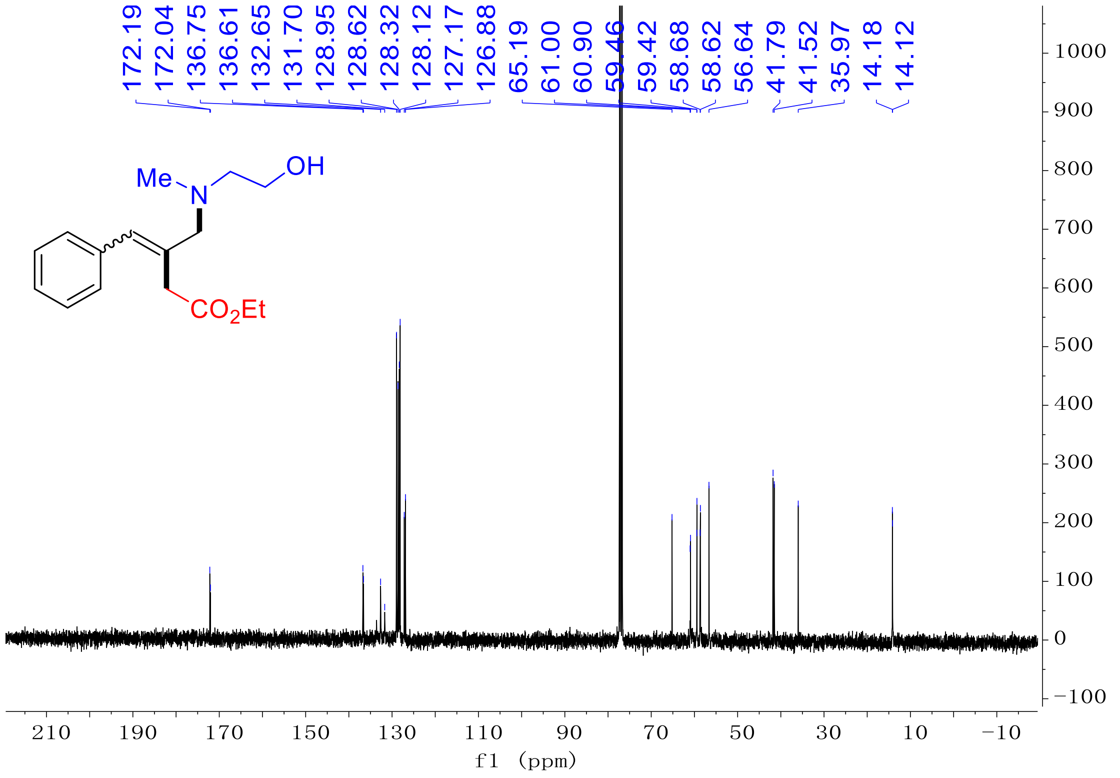

^{1}H NMR (400 MHz, CDCl3) and ^{13}C NMR (126 MHz, CDCl3) spectra for product 6c

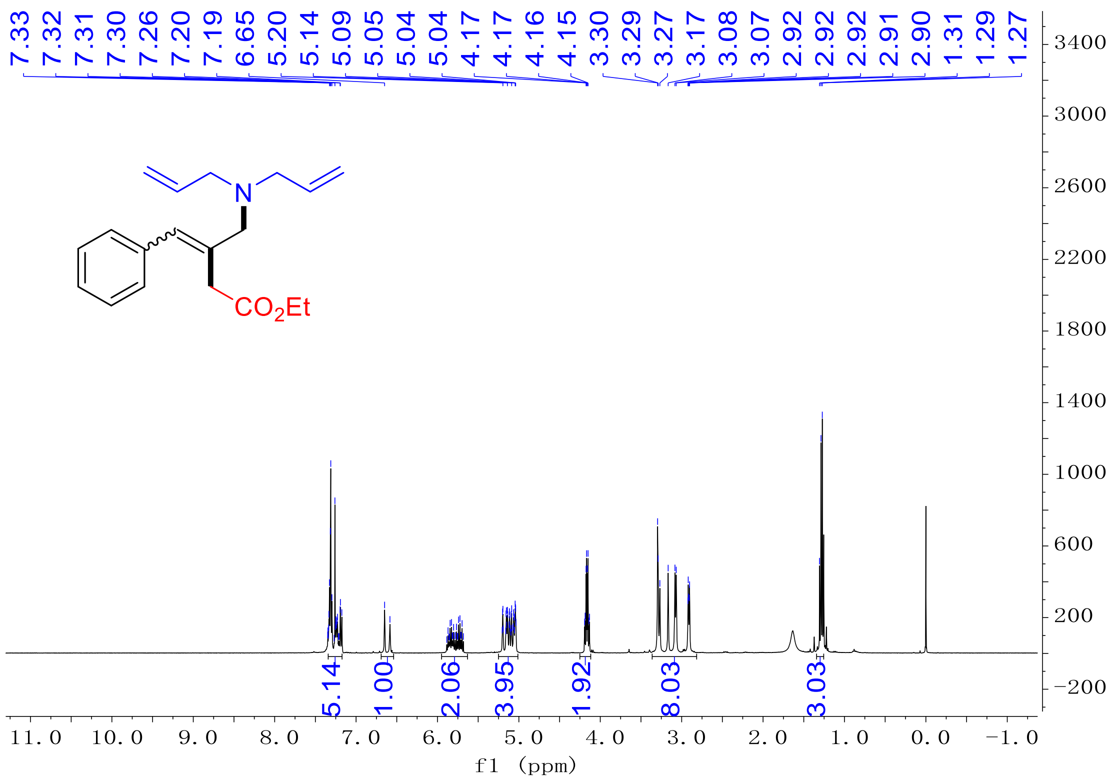

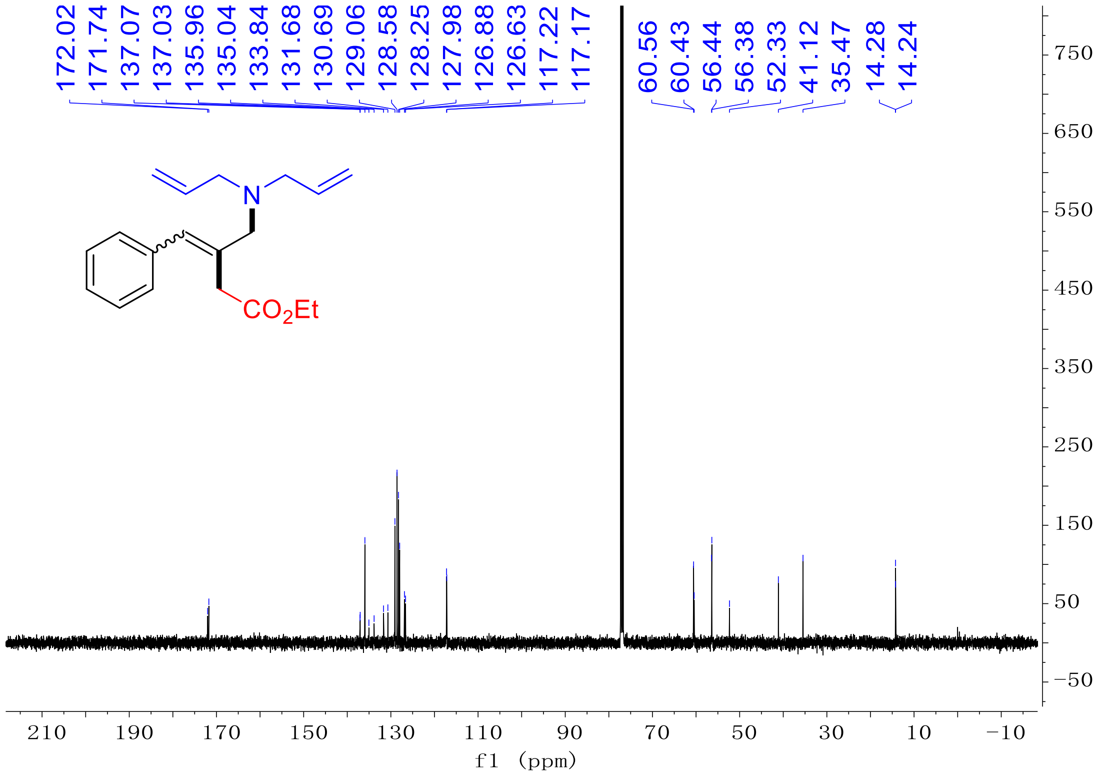

^{1}H NMR (500 MHz, CDCl$_3$) and ^{13}C NMR (126 MHz, CDCl$_3$) spectra for product 6d

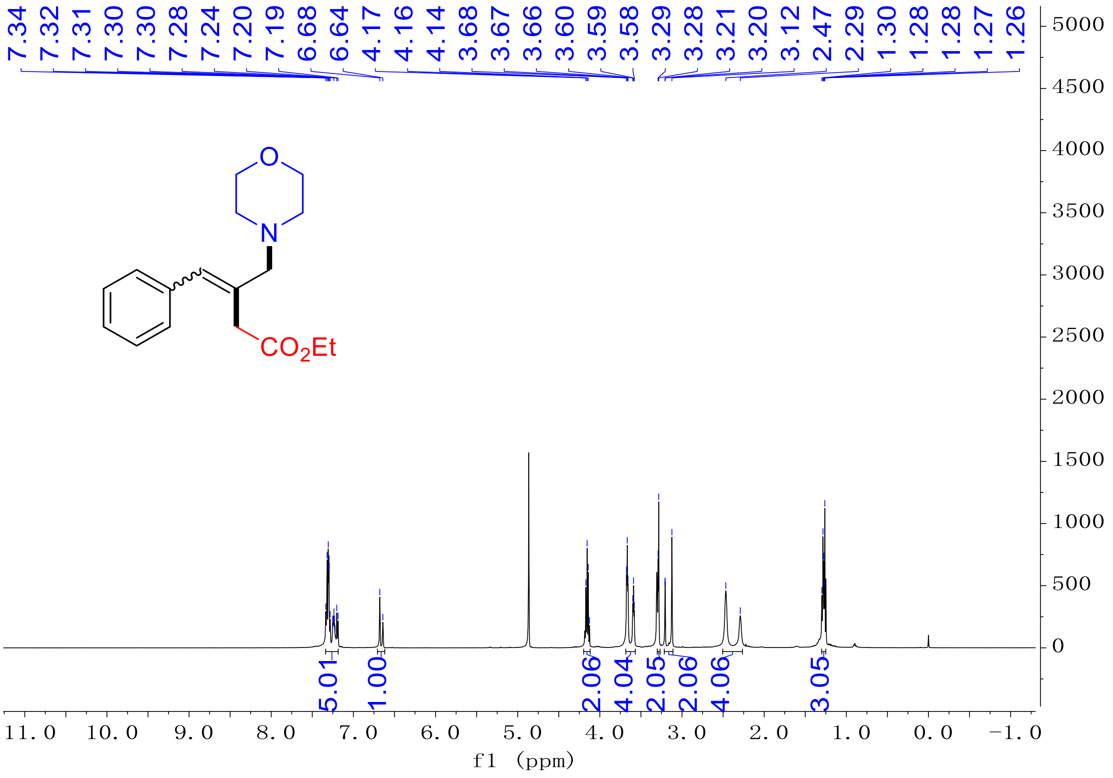

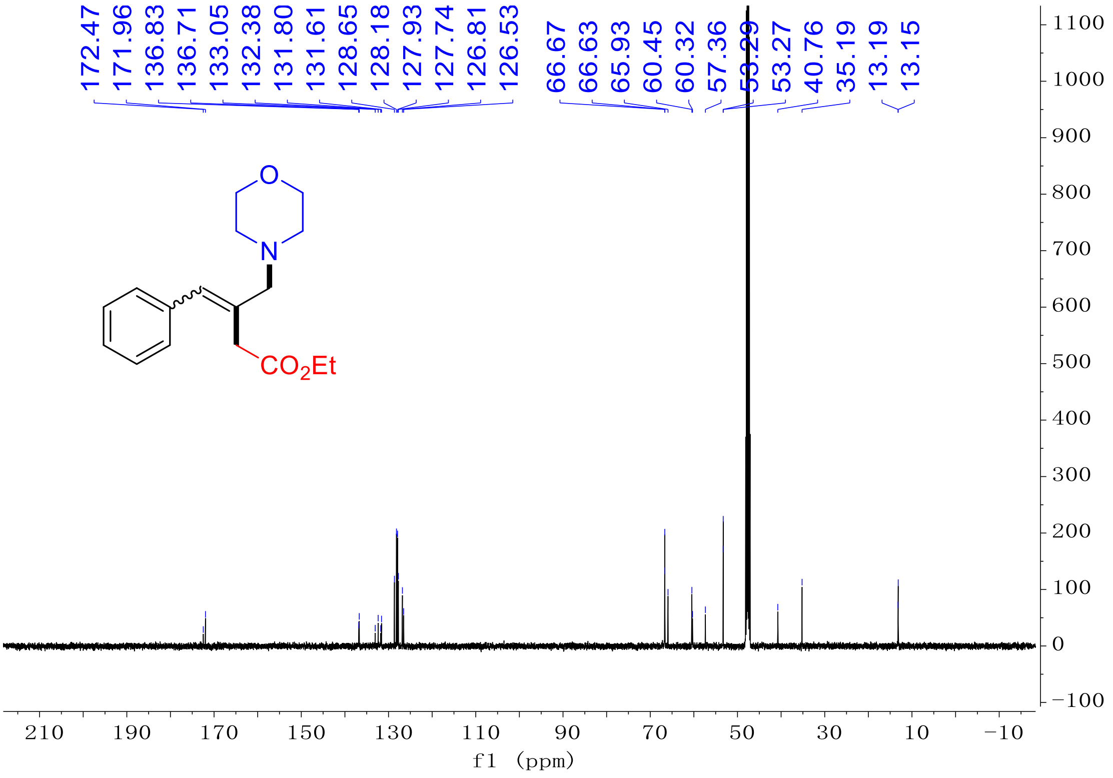

^{1}H NMR (500 MHz, $CDCl_3$) and ^{13}C NMR (101 MHz, $CDCl_3$) spectra for product 6e

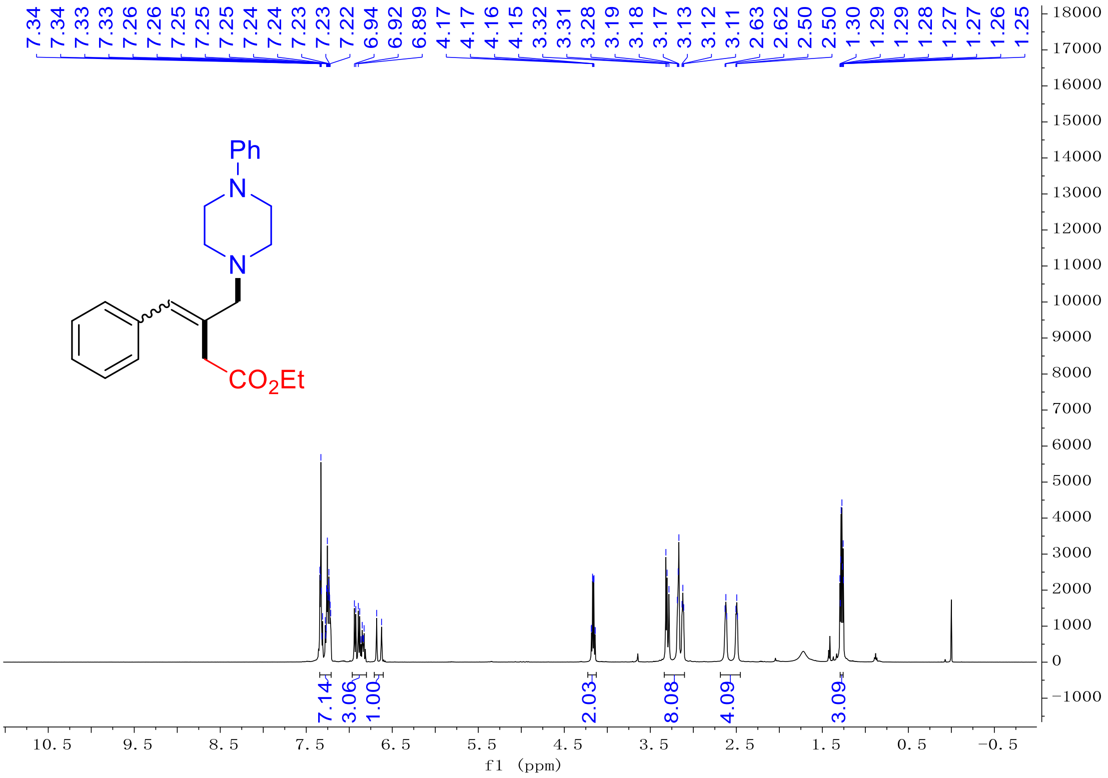

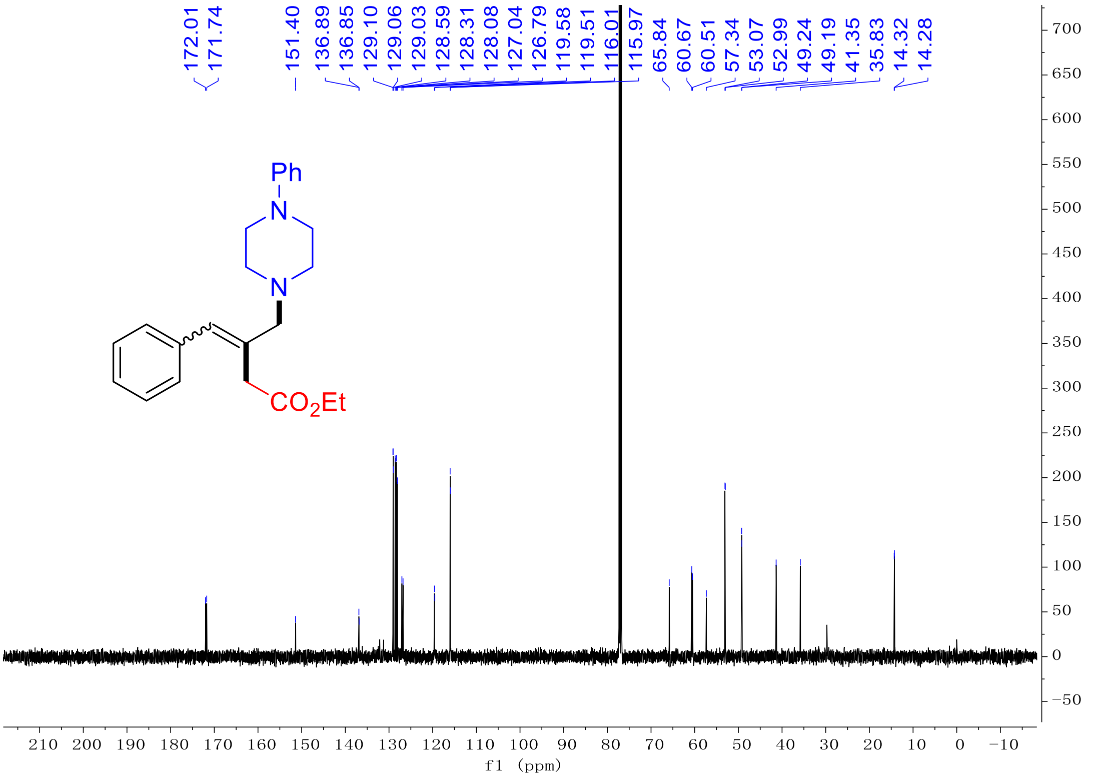

^{1}H NMR (400 MHz, CDCl3) and ^{13}C NMR (126 MHz, CDCl3) spectra for product 6f

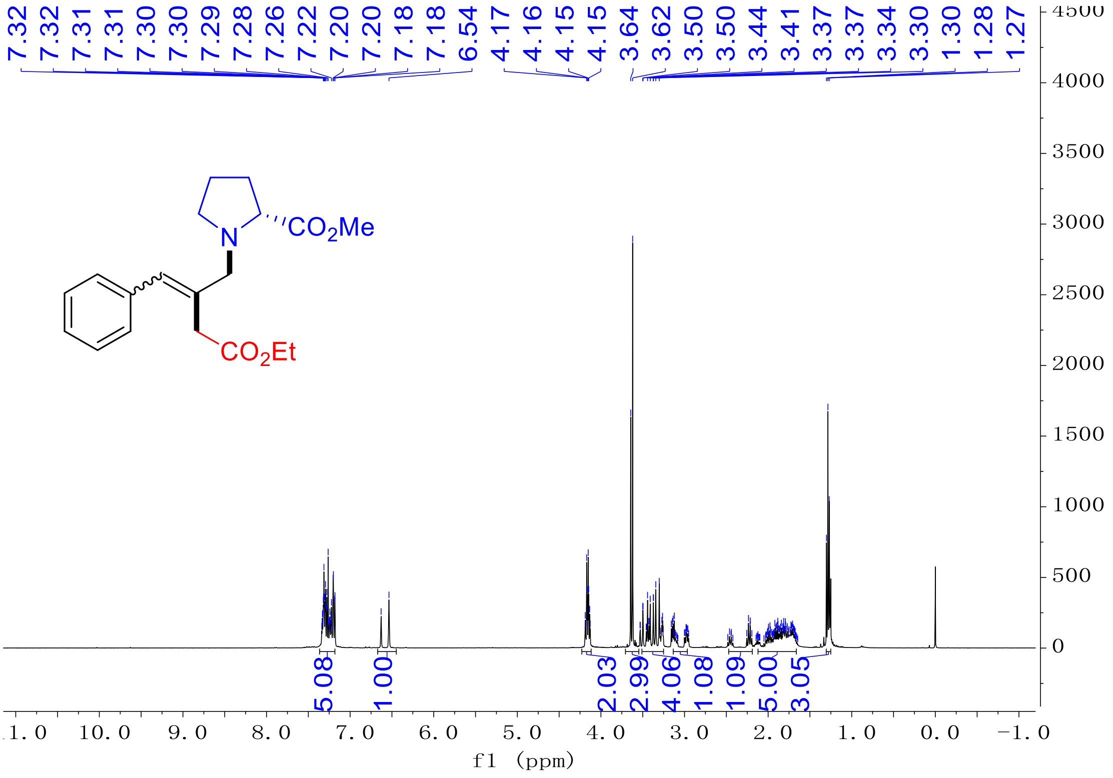

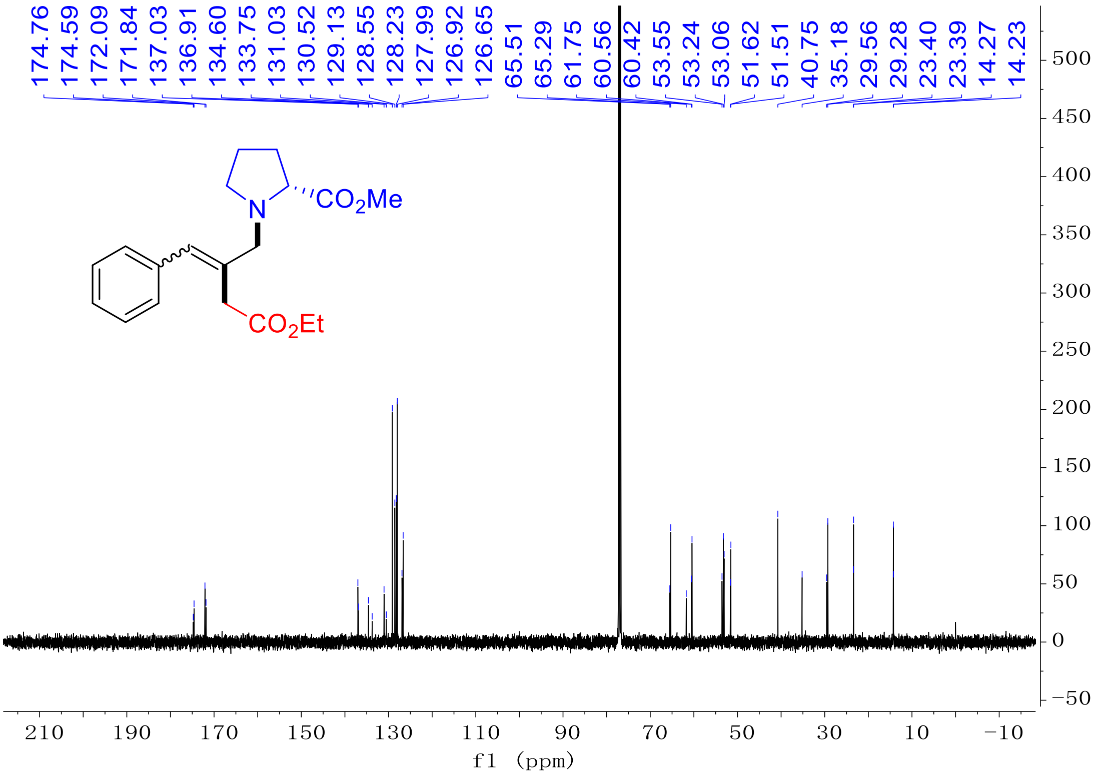

^{1}H NMR (400 MHz, $CDCl_3$) and ^{13}C NMR (101 MHz, $CDCl_3$) spectra for product 6g

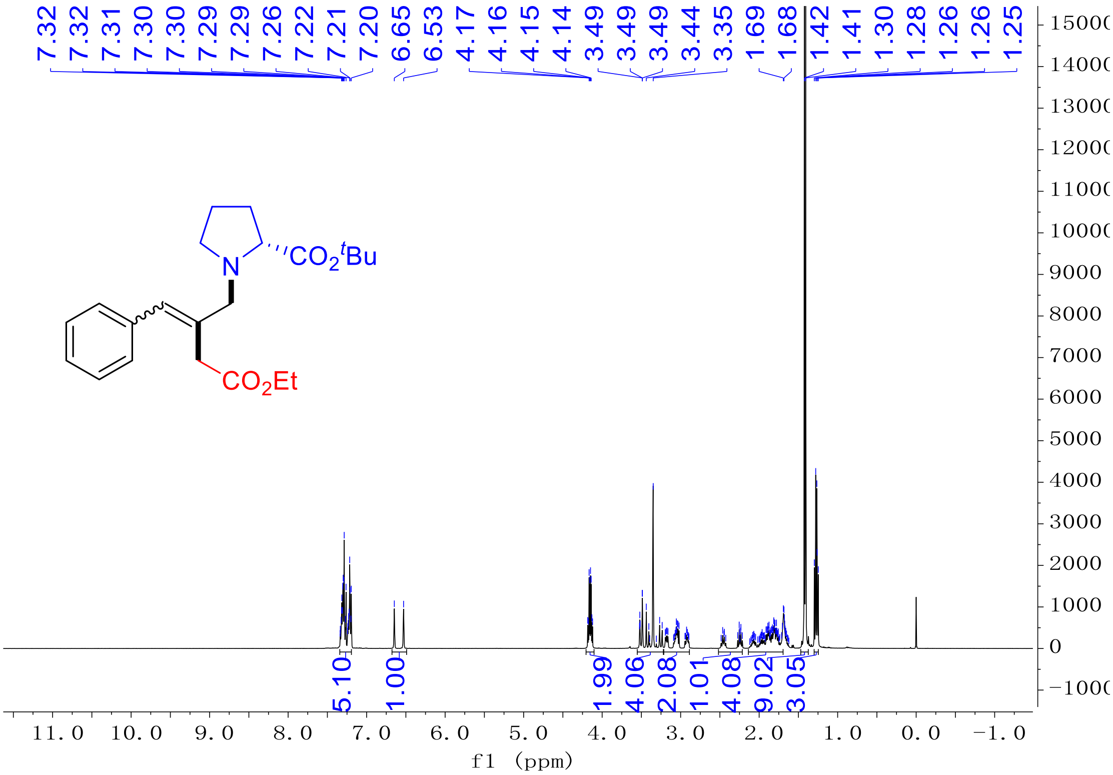

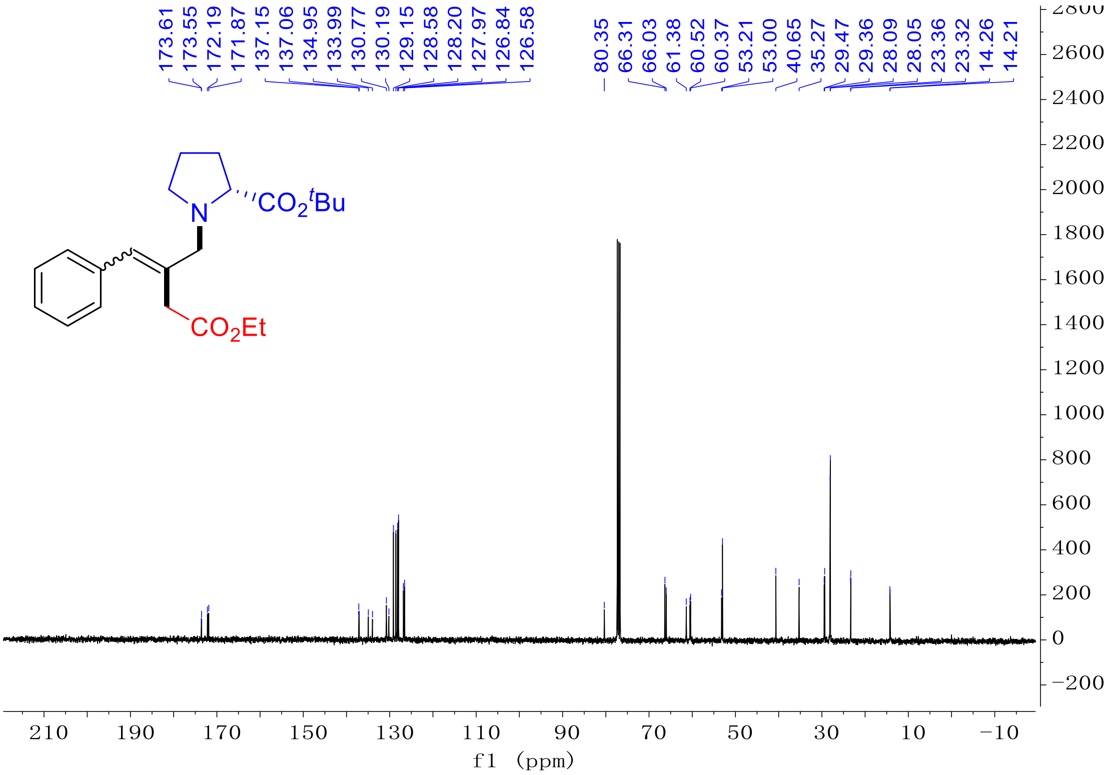

^{1}H NMR (400 MHz, CDCl3) and ^{13}C NMR (101 MHz, CDCl3) spectra for product 6h

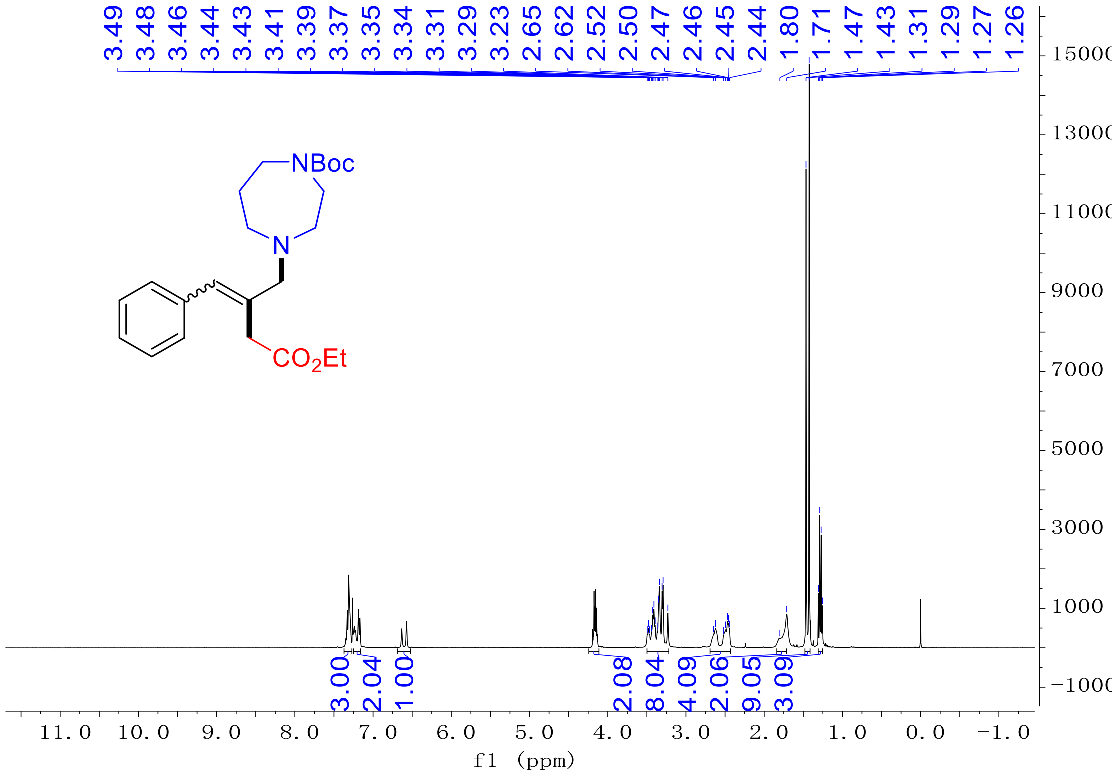

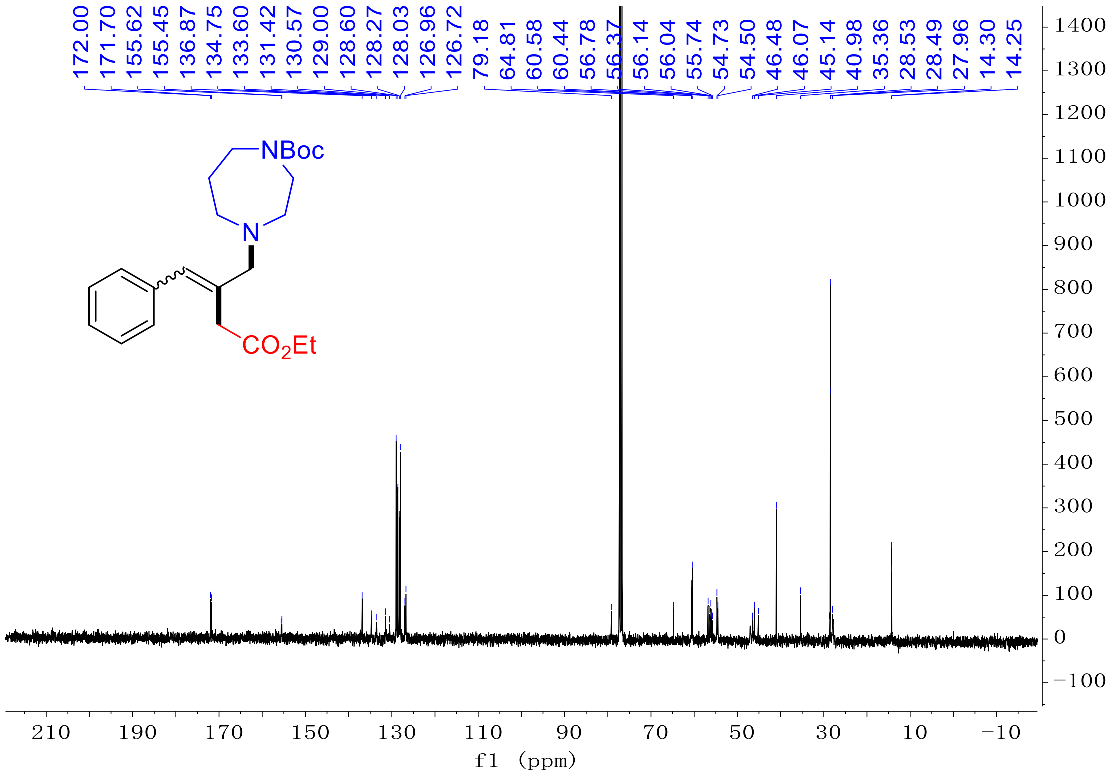

^{1}H NMR (500 MHz, $CDCl_3$) and ^{13}C NMR (126 MHz, $CDCl_3$) spectra for product 6i

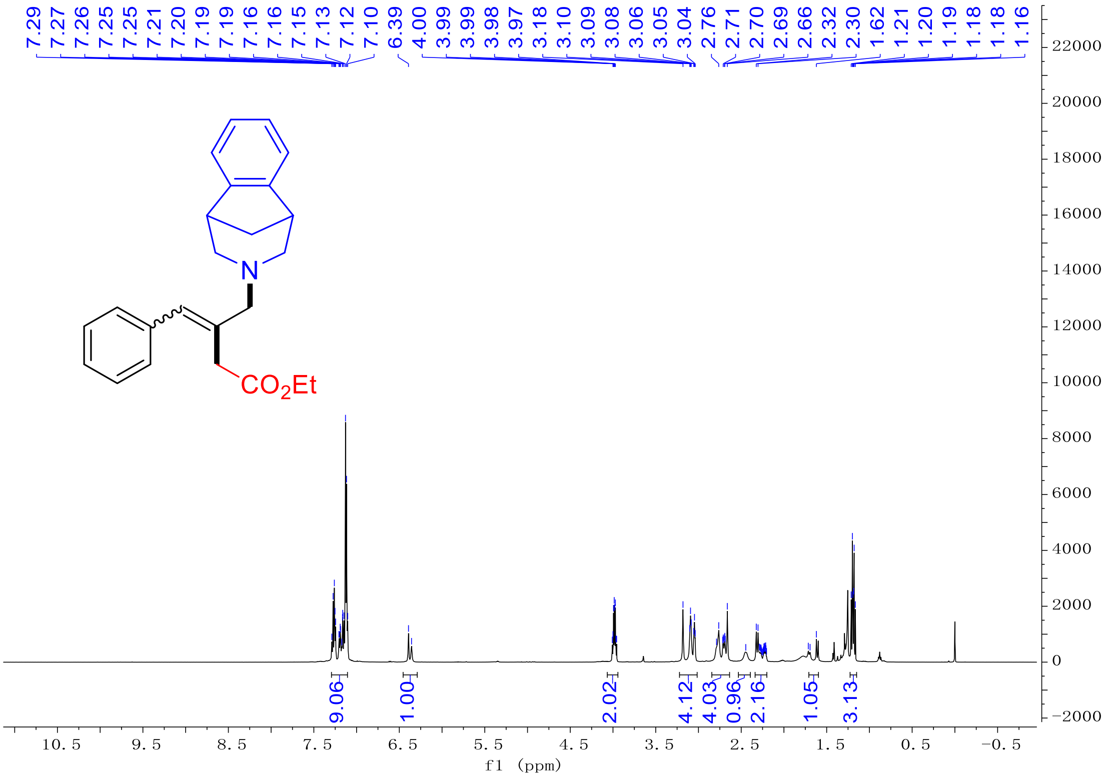

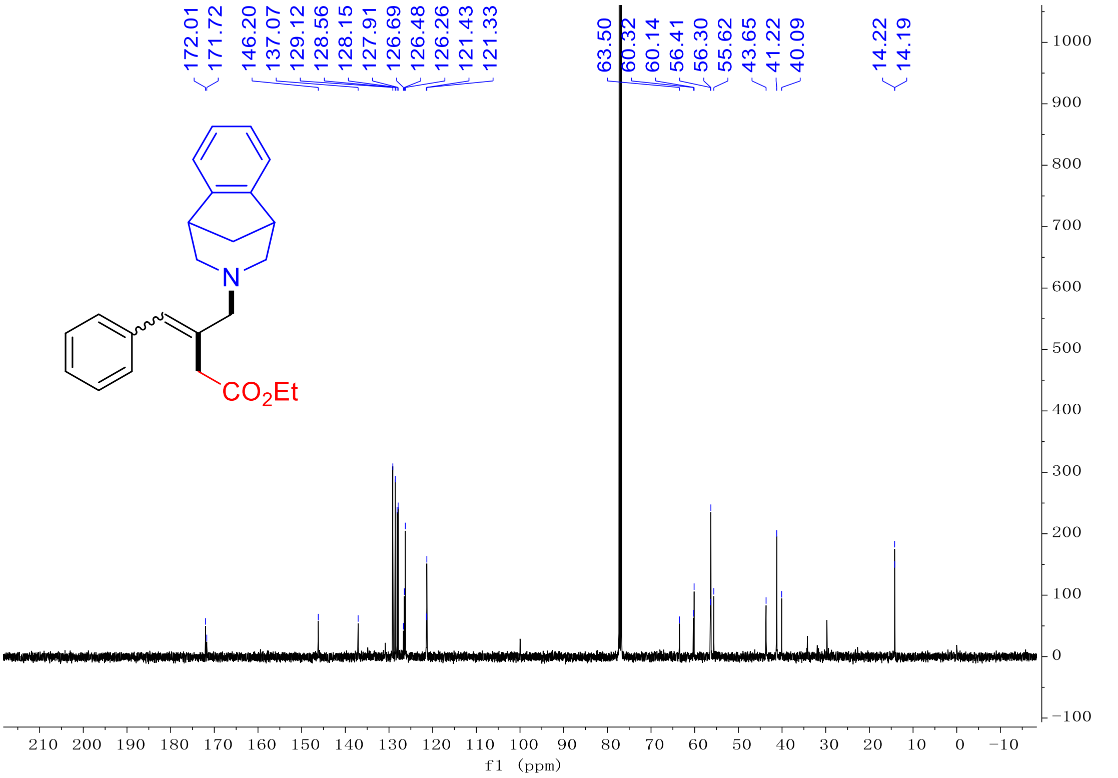

^{1}H NMR (400 MHz, $CDCl_3$) and ^{13}C NMR (101 MHz, $CDCl_3$) spectra for product *Z*-6i

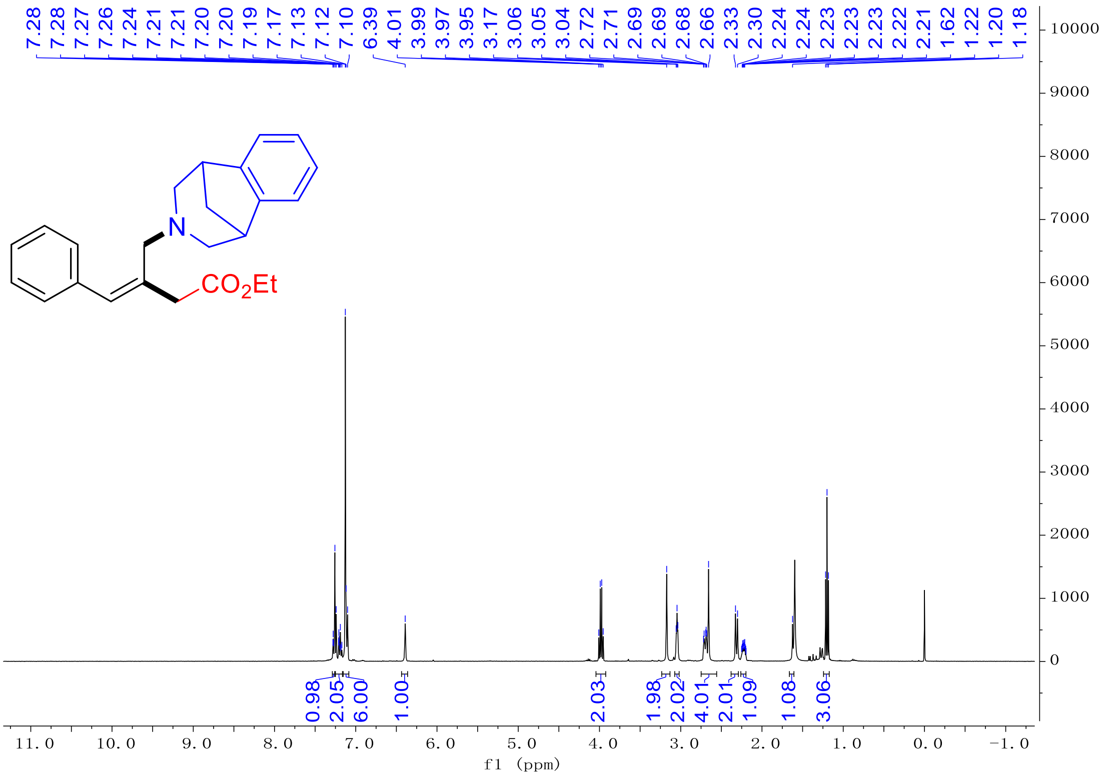

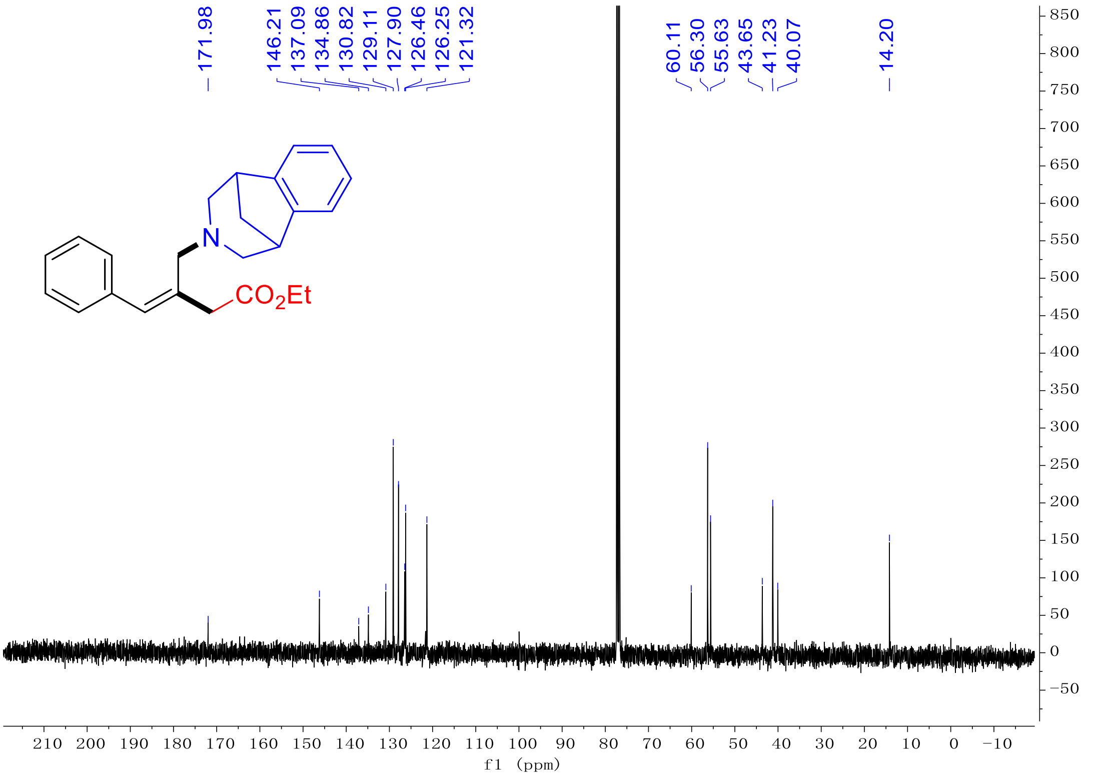

^{1}H NMR (500 MHz, CDCl$_3$) and ^{13}C NMR (126 MHz, CDCl$_3$) spectra for product 6j

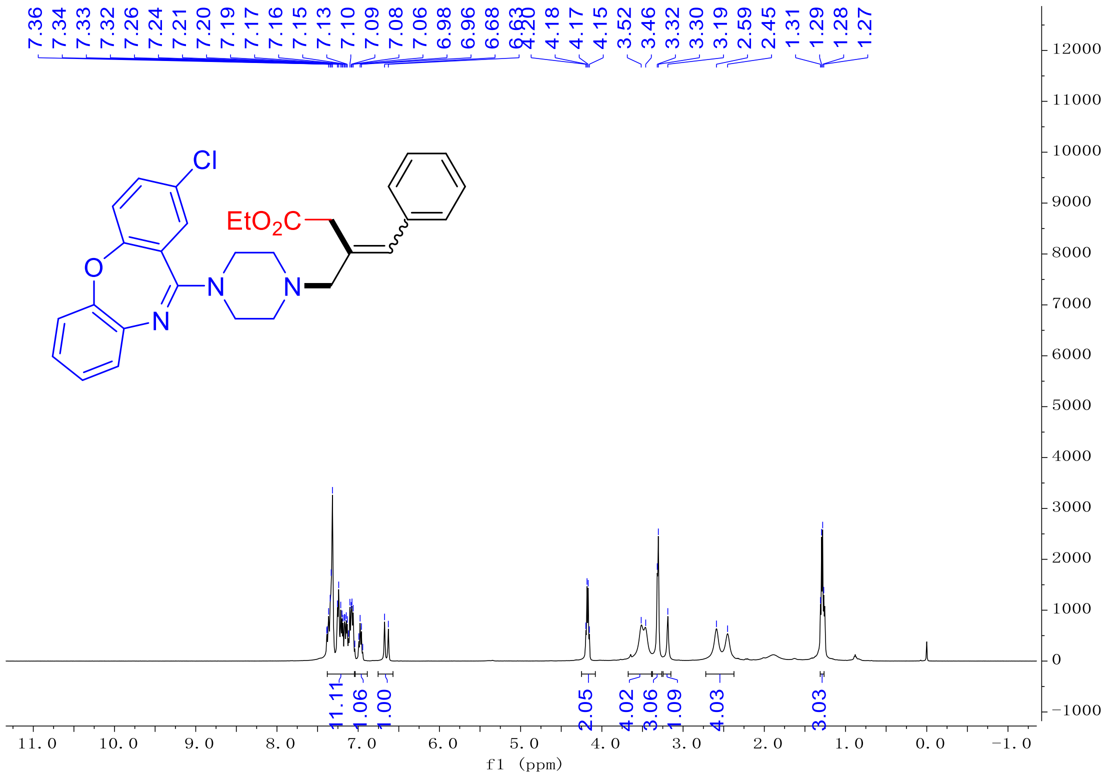

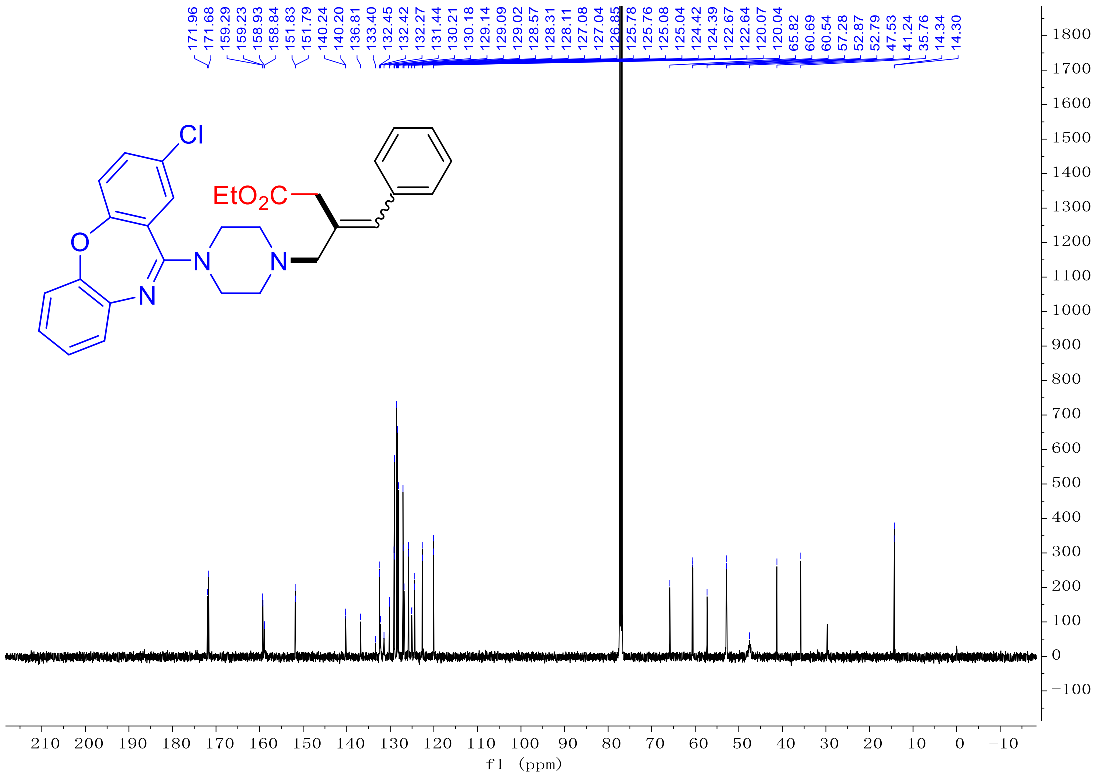

^{1}H NMR (400 MHz, CDCl3) and ^{13}C NMR (126 MHz, CDCl3) spectra for product 6k

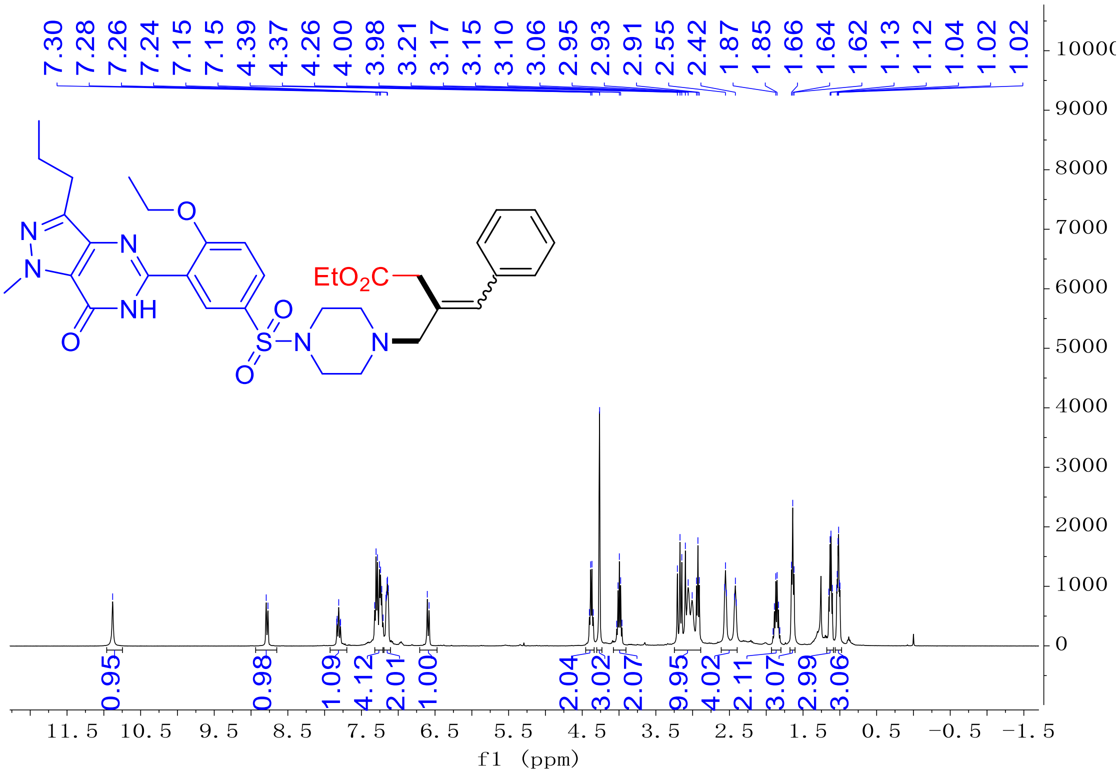

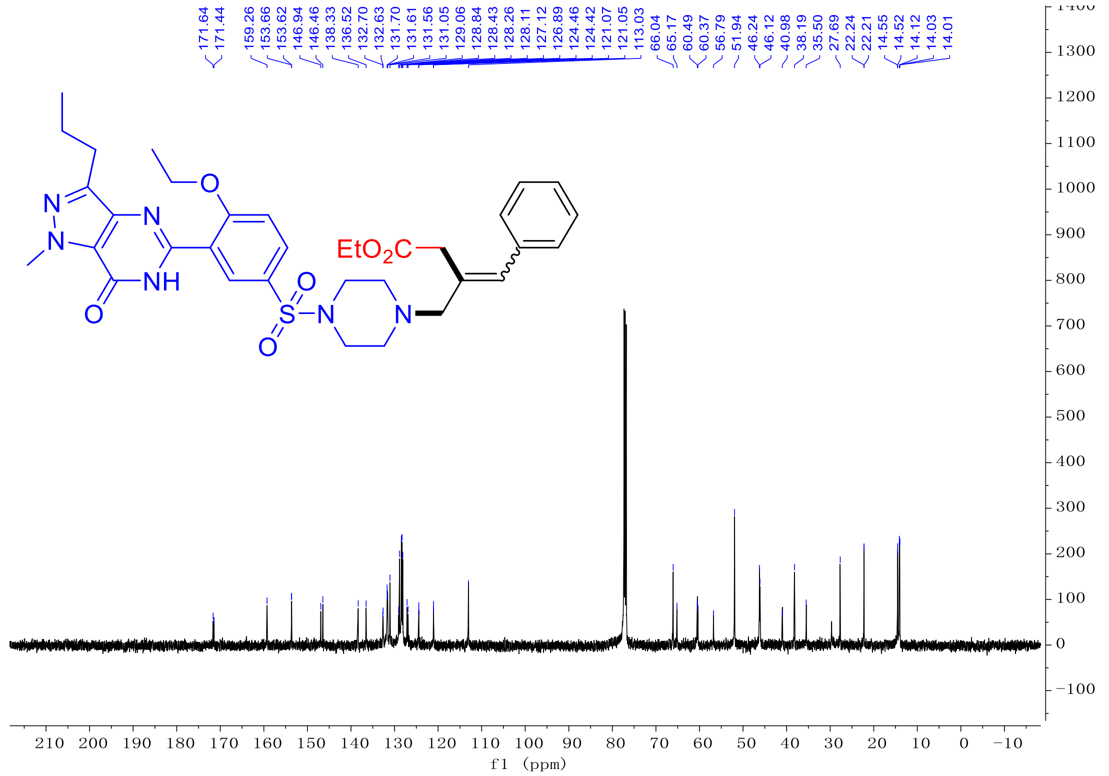

^{1}H NMR (400 MHz, $CDCl_3$) and ^{13}C NMR (101 MHz, $CDCl_3$) spectra for product 6l

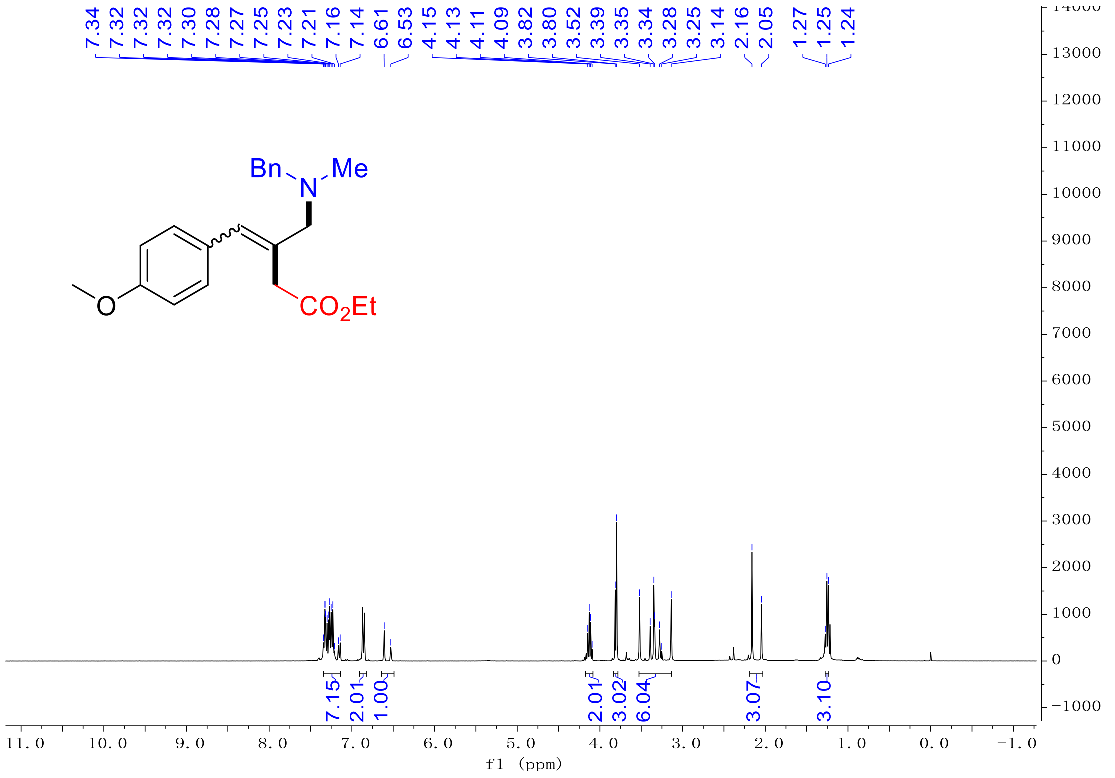

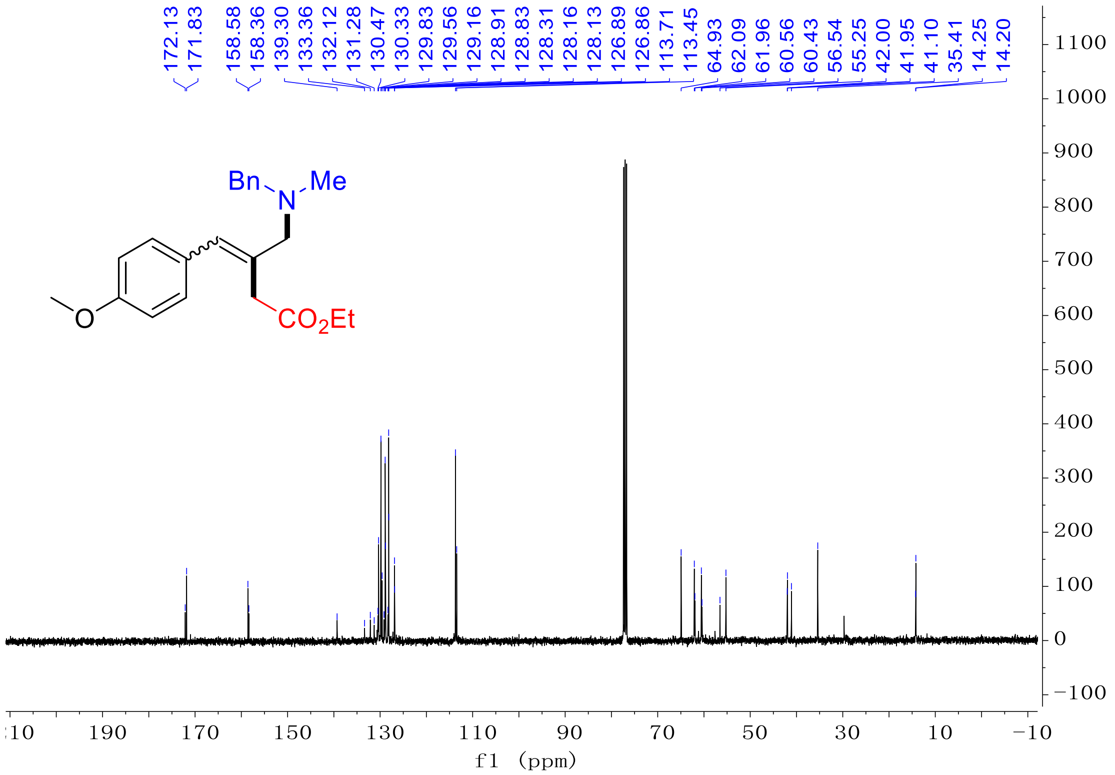

^{1}H NMR (500 MHz, $CDCl_3$) and ^{13}C NMR (126 MHz, $CDCl_3$) spectra for product 6m

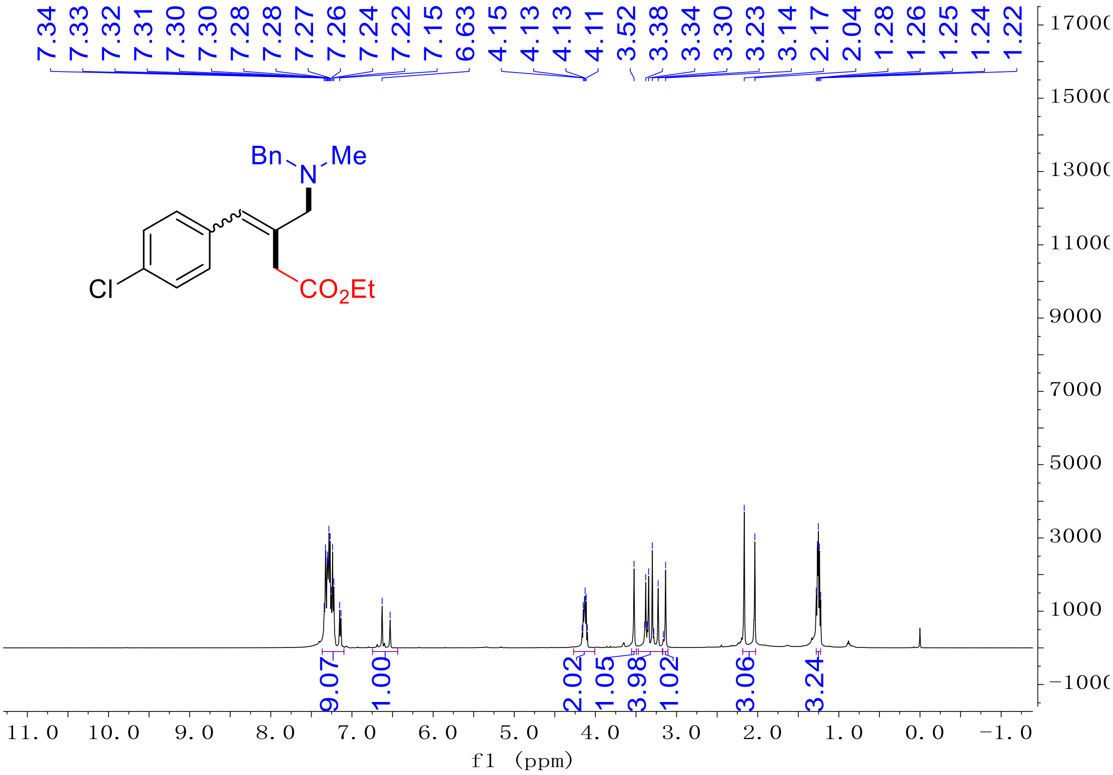

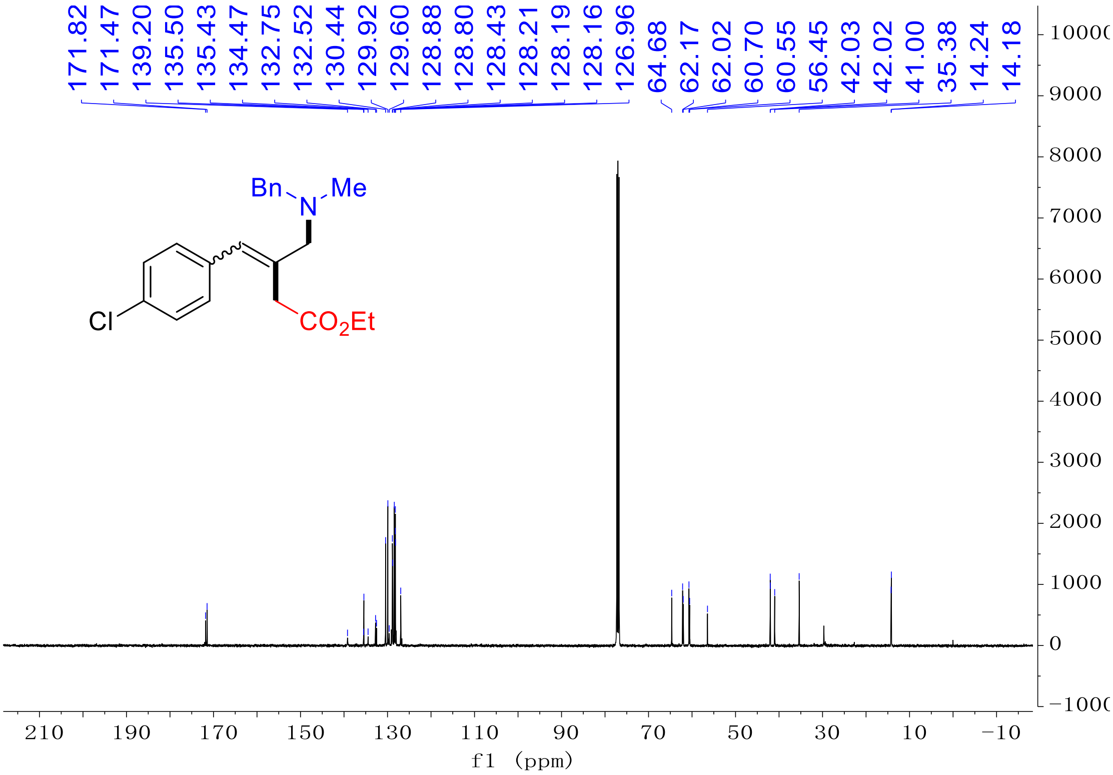

^{1}H NMR (500 MHz, CDCl3) and ^{13}C NMR (126 MHz, CDCl3) spectra for product 6n

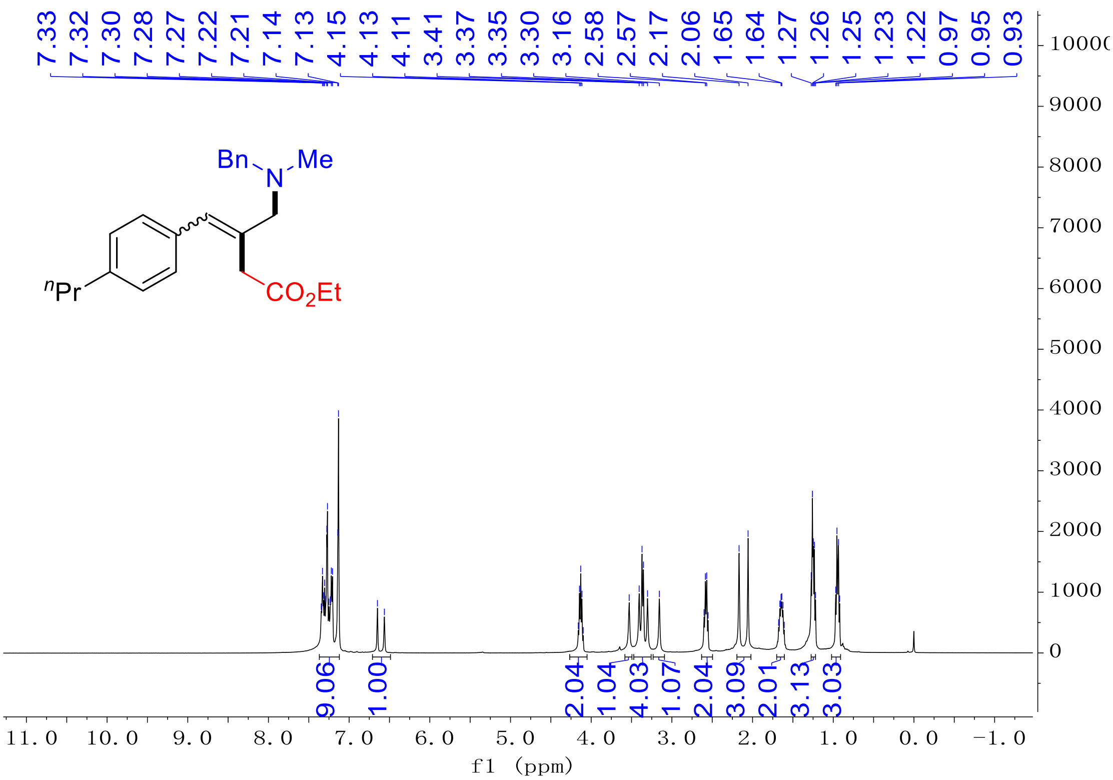

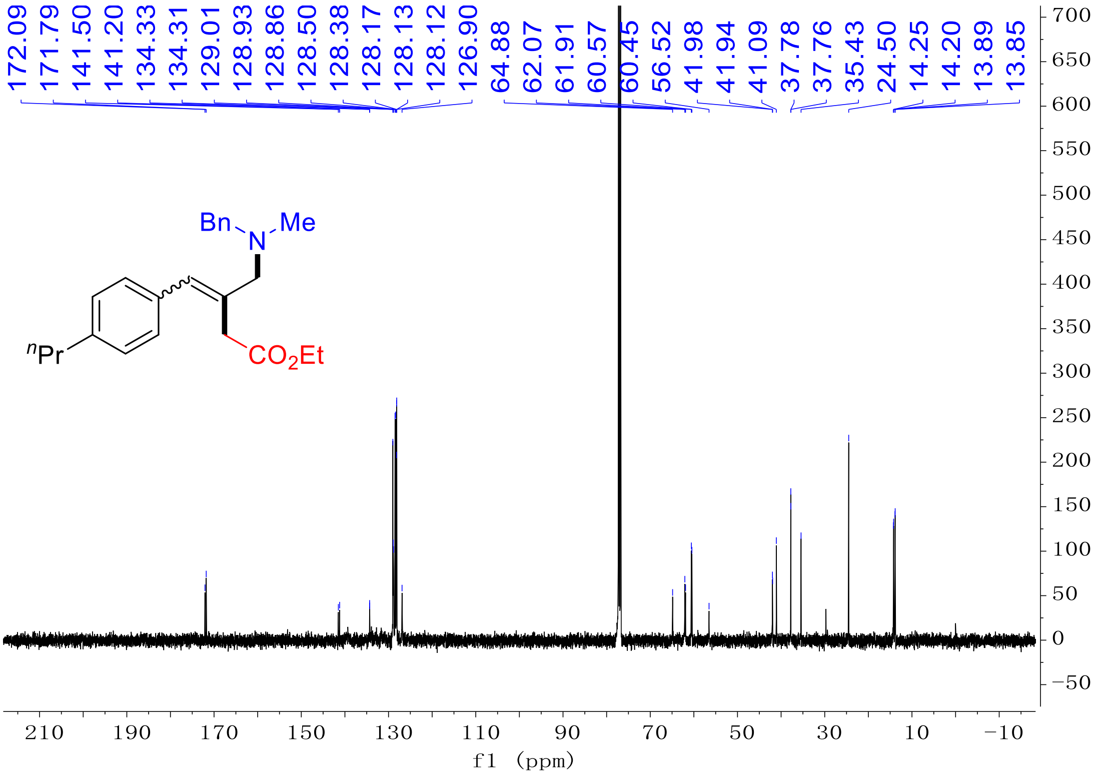

^{1}H NMR (400 MHz, $CDCl_3$) , ^{13}C NMR (101 MHz, $CDCl_3$) and ^{19}F NMR (376 MHz, $CDCl_3$) spectra for product 6o

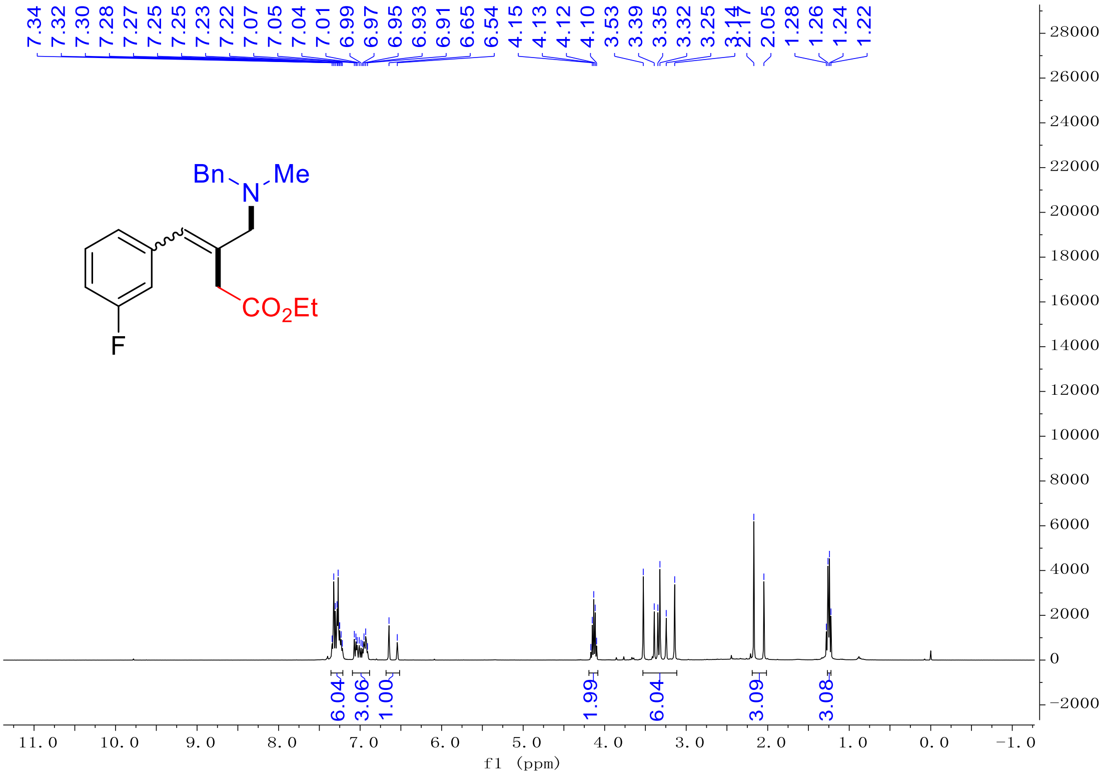

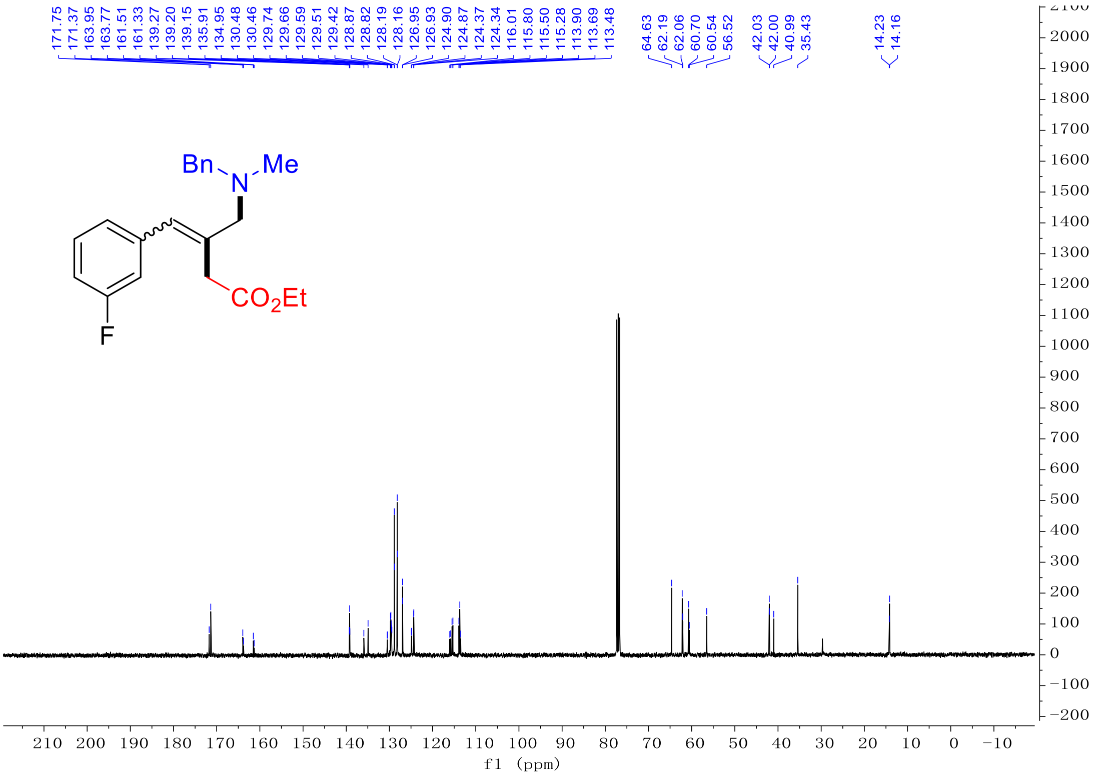

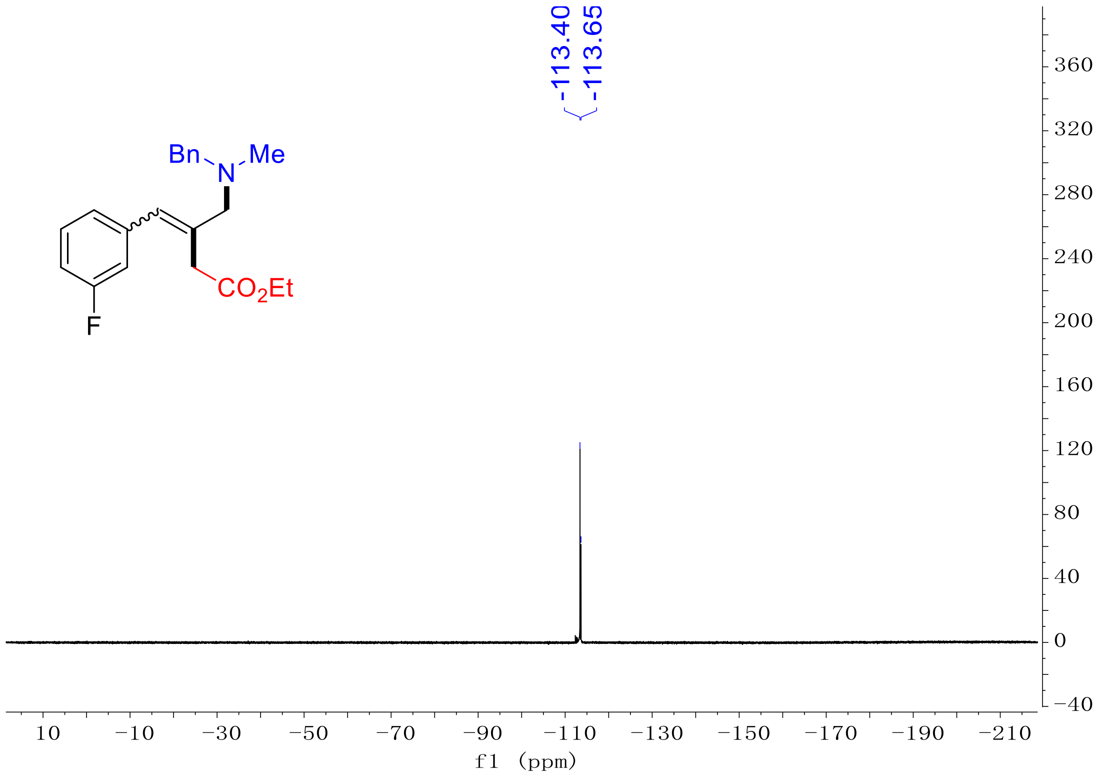
-113.40
-113.65
Bn
N
Me
CO2Et
F
360
320
280
240
200
160
120
80
40
0
−40
10
−10
−30
−50
−70
−90
−110
−130
−150
−170
−190
−210
f1 (ppm)

^{1}H NMR (500 MHz, $CDCl_3$) and ^{13}C NMR (126 MHz, $CDCl_3$) spectra for product 6p

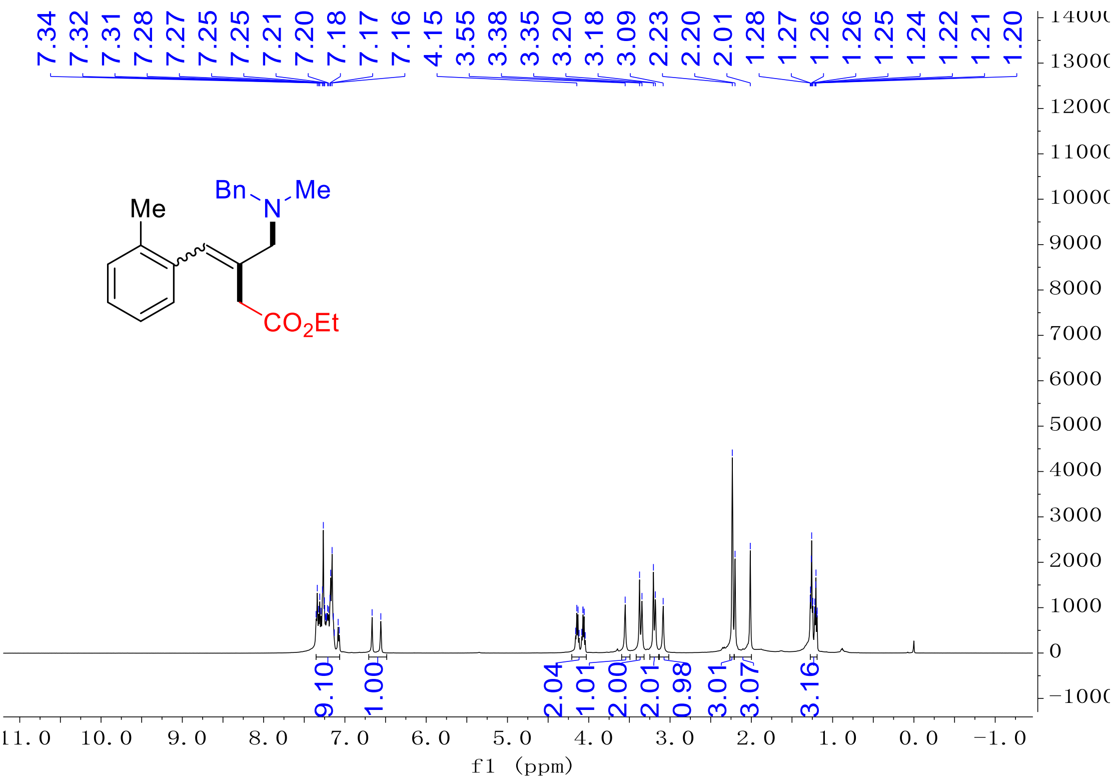

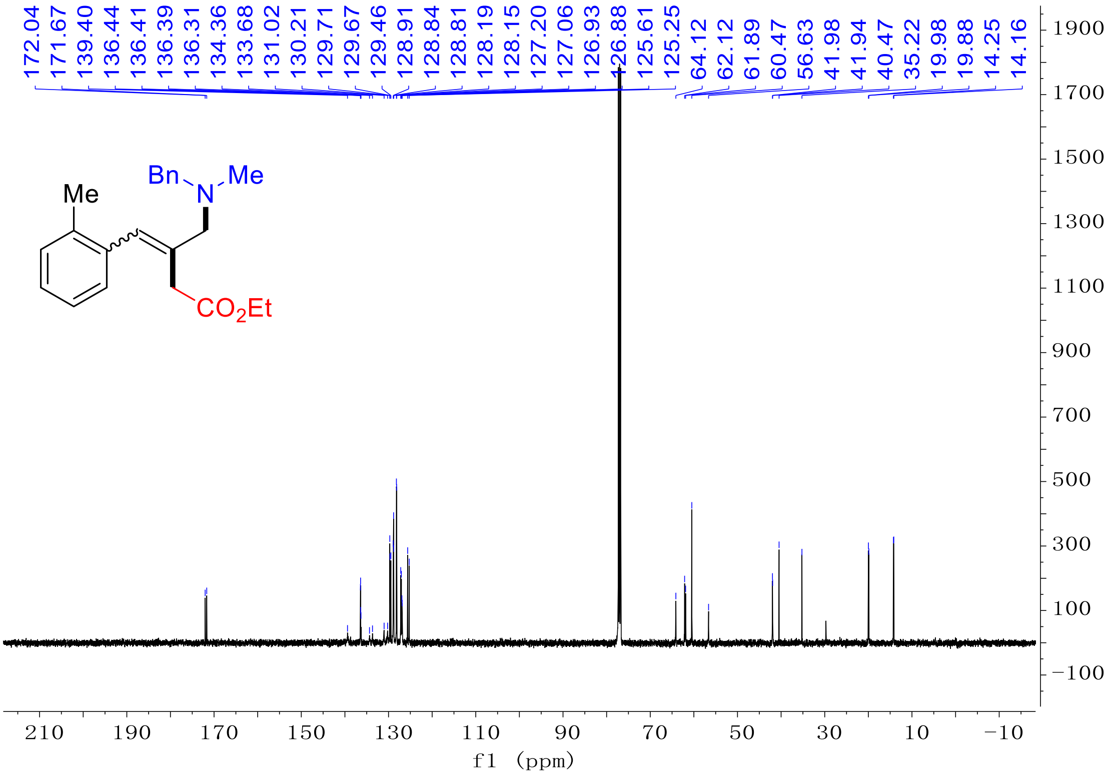

^{1}H NMR (500 MHz, CDCl$_3$) , ^{13}C NMR (126 MHz, CDCl$_3$) and ^{19}F NMR (376 MHz, CDCl$_3$) spectra for product 6q

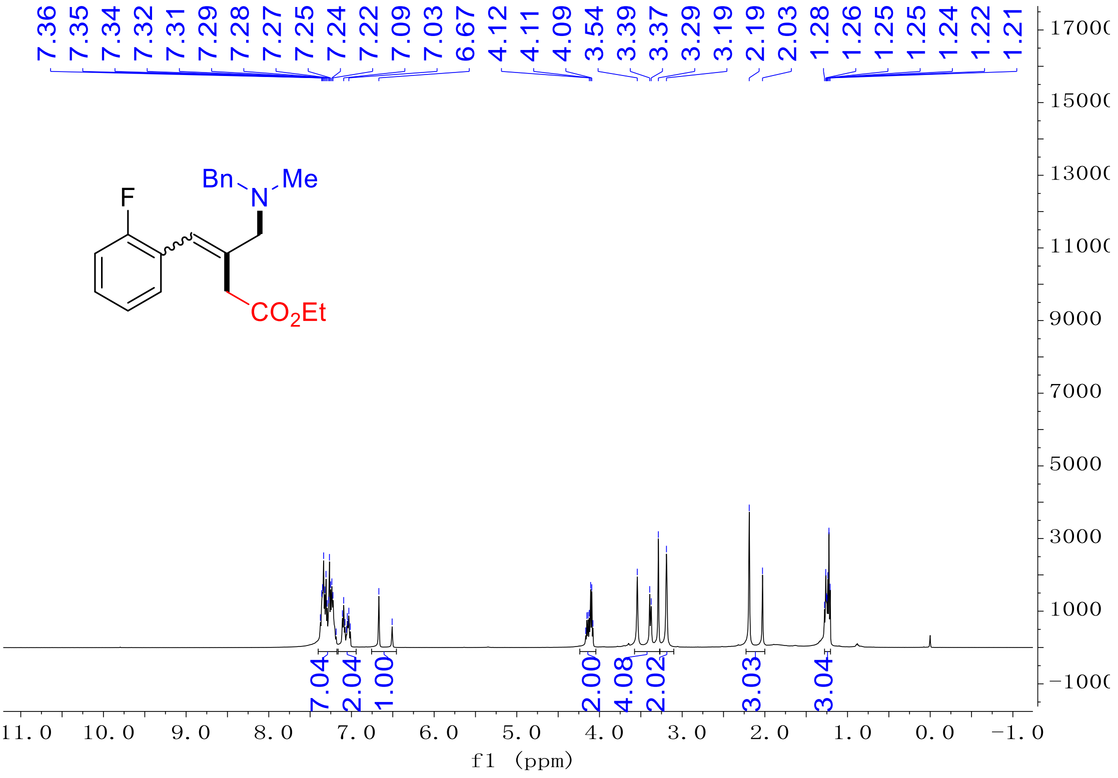

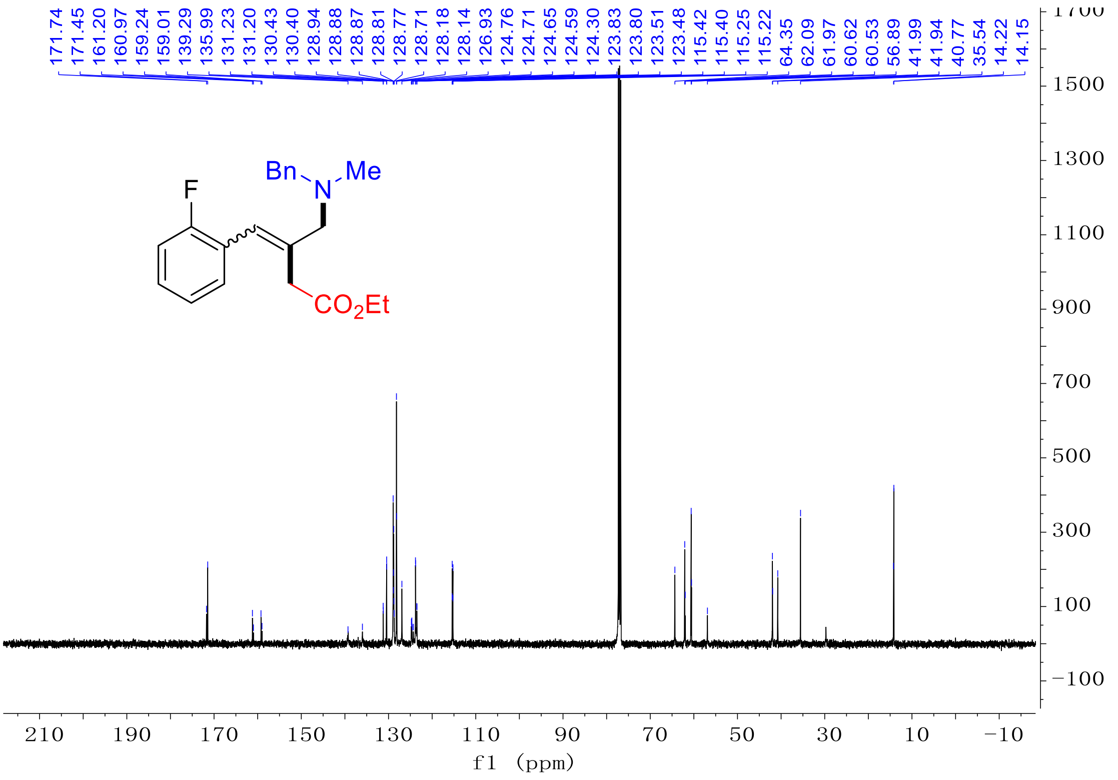

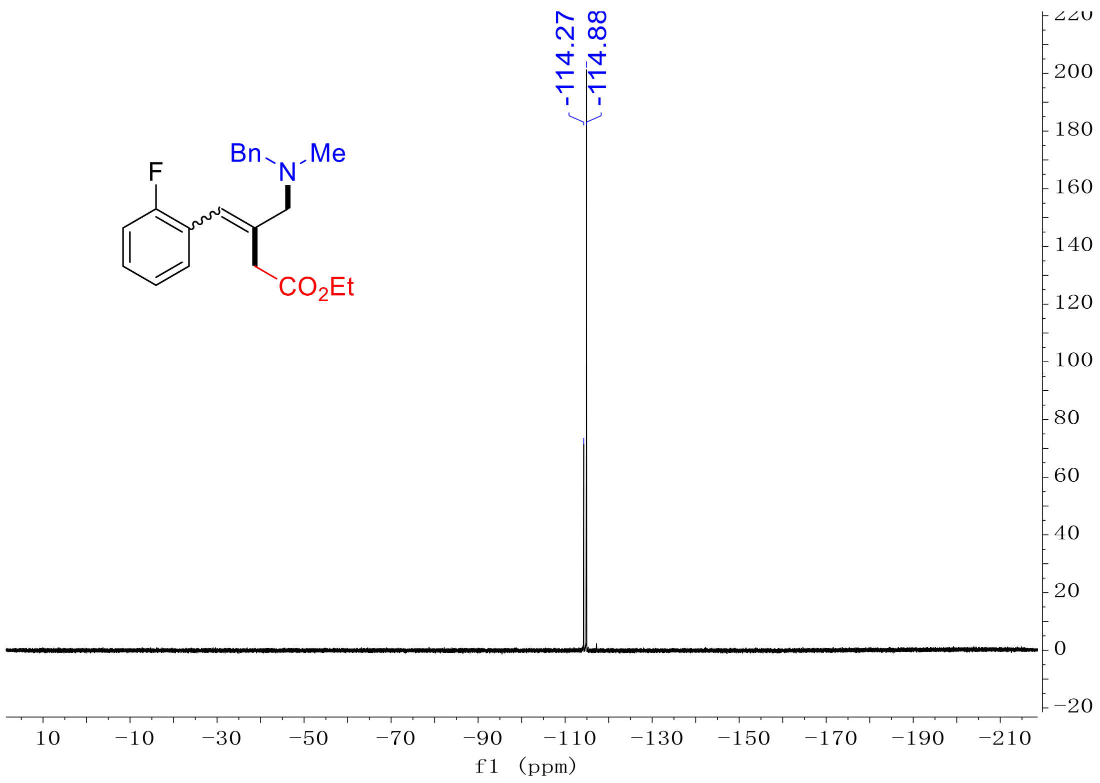
-114.27
-114.88
F
Bn
N
Me
CO2Et
220
200
180
160
140
120
100
80
60
40
20
0
−20
10
−10
−30
−50
−70
−90
−110
−130
−150
−170
−190
−210
f1 (ppm)

^{1}H NMR (500 MHz, CDCl3) and ^{13}C NMR (126 MHz, CDCl3) spectra for product 6r

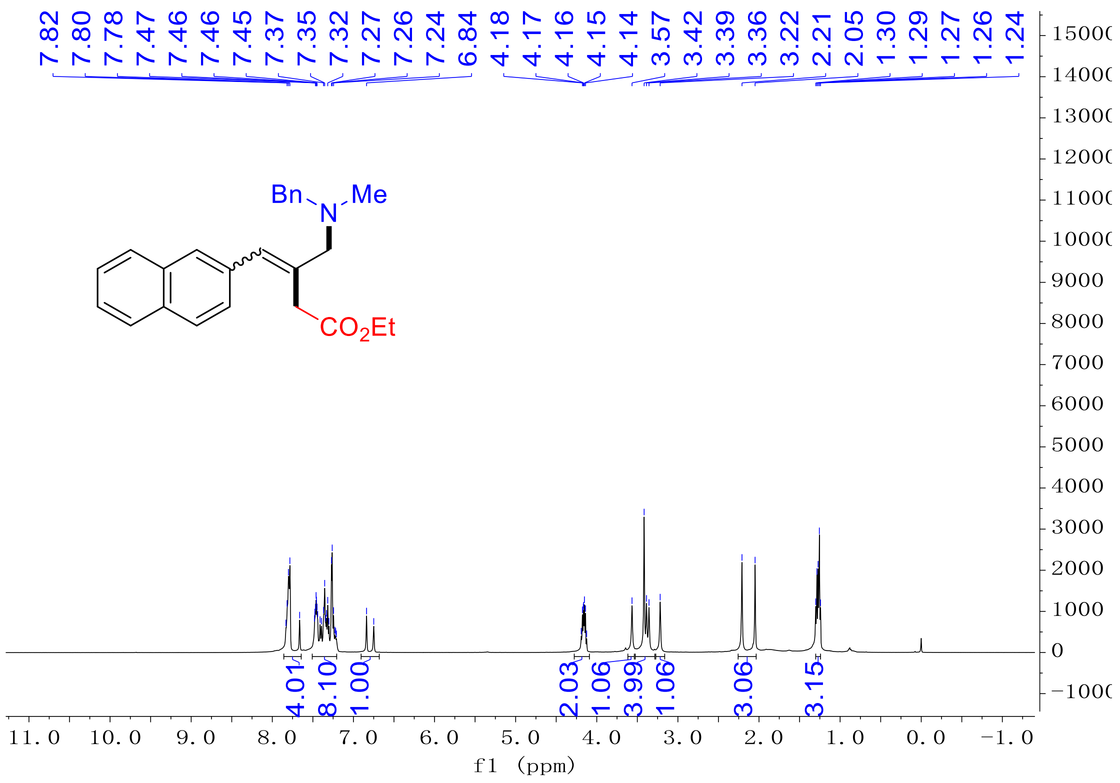

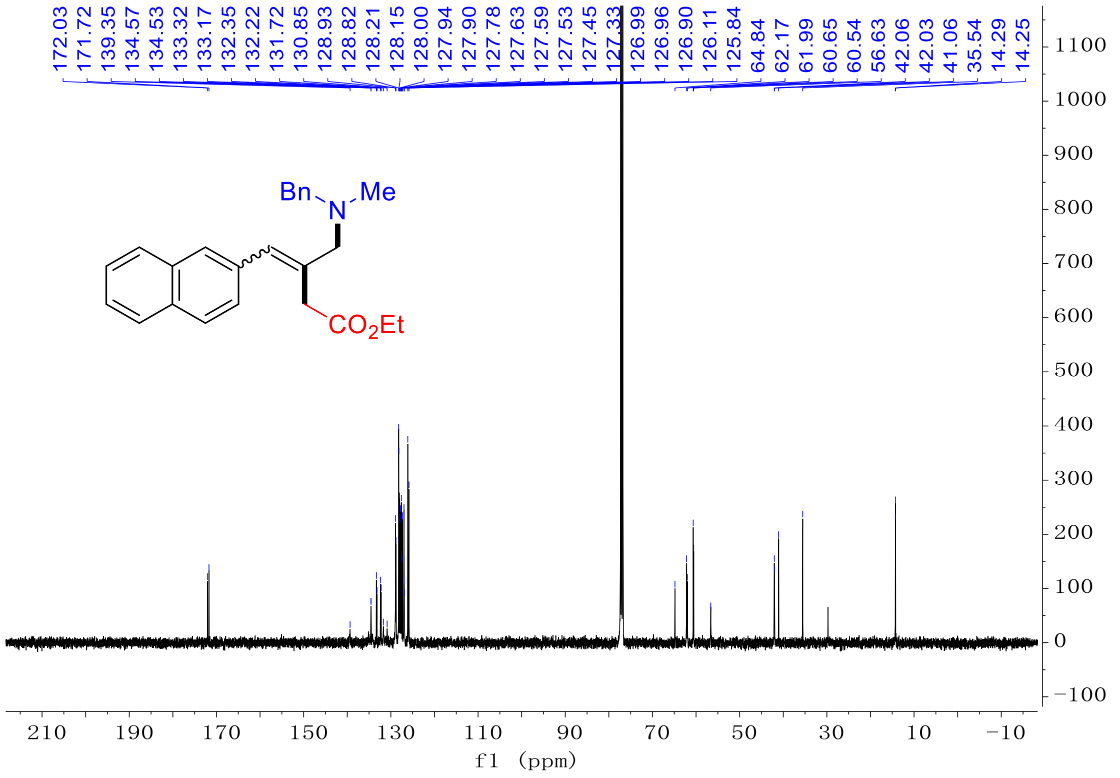

^{1}H NMR (400 MHz, CDCl3) and ^{13}C NMR (101 MHz, CDCl3) spectra for product 6s

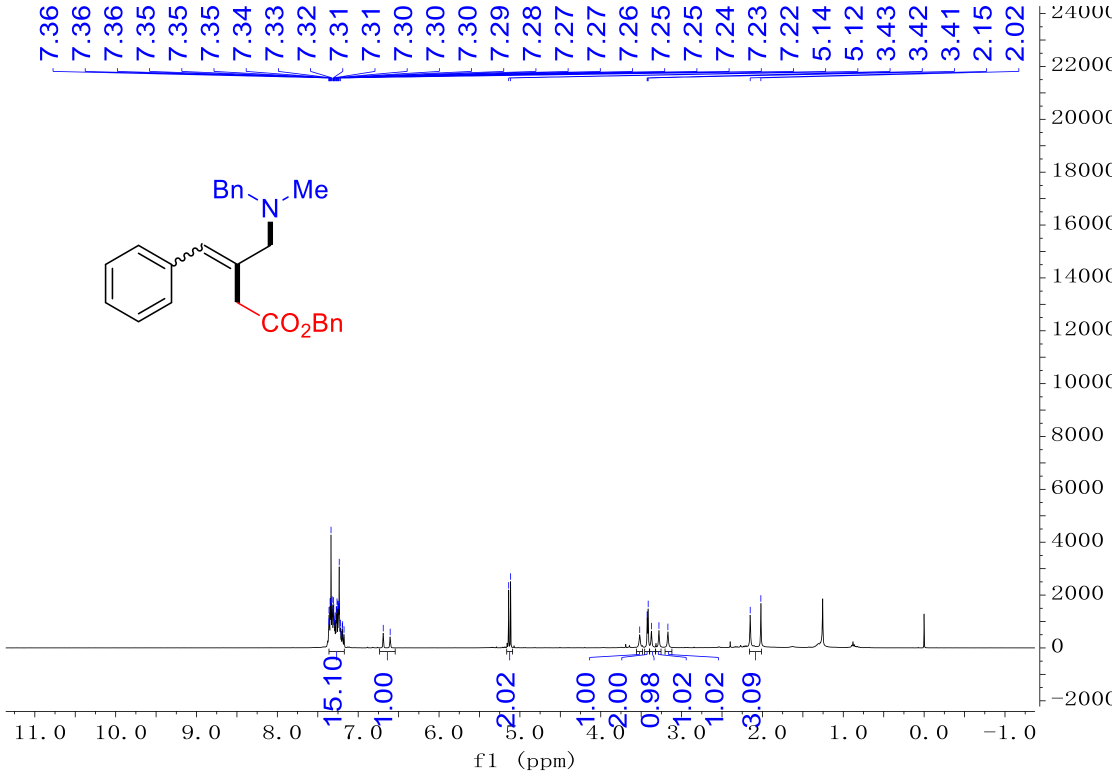

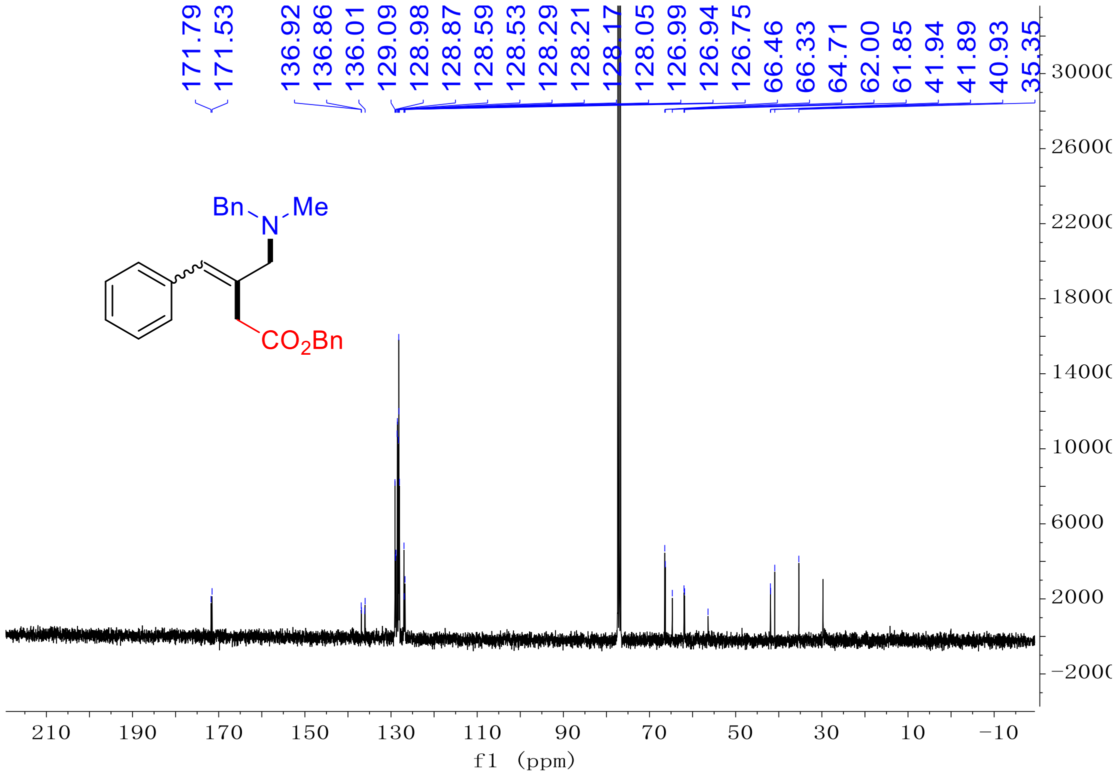

^{1}H NMR (500 MHz, CDCl3) and ^{13}C NMR (126 MHz, CDCl3) spectra for product 6t

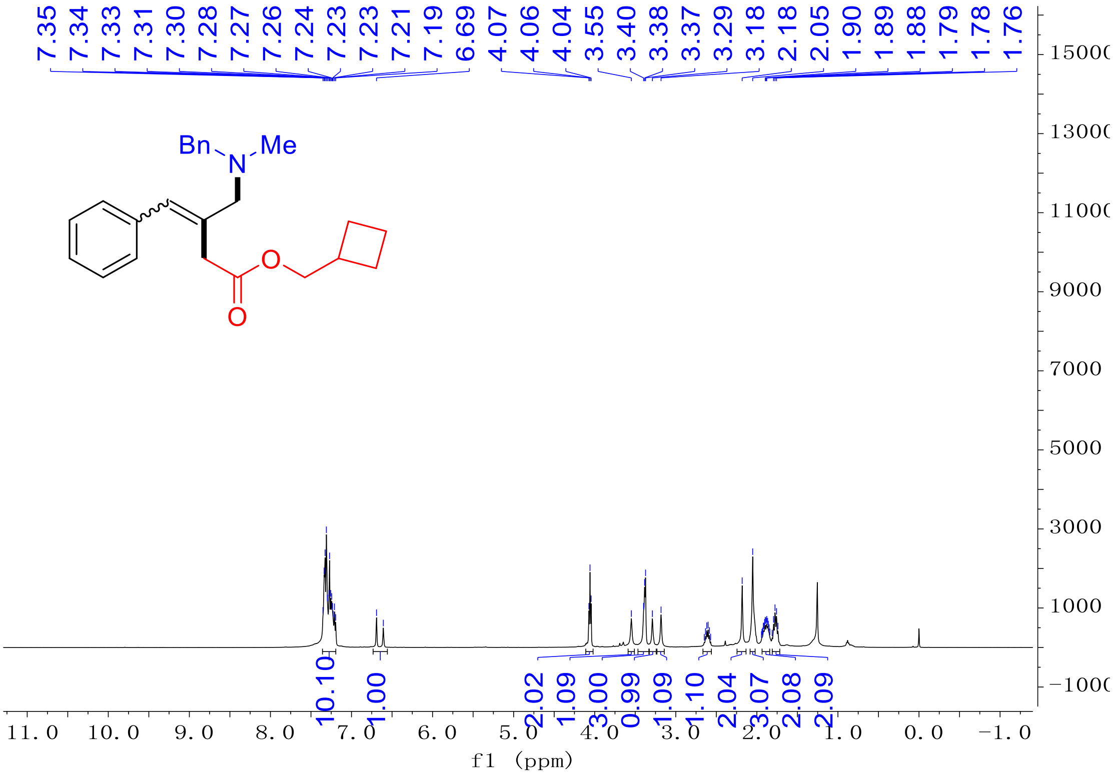

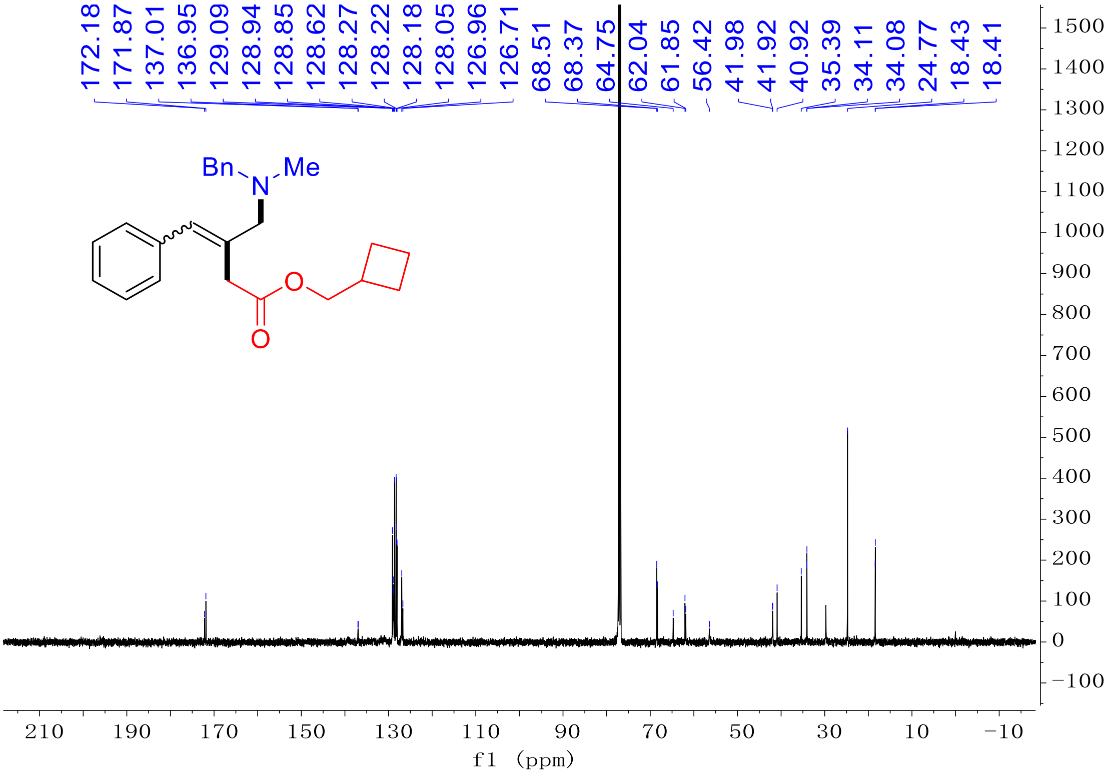

^{1}H NMR (400 MHz, $CDCl_3$) and ^{13}C NMR (101 MHz, $CDCl_3$) spectra for product 6u

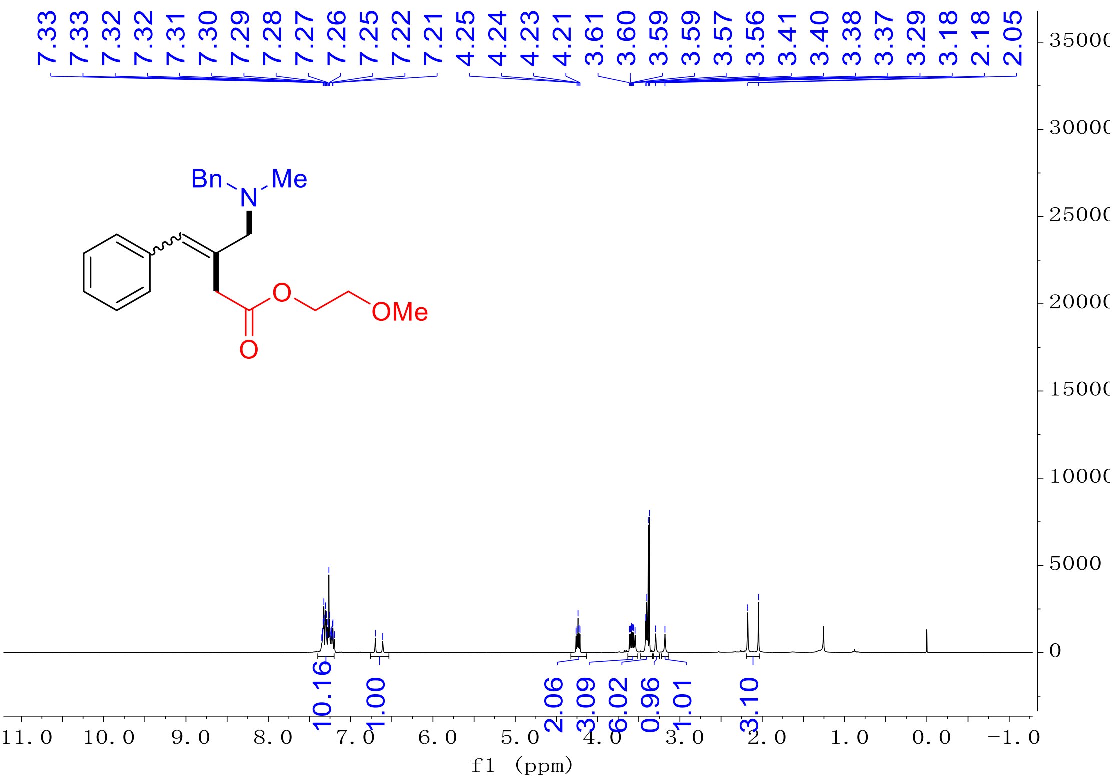

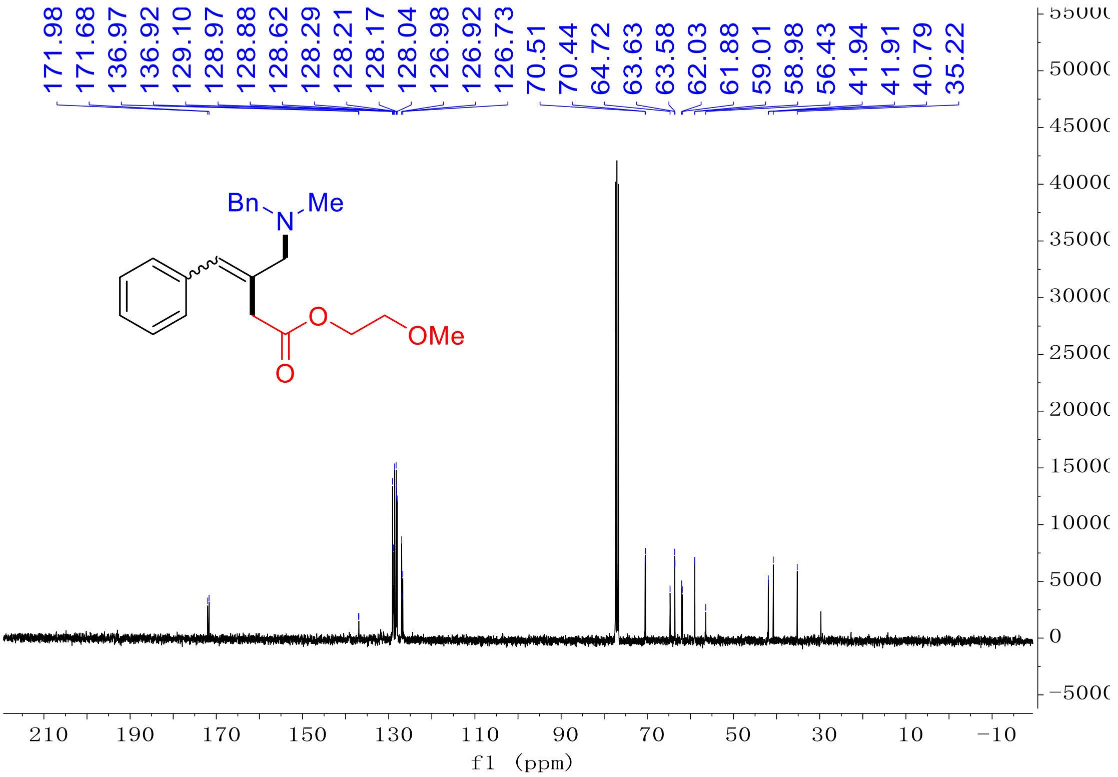

^{1}H NMR (500 MHz, $CDCl_3$) and ^{13}C NMR (126 MHz, $CDCl_3$) spectra for product 6v

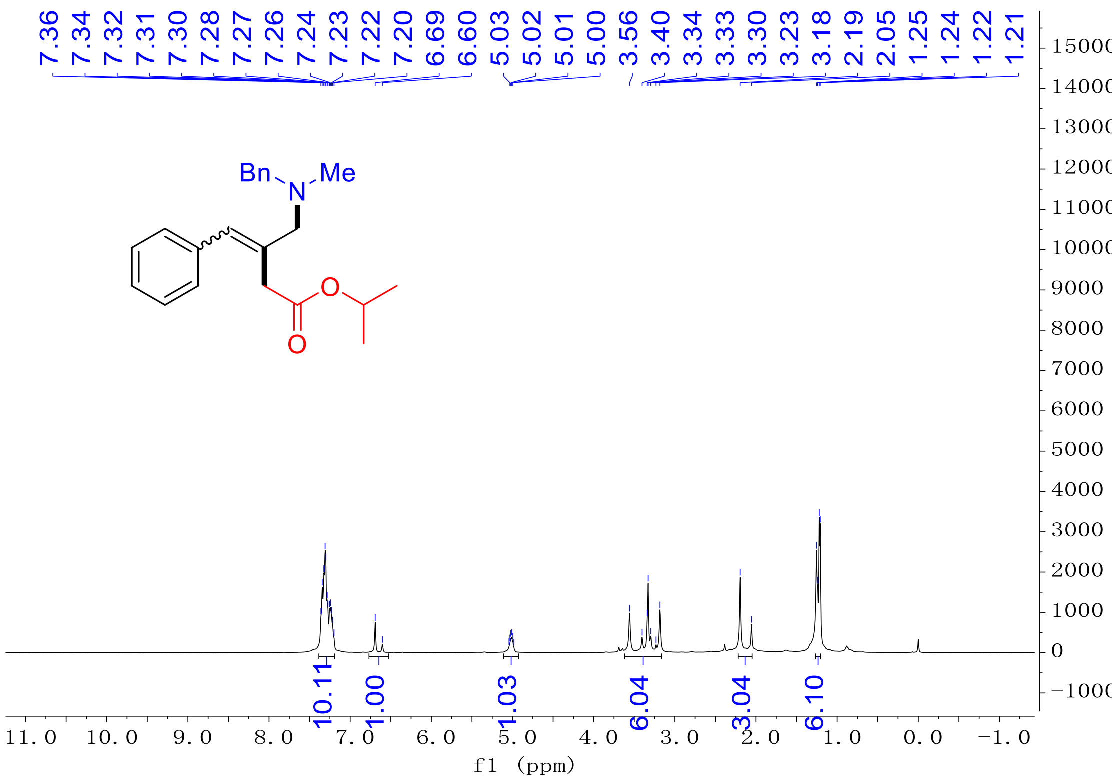

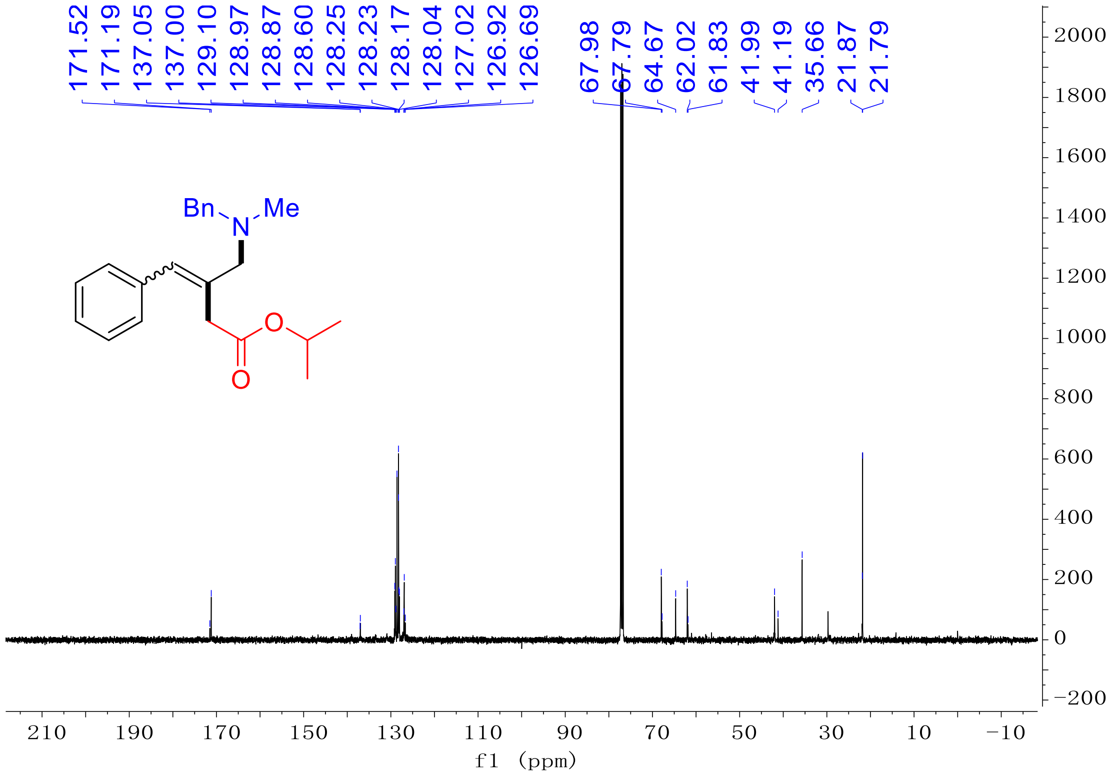

^{1}H NMR (500 MHz, $CDCl_3$) and ^{13}C NMR (126 MHz, $CDCl_3$) spectra for product 6w

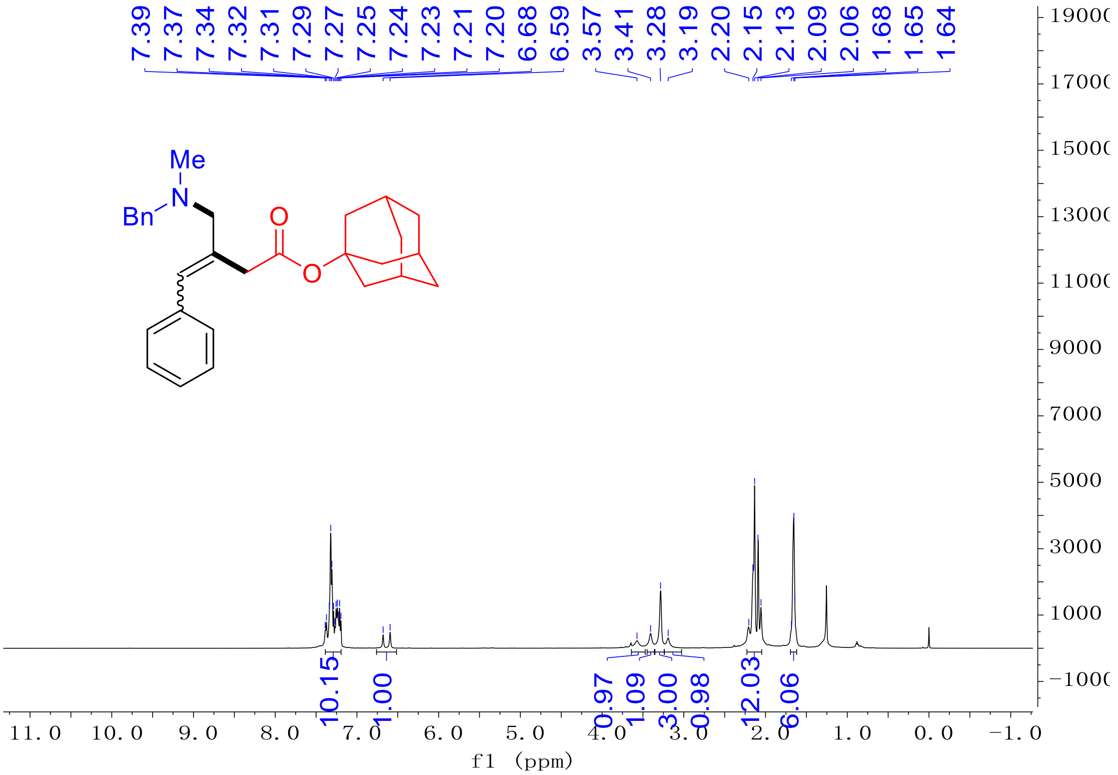

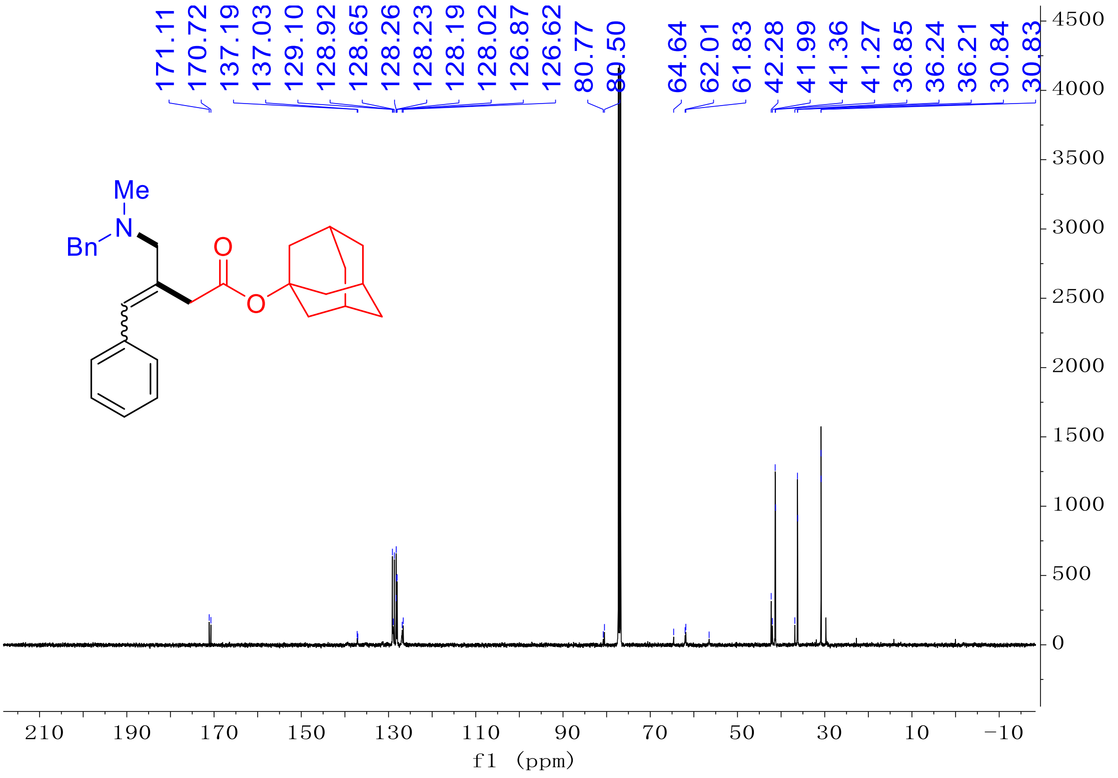

^{1}H NMR (500 MHz, CDCl3) and ^{13}C NMR (101 MHz, CDCl3) spectra for product 6x

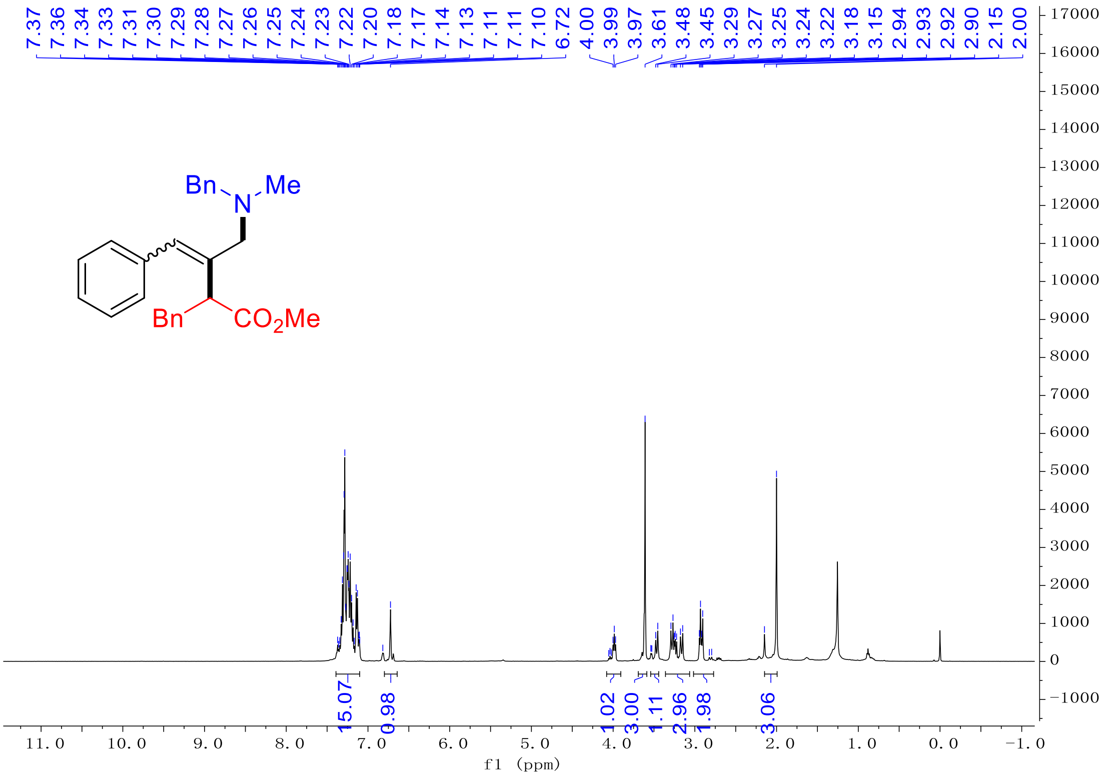

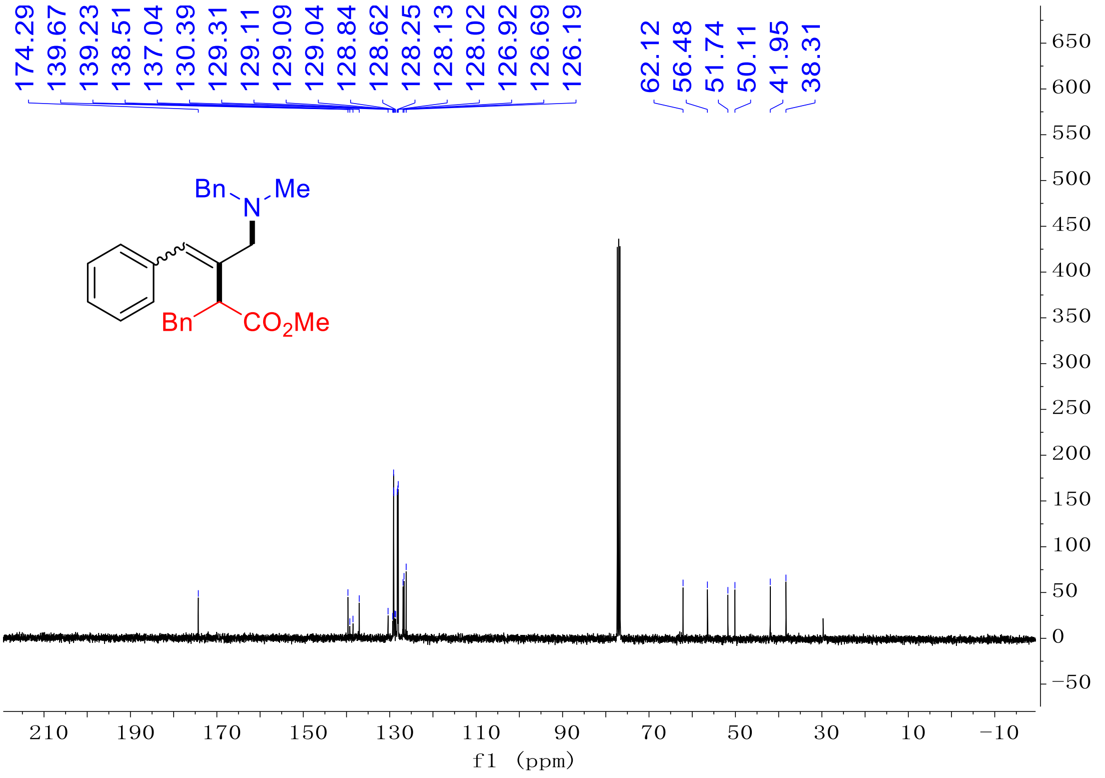

^{1}H NMR (400 MHz, CDCl3) and ^{13}C NMR (126 MHz, CDCl3) spectra for product 6y

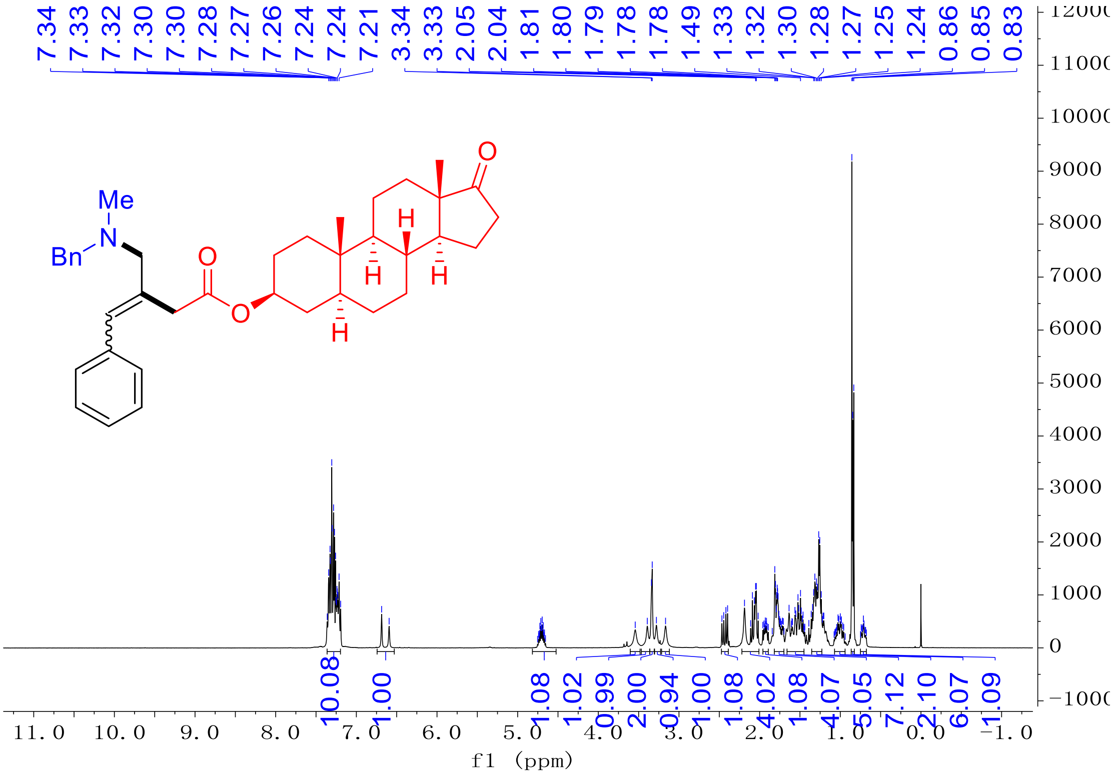

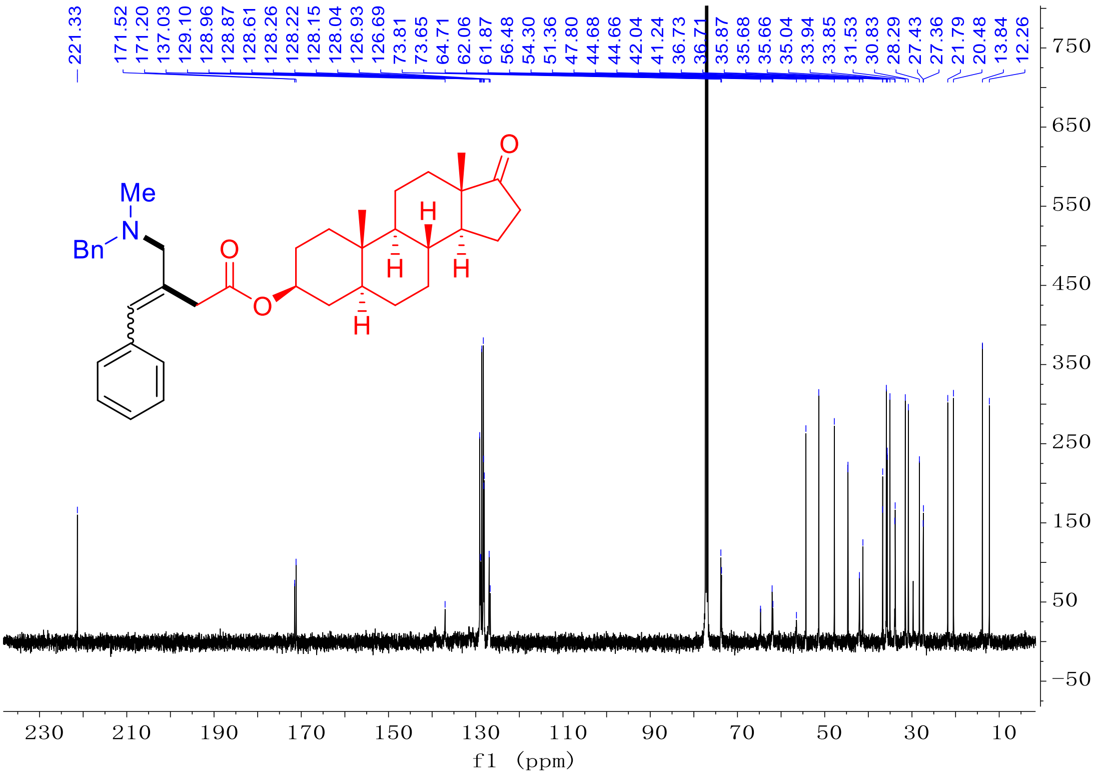

^{1}H NMR (500 MHz, CDCl$_3$) and ^{13}C NMR (126 MHz, CDCl$_3$) spectra for product 6z

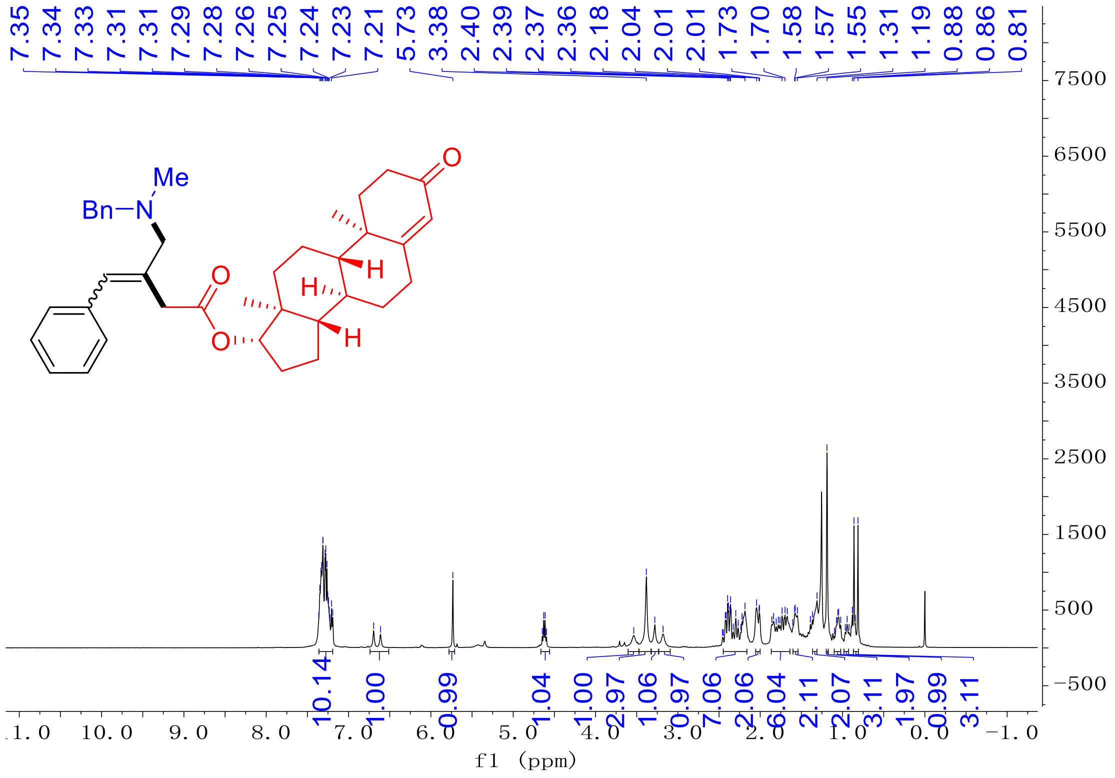

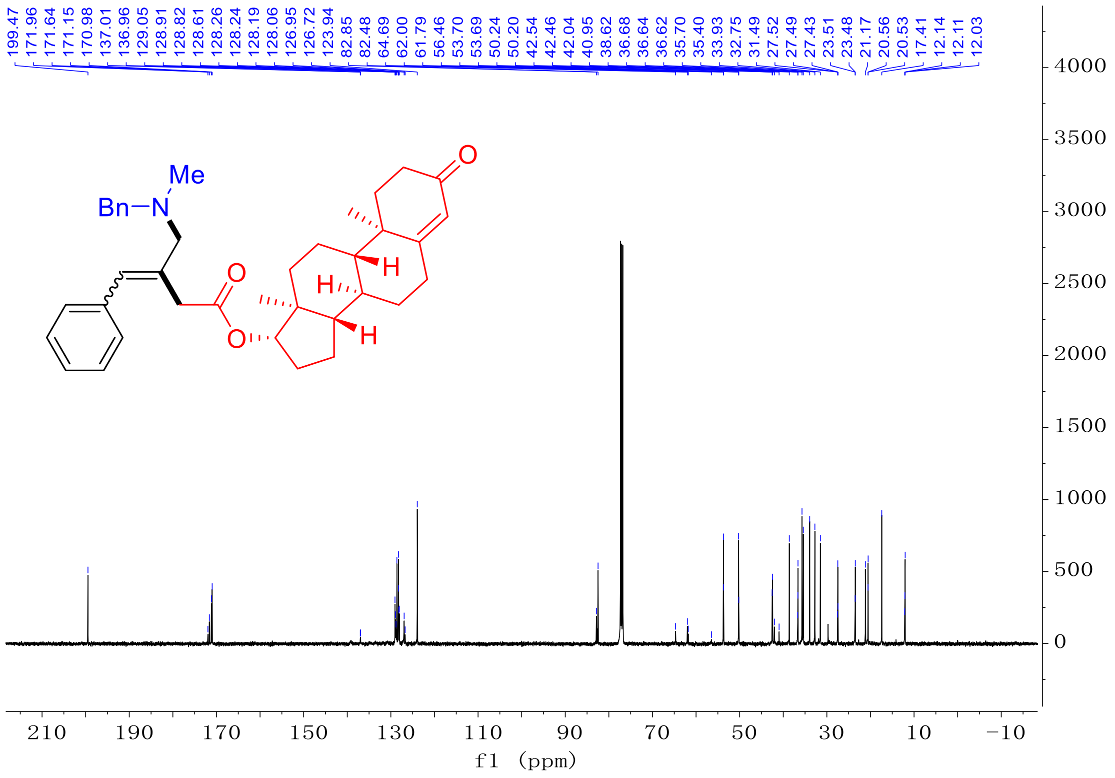

^{1}H NMR (400 MHz, $CDCl_3$) and ^{13}C NMR (101 MHz, $CDCl_3$) spectra for product 7

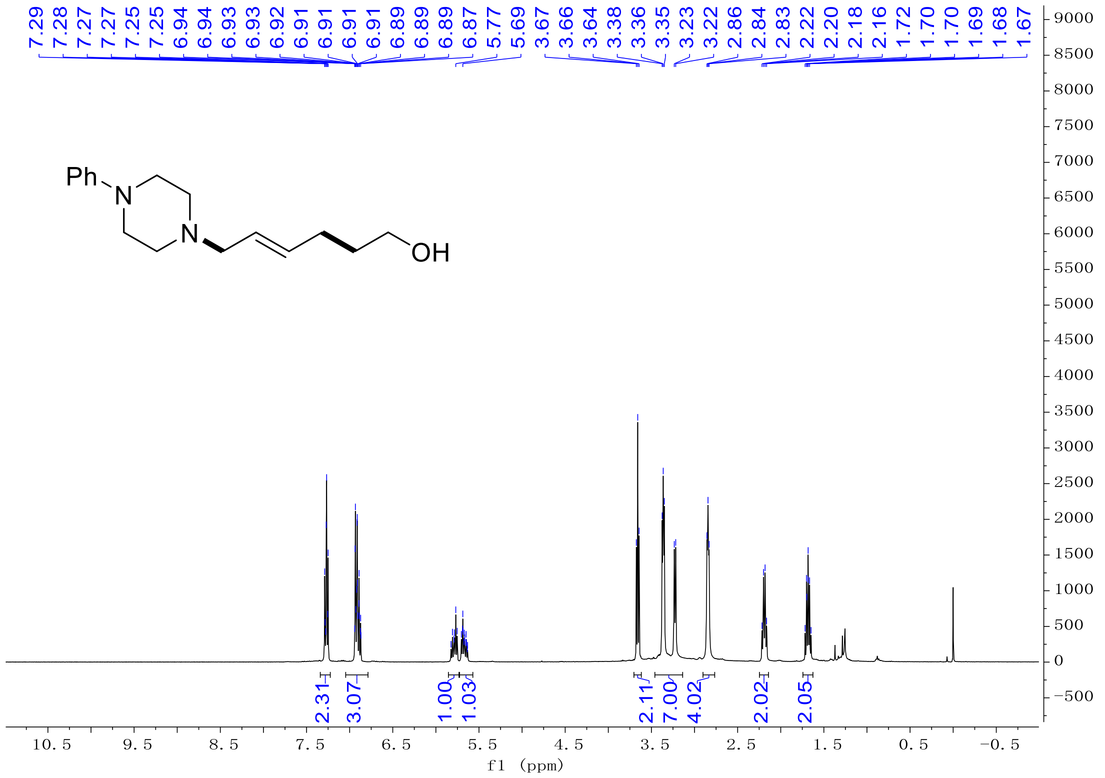

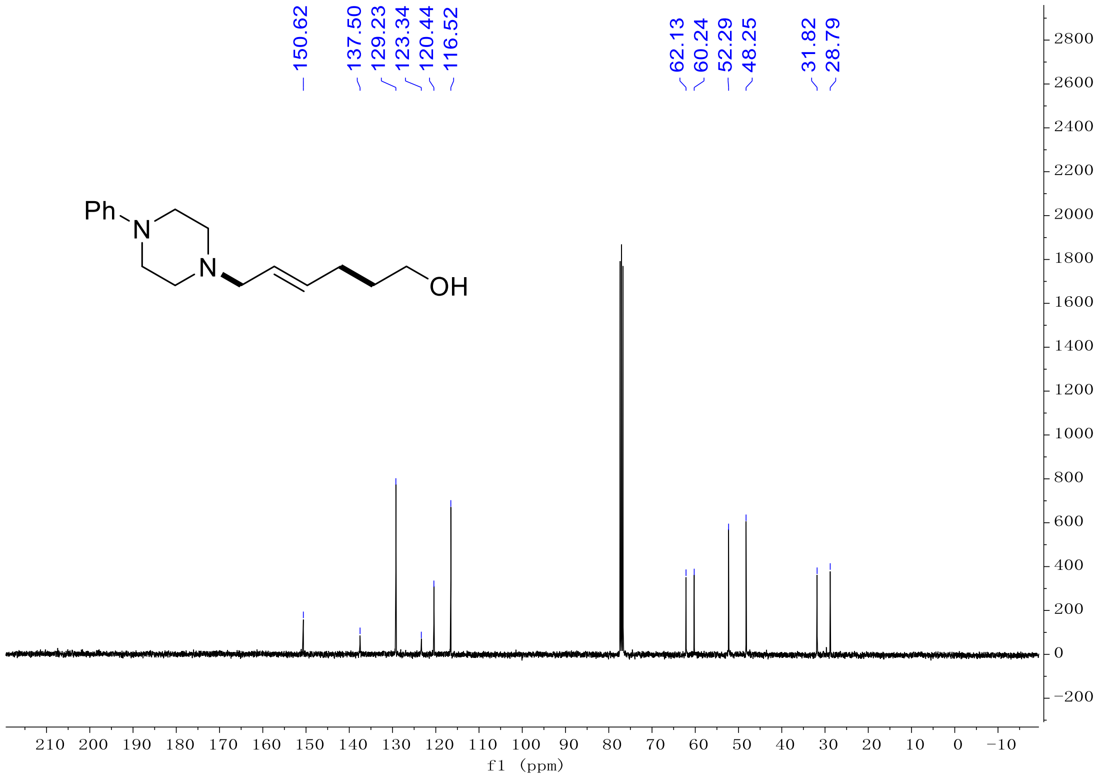

^{1}H NMR (400 MHz, $CDCl_3$) and ^{13}C NMR (126 MHz, $CDCl_3$) spectra for product 8

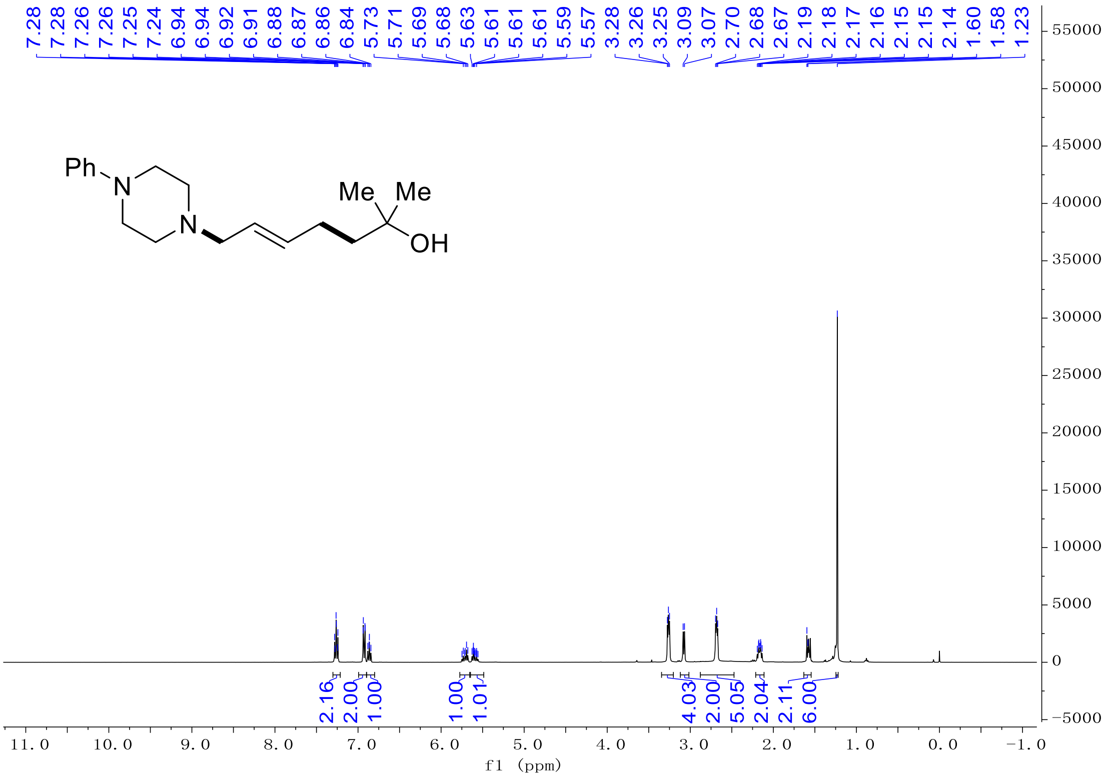

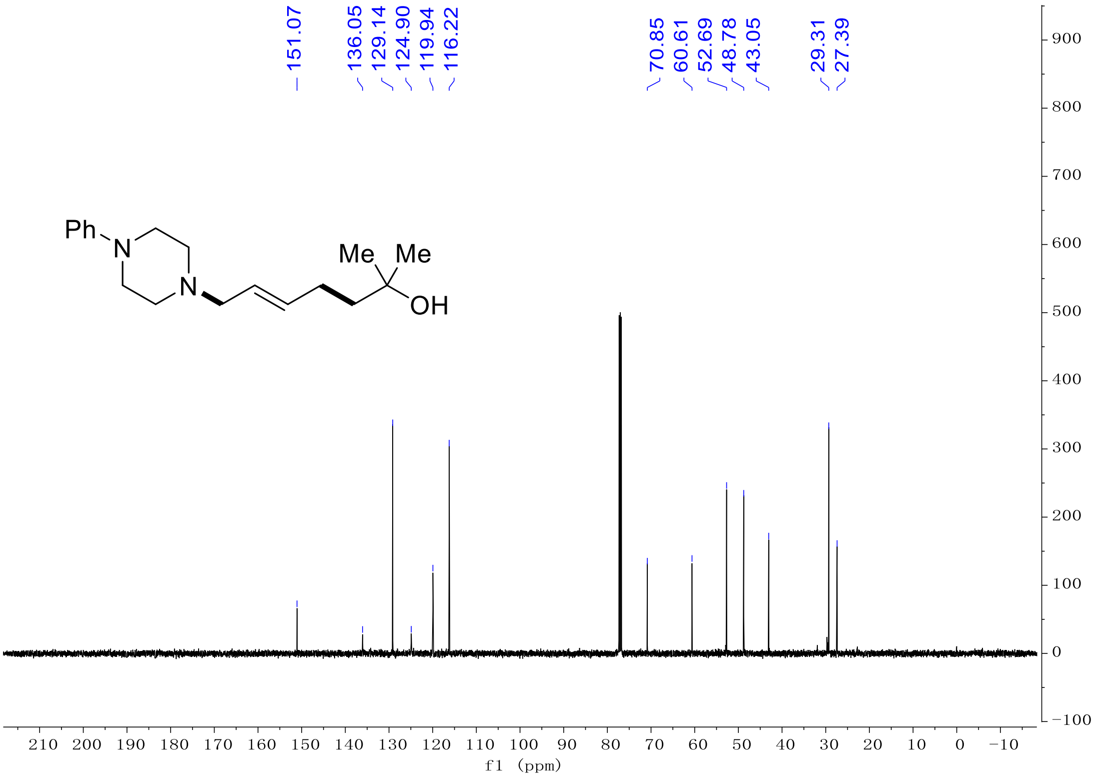

^{1}H NMR (500 MHz, CDCl3) and ^{13}C NMR (126 MHz, CDCl3) spectra for product 9

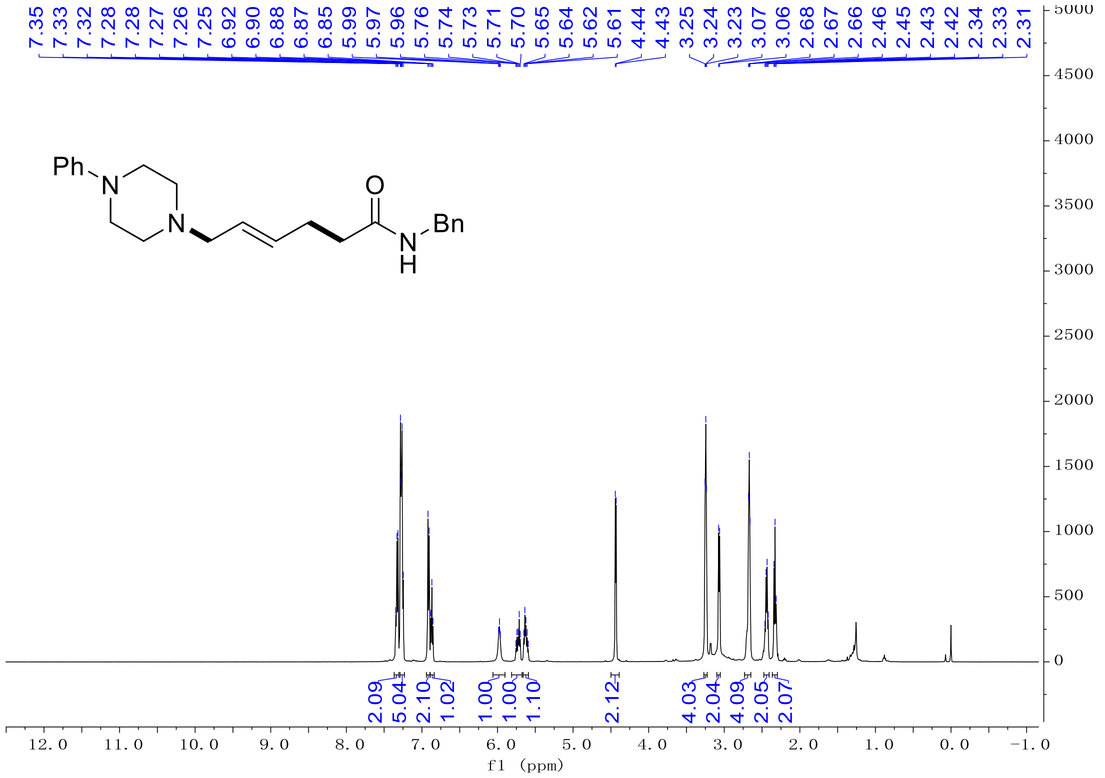

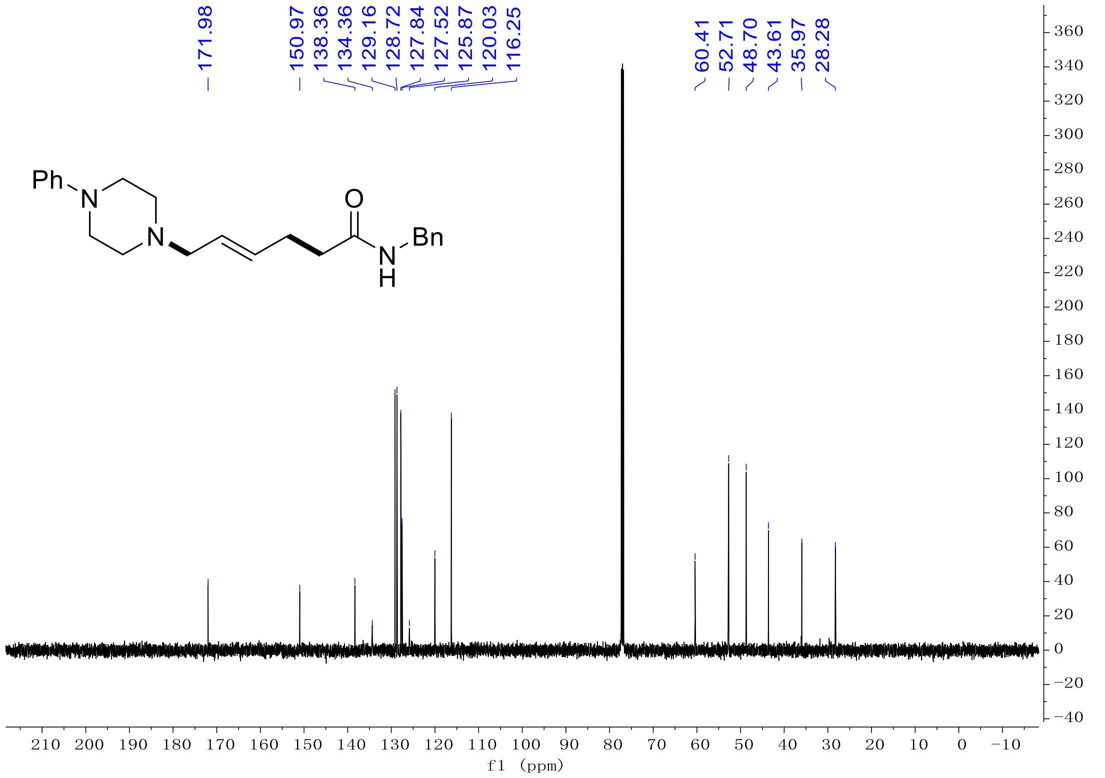

^{1}H NMR (400 MHz, CDCl3) and ^{13}C NMR (126 MHz, CDCl3) spectra for product 10

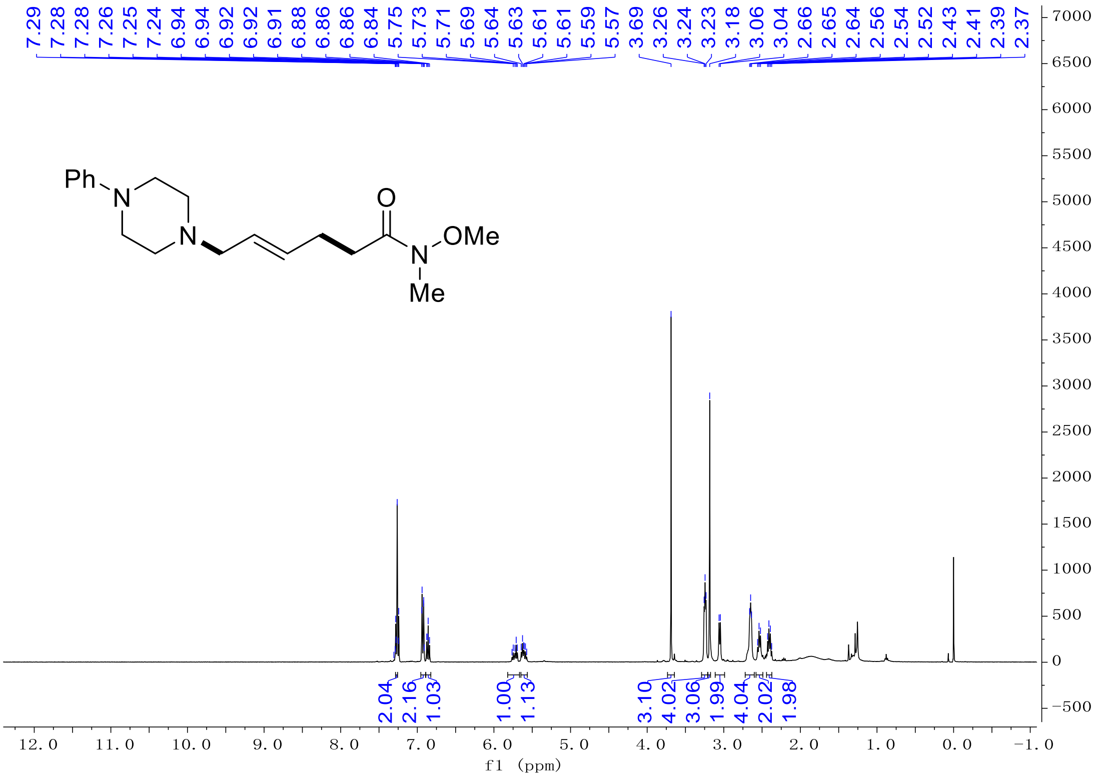

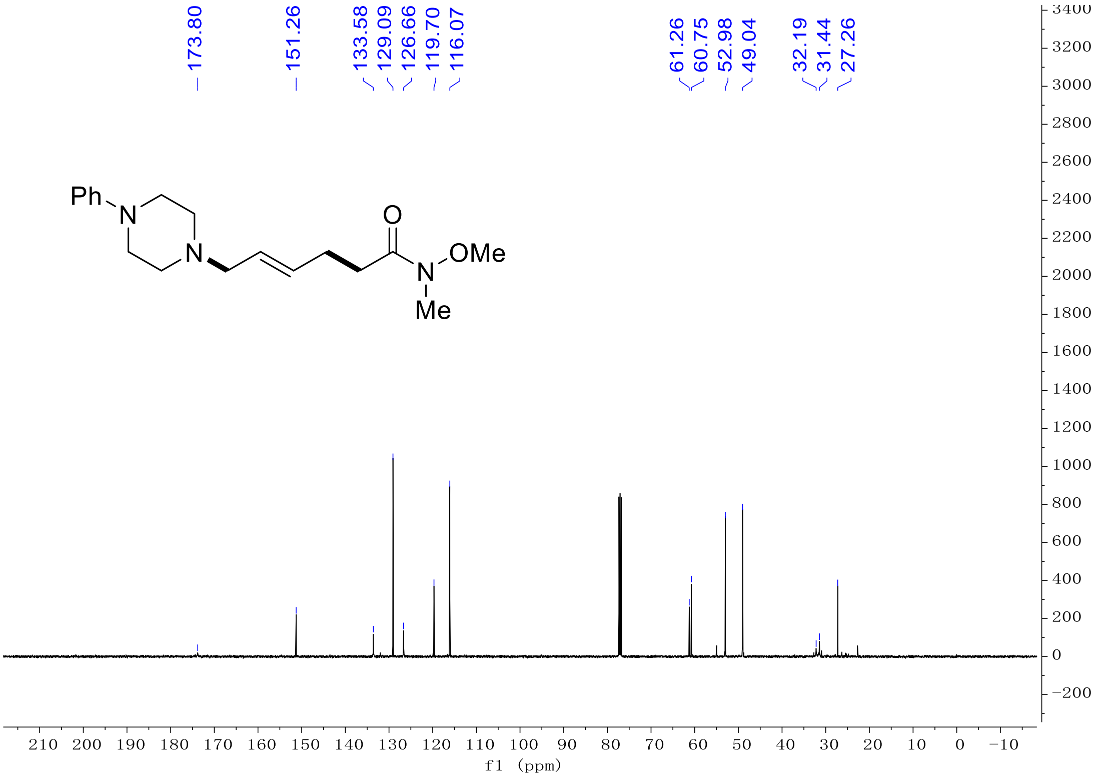

^{1}H NMR (500 MHz, $CDCl_3$) and ^{13}C NMR (126 MHz, $CDCl_3$) spectra for product 11

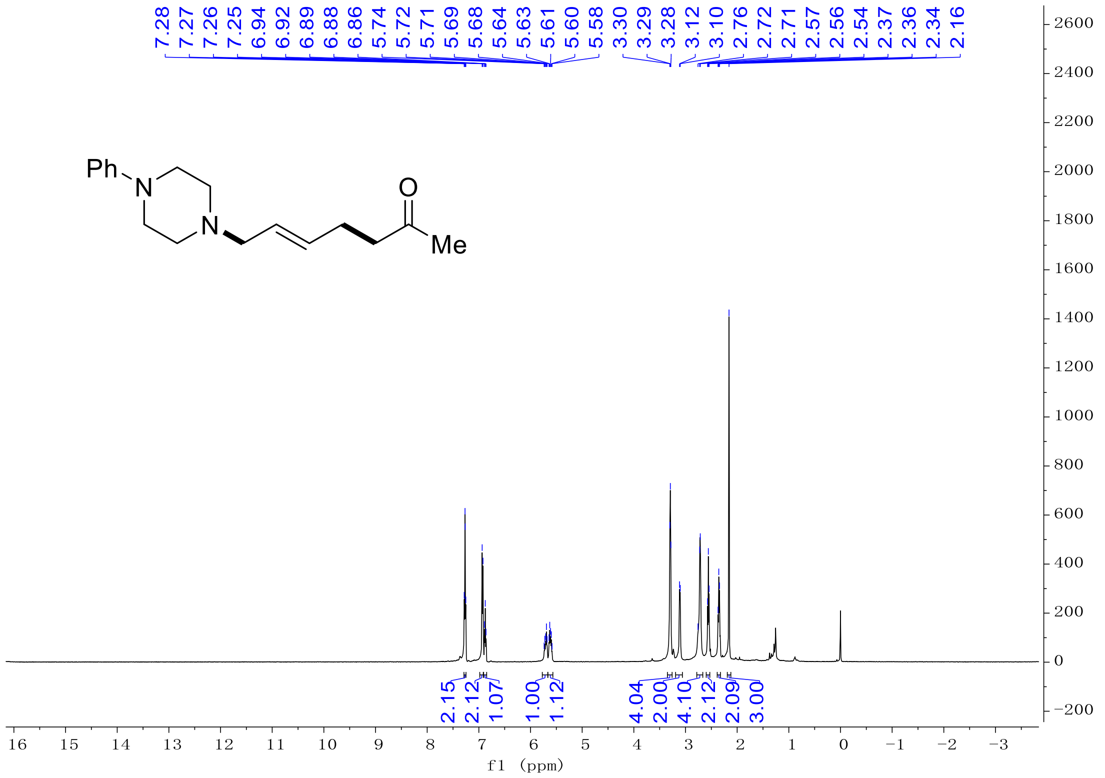

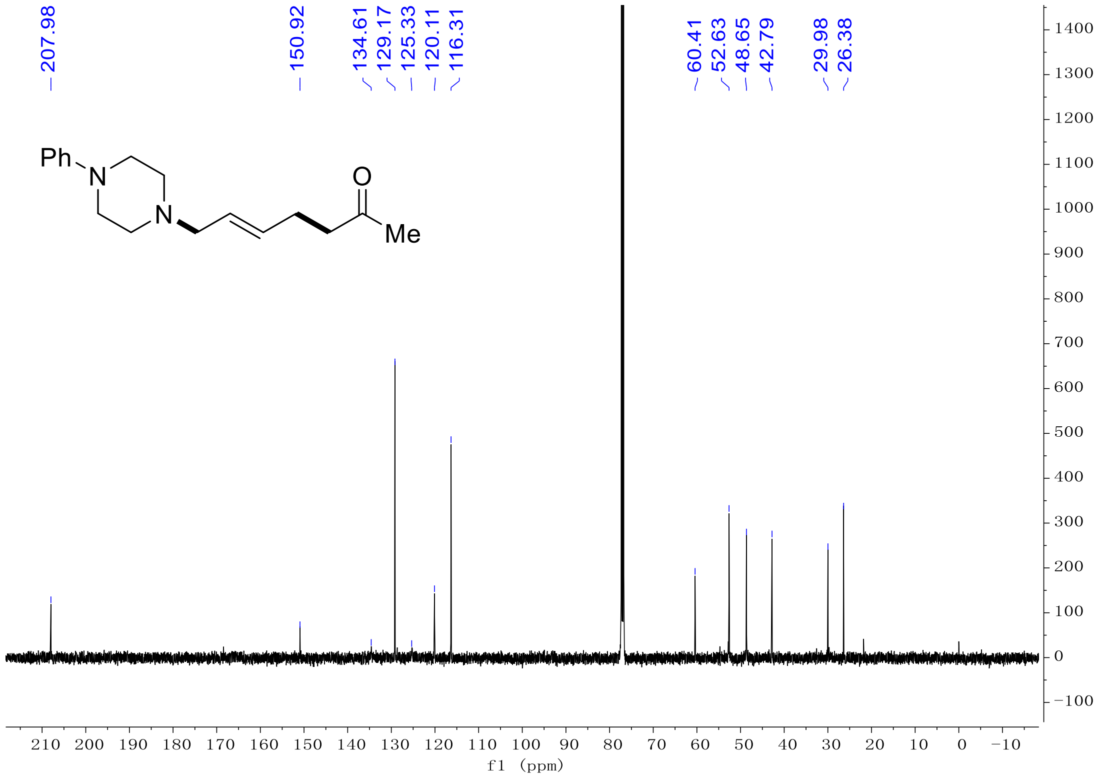

^{1}H NMR (400 MHz, $CDCl_3$) and ^{13}C NMR (101 MHz, $CDCl_3$) spectra for product 12

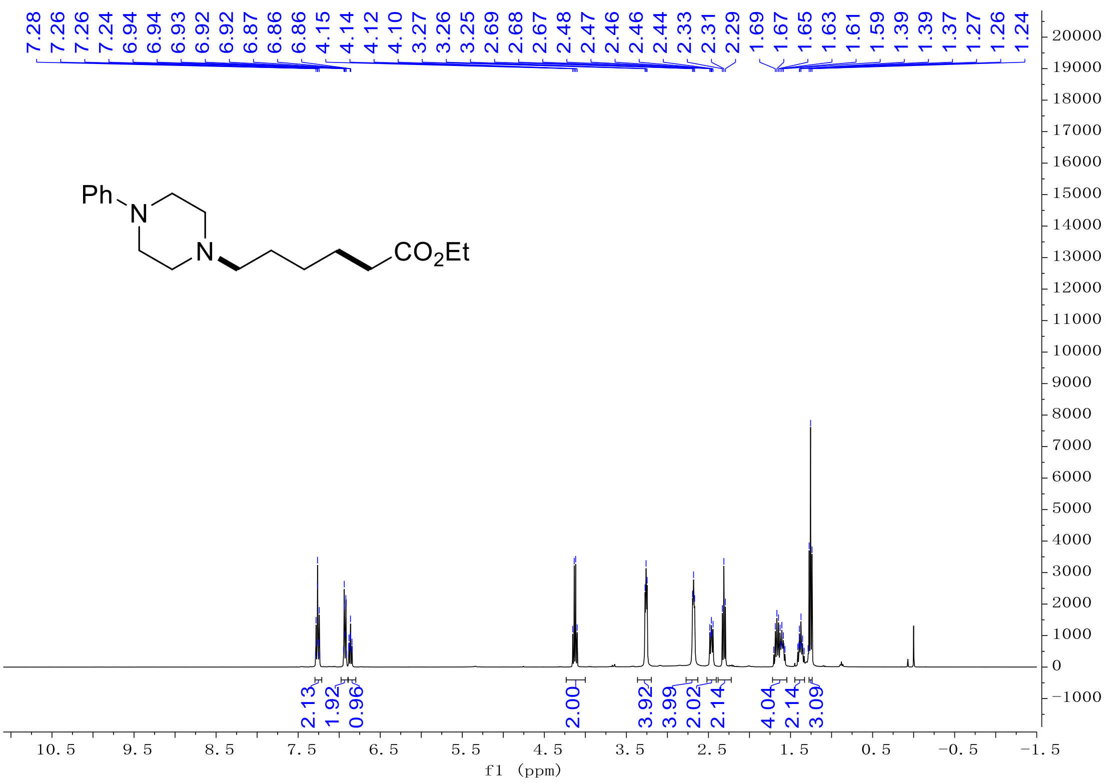

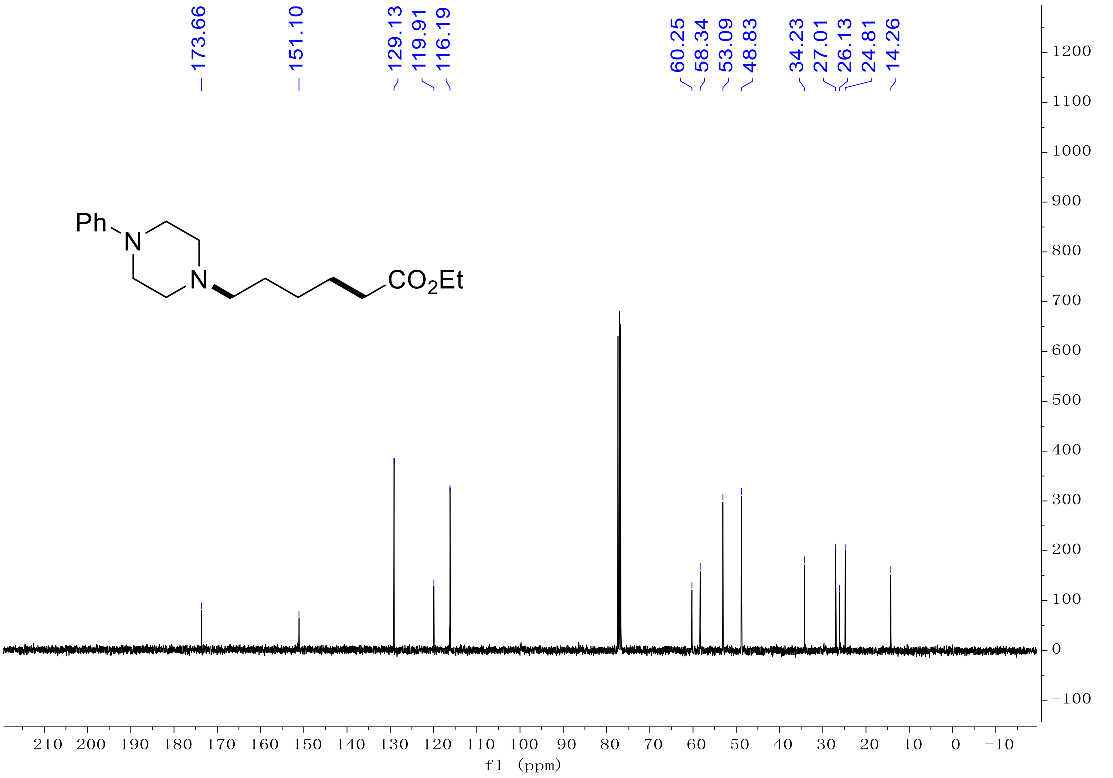

^{1}H NMR (400 MHz, CDCl3) and ^{13}C NMR (101 MHz, CDCl3) spectra for product 13

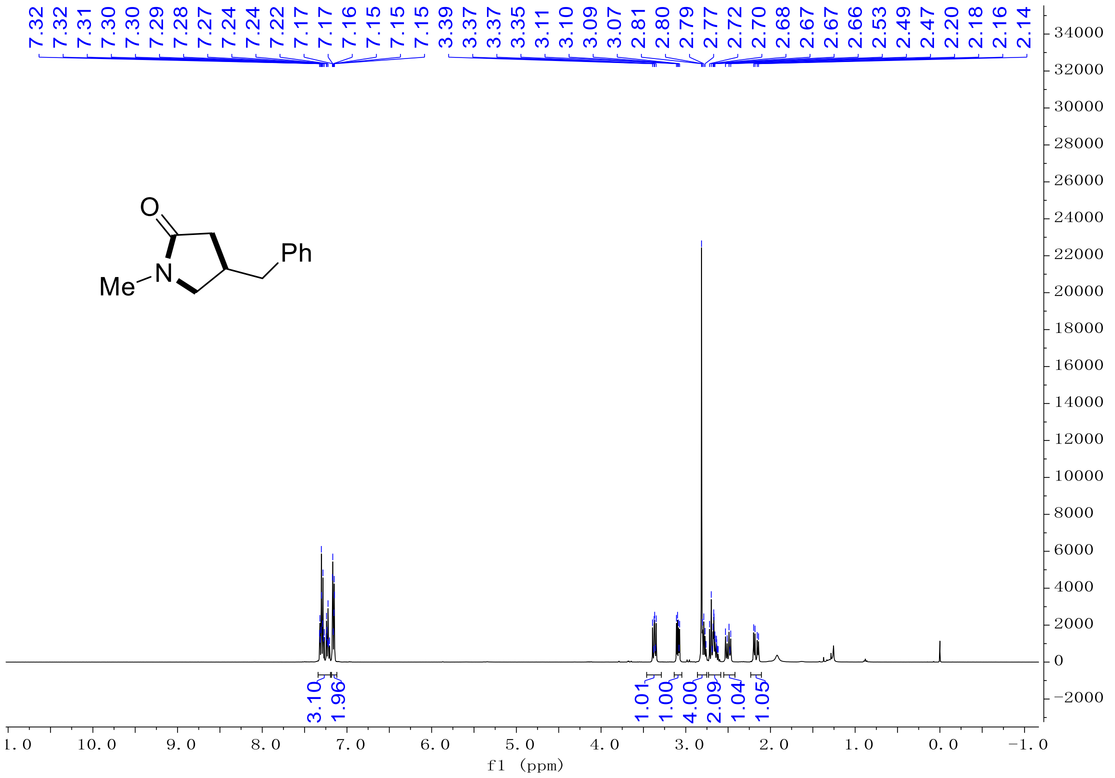

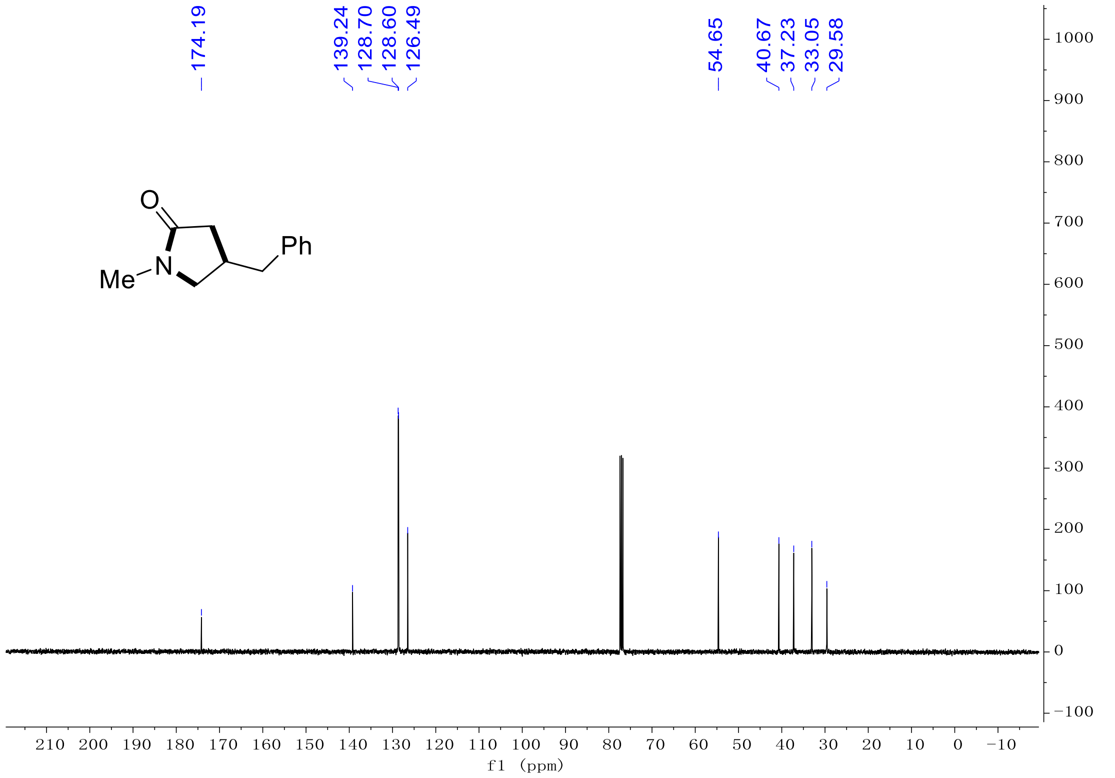

Supplement: File 1 — Full experimental details, analytical data and NMR spetra. [file Beilstein_J_Org_Chem-20-661-s001.pdf]
